# The prognostic genes model of breast cancer drug resistance based on single-cell sequencing analysis and transcriptome analysis

**DOI:** 10.1007/s10238-024-01372-6

**Published:** 2024-05-25

**Authors:** Yao Liu, Lun Dong, Jing Ma, Linghui Chen, Liaoqiong Fang, Zhibiao Wang

**Affiliations:** 1 State Key Laboratory of Ultrasound in Medicine and Engineering, College of Biomedical Engineering, Chongqing Medical University, Chongqing, 400016 China; 2 Chongqing Key Laboratory of Biomedical Engineering, Chongqing Medical University, Chongqing, 400016 China; 3 Department of Endocrinology, The Second Affiliated Hospital, Chongqing Medical University, Chongqing, China; 4 National Engineering Research Center of Ultrasound Medicine, Chongqing, 401121 China

**Keywords:** Breast cancer, Exosomes, Drug resistance, Biomarkers, Immunoinfiltration, Prognostic model

## Abstract

Breast cancer (BC) represents a multifaceted malignancy, with escalating incidence and mortality rates annually. Chemotherapy stands as an indispensable approach for treating breast cancer, yet drug resistance poses a formidable challenge. Through transcriptome data analysis, we have identified two sets of genes exhibiting differential expression in this context. Furthermore, we have confirmed the overlap between these genes and those associated with exosomes, which were subsequently validated in cell lines. The investigation screened the identified genes to determine prognostic markers for BC and utilized them to formulate a prognostic model. The disparities in prognosis and immunity between the high- and low-risk groups were validated using the test dataset. We have discerned different BC subtypes based on the expression levels of prognostic genes in BC samples. Variations in prognosis, immunity, and drug sensitivity among distinct subtypes were examined. Leveraging data from single-cell sequencing and prognostic gene expression, the AUCell algorithm was employed to score individual cell clusters and analyze the pathways implicated in high-scoring groups. Prognostic genes (CCT4, CXCL13, MTDH, PSMD2, and RAB27A) were subsewoquently validated using RT-qPCR. Consequently, we have established a model for predicting prognosis in breast cancer that hinges on drug resistance and ERGs. Furthermore, we have evaluated the prognostic value of this model. The genes identified as prognostic markers can now serve as a reference for precise treatment of this condition.

**Supplementary Information:**

The online version contains supplementary material available at 10.1007/s10238-024-01372-6.

## Introduction

Breast cancer (BC), a pervasive malignancy worldwide, is witnessing an increase in both incidence and mortality rates [1]. This trend underscores the necessity for effective BC management strategies, including prompt diagnosis, precise treatment, and notably, chemotherapy. Although chemotherapy remains a cornerstone of BC treatment, leading to tumor shrinkage and potential cures, it is increasingly impeded by the emergence of drug resistance in various BC subtypes [2]. This resistance not only diminishes the effectiveness of chemotherapy but also complicates the anti-tumor treatment process. Therefore, it is crucial to investigate the underlying mechanisms of resistance.

Drug resistance can manifest in various subtypes of BC through diverse mechanisms. For example, certain genes are commonly or differentially expressed across BC types and might impact drug resistance [3, 4]. Research has concluded that the overexpression of the human epidermal growth factor receptor (HER-2) significantly affects BC prognosis. The use of trastuzumab has markedly improved patient outcomes in cases of HER-2 overexpression, while resistance to HER-2 targeted agents is increasing [5]. Furthermore, mutations in the estrogen receptor gene have been specifically linked to harm in metastatic BC [6]. These genetic elements contribute to the growing complexity of drug resistance at the molecular level, potentially leading to unmanageable BC events and increased fatality. It has been demonstrated that genes associated with drug resistance have a significant impact in this context [6]. For instance, PTX-induced exosomal circBACH1 stimulates the migration and stemness of BC cells, influencing drug resistance mechanisms by absorbing miR-217 to increase the expression of G3BP2 [7]. Moreover, inhibiting exosomal tRF-16-K8J7K1B enhances the sensitivity of BC cells to tamoxifen, highlighting the potential of exosomal pathways as targets to overcome drug resistance [8]. These findings suggest that a significant aspect of drug resistance mechanisms in BC may be present at the cellular level, especially within exosomes.

Exosomes possess a lipid bilayer structure and are typically sized between 30 and 150 nm. Initially, exosomes were believed to primarily serve as a mechanism for cellular waste disposal [9]. However, subsequent research has revealed their crucial role in both tumor cell proliferation and anti-tumor immunity [10, 11]. Exosomes also regulate the microenvironment between cells and the immune system by transporting bioactive molecules. They function as intercellular communicative vectors in the tumor microenvironment and play a pivotal role in the progression of BC [12]. Exosomes are instrumental in remodeling the tumor microenvironment, facilitating immune escape, and even contributing to drug resistance [13]. Exosomes transport functional proteins and noncoding RNAs that impact drug efflux, metabolism, pro-survival signaling, epithelial-mesenchymal transition, stem-like characteristics, and remodeling of the microenvironment in tumors [14]. Exosomes’ attributes have led to a significant focus on research to comprehend the progression of breast cancer and treatment resistance.

BC samples were obtained from the GEO cohort (https://www.ncbi.nlm.nih.gov/geo/), considering both drug resistance and the impact of exosome-associated genes (ERGs). The samples were categorized into drug-resistant and drug-sensitive groups. Differential expression analysis was carried out on both groups, followed by the intersection of the resulting differentially expressed genes (DEGs) with the previously compiled ERGs. Details of the gene.csv file can be found in the Supplementary Material. Univariate Cox regression and Lasso-Cox analyses were conducted on the gene intersection to identify prognostic genes and formulate a prognostic model. The prognostic genes and models were subsequently validated using BC samples from the GEO cohort. BC subtypes were determined based on the expression of the prognostic genes, and differences in prognosis and immunity among these subtypes were analyzed using the NMF method. The study performed a drug sensitivity analysis to compare IC50 values among different BC subtypes, revealing substantial differences among the drugs. Additionally, scRNA data from the GEO database were utilized to confirm the expression of prognostic genes in immune cells. High-scoring cell populations were analyzed using AUCell to identify significant pathways.

## Method

### Acquisition of data sets

We obtained transcriptome data from 224 BC samples, comprising 143 drug-resistant samples and 81 sensitive samples. These samples were sourced from the GSE163882 dataset, accessible in the GEO database (https://www.ncbi.nlm.nih.gov/geo/). Technical term abbreviations are explained upon first usage. The data were utilized to identify drug resistance-associated exosome genes (EGDR) by identifying overlaps between DEGs and genes associated with exosomes. For our study, we utilized 1,113 BC samples and 113 controls from the TCGA database (https://portal.gdc.cancer.gov/) as our training dataset. We introduced the GSE163882 dataset, which is based on the GPL570 Affymetrix Human Genome U133 Plus 2.0 Array platform. It comprises gene expression profiles of 327 fresh frozen breast cancer tissues from one-third of the patients diagnosed and treated at the Sun Yat-sen Cancer Center of the Sun Yat-sen Foundation between 1991 and 2004. The GSE163882 dataset served as the training set for prognostic gene screening and risk model construction. The GSE20685 dataset served as an independent test set to validate differences in survival and other aspects between high- and low-risk groups. Additionally, scRNA-seq data from six BC samples, totaling 1,534 cells, were obtained from the GSE118389 dataset. This dataset is based on the GPL9052 Illumina Genome Analyzer (Homo sapiens) platform.

### Differential expression analysis and GO enrichment analysis

The study examined the differences in gene expression between two groups of breast cancer samples: drug-resistant and drug-sensitive. The investigation employed the "limma" algorithm from the R package "limma." A total of 4,996 genes exhibited a p value of less than 0.05 between the two groups. Additionally, gene ontology (GO) enrichment analysis was performed using the R package "clusterProfiler." The results were visualized using the R package "ggplot2."

### Screening of prognostic genes and construction of prognostic models

Prognostic genes associated with exosomes were identified in this study at a significance level of *p* < 0.05 through univariate Cox regression analysis. Key genes that influence the prognosis of patients among the previously identified genes were further determined using Lasso regression and tenfold cross-validation. Subsequently, prognostic models for BC were developed based on these genes. Patients were assigned a risk score according to the constructed model, and BC samples were categorized into high- and low-risk groups using the median risk score value. To validate the model, ROC curve analysis and Kaplan–Meier (KM) survival curve analysis were performed on both training and test sets. KM curves were generated for prognostic genes to assess their predictive capability for BC cell metastasis, utilizing distant metastasis-free survival (DMFS). The Kaplan–Meier database, accessible at http://kmplot.com/analysis/index.php?p=service&cancer=breast, was employed for the analysis.

### Construction of nomogram model

Based on the predictions generated by the prognostic model, we constructed a nomogram model. This confirms the independent prognostic factor status of the risk score for breast cancer (BC). Our team customized a clinical nomogram model for BC using the R packages “rms” and “regplot.” The model considers the risk score, clinical stage, and baseline patient information from the training dataset. To assess the predictive capability of the nomogram model in breast cancer patients, we developed calibration and concordance index (C-index) curves and conducted decision curve analysis (DCA).

### Immune correlation analysis

To compare the levels of immune cell infiltration between high- and low-risk groups, we employed the ssGSEA algorithm from the R package “GSEA.” This algorithm provides abundance information for various subtypes of immune cells in BC samples. Additionally, we utilized the “ESTIMATE” algorithm, based on the R package of the same name, to compute matrix, immune, and stromal scores for samples in both high- and low-risk groups. We collected multiple immune checkpoint and HLA-related genes to assess their differential expression between the two groups. The Immune Surface Scores (IPS), obtained impartially, exhibit a positive correlation with responses to immunotherapy. IPS data for BC patients were obtained from the Cancer Immunome Atlas (TCIA, https://tcia.at/home).

### Drug sensitivity analysis

Using the R package “pRRophetic,” we have predicted the IC50 values of 138 compounds obtained from diverse BC tissues. Technical terms were explained upon their initial usage, and a formal tone was consistently maintained throughout the text, adhering to common academic structure and formatting conventions. We have identified compounds with significantly distinct IC50 values between two groups. The IC50 value serves as an indicator of a compound’s potential to inhibit a specific biological or biochemical function. Comprehensive information about the 138 compounds was sourced from the Cancer Genome Project database (https://www.sanger.ac.uk/group/cancer-genome-project/). Criteria for retention were established based on compounds exhibiting substantial differences between the high- and low-risk groups (*p* < 1e–10).

### BC typing based on NMF/consensus clustering

This study categorized breast cancer (BC) according to the expression of prognostic genes. Specifically, the study employed the R package “NMF” to execute the nonnegative matrix clustering algorithm 50 times using the standard “brunet” approach. We varied the number of clusters (k) from two to ten, and the average contour width of the common member matrix was calculated using the R package “NMF.” The optimal number of clusters was determined based on factors such as phenotype, dispersion, and silhouette.

### Correlation analysis of scRNA data

The analysis of single-cell RNA data was conducted using the R package “Seurat.” The initial step involved performing data quality control, during which genes associated with mitochondria and erythrocytes were excluded. Subsequently, the following screening parameters were applied: the “nFeature_RNA” criterion selected values above 200 and below 100,000; the “percent.mt” criterion selected values below 20, and the “nCount_RNA” criterion selected values below 100,000. In the normalization process, we identified the top 2,000 genes with high variability across all cells using the “LogNormalize” and “vst” methods. Here, “nFeature_RNA” refers to the number of detected genes in each single-cell sample. “percent.mt” represents the percentage of mitochondrial gene expression in the total gene expression. “nCount_RNA” indicates the total number of RNA molecules detected in each single-cell sample. “LogNormalize” denotes the normalization of gene expression for each cell to make the data distribution more akin to a normal distribution. “vst” refers to the variance-stabilizing transformation applied to minimize technical noise in the data.

We subsequently conducted principal component analysis (PCA) analysis, scaled the data, and retained the first 15 principal components for clustering purposes. The cells were then subjected to cluster analysis with a resolution of 0.5. The cell clusters were labeled using the R package "singleR." We then used the t-SNE algorithm to generate a two-dimensional visualization of the cell clusters and their respective types. Additionally, we employed the R package "AUCell" to calculate the area under the curve (AUC) value for each cluster. Our study identified the population of cells with high scores and the pathway scores that had significant enrichment in this particular cell population based on prognostic genes. The gene set we used is "h.all.v2022.1.Hs.symbols.gmt."

### RT-qPCR method for the verification of prognostic genes

Total RNA was extracted from A10 breast epithelial cells and MDA-MB-231 breast cancer cells, both of which were obtained from the Cell Bank of the Chinese Academy of Sciences, using the AG RNA Extraction Kit II (AG21022). Reverse transcription was performed using the Evo M-MLV RT Kit, and gDNA Clean for qPCR (AG11711). RT-PCR was conducted with the SYBR Green Premix Pro Taq HS qPCR Kit (AG11701) in a real-time fluorescent quantitative PCR analyzer from Agilent. Primer information for the validated genes is provided in Table S1 in the Supplementary material.

### Statistical analysis

Statistical analyses were performed using R software (version 4.2.0) and SPSS software (version 26.0). For continuous and categorical variables, we utilized the Wilcoxon rank-sum and chi-square tests, respectively. All analyses were deemed statistically significant at a *p*-value of < 0.05. We denote *p*-values less than 0.05 and 0.01 with “*” and “**,” respectively.

## Result

### Identification of differential drug resistance and exosome-related genes in BC

**Fig. 1:**
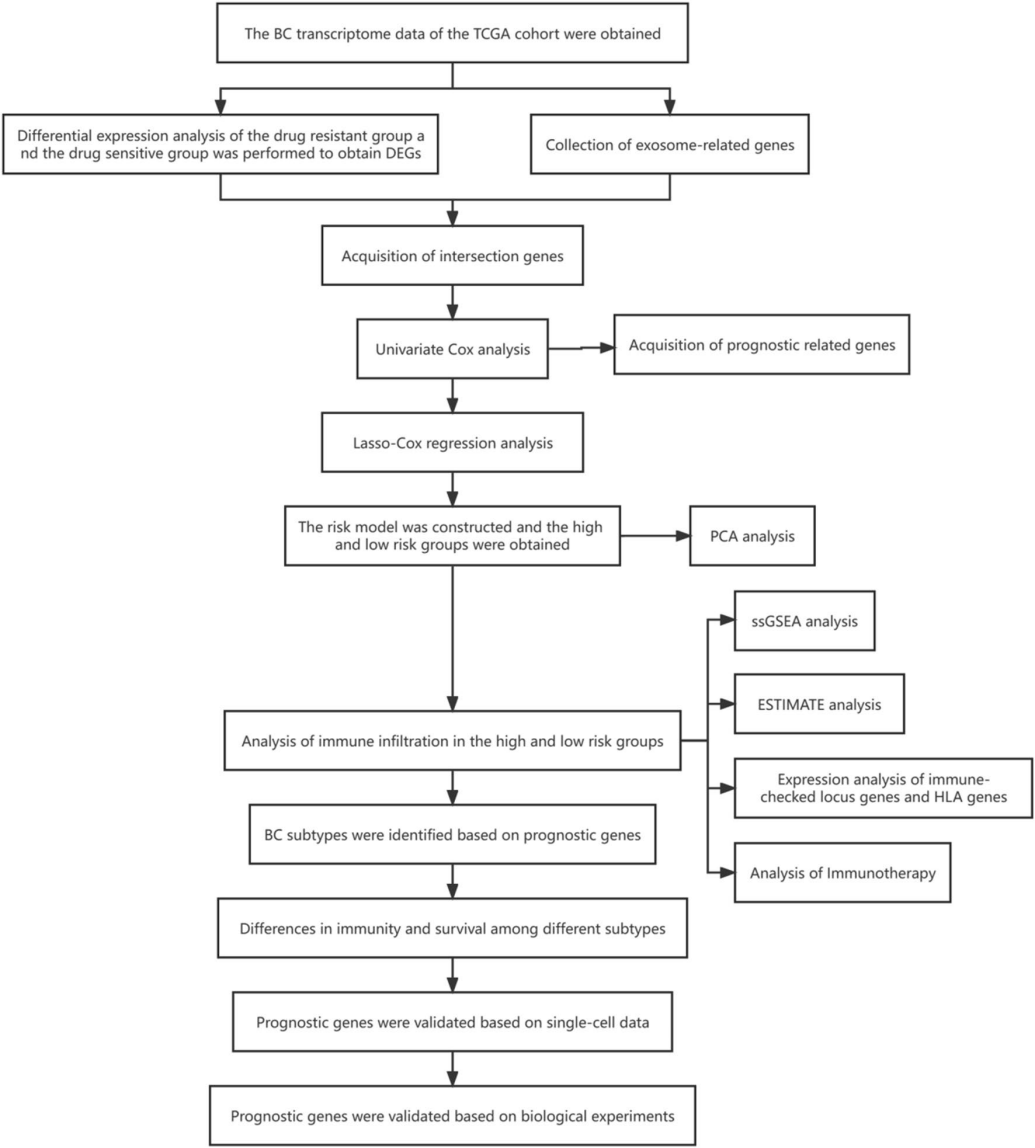
Flowchart

**Fig. 2:**
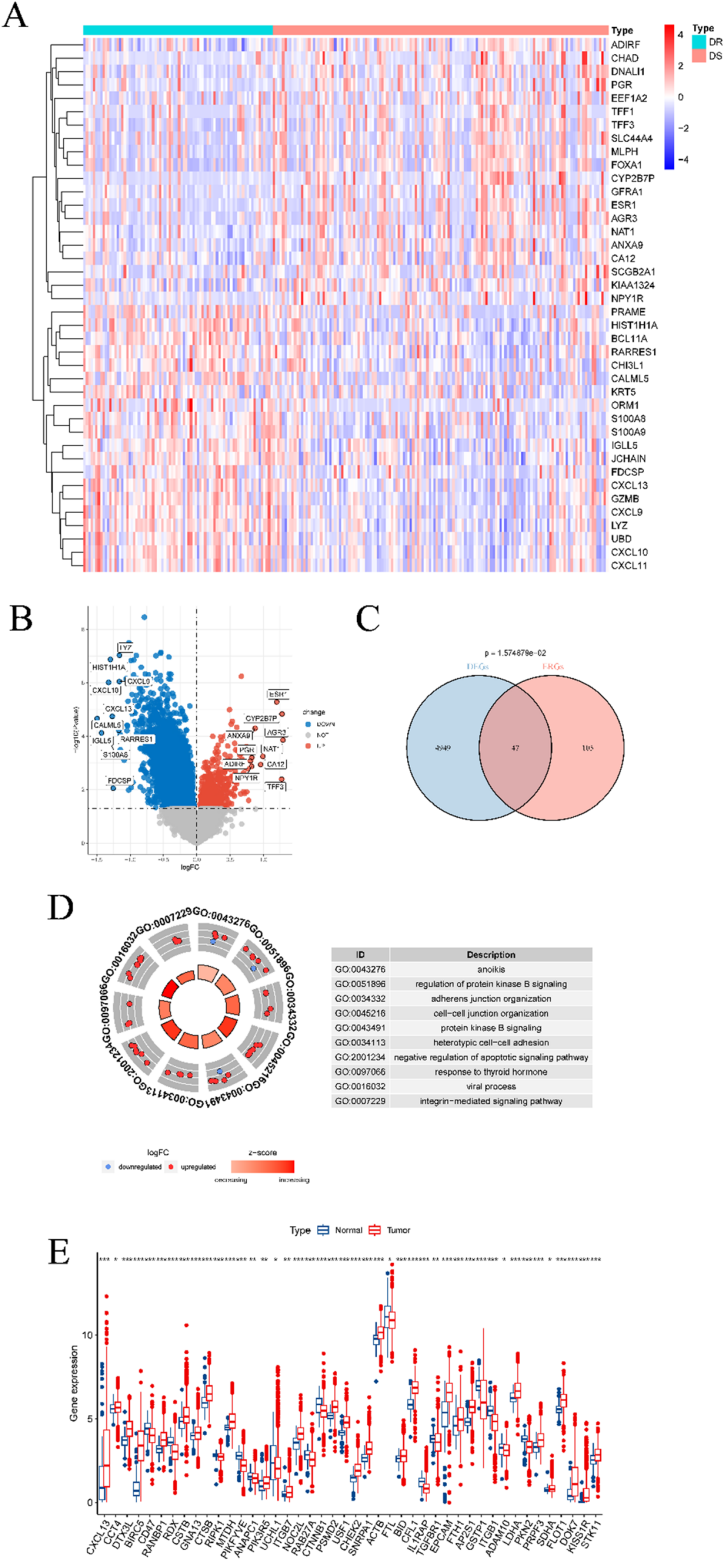
Results of differential analysis between drug-sensitive and resistant groups in GEO cohort. **A** and **B** are the heat map and volcano map obtained from the difference analysis, respectively. **C** is the Venn diagram of the intersection of DEGs and exosome-related genes. **D** is the GO enrichment result of EGDR. **E** is the expression boxplot of EGDR in the transcriptome data of TCGA cohort

The technical process outlined in this article is depicted in Fig. 1. A differential analysis of transcriptome data was conducted to identify genes associated with drug resistance by comparing the drug-sensitive and drug-resistant groups from the GSE163882 dataset. The results of the differential analysis, including the heatmap and volcano plot, are presented in Fig. 2A, B. Details of DEGs are provided in the “GSE163882_diff.xls” file in the Supplementary Material. Figure 2C illustrates the Venn diagram produced from the intersection of DEGs and exosome-associated genes, resulting in 47 shared genes. Gene enrichment analysis using GO on these shared genes revealed pathways linked to BC and BC-associated medication responses, as shown in Fig. 2D. The biological significance of these pathways will be discussed in the following section. Box plots were utilized to evaluate the expression of communication-related genes in breast cancer patients and their controls from the TCGA cohort. The results showcased significant variations in the expression levels of most of these genes between the two groups.

### Establishment and verification of prognostic model

**Fig. 3:**
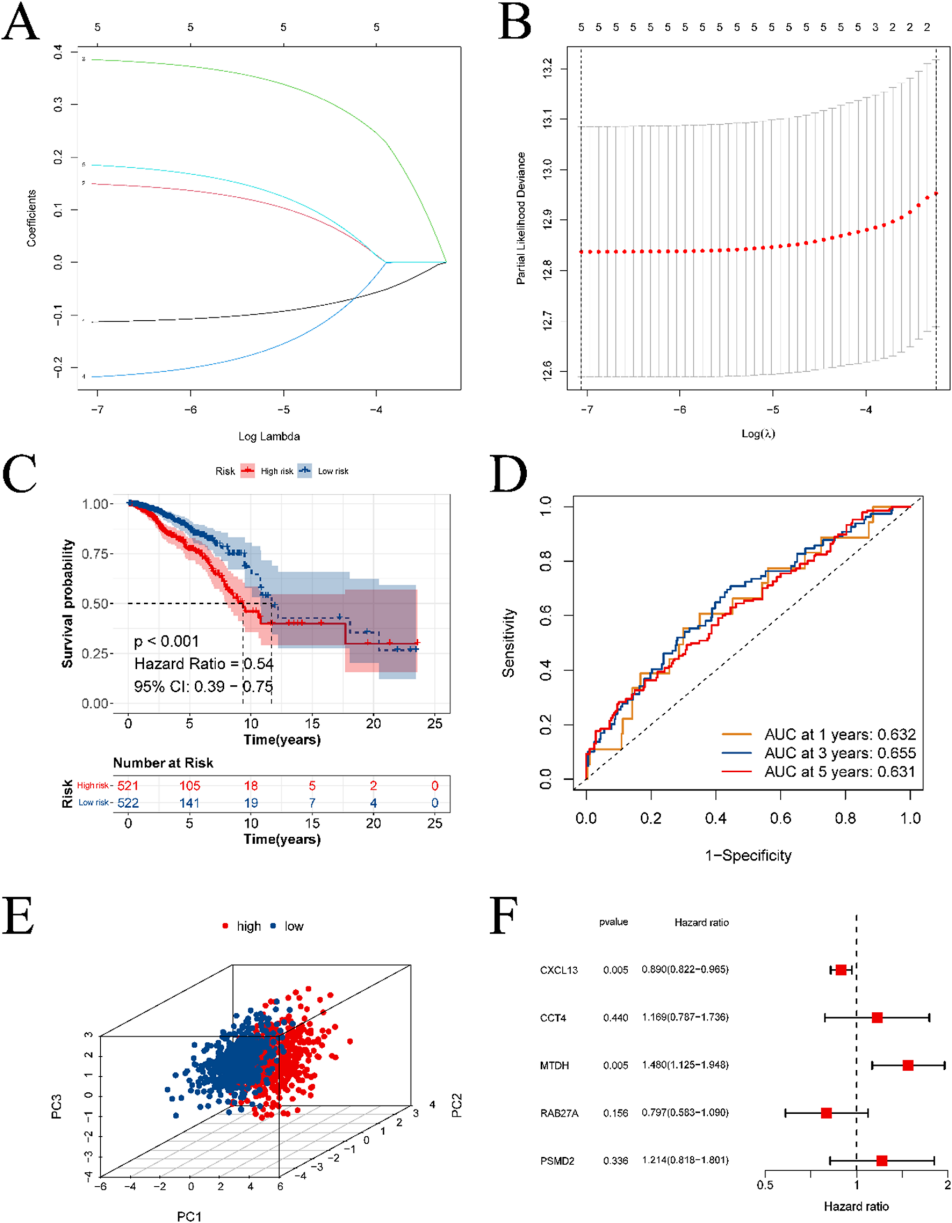
Results of prognostic model construction. **A** is the least absolute shrinkage and selection operator (LASSO) regression of prognostic genes. **B** is the cross-validation used to adjust the choice of parameters in the LASSO regression. **C** and **D** are the results of survival analysis and ROC analysis of the prognostic model, respectively. **E** is a scatter plot after dimensionality reduction using PCA for samples from the high- and low-risk groups. **F** is the forest plot obtained by multivariate Cox regression

In this study, we developed a prognostic model for BC using Lasso-Cox regression analysis. Five gene markers were identified through the Lasso algorithm, based on the optimal λ value (refer to Fig. 3A, B). Specifically, LASSO calculates a coefficient for each gene. The first graph is the coefficient distribution graph. Each line in the graph represents a gene, and the end of these genes will point to an ordinate (representing the coefficient of the gene) from which the gene can be further screened after taking λ as the threshold value. Figure 3B shows that the coefficients of five genes could be retained. Utilizing the calculated risk scores, the model categorized BC samples into high- and low-risk groups. We presented the results of survival analysis and ROC curve examination for these groups on the TCGA dataset in Fig. 3C, D. Importantly, a significant variance in survival time was observed between the two cohorts. The model for estimating patient prognosis indicated that the risk score yielded area under the curve (AUC) values of 0.632, 0.655, and 0.631 for predicting patient survival after 1, 3, and 5 years, respectively. PCA of the samples from the training group revealed a clear separation of low and high-risk groups on a two-dimensional plot (refer to Fig. 3F). The forest plot in Fig. 3F emphasizes the identification of five genes as prognostic markers through multivariate Cox regression analysis. In addition, we performed an independent prognostic analysis to assess whether the five genes constituting the prognostic model could be used as independent prognostic factors to predict breast cancer survival (Fig. 5). The results showed that only CXCL13 and MTDH could be used as independent prognostic factors.

**Fig. 4:**
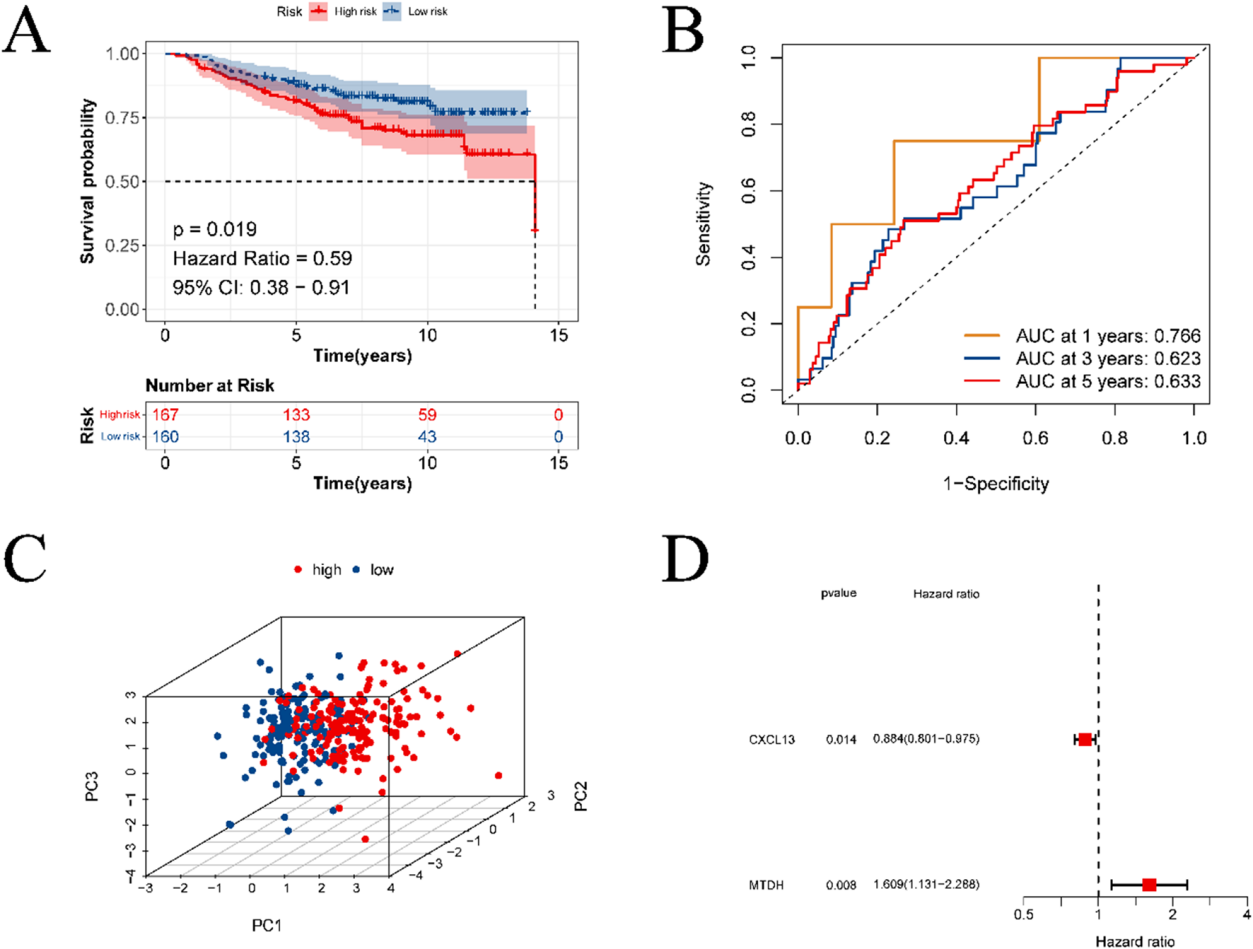
External validation of prognostic models. **A** is the KM survival curve of the prognostic model on the validation set. **B** is the result of ROC analysis on the validation set. **C** is the forest plot obtained from multivariate COX regression analysis to screen independent prognostic factors. **D** is the result of PCA analysis on the validation set

Subsequently, a test dataset was utilized to validate the model’s performance. Figures 4A, B provide details on the Kaplan–Meier survival and ROC curves for the high-risk and low-risk groups in the test set, indicating a significant difference in survival between the two groups. The AUC values for predicting survival at 1, 3, and 5 years were 0.766, 0.623, and 0.633, respectively. Figure 4C, D presents the outcomes of both PCA and multivariate Cox regression analyses. The results further confirmed that CXCL13 and MTDH can be used as independent prognostic factors of breast cancer to predict the survival of breast cancer patients.

**Fig. 5:**
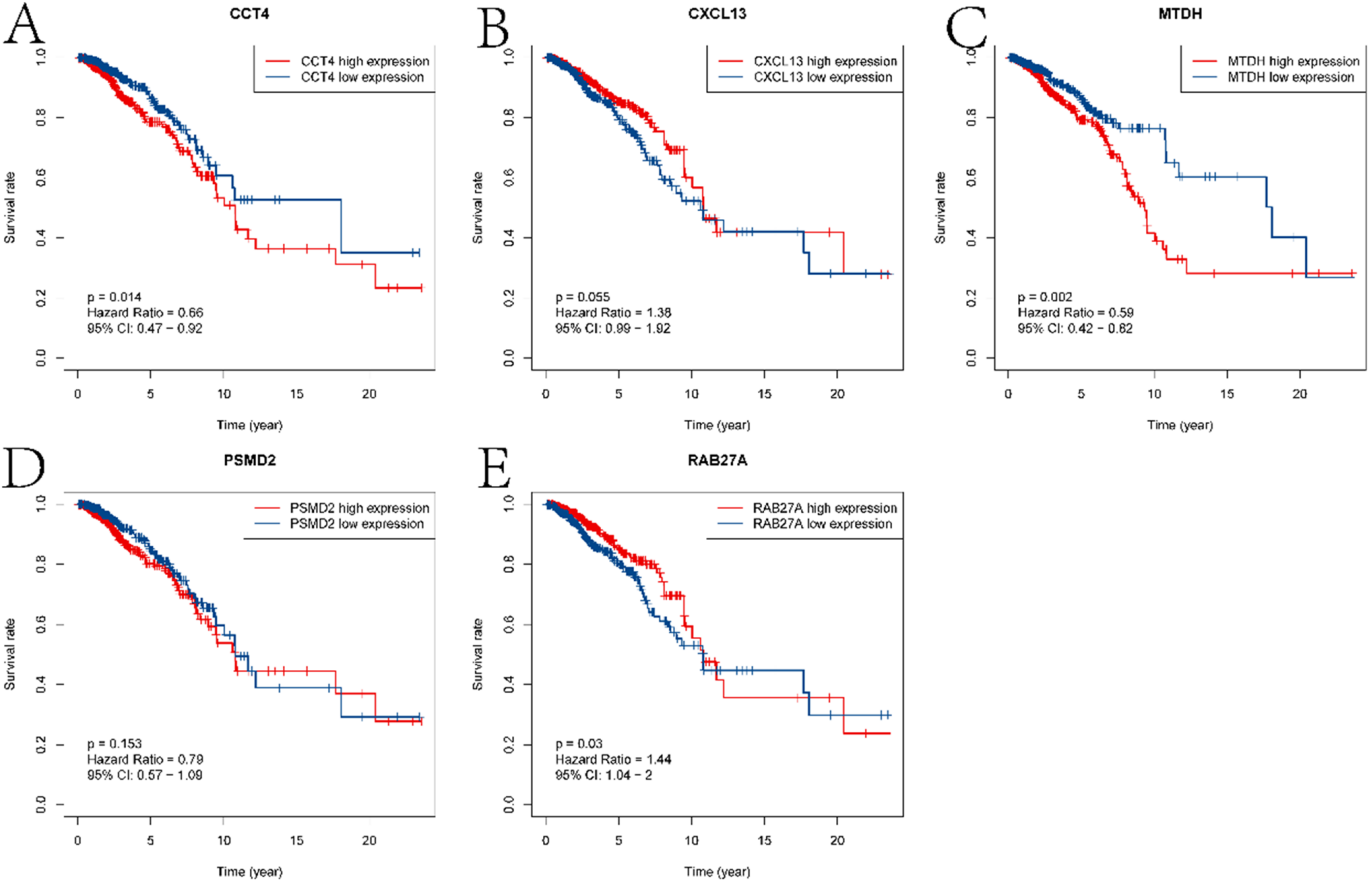
Survival validation of prognostic genes. **A**–**E** are the survival curves for CCT4, CXCL13, MTDH, PSMD2, and RAB27A, respectively

**Fig. 6:**
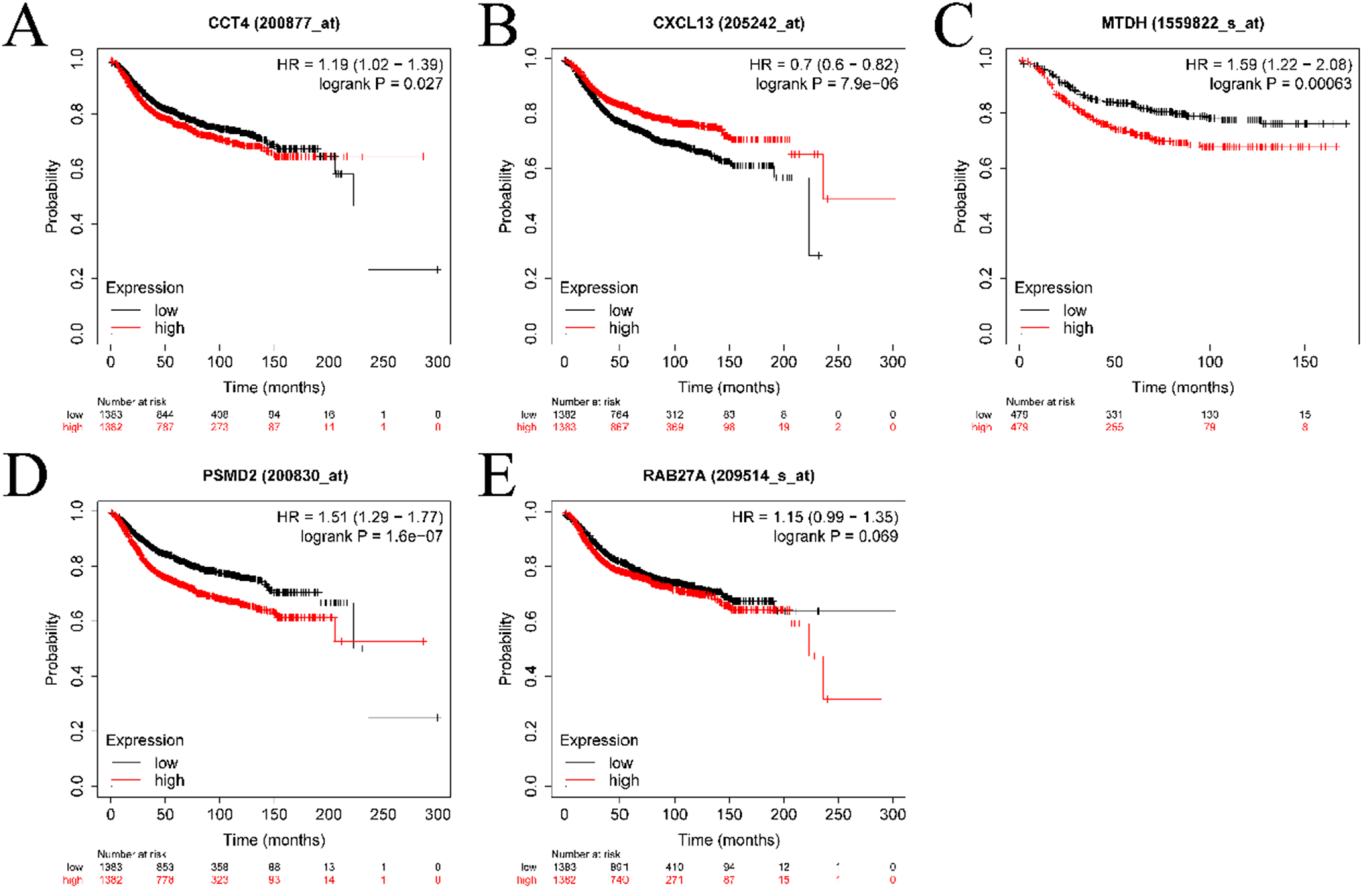
KM curves of prognostic genes for distant metastasis (DMFS). **A**–**E** are the survival curves for CCT4, CXCL13, MTDH, PSMD2, and RAB27A, respectively

Prognostic validation was conducted for the five prognostic genes identified in the training set (refer to Fig. 5). Notable disparities in survival were observed between the high and low expression groups of each of the five genes when breast cancer samples were stratified accordingly. Additionally, DMFS for the high and low expression groups, segregated by these prognostic genes, was validated. The KM survival curves displayed in Fig. 6 reveal significant DMFS survival differences for the two expression groups.

### Construction and validation of nomogram model

**Fig. 7:**
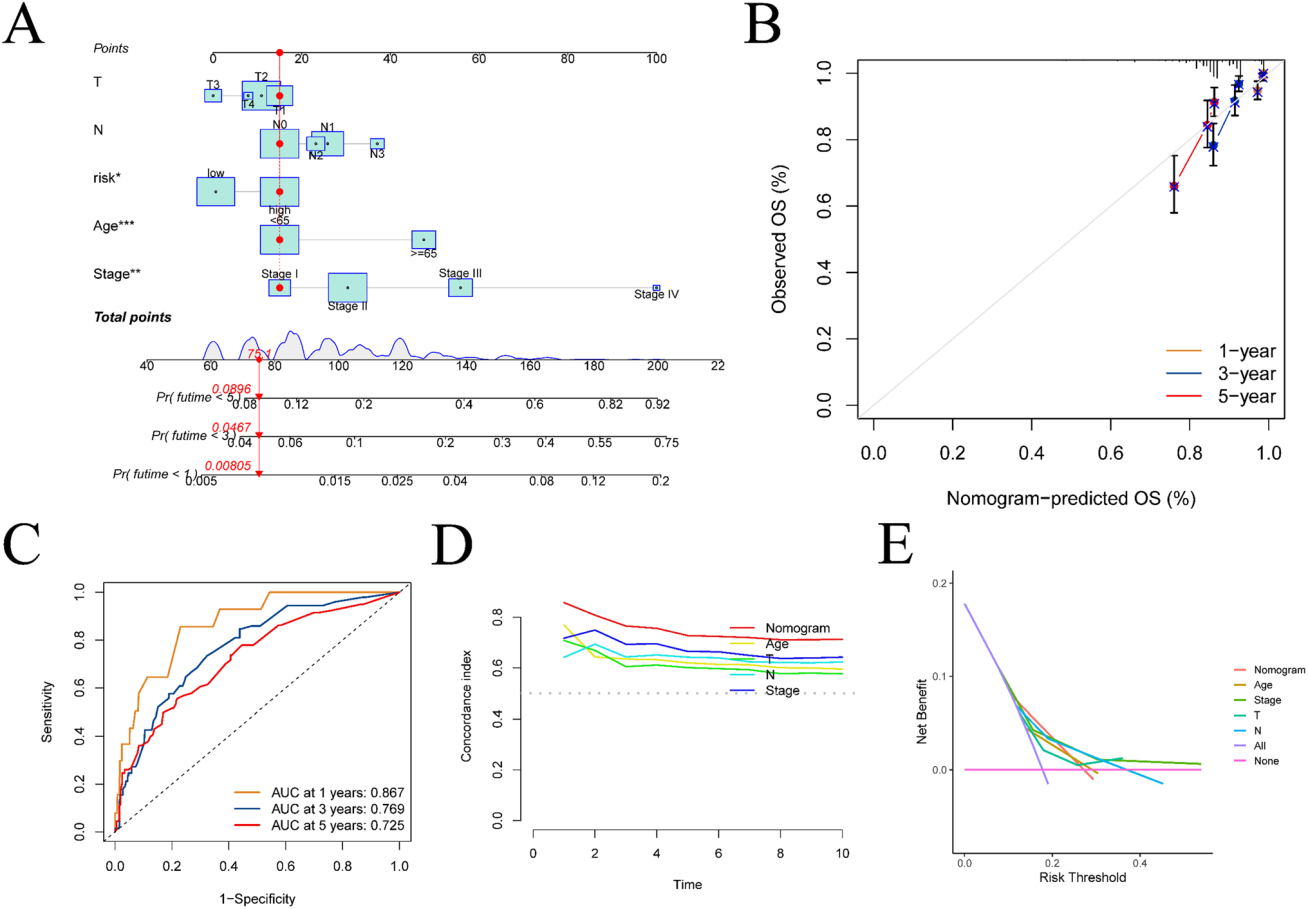
Construction and validation of the nomogram model. **A** is a nomogram model constructed based on clinical stage, baseline information of patients, and risk score. **B** is the calibration curve of the nomogram model. **C** is the result of ROC curve analysis of the nomogram model. **D** is the result of C-index analysis. **E** is the DCA result of the nomogram model

The present study outlines the development of a nomogram model (depicted in Fig. 7A) utilizing baseline information from patients (including age and clinical stage) and a risk score. This is done to assess the potential risk associated with BC. The calibration curve for this model, as shown in Fig. 7B, illustrates the extent of deviation from the ideal model. Additionally, the ROC analysis results for the nomogram model are presented in Fig. 7C, revealing AUC values of 0.867, 0.769, and 0.725 for predicting the 1-, 3-, and 5-year survival rates of patients, respectively. Notably, these values surpass those obtained using the prognostic model. Figure 7D displays curves exhibiting the C-index over time for various models, with the nomogram model achieving the highest C-index. According to the DCA depicted in Fig. 7E, the predictive accuracy of the nomogram model surpasses that of age and clinical stage.

### Results of correlation analysis of immune infiltration

**Fig. 8:**
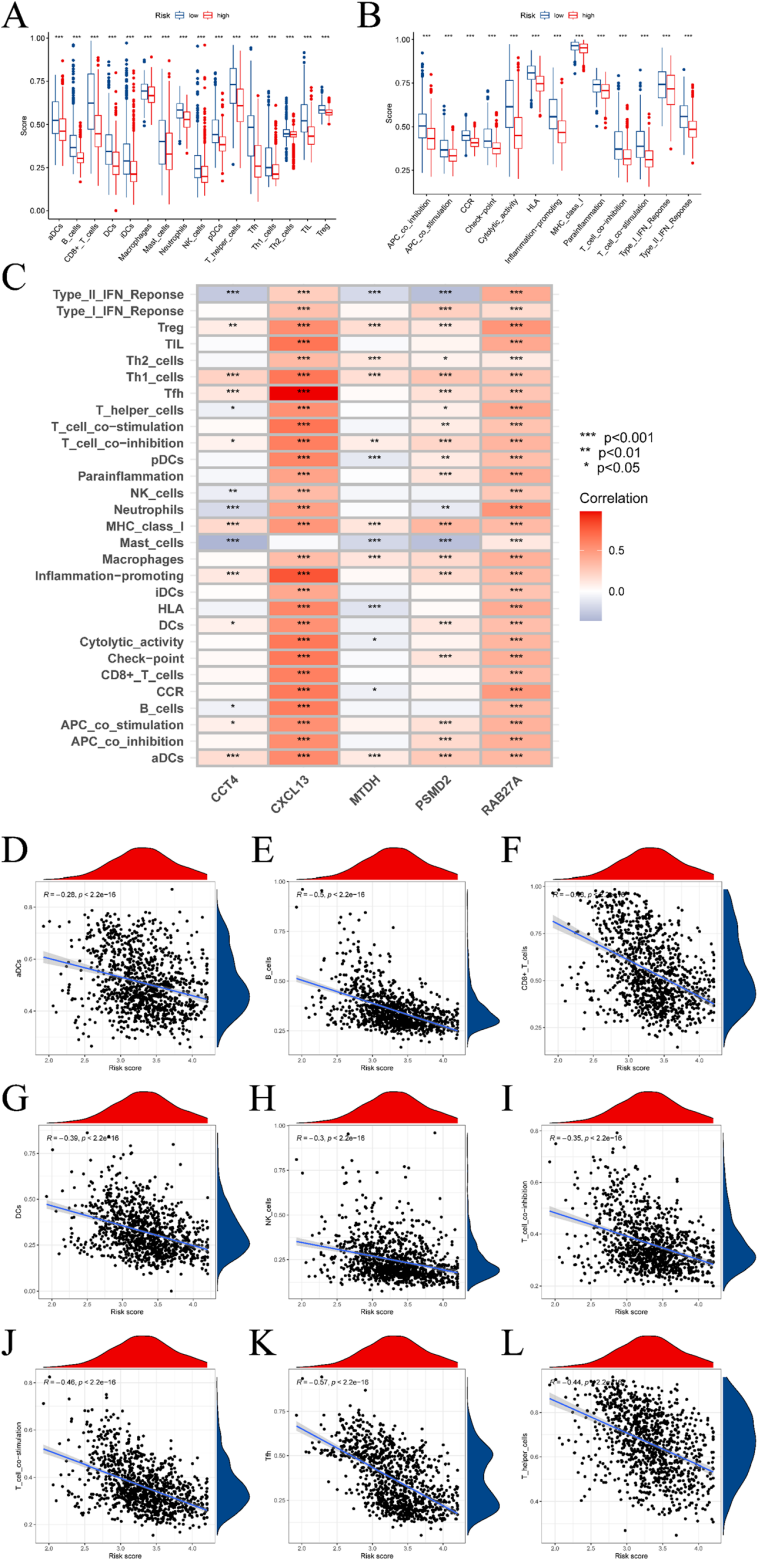
Evaluation of immune infiltration in BC samples based on ssGSEA analysis. **A** is the boxplot of the difference in the abundance of immune cell infiltration in the high- and low-risk groups. **B** is the boxplot of the difference in immune function between the high- and low-risk groups. **C**–**L** is the correlation analysis of prognostic genes and immune cells; correlation analysis of risk scores and immune cells

The ssGSEA algorithm was utilized to assess variances in the abundance of immune cell infiltration and immune function scores of multiple immune cells in high- and low-risk groups, determined by gene expression levels in BC specimens (refer to Fig. 8A). Significantly different infiltration abundance and immune function scores were observed in immunocytes of both groups. Figure 8C–L displays scatter plots delineating specific genes that exhibit substantial links with immune cells. Further outcomes can be found in the supplementary materials’ Immune section.

**Fig. 9:**
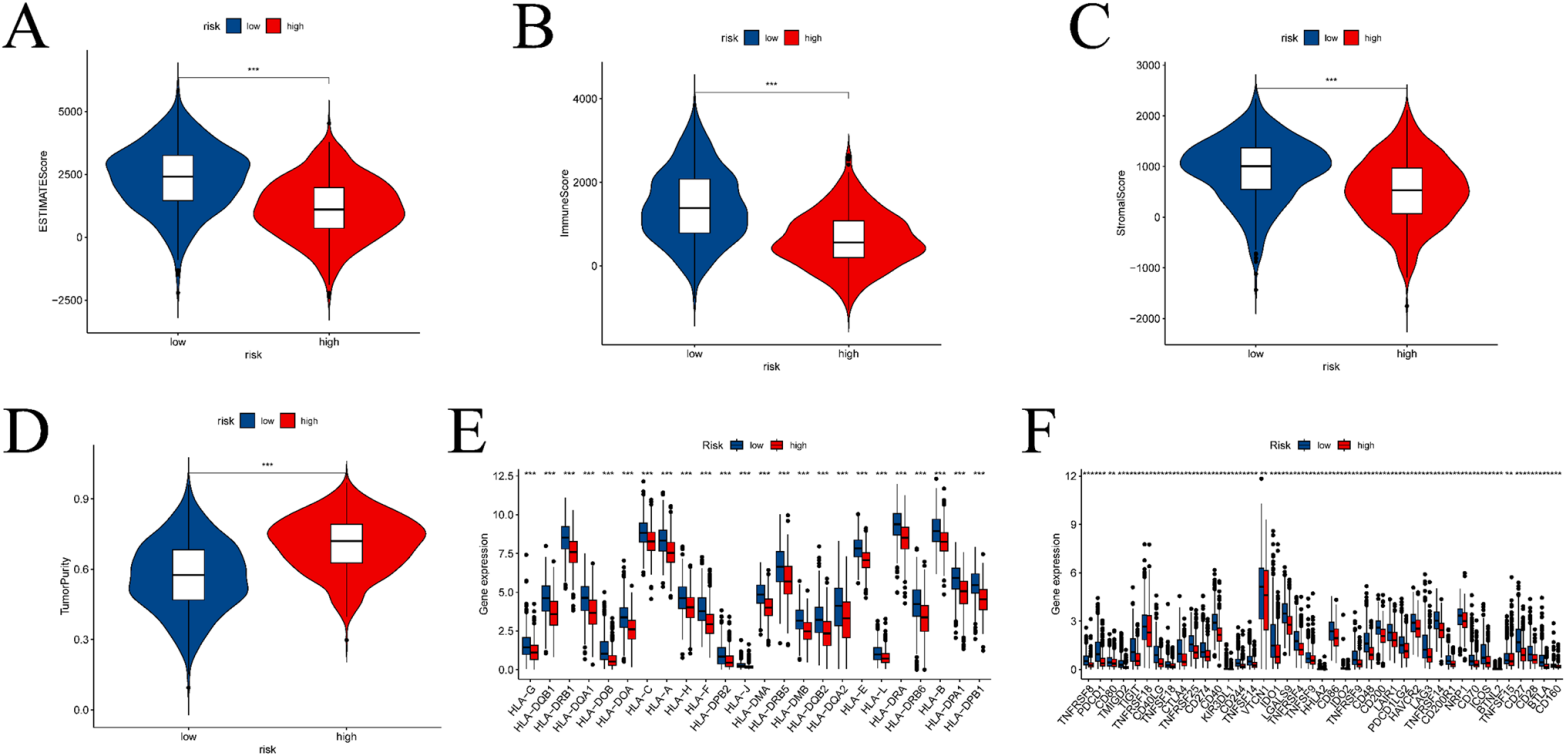
ESTIMATE analysis between high- and low-risk groups, expression analysis of HLA-related genes and immunoassay sites. **A**–**D** is the result of ESTIMATE score, immune score, Stromal score, and tumor purity in the high- and low-risk groups based on ESTIMATE analysis, respectively. **E** and **F** is a boxplot based on the expression of HLA-related genes and immunoassay sites in the high- and low-risk groups, respectively

**Fig. 10:**
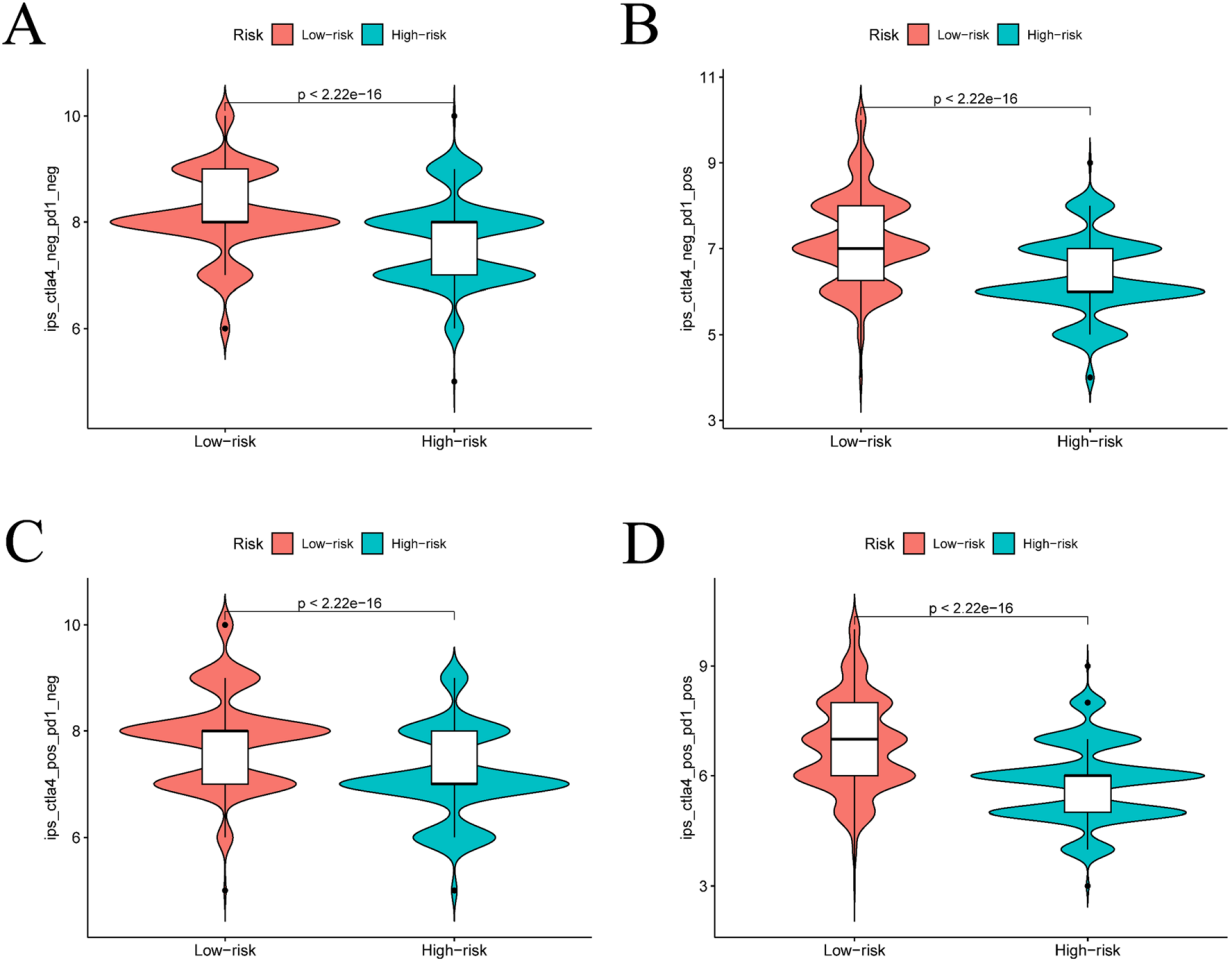
Results of immunotherapy analysis. The IPS (**A**), IPs-ctLA4 (**B**), and IPs-PD1/PD-L1/PD-L2+CTLA4 high- and low-risk groups (*p* < 0.05)

**Fig. 11:**
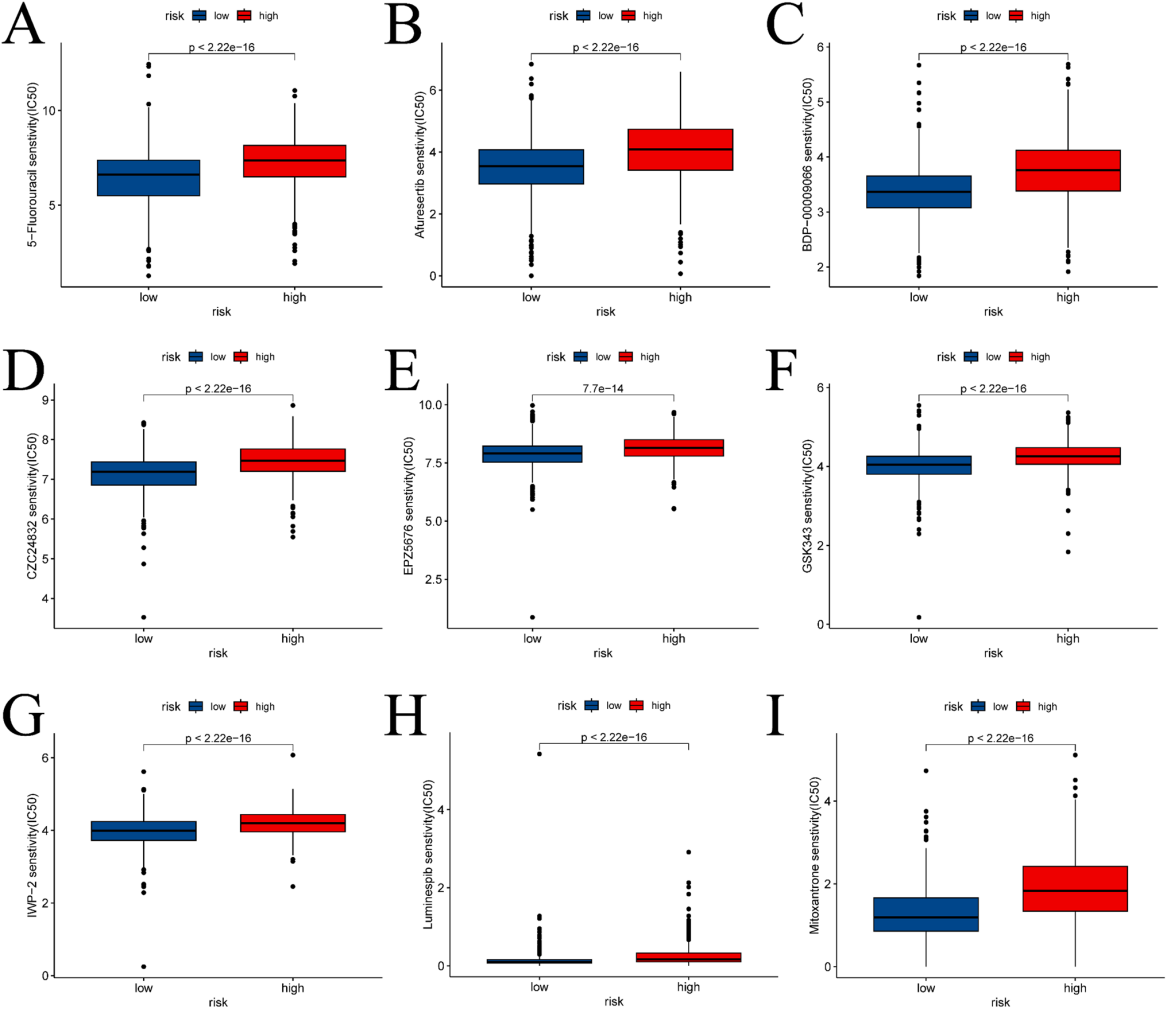
Results of drug sensitivity analysis of the high- and low-risk groups. The fractions of **A**–**I** had compounds with significant differences between the high- and low-risk groups

The dissimilarities in the immune landscape between the high-risk and low-risk groups were evaluated from multiple viewpoints in this study. Notably, the results show statistically significant differences between the two groups in ESTIMATE score, immune score, stromal score, and tumor purity utilizing the ESTIMATE analysis assessment outcomes (refer to Fig. 9A–D). Additionally, we conducted an investigation into variances in the expression of HLA-related genes and immunoassay sites among both groups (see Fig. 9E, F) and examined variations in immunotherapy (see Fig. 10). Figure 11 presents IC50 values of specific drugs that significantly differ between the two groups (further information can be found in the supplementary materials, in the Drug1 folder).

### BC typing based on prognostic genes

**Fig. 12:**
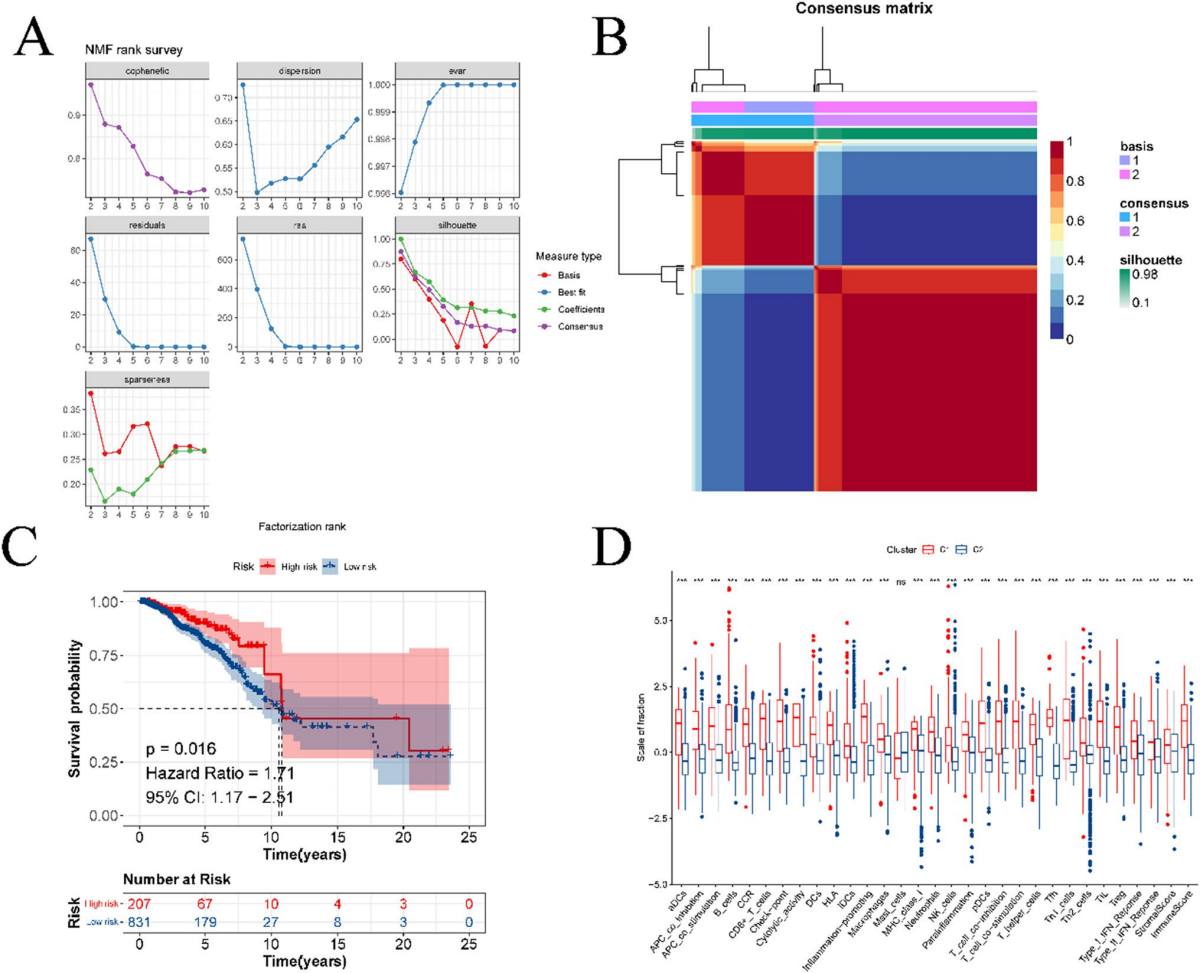
Identification of BC subtypes based on prognostic genes. **A** is phenotype distribution, rss distribution and dispersion distribution when rank = 2–10; **B** is the consensus map of NMF clustering. **C** is the prognostic survival curve of the two molecular subtypes. **D** is the difference in the abundance of immune cell infiltration among different subtypes

**Fig. 13:**
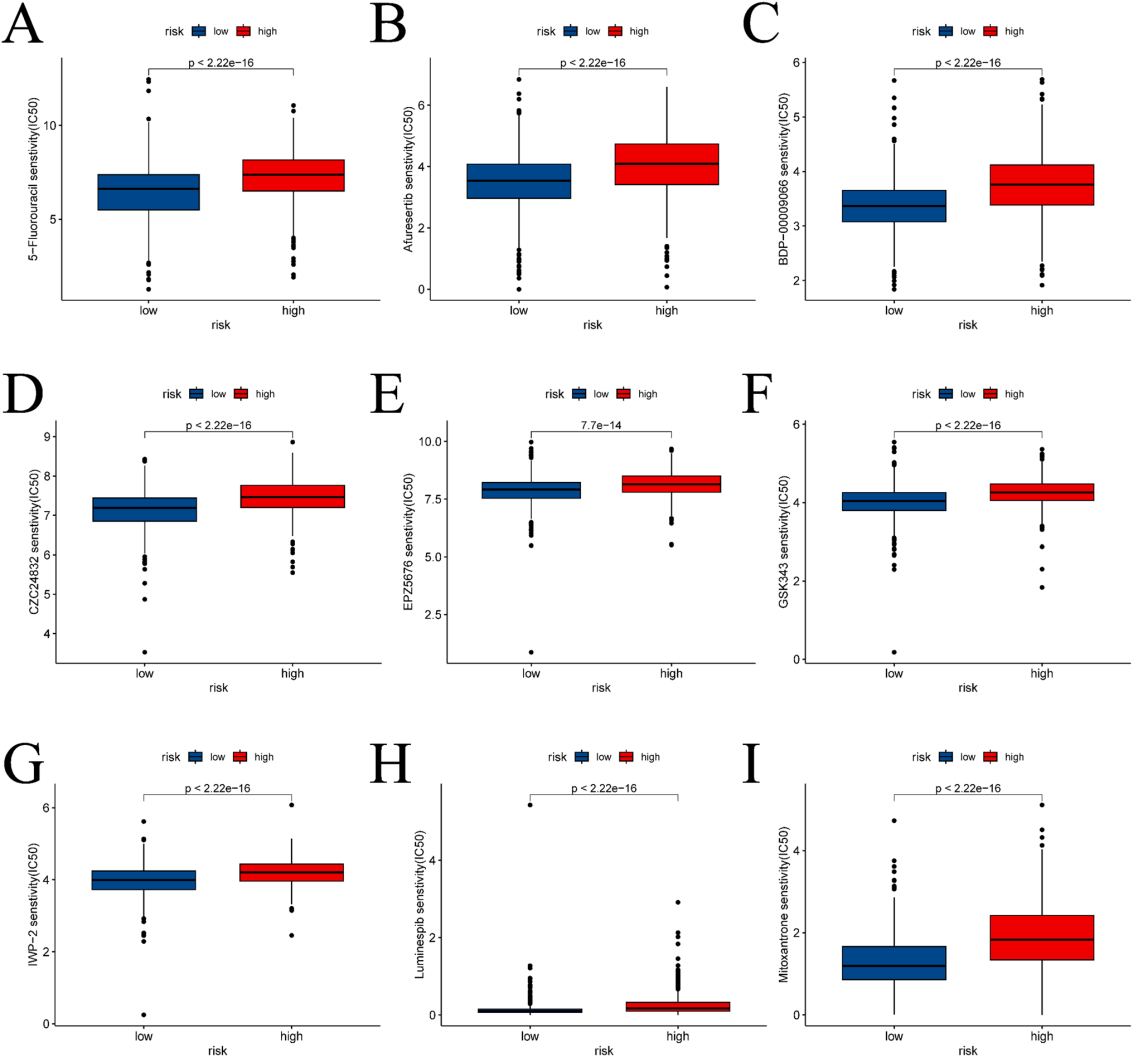
Drug sensitivity analysis of different BC subtypes

In this study, we utilized prognostic gene expression profiles to classify BC using the NMF clustering algorithm. As depicted in Fig. 12A, the NMF algorithm exhibits distinct phenotypic characteristics, RSS, and dispersion distribution across various cluster numbers. According to the most cophenetic curve in this figure, it can be judged that the leading point of the largest decrease is 2. Therefore, the NMF algorithm achieves the best performance when the number of clusters is 2. The figure highlights that the performance of the NMF algorithm peaks when the cluster number is set to 2, as illustrated in the consensus plot in Fig. 12B. Additionally, Fig. 12C and D depicts the survival curves for the two subtypes and the level of immune cell infiltration. Notably, significant differences in both survival and immune cell infiltration were observed between the two subtypes. Finally, we investigated the disparities in drug sensitivity between the two BC subtypes, as presented in Fig. 13 in the Drug2 folder in the supplementary material.

### Results of immune landscape and AUCell analysis at the single-cell level

**Fig. 14:**
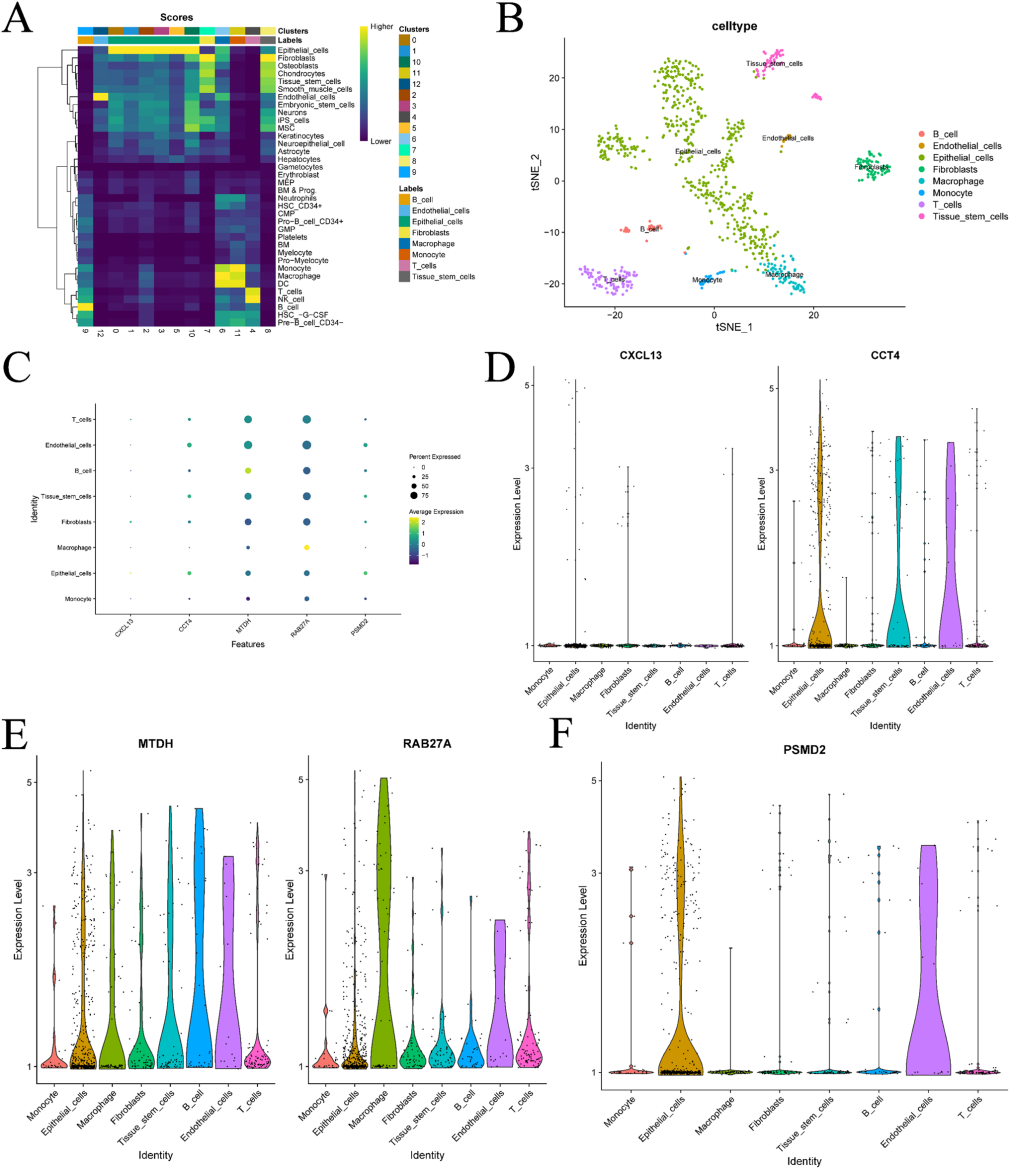
Results of cell clustering and annotation analysis of single-cell sequencing data. **A** is a heatmap that annotates the cell population based on the single-cell algorithm. **B** is the result of the visualization of the cell population based on tnse dimensionality reduction. **C** is a bubble plot of the expression of prognostic genes on different cell populations. **D**–**F** is a violin plot of the expression of prognostic genes on different cell populations

**Fig. 15:**
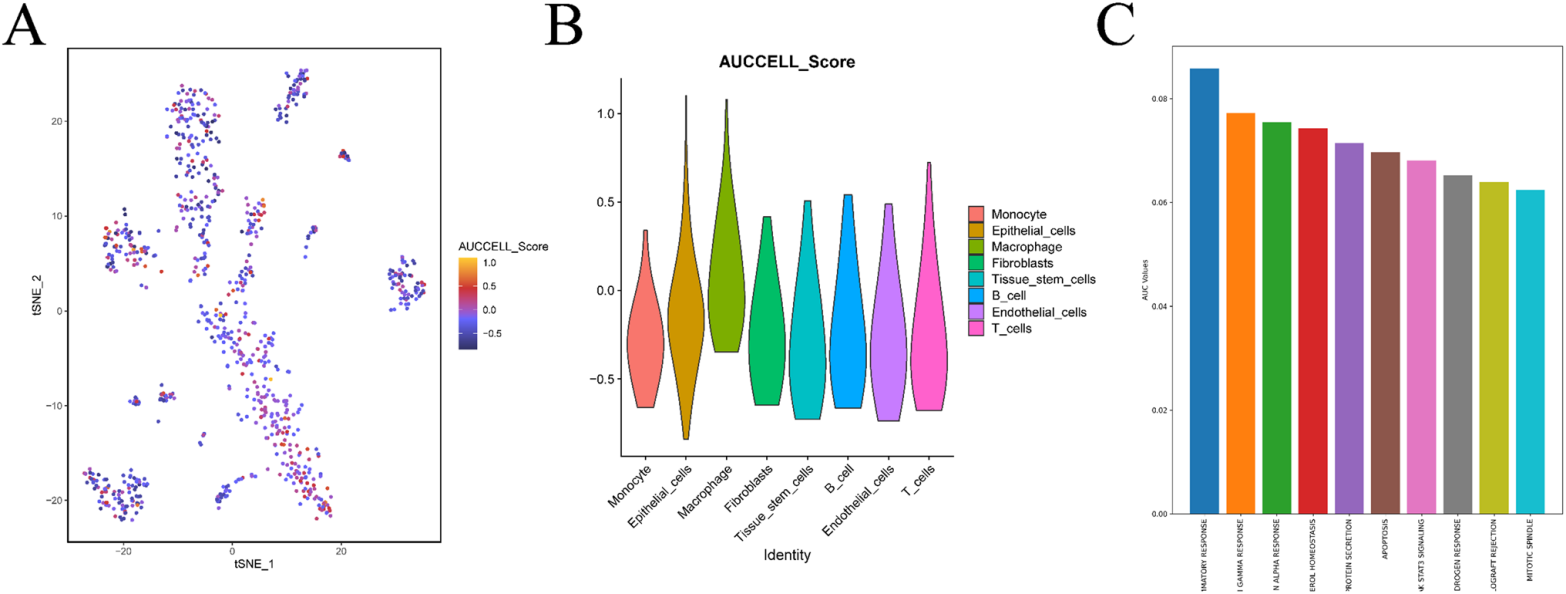
Results of the analysis based on AUCell. **A** is a scatter plot obtained by scoring different cell types by AUCell based on tsne. **B** is a violin plot based on the distribution of AUCell scores for different cell populations. **C** is the bar graph drawn for the top cell types and their corresponding AUC scores in the macrophage population

To generate multiple cell clusters, we conducted quality control, normalization, dimensionality reduction, and clustering using the R package Seurat. The cell clusters were then annotated using the R package singleR. Figure 14A depicts the scores of different cell clusters associated with various cell types. Figure 14B presents the final cell type annotations based on these scores. In Fig. 14C–F, the bubble and violin plots illustrate the expression of prognostic genes in different cell clusters. Notably, we observed high expression of MTDH and RAB27A in various immune cell types. Furthermore, we utilized the AUCell algorithm (Fig. 15A, B) to evaluate the scores for different cell types, with macrophages exhibiting the highest score. Figure 15C displays the different cell types within the macrophage population with elevated AUCell scores. The Discussion section provides a comprehensive explanation of the relationship between these pathways and breast cancer, along with potential drug targets.

Additionally, based on the gene expression matrix and cell type information from scRNA-seq data, we used AUCell to calculate the expression levels of genes in specified gene sets within each cell. Scatter plots of the top 10 pathways before scoring are provided in Supplementary Material Fig. S1. We will analyze the relationship between these pathways and the progression of breast cancer in detail in the Discussion section.

### The results of RT-qPCR were verified for the prognostic genes

**Fig. 16:**
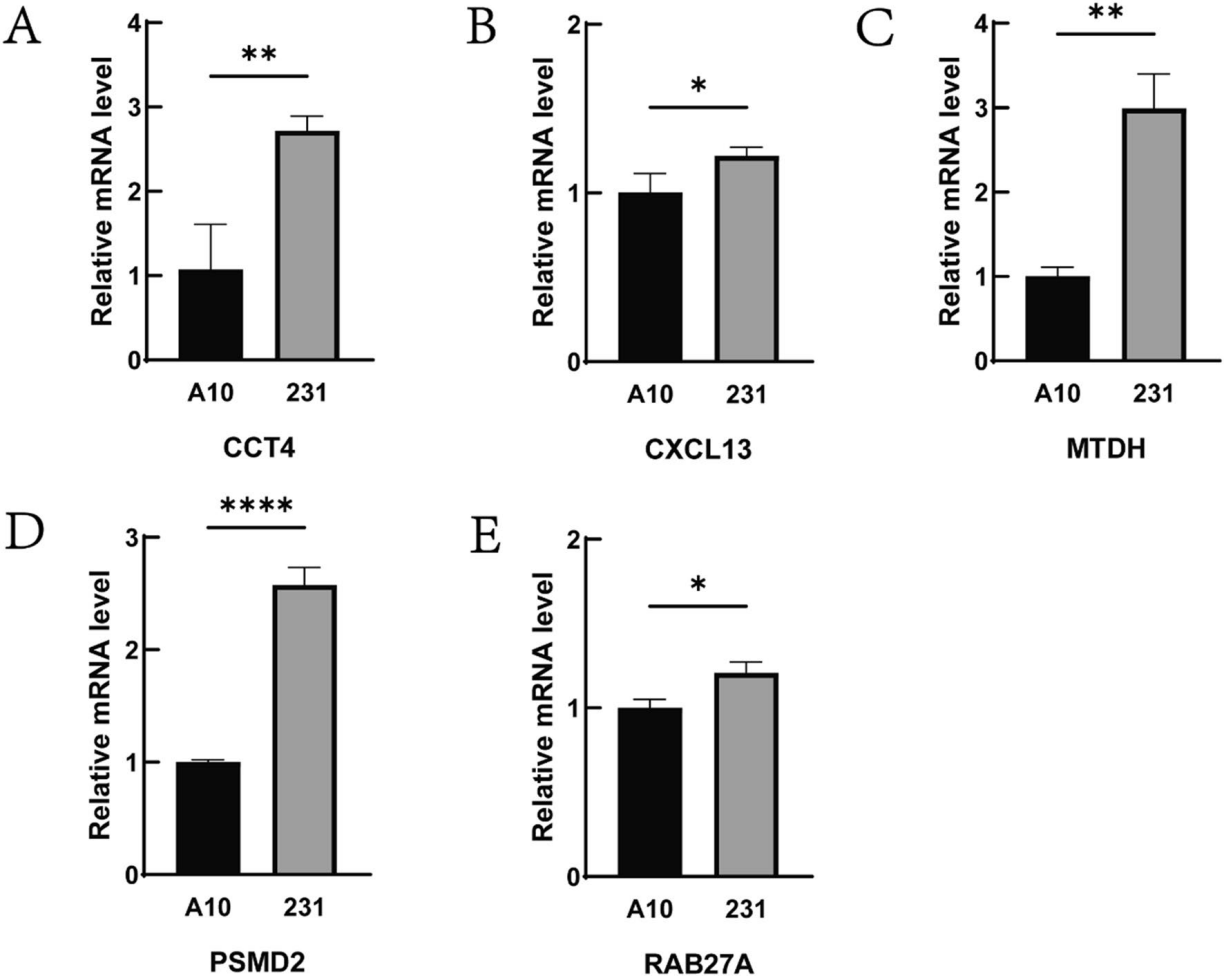
Verification results of gene expression by RT-qPCR. **A**–**E** is the expression histogram of CCT4, CXCL13, MTDH, PSMD2 and RAB27A in normal breast epithelial cells and breast cancer cells, respectively

Differences in the expression of prognostic genes between normal mammary epithelial cells and breast cancer cells were subsequently verified. Each sample was tested in triplicate, and a melting curve analysis was conducted to assess the specificity of amplification. Figure 16A–E presents a box plot illustrating the disparities in gene expression between the two cell groups. The verification process confirmed distinctions in the expression of the majority of genes between the two cell groups.

## Discussion

The heterogeneity observed in BC presents a significant challenge to the efficacy of isochemoth erapy across different stages and subtypes. Our study addresses this challenge by investigating personalized treatment strategies, with a particular focus on the role of exosomes in inhibiting tumor growth and regulating the immune response. We performed an overlap analysis of DEGs and ERGs between resistant and sensitive groups, followed by GO enrichment analysis of the common genes. This approach highlights the potential for tailored therapies based on genetic profiling, as shown in our flowchart. We also elucidated the role of exosomes in BC treatment by examining their interactions with key genes and pathways. These findings from our comprehensive analysis underscore the importance of the exosome pathway in the development of targeted BC therapies. A study by Du et al. demonstrated that everolimus has the potential to decrease the expression of protein kinase B (AKT) in BC cells, indicating its promise as a therapeutic agent [15]. According to research by Zheng et al., adriamycin-induced apoptotic MCF-7 cells show potential for BC treatment [16]. A study by Irene Lopez-Mateo suggested that thyroid hormone receptor β may act as a tumor suppressor in BC [17]. Ji-Young Park and colleagues induced the expression of manganese superoxide dismutase in BC cells through the protein kinase B and extracellular signal-regulated kinase signaling pathways [18]. Philip Bischoff and colleagues found that the progression of BC depends on the disruption of adherent tissue integrity [19]. In this study, Siyoung Choi and colleagues discovered that mineralization of the matrix inhibited integrin-mediated mechanical signal transduction by using a collagen matrix with adjustable mineralization, thereby inducing a less proliferative stem cell-like phenotype in BC cells [20].

We have also explored the prognostic potential of genes such as CCT4, CXCL13, MTDH, PSMD2, and RAB27A. These genes exhibit significant associations with BC prognosis in both TCGA and GEO cohorts. Our flowchart illustrates how these genetic markers can be employed in a clinical context to predict treatment outcomes and identify high-risk patients. This comprehension is crucial for advancing novel therapeutic targets and personalized treatment regimens. They have been validated as significantly associated with BC prognosis in the TCGA and GEO cohorts. Further exploration into the roles of these genes in BC initiation and progression may lead to the discovery of new treatment targets. According to Wang et al., the inhibition of CCT4 by anti-carlin-β treatment disrupts protein balance and specifically inhibits tumor cell growth [21]. The significance of CXCL13 in an effective T cell response to anti-PD-L1 therapy was demonstrated through the analysis of scRNA-seq and scRNA-ATAC data in triple-negative BC [22]. Shen et al. observed frequent overexpression of MTDH in BC patients with a poor prognosis. MTDH promotes metastasis and treatment resistance by interacting with staphylococcal nuclease domain-containing 1 (SND1). This complex plays a critical role in suppressing anti-tumor T cell responses in BC [23]. RAB27A has been identified as a mediator of human milk BC stem cells, promoting the growth of mammospheres. Additionally, RAB27A has been found to be synergistically inhibited [24].

The risk model utilized in our study is based on LASSO-Cox analysis, effectively categorizing breast cancer (BC) patients into high-risk and low-risk groups. This model has been seamlessly integrated into our flowchart and functions as a crucial tool for evaluating patient outcomes and customizing treatment strategies. We further investigated the variations in survival and immune landscapes between these risk groups, employing the ssGSEA algorithm to underscore noteworthy variations in immune cell infiltration and function. As depicted in Fig. 8C, five prognostic genes exhibit a statistically significant correlation with distinct immune cells. Consistent with the findings of Li et al.’s study, clinical evidence indicates an inhibitory effect on BC metastasis. Moreover, certain sources suggest that the TAM/CXCL1/NF-κB/FOXP3 signaling pathway could potentially serve as a therapeutic target to modulate Tregs and enhance immunotherapy for BC [25]. Janakiram and colleagues have comprehensively reviewed the interplay between T cell inhibition and immunotherapy in BC [26].

The immune-related aspects of breast cancer, particularly the role of HLA-associated genes, constitute the focal point of our discussion. Studies have illuminated the intricate relationship between these genes and the progression of breast cancer, along with their influence on therapy response. Our flowchart, incorporating these findings, underscores the significance of immunological assessments in formulating comprehensive treatment strategies. A substantial number of HLA-related genes and immunoassay loci exhibit noteworthy differences between high- and low-risk groups. The majority of these genes have been linked to breast cancer and its treatment. In their review, Zheng et al. expounded on the role of HLA-G in the immune microenvironment of breast cancer, emphasizing its utility in identifying new biomarkers for breast cancer [27]. Woll et al. reported that HLA-A2 dimers can accurately measure and track antigen-specific T cell immune responses in peptide vaccine clinical trials [28]. It was observed that a reduction in EZH435 expression in the human breast cancer cell line MDA-MB-231 significantly increased HLA-DRA mRNA expression, even without IFN-γ stimulation [29]. In a study involving 89 patients with metastatic breast cancer and 50 age- and sex-matched healthy volunteers, Song et al. investigated the percentage of peripheral blood T lymphocyte subsets and plasma cytokine levels. The study revealed that an elevated level of CD8(+) CD28(−) suppressor T lymphocytes may independently predict progression-free survival during follow-up after chemotherapy [30]. Abdullah et al. identified that pharmacological combinations of NRP1 with FGFR-targeted kinase inhibitors could be an effective treatment for patients with drug-resistant metastatic breast cancer [31].

We have identified BC subtypes by employing prognostic genes and subsequently explored variations in prognosis, immune response, and drug sensitivity among these subtypes. Notably, therapeutic agents such as oxaliplatin [32], paclitaxel [33], and gefitinib [34] have shown effectiveness in treating breast cancer.

Finally, we employed single-cell RNA sequencing (scRNA-seq) data to offer a more intricate insight into gene expression across various cell types. This enabled the identification of cell populations at risk. Seamlessly integrated into our flowchart, this approach underscores the significance of comprehending gene expression patterns within the context of breast cancer (BC) treatment and prognosis. The utilization of the AUCell algorithm facilitated the identification of high-scoring cell populations and pathways, many of which are pertinent to BC and its treatment, thus affirming the practicality of our model in clinical settings. X-box binding protein 1 (XBP1) plays a crucial role in the unfolded protein response (UPR). Vahid Arabkari et al. discovered the involvement of XBP1 in UPR, correlating it with endocrine resistance in BC [35]. In BRCA2 mutation carriers, Rachel Joyce et al. identified mTORC1 as a potential target for breast cancer prevention [36]. Results from Theresa E Hickey et al.’s experiments suggest that androgen receptor (AR) exerts a tumor-suppressive role in estrogen receptor (ER)-α-positive BC [37]. Kurt W Evans et al. found that oxidative phosphorylation represents a metabolic vulnerability in triple-negative BC, potentially exploitable in combination therapy [38]. Yvette Drabsch et al. provided a comprehensive review of the crucial role of TGF-β in the invasion and metastasis of BC [39].

In conclusion, our study offers a comprehensive overview of the intricate interplay among genetic, immune, and therapeutic elements in breast cancer. This integrated approach enhances our understanding of breast cancer and paves the way for more effective and personalized treatments.

## Conclusion

This study has developed and validated a prognostic model for the accurate stratification of BC samples using bioinformatics algorithms and biological experiments. The model predicts prognosis, immunity, and drug sensitivity in drug-resistant BC. It can serve as an independent prognostic factor, enhancing the comprehension of BC treatment. Furthermore, the prognostic genes identified can be utilized as a reference for the precise treatment of BC.

## Supplementary Information

Below is the link to the electronic supplementary material.

Supplementary file1 (DOCX 387 KB)

Supplementary file2 (ZIP 3179 KB)

## Data Availability

The data of the paper were downloaded from the TCGA database (https://portal.gdc.cancer.gov/) and the GEO database (https://ncbi.nlm.nih.gov/geo/).
