# Supplementary material for: The prognostic genes model of breast cancer drug resistance based on single-cell sequencing analysis and transcriptome analysis
Source: Clin Exp Med. 2024 May 25;24(1):113. doi: 10.1007/s10238-024-01372-6 (PMC11127859; doi:10.1007/s10238-024-01372-6)
Supplement: Supplementary file 1 — Supplementary file1 (DOCX 387 KB) [file 10238_2024_1372_MOESM1_ESM.docx]

**Supplementary Material**

Table S1 Primer information for the gene

| Gene | Forward Primer | Reverse Primer |
| --- | --- | --- |
| CCT4 | GTTGTCCAGCCTCTGTTGGTA | TCTTCTTCCATTCCAGCCACA |
| CXCL13 | GCTTGAGGTGTAGATGTGTCC | TTGACTTGTTCTTCTTCCAGACTA |
| MTDH | CAGGTGAGGAGAAGTGGAAC | CAGTAGACCCAATGCCAGAA |
| RAB27A | TTGTTGAAGCATTGGTAACTCC | TACAGGGTAGAGAACCGCT |
| PSMD2 | ATTGTATGGGCTGGTGGCTG | TATGCGTCTGGAACCCTGTG |
| GAPDH | GCACCGTCAAGGCTGAGAA | TGGTGAAGACGCCAGTGGA |


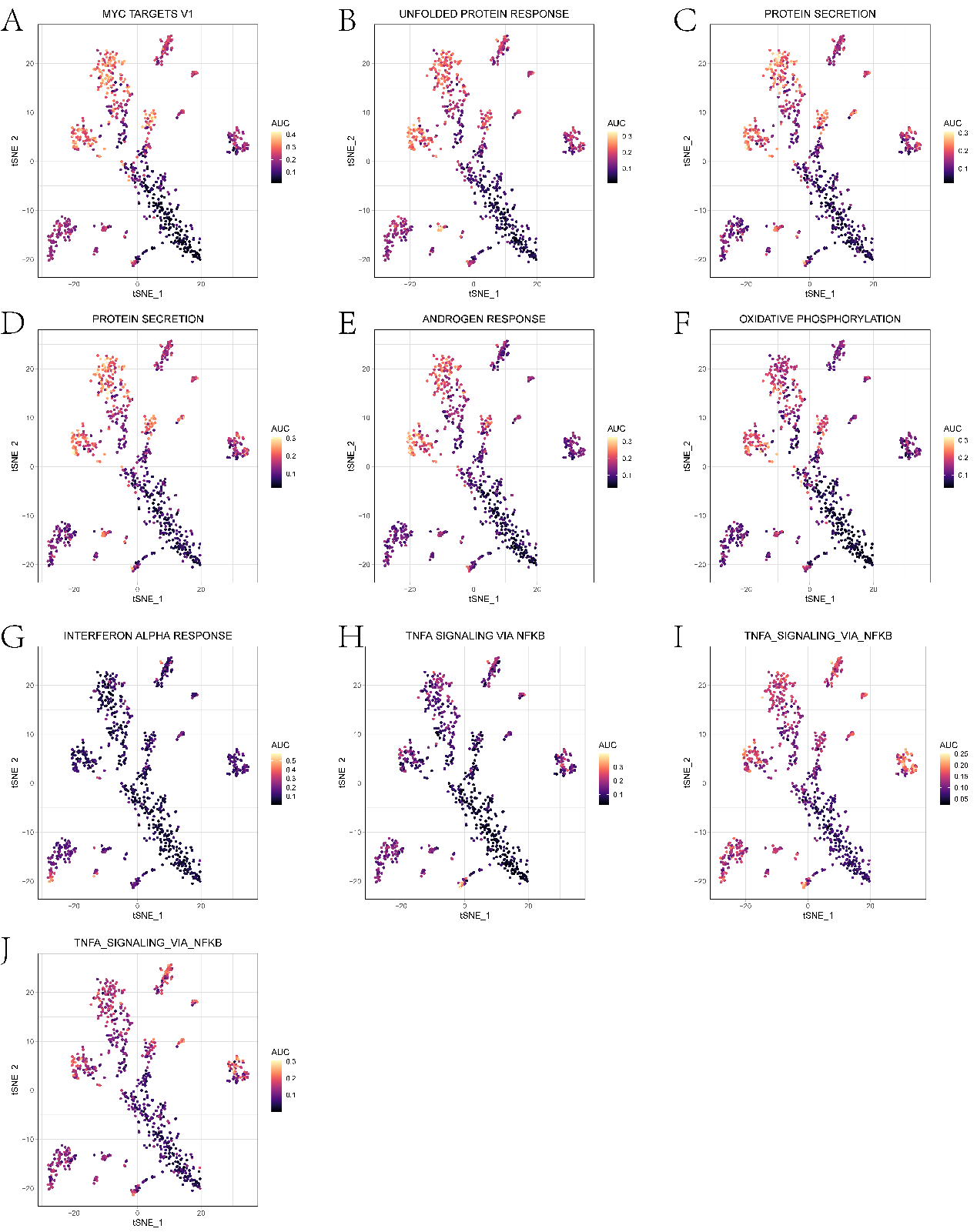


Figure S1 The dimensionality reduction of the top 10 pathways obtained based on the AUCell algorithm is visualized
