# Supplementary figures and images for: The prognostic genes model of breast cancer drug resistance based on single-cell sequencing analysis and transcriptome analysis (part 2 of 2)
Source: Clin Exp Med. 2024 May 25;24(1):113. doi: 10.1007/s10238-024-01372-6 (PMC11127859; doi:10.1007/s10238-024-01372-6)

risk low high

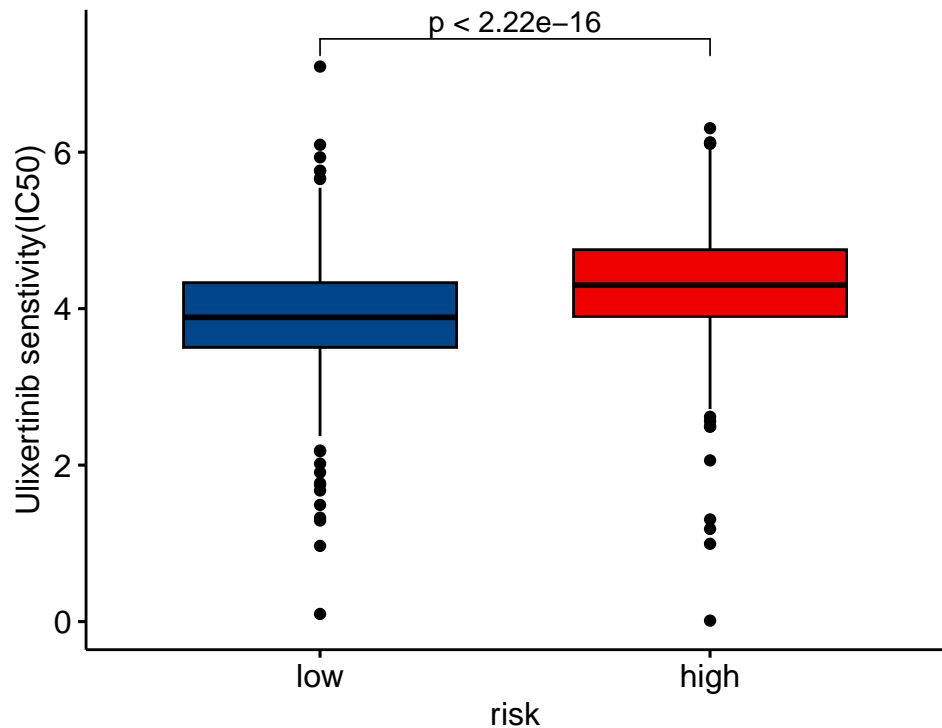

Supplement: Supplementary file 2 — Supplementary file2 (ZIP 3179 KB) [file 10238_2024_1372_MOESM2_ESM.zip › Supplementary Material/Drug1/drugSenstivity.Ulixertinib.pdf]

risk low high

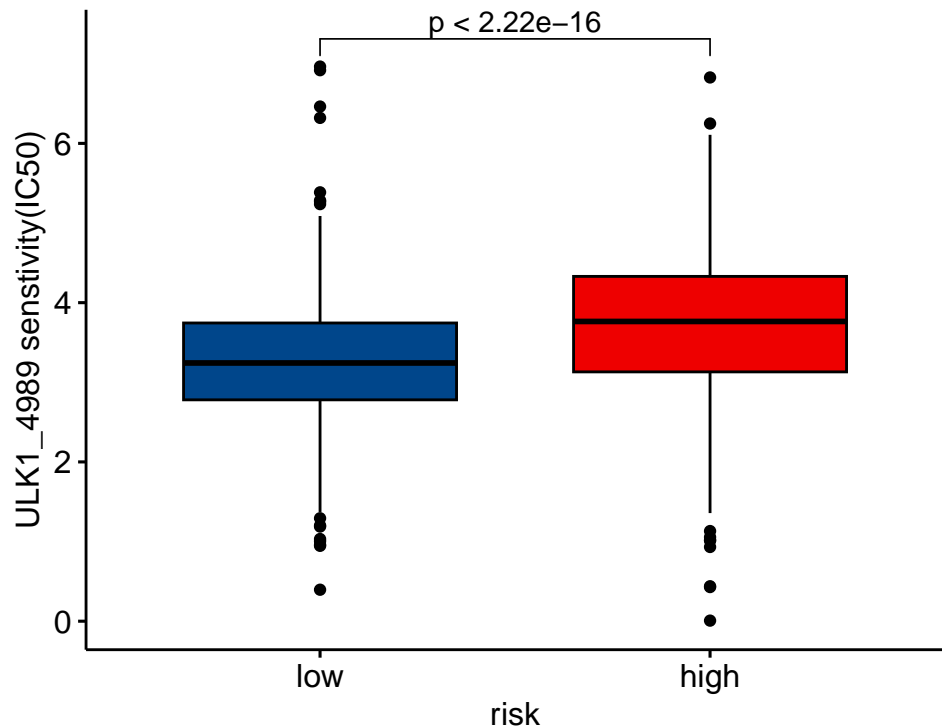

Supplement: Supplementary file 2 — Supplementary file2 (ZIP 3179 KB) [file 10238_2024_1372_MOESM2_ESM.zip › Supplementary Material/Drug1/drugSenstivity.ULK1_4989.pdf]

risk low high

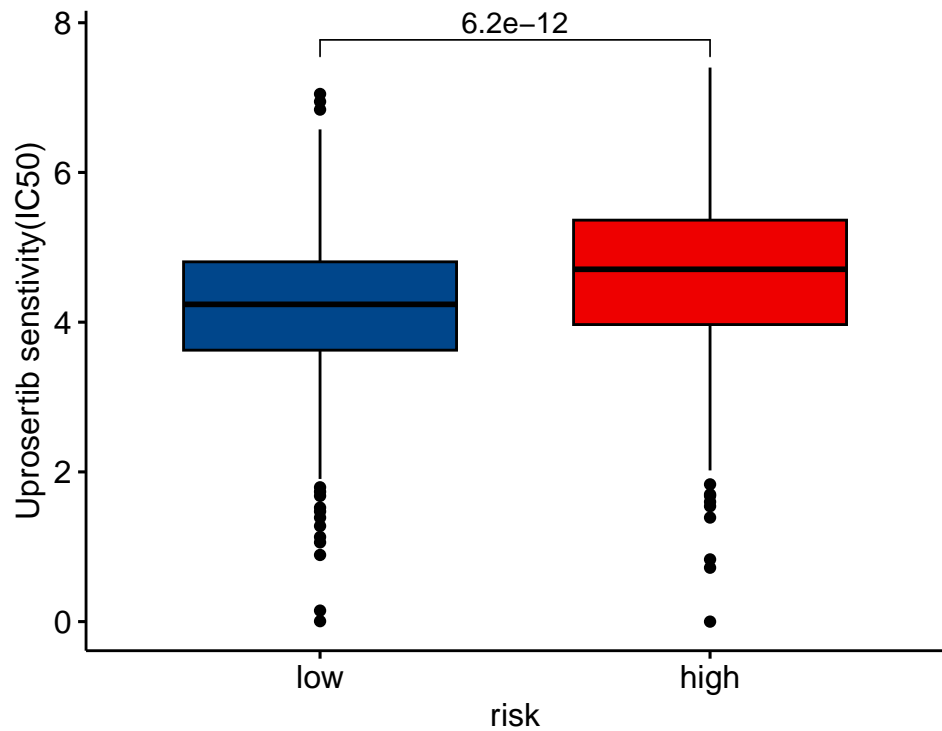

Supplement: Supplementary file 2 — Supplementary file2 (ZIP 3179 KB) [file 10238_2024_1372_MOESM2_ESM.zip › Supplementary Material/Drug1/drugSenstivity.Uprosertib.pdf]

risk low high

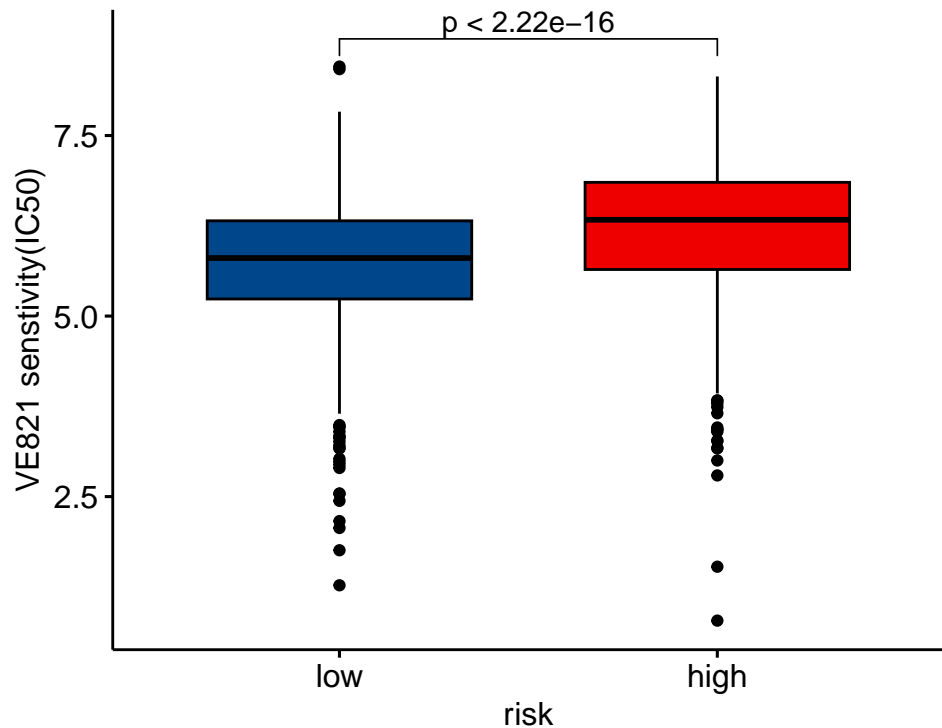

Supplement: Supplementary file 2 — Supplementary file2 (ZIP 3179 KB) [file 10238_2024_1372_MOESM2_ESM.zip › Supplementary Material/Drug1/drugSenstivity.VE821.pdf]

risk low high

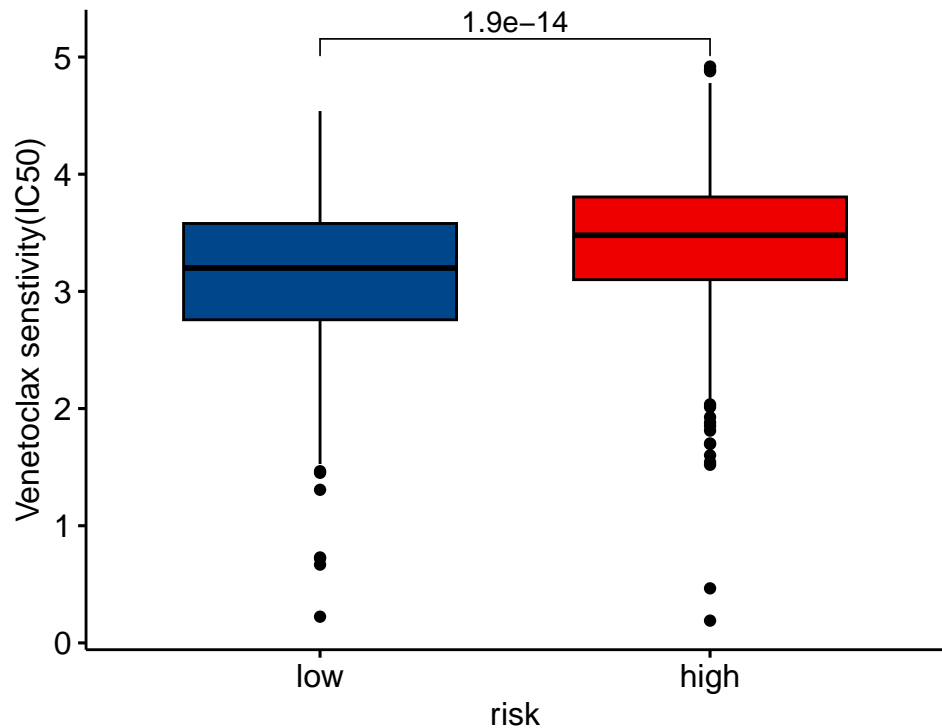

Supplement: Supplementary file 2 — Supplementary file2 (ZIP 3179 KB) [file 10238_2024_1372_MOESM2_ESM.zip › Supplementary Material/Drug1/drugSenstivity.Venetoclax.pdf]

risk low high

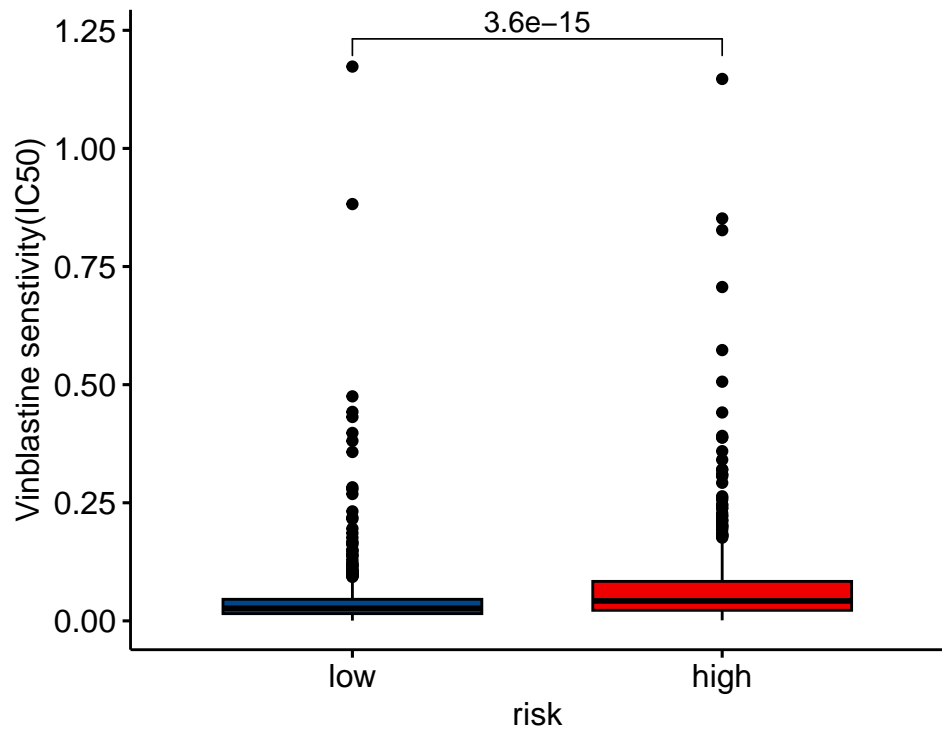

Supplement: Supplementary file 2 — Supplementary file2 (ZIP 3179 KB) [file 10238_2024_1372_MOESM2_ESM.zip › Supplementary Material/Drug1/drugSenstivity.Vinblastine.pdf]

risk low high

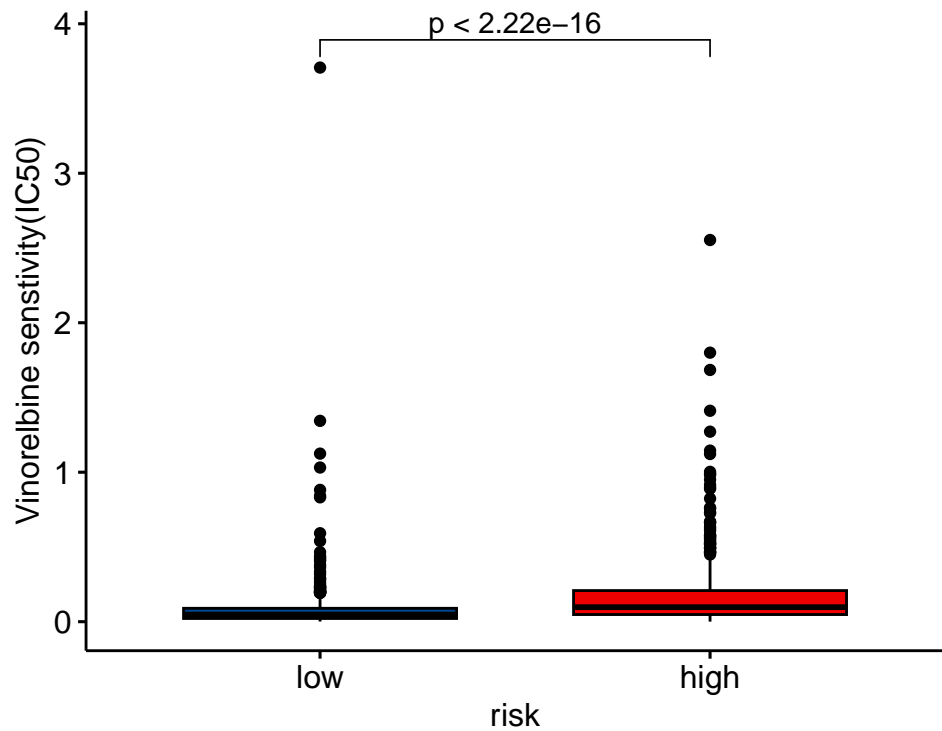

Supplement: Supplementary file 2 — Supplementary file2 (ZIP 3179 KB) [file 10238_2024_1372_MOESM2_ESM.zip › Supplementary Material/Drug1/drugSenstivity.Vinorelbine.pdf]

risk low high

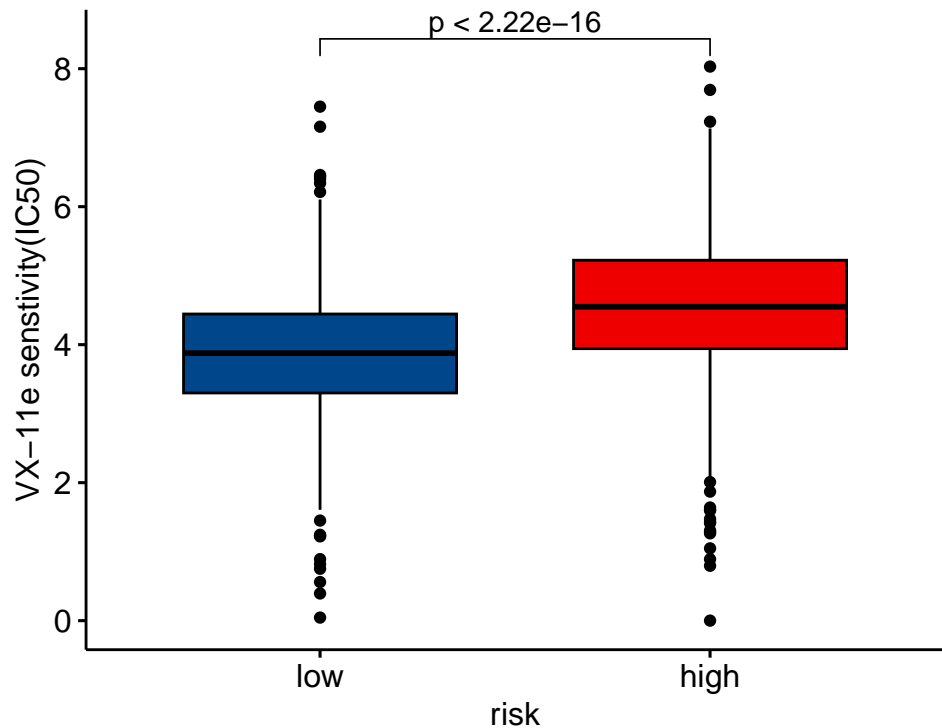

Supplement: Supplementary file 2 — Supplementary file2 (ZIP 3179 KB) [file 10238_2024_1372_MOESM2_ESM.zip › Supplementary Material/Drug1/drugSenstivity.VX-11e.pdf]

risk low high

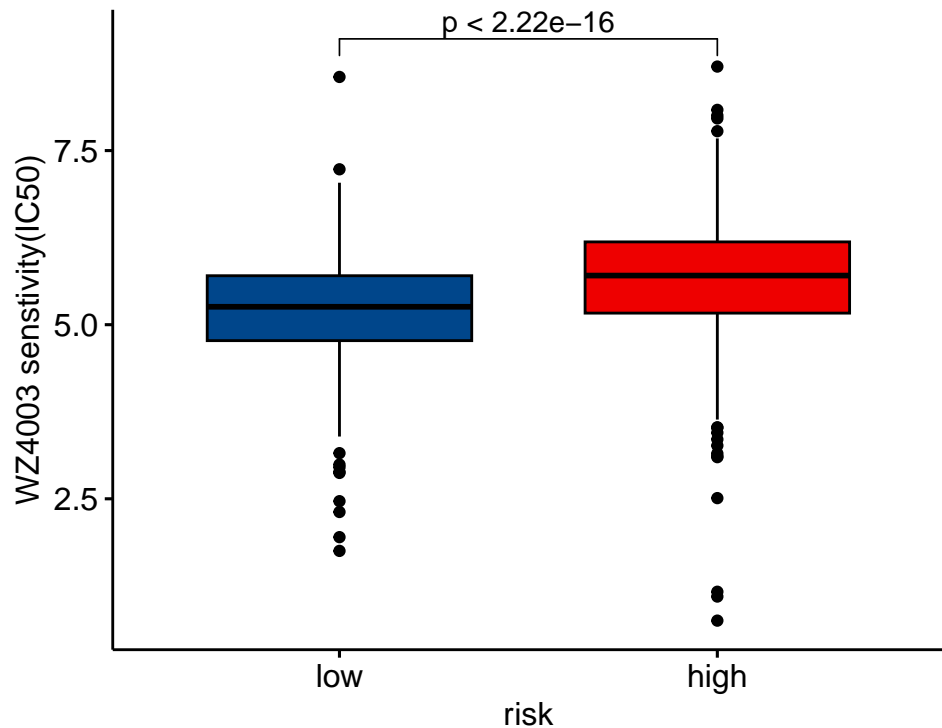

Supplement: Supplementary file 2 — Supplementary file2 (ZIP 3179 KB) [file 10238_2024_1372_MOESM2_ESM.zip › Supplementary Material/Drug1/drugSenstivity.WZ4003.pdf]

risk low high

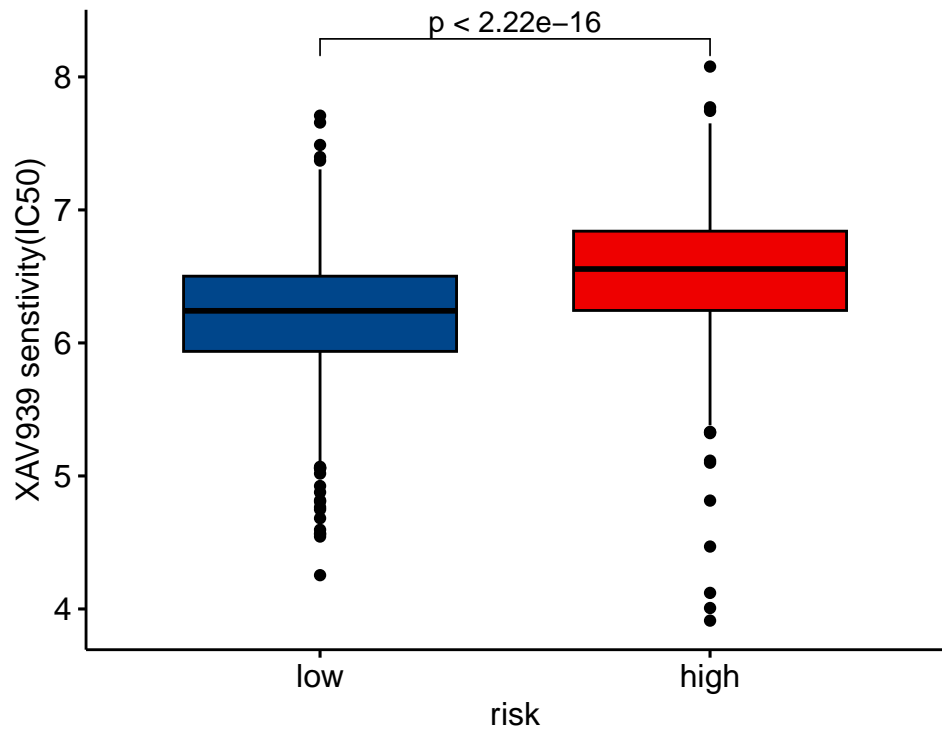

Supplement: Supplementary file 2 — Supplementary file2 (ZIP 3179 KB) [file 10238_2024_1372_MOESM2_ESM.zip › Supplementary Material/Drug1/drugSenstivity.XAV939.pdf]

YK-4-279 sensitivity(IC50)

risk low high

$p < 2.22e-16$

low

high

risk

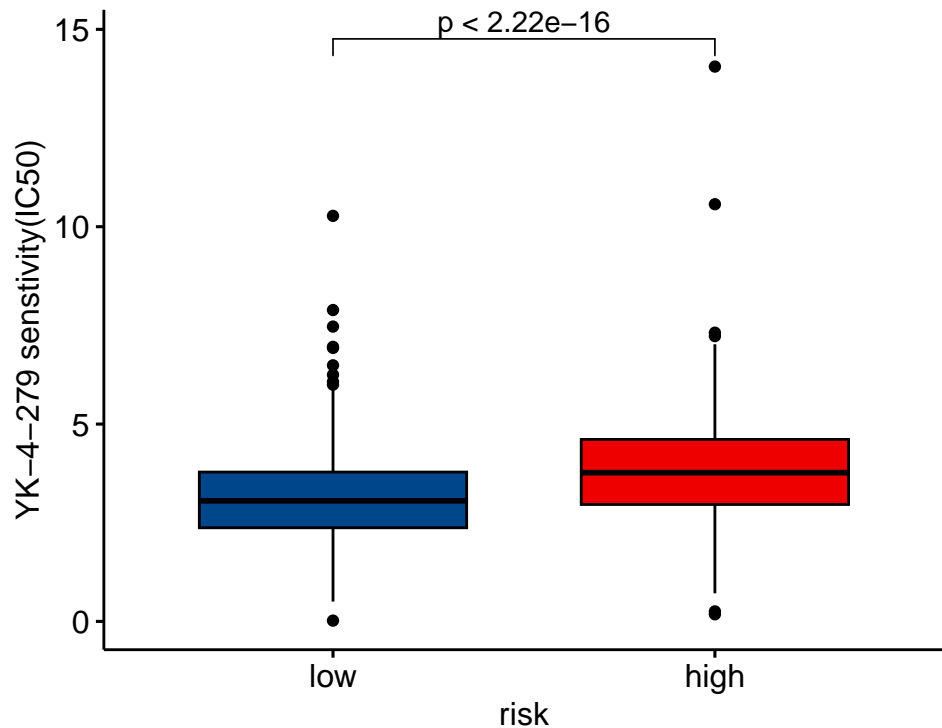

Supplement: Supplementary file 2 — Supplementary file2 (ZIP 3179 KB) [file 10238_2024_1372_MOESM2_ESM.zip › Supplementary Material/Drug1/drugSenstivity.YK-4-279.pdf]

risk low high

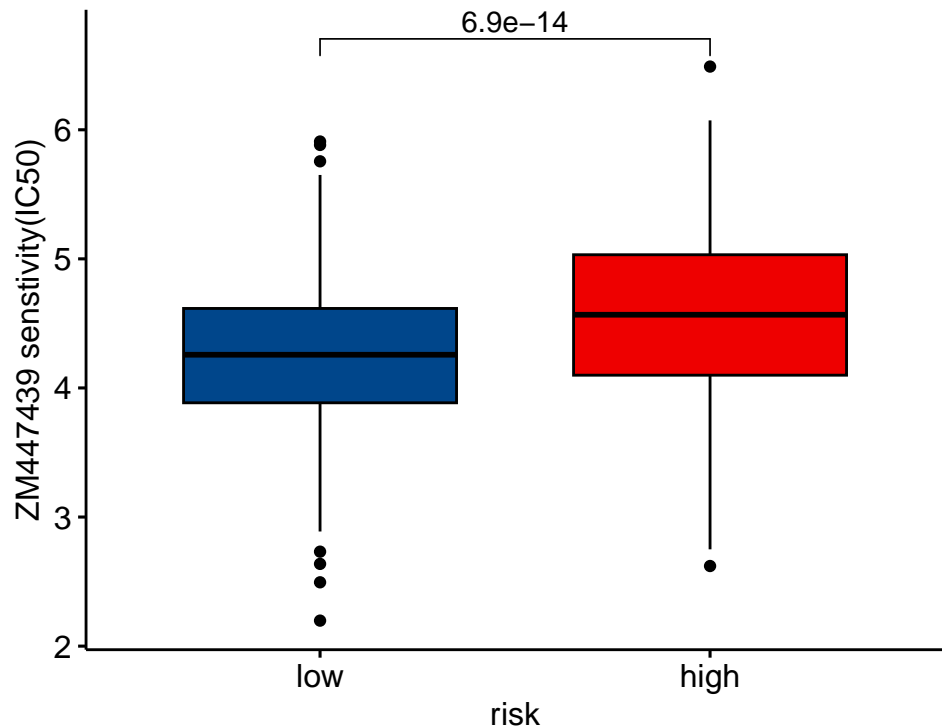

Supplement: Supplementary file 2 — Supplementary file2 (ZIP 3179 KB) [file 10238_2024_1372_MOESM2_ESM.zip › Supplementary Material/Drug1/drugSenstivity.ZM447439.pdf]

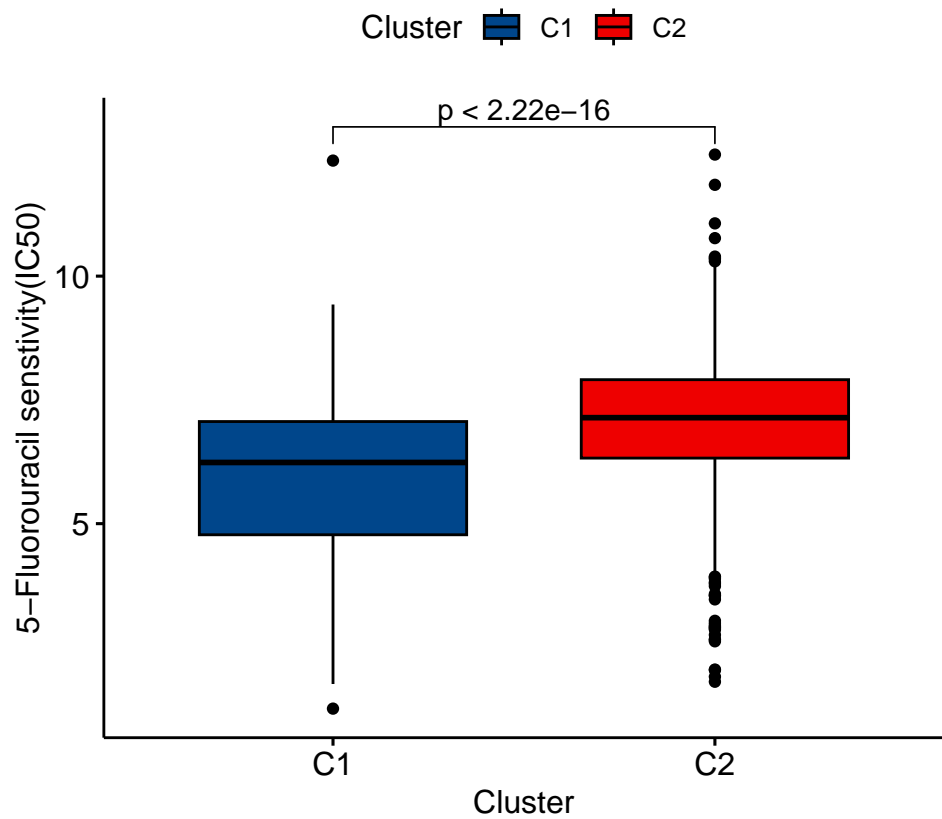

Supplement: Supplementary file 2 — Supplementary file2 (ZIP 3179 KB) [file 10238_2024_1372_MOESM2_ESM.zip › Supplementary Material/Drug2/drugSenstivity.5-Fluorouracil.pdf]

Cluster C1 C2

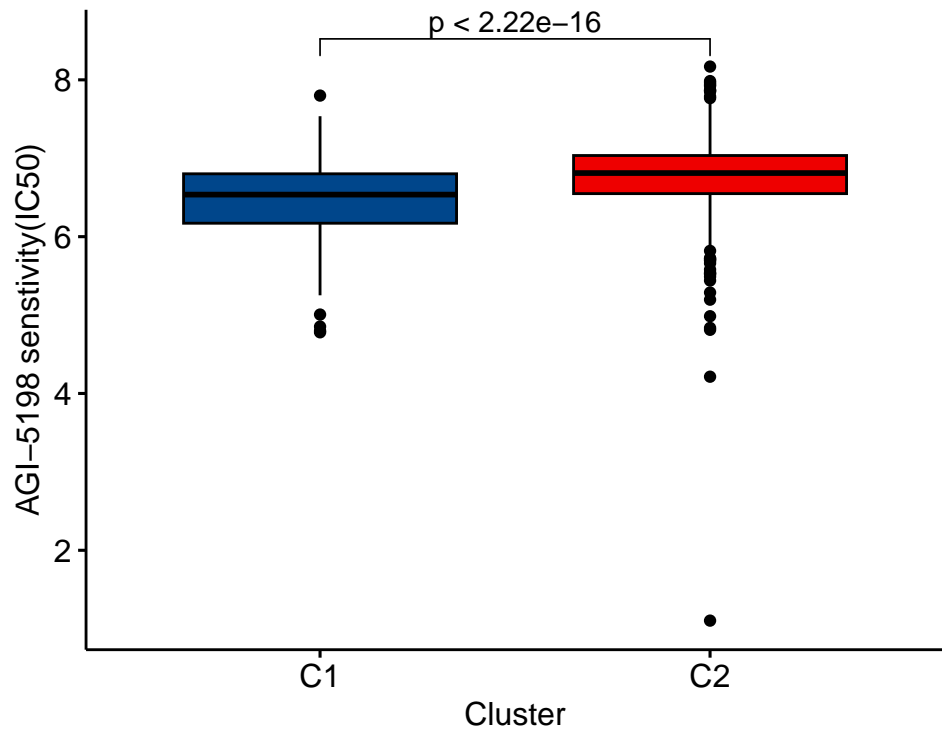

Supplement: Supplementary file 2 — Supplementary file2 (ZIP 3179 KB) [file 10238_2024_1372_MOESM2_ESM.zip › Supplementary Material/Drug2/drugSenstivity.AGI-5198.pdf]

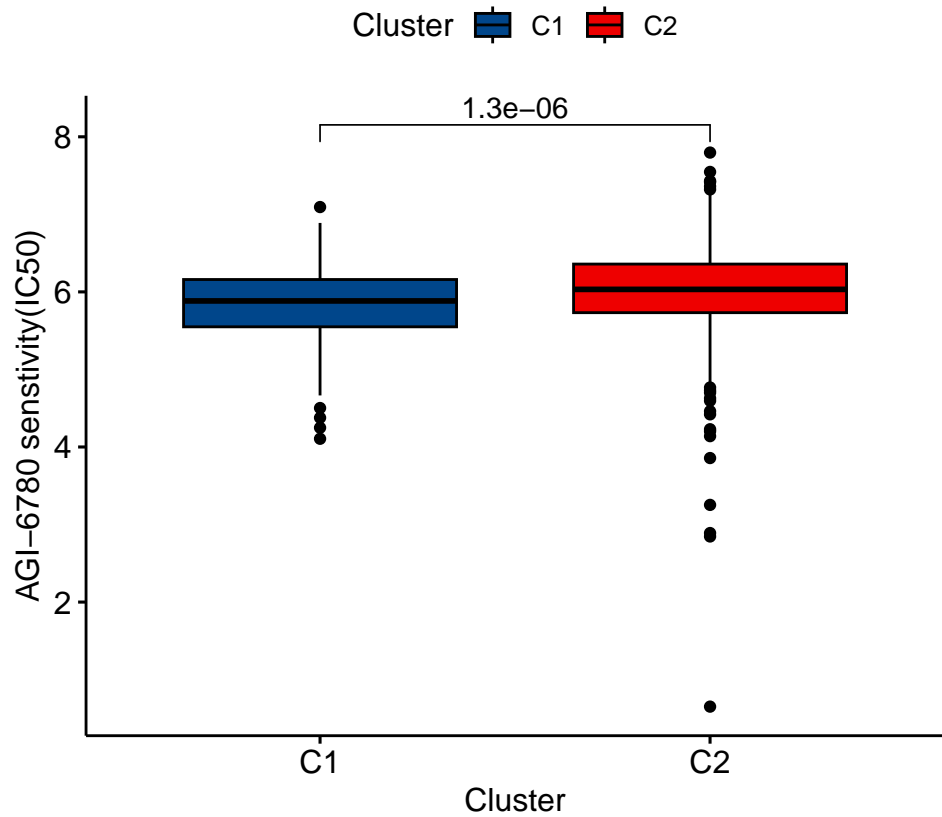

Supplement: Supplementary file 2 — Supplementary file2 (ZIP 3179 KB) [file 10238_2024_1372_MOESM2_ESM.zip › Supplementary Material/Drug2/drugSenstivity.AGI-6780.pdf]

Cluster 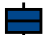 C1 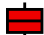 C2

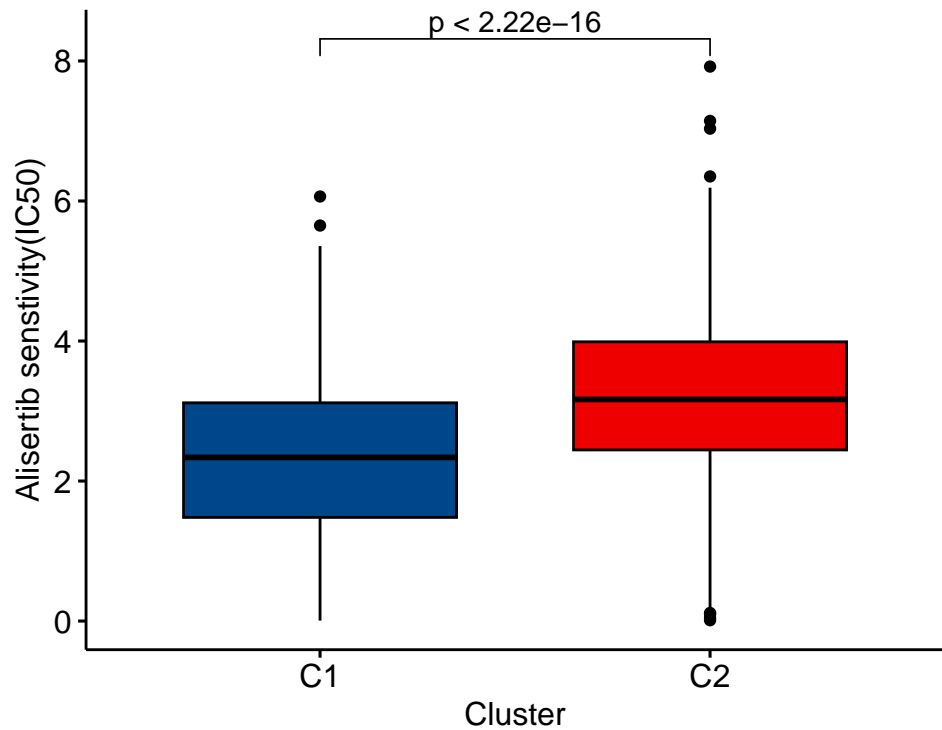

Supplement: Supplementary file 2 — Supplementary file2 (ZIP 3179 KB) [file 10238_2024_1372_MOESM2_ESM.zip › Supplementary Material/Drug2/drugSenstivity.Alisertib.pdf]

Cluster 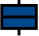 C1 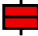 C2

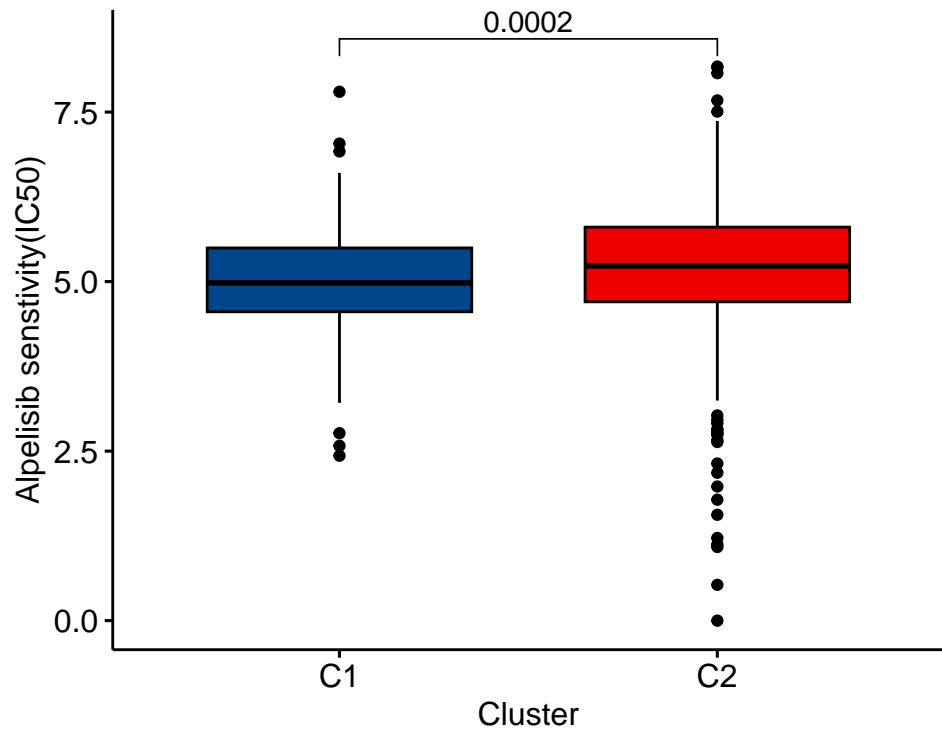

Supplement: Supplementary file 2 — Supplementary file2 (ZIP 3179 KB) [file 10238_2024_1372_MOESM2_ESM.zip › Supplementary Material/Drug2/drugSenstivity.Alpelisib.pdf]

Cluster 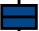 C1 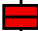 C2

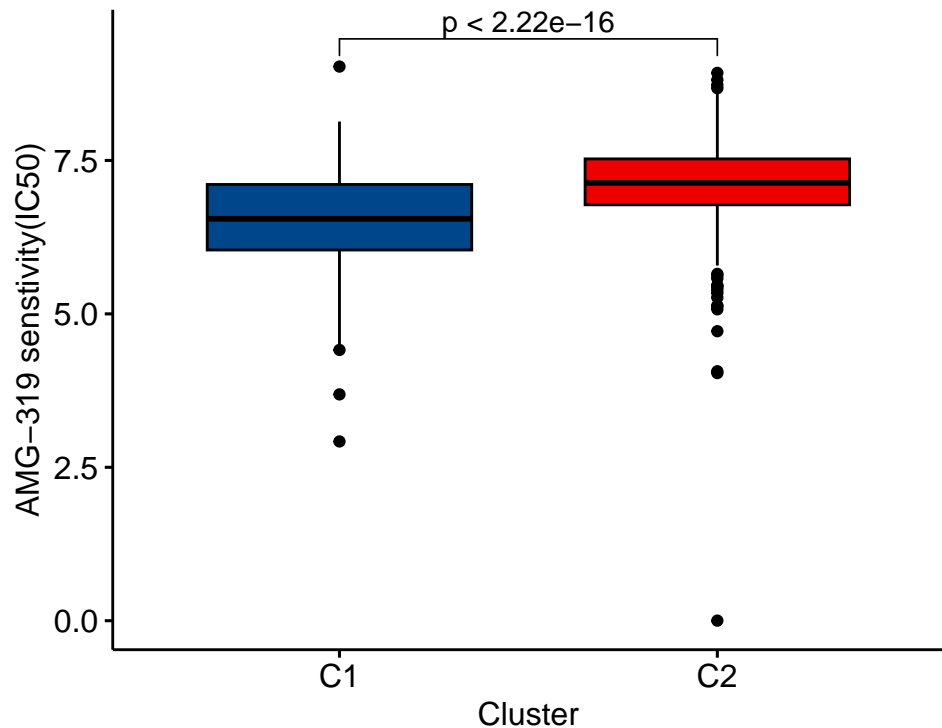

Supplement: Supplementary file 2 — Supplementary file2 (ZIP 3179 KB) [file 10238_2024_1372_MOESM2_ESM.zip › Supplementary Material/Drug2/drugSenstivity.AMG-319.pdf]

AT13148 sensitivity(IC50)

Cluster

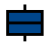

C1

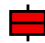

C2

$4.5e-11$

C1

C2

Cluster

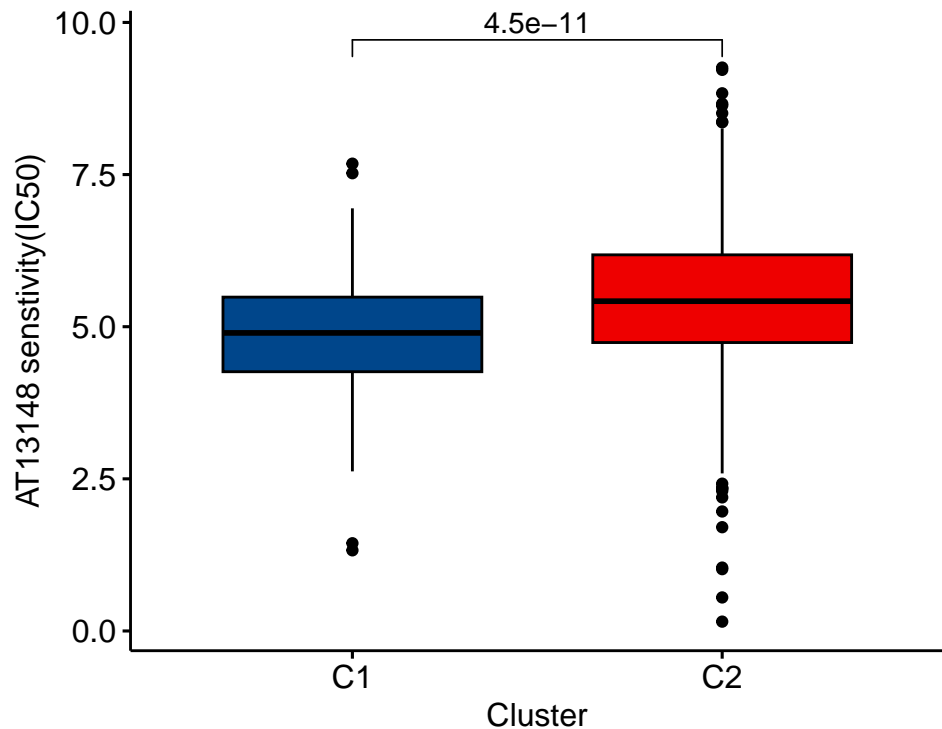

Supplement: Supplementary file 2 — Supplementary file2 (ZIP 3179 KB) [file 10238_2024_1372_MOESM2_ESM.zip › Supplementary Material/Drug2/drugSenstivity.AT13148.pdf]

Cluster 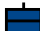 C1 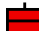 C2

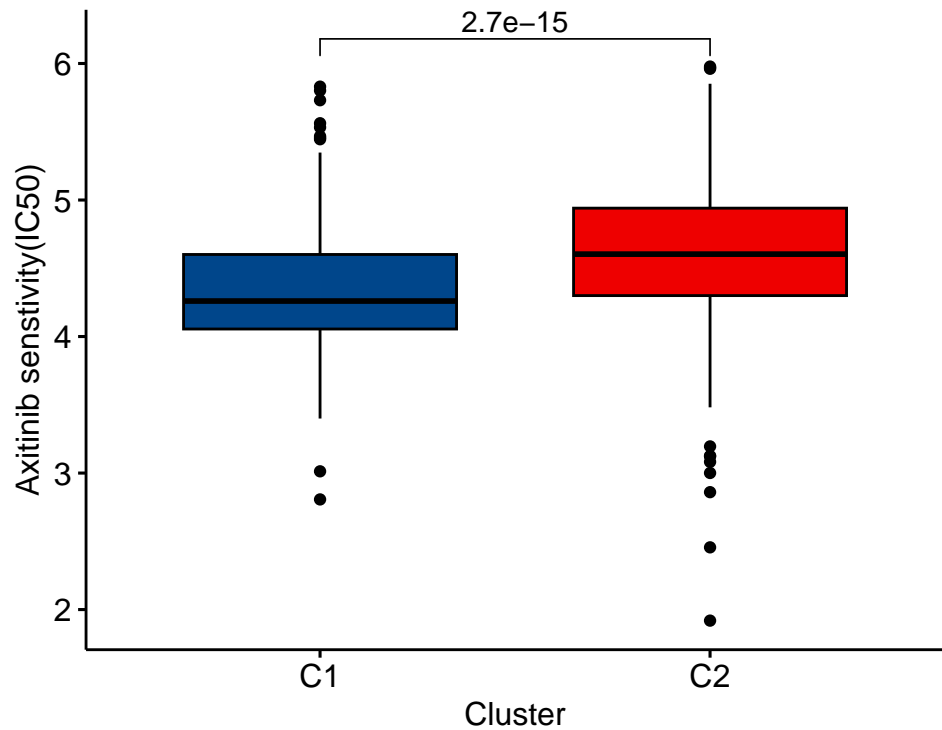

Supplement: Supplementary file 2 — Supplementary file2 (ZIP 3179 KB) [file 10238_2024_1372_MOESM2_ESM.zip › Supplementary Material/Drug2/drugSenstivity.Axitinib.pdf]

Cluster 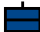 C1 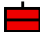 C2

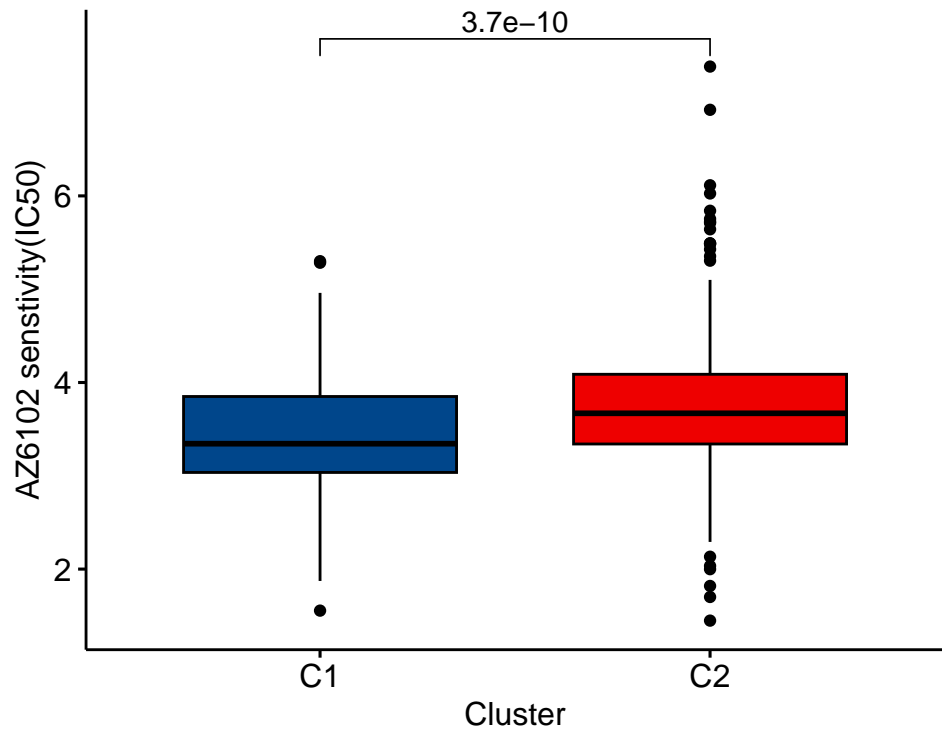

Supplement: Supplementary file 2 — Supplementary file2 (ZIP 3179 KB) [file 10238_2024_1372_MOESM2_ESM.zip › Supplementary Material/Drug2/drugSenstivity.AZ6102.pdf]

Cluster 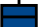 C1 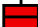 C2

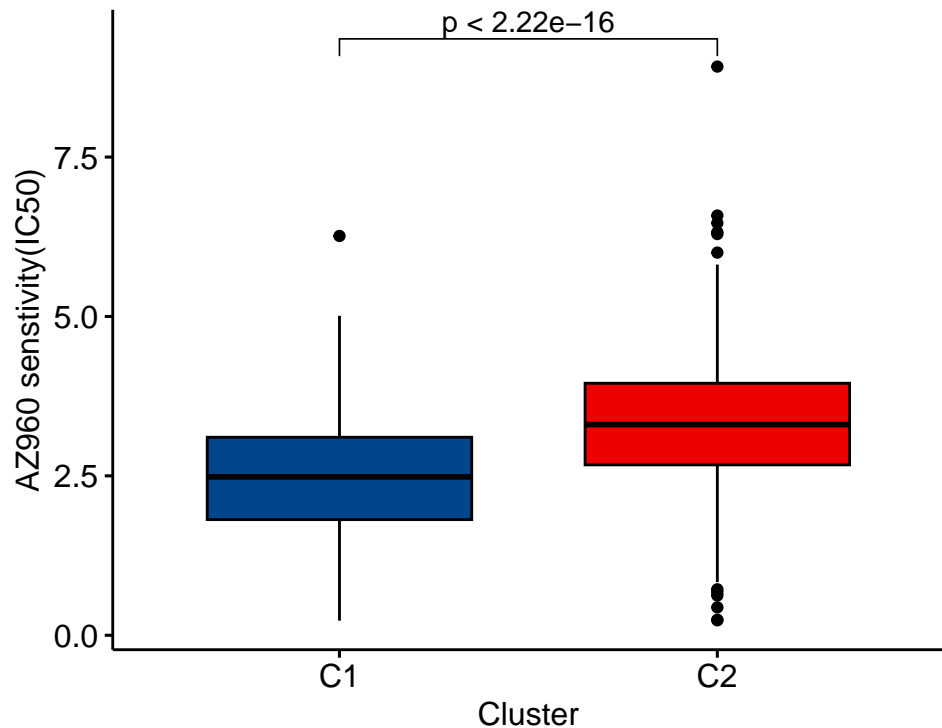

Supplement: Supplementary file 2 — Supplementary file2 (ZIP 3179 KB) [file 10238_2024_1372_MOESM2_ESM.zip › Supplementary Material/Drug2/drugSenstivity.AZ960.pdf]

AZD1208 sensitivity(IC50)

Cluster

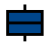

C1

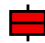

C2

1.9e-05

C1

C2

Cluster

10.0  
7.5  
5.0  
2.5  
0.0

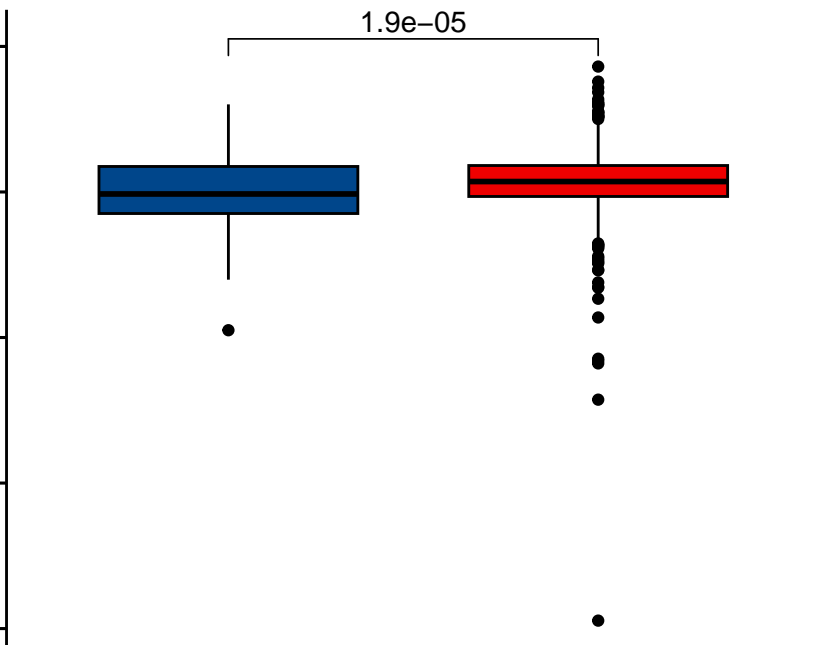

Supplement: Supplementary file 2 — Supplementary file2 (ZIP 3179 KB) [file 10238_2024_1372_MOESM2_ESM.zip › Supplementary Material/Drug2/drugSenstivity.AZD1208.pdf]

Cluster 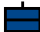 C1 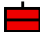 C2

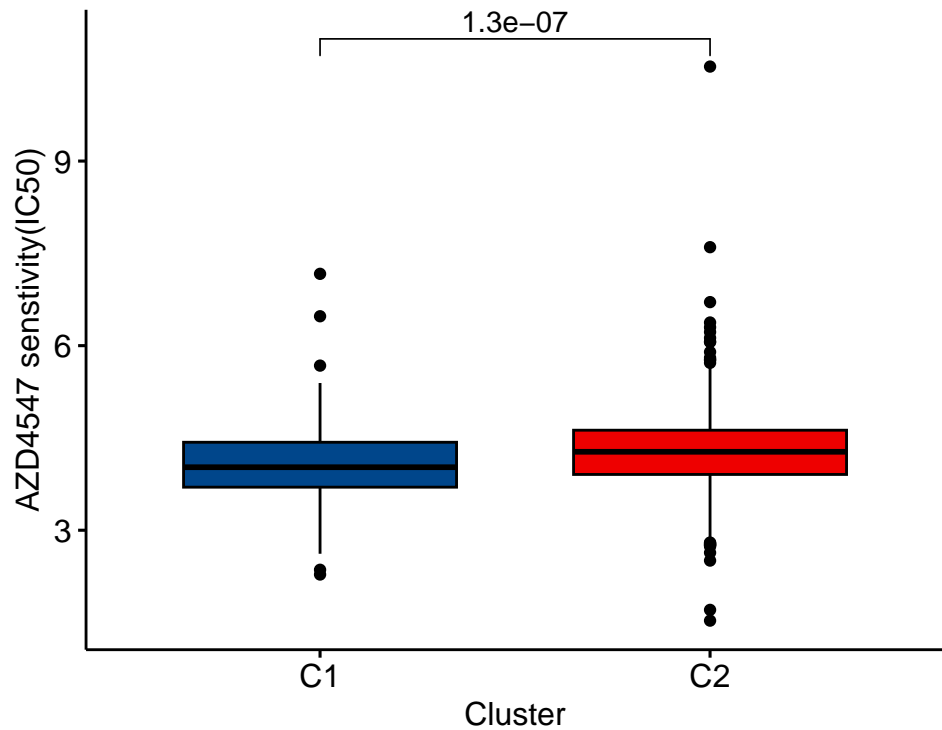

Supplement: Supplementary file 2 — Supplementary file2 (ZIP 3179 KB) [file 10238_2024_1372_MOESM2_ESM.zip › Supplementary Material/Drug2/drugSenstivity.AZD4547.pdf]

Cluster

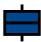

C1

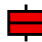

C2

$1.4e-14$

AZD5153 sensitivity(IC50)

7.5  
5.0  
2.5  
0.0

C1

C2

Cluster

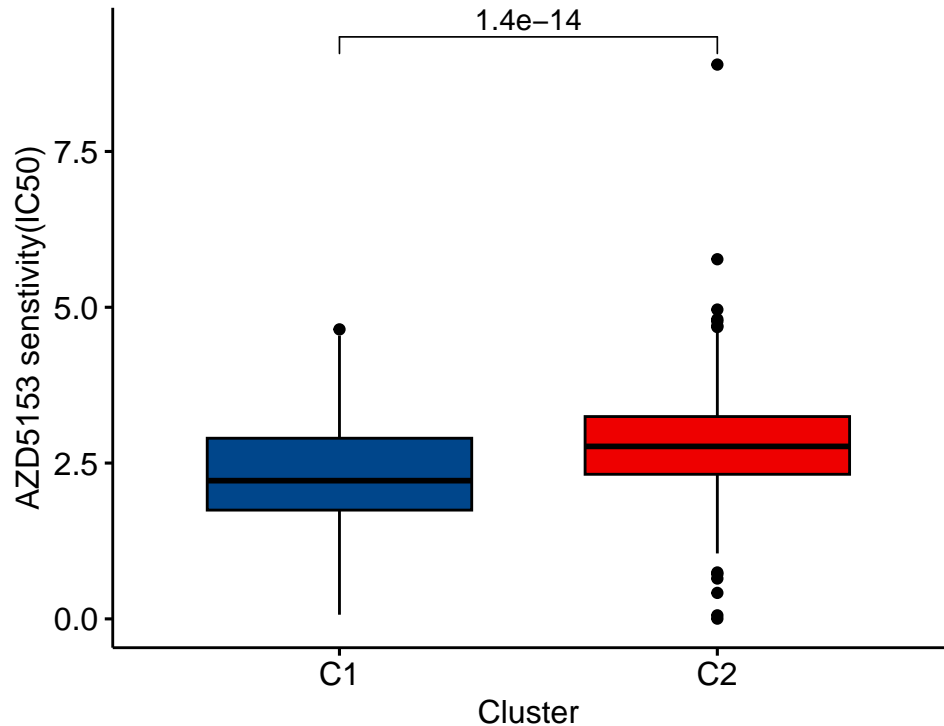

Supplement: Supplementary file 2 — Supplementary file2 (ZIP 3179 KB) [file 10238_2024_1372_MOESM2_ESM.zip › Supplementary Material/Drug2/drugSenstivity.AZD5153.pdf]

Cluster 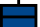 C1 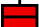 C2

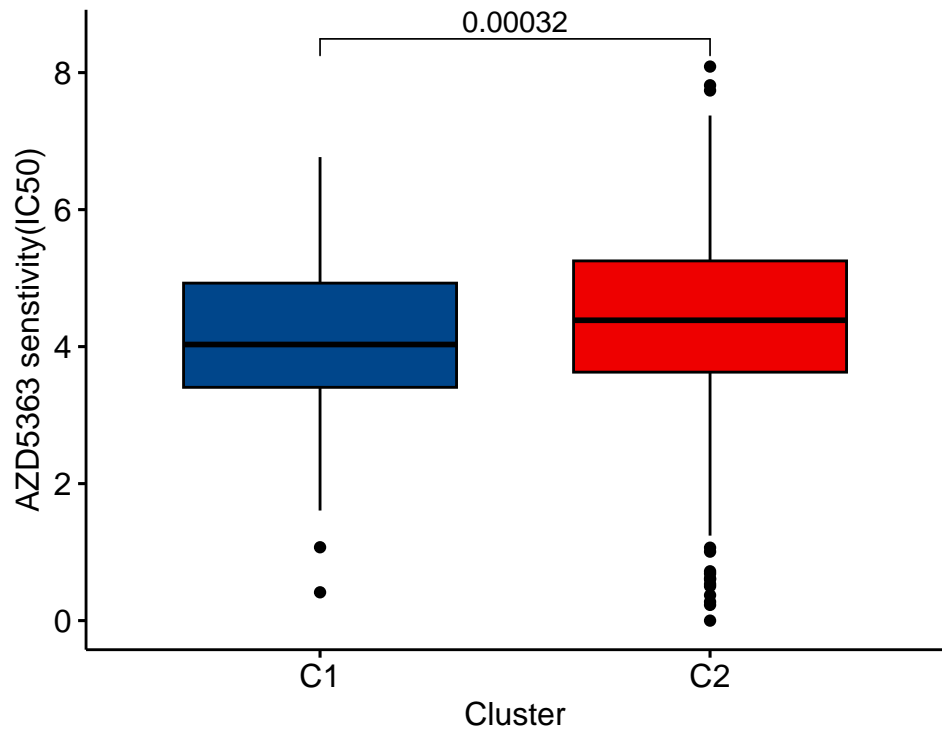

Supplement: Supplementary file 2 — Supplementary file2 (ZIP 3179 KB) [file 10238_2024_1372_MOESM2_ESM.zip › Supplementary Material/Drug2/drugSenstivity.AZD5363.pdf]

Cluster 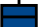 C1 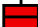 C2

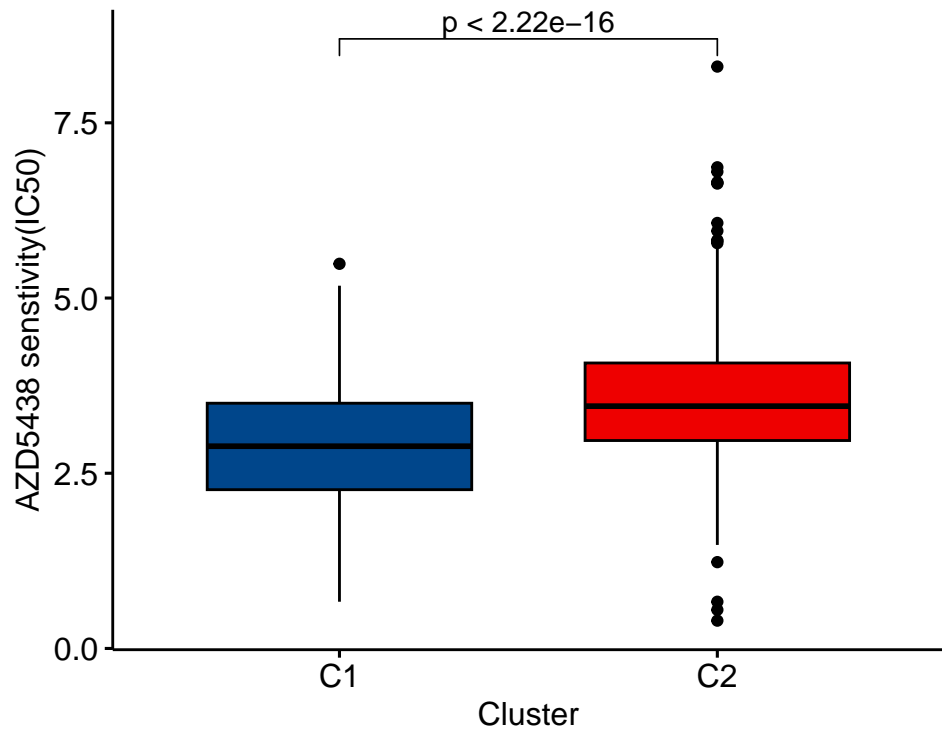

Supplement: Supplementary file 2 — Supplementary file2 (ZIP 3179 KB) [file 10238_2024_1372_MOESM2_ESM.zip › Supplementary Material/Drug2/drugSenstivity.AZD5438.pdf]

Cluster 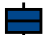 C1 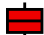 C2

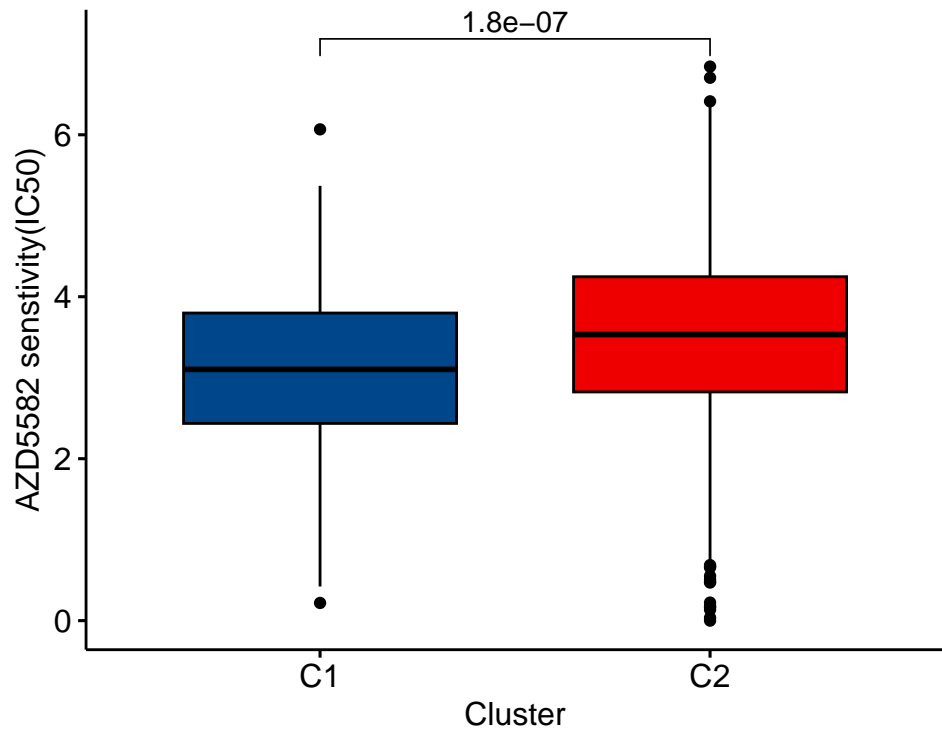

Supplement: Supplementary file 2 — Supplementary file2 (ZIP 3179 KB) [file 10238_2024_1372_MOESM2_ESM.zip › Supplementary Material/Drug2/drugSenstivity.AZD5582.pdf]

AZD5991 sensitivity(IC50)

Cluster

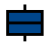

C1

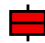

C2

0.0002

C1

C2

Cluster

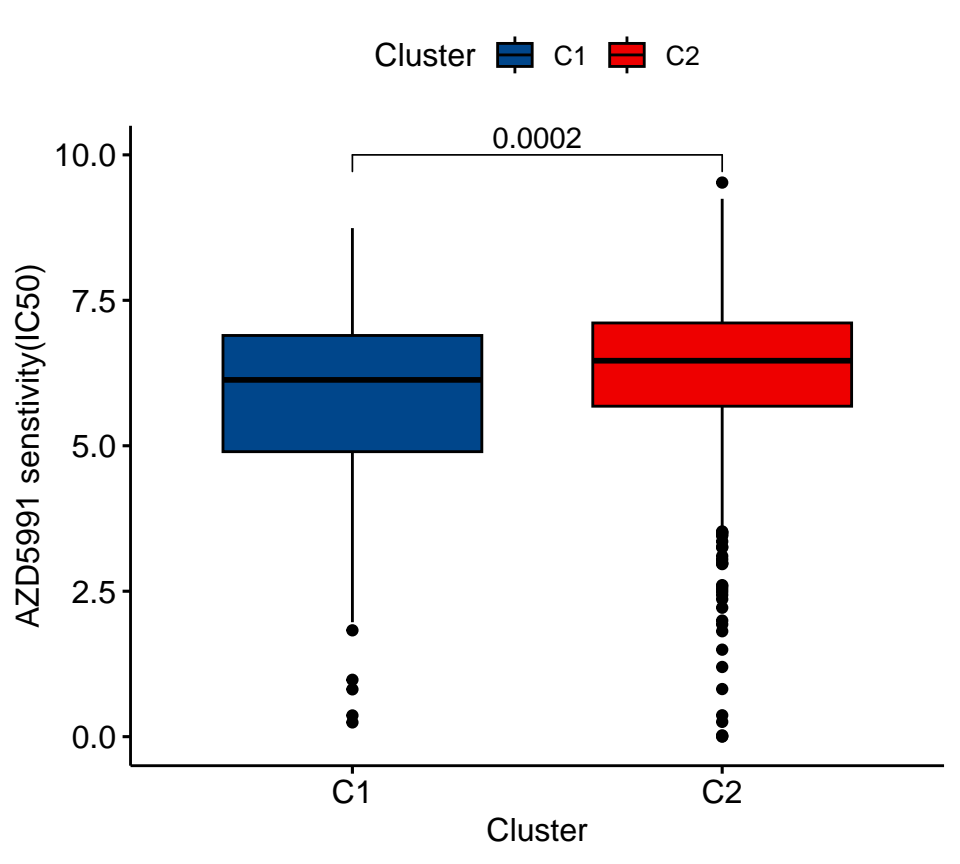

Supplement: Supplementary file 2 — Supplementary file2 (ZIP 3179 KB) [file 10238_2024_1372_MOESM2_ESM.zip › Supplementary Material/Drug2/drugSenstivity.AZD5991.pdf]

Cluster C1 C2

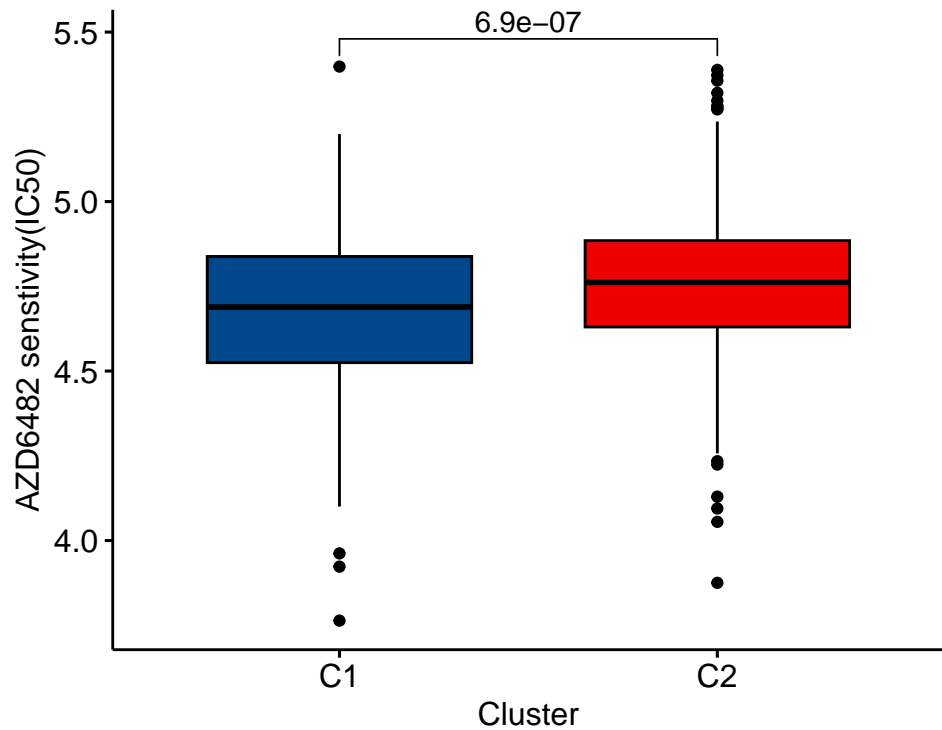

Supplement: Supplementary file 2 — Supplementary file2 (ZIP 3179 KB) [file 10238_2024_1372_MOESM2_ESM.zip › Supplementary Material/Drug2/drugSenstivity.AZD6482.pdf]

AZD6738 sensitivity(IC50)

Cluster

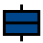

C1

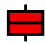

C2

1.4e-14

C1

C2

Cluster

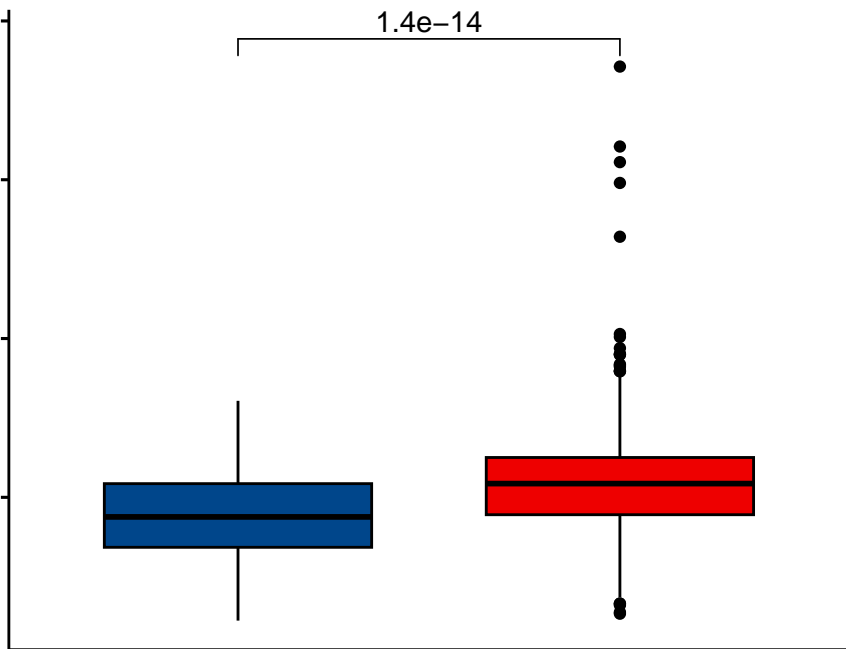

Supplement: Supplementary file 2 — Supplementary file2 (ZIP 3179 KB) [file 10238_2024_1372_MOESM2_ESM.zip › Supplementary Material/Drug2/drugSenstivity.AZD6738.pdf]

AZD7762 sensitivity(IC50)

Cluster

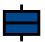

C1

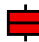

C2

$p < 2.22e-16$

15

10

5

0

C1

C2

Cluster

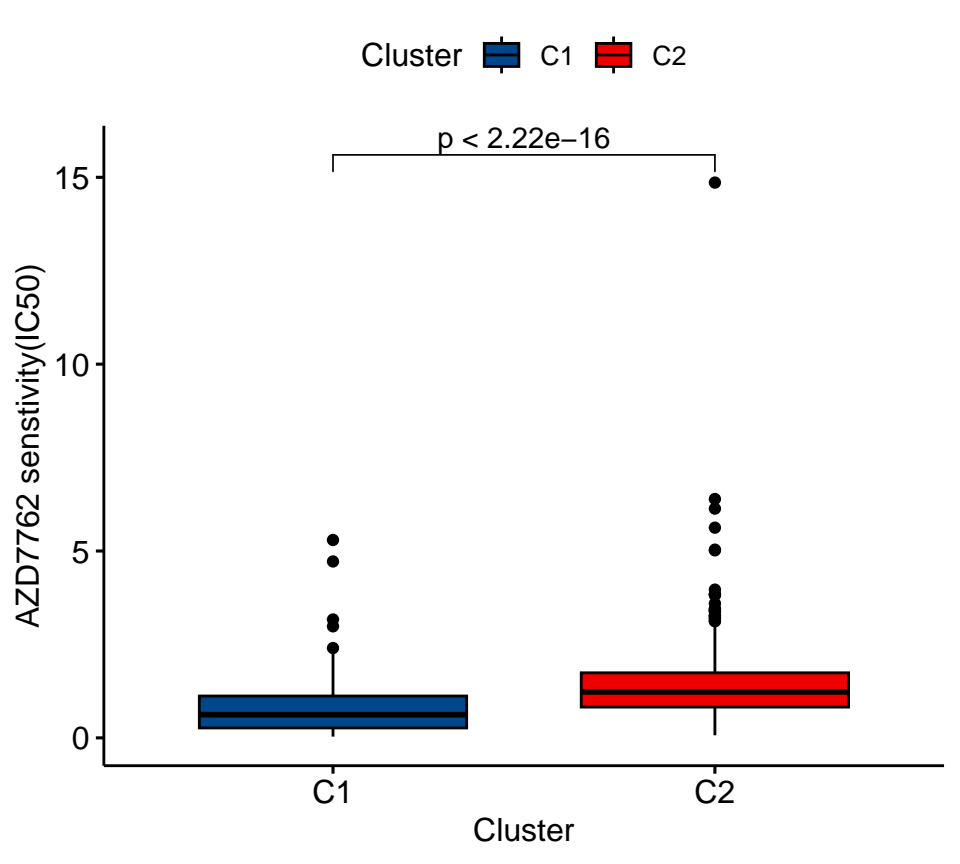

Supplement: Supplementary file 2 — Supplementary file2 (ZIP 3179 KB) [file 10238_2024_1372_MOESM2_ESM.zip › Supplementary Material/Drug2/drugSenstivity.AZD7762.pdf]

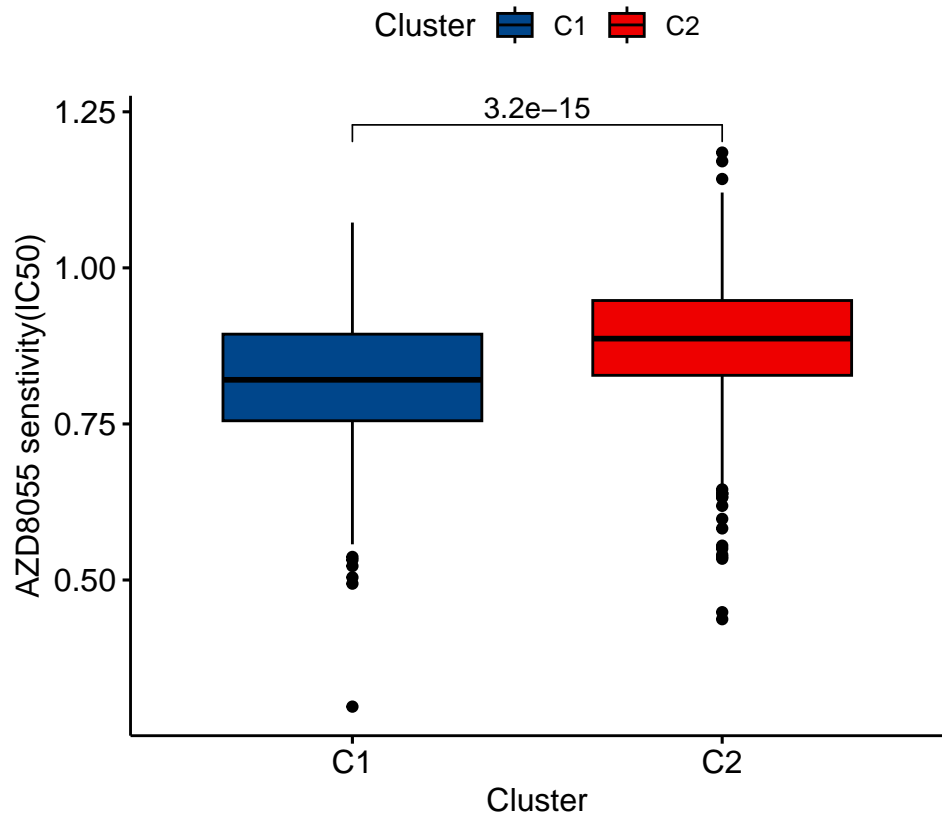

Supplement: Supplementary file 2 — Supplementary file2 (ZIP 3179 KB) [file 10238_2024_1372_MOESM2_ESM.zip › Supplementary Material/Drug2/drugSenstivity.AZD8055.pdf]

AZD8186 sensitivity(IC50)

Cluster

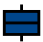

C1

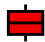

C2

1.3e-06

C1

C2

Cluster

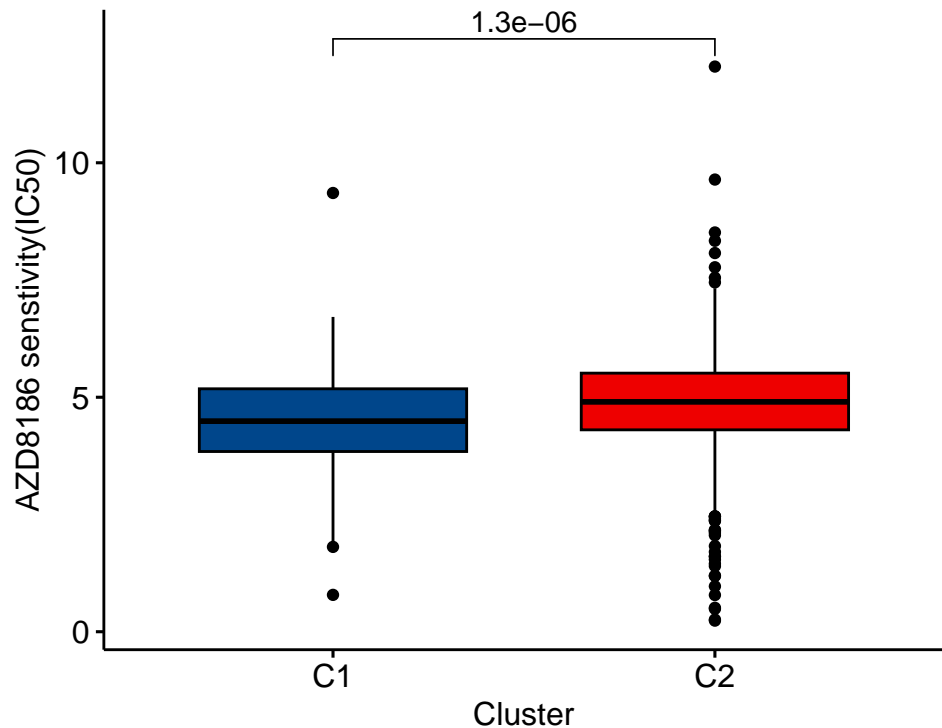

Supplement: Supplementary file 2 — Supplementary file2 (ZIP 3179 KB) [file 10238_2024_1372_MOESM2_ESM.zip › Supplementary Material/Drug2/drugSenstivity.AZD8186.pdf]

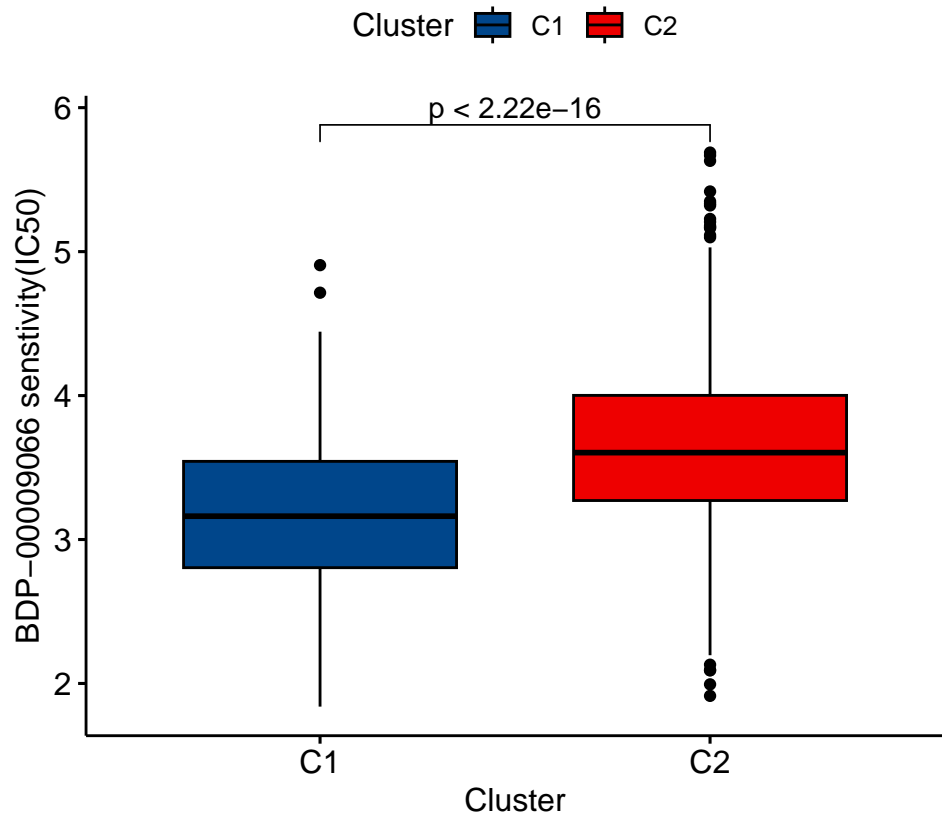

Supplement: Supplementary file 2 — Supplementary file2 (ZIP 3179 KB) [file 10238_2024_1372_MOESM2_ESM.zip › Supplementary Material/Drug2/drugSenstivity.BDP-00009066.pdf]

Cluster 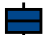 C1 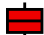 C2

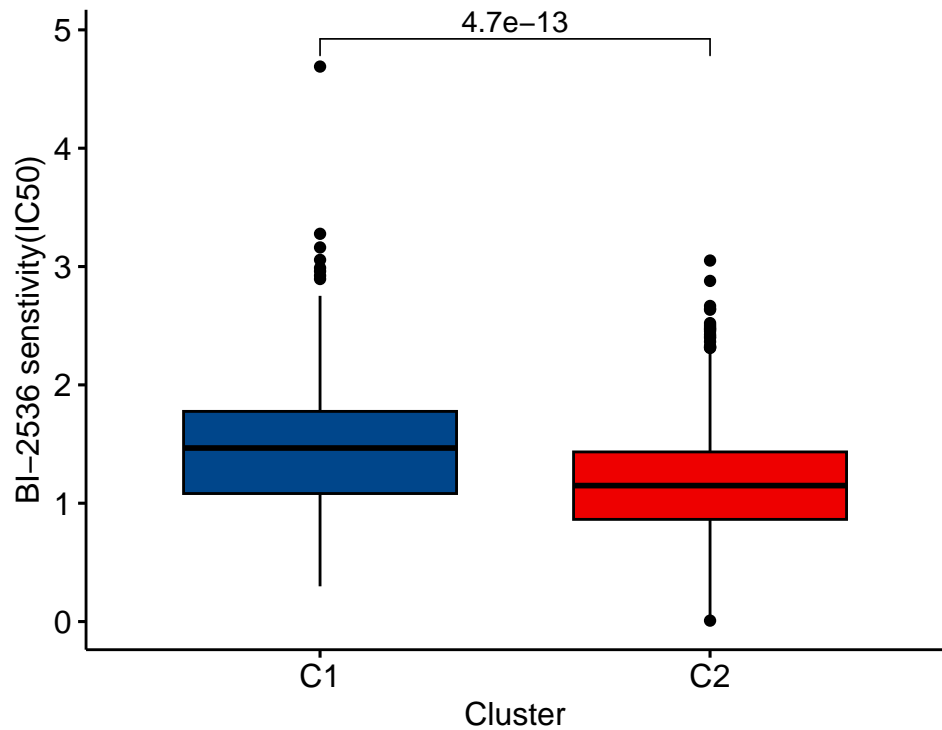

Supplement: Supplementary file 2 — Supplementary file2 (ZIP 3179 KB) [file 10238_2024_1372_MOESM2_ESM.zip › Supplementary Material/Drug2/drugSenstivity.BI-2536.pdf]

Cluster 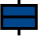 C1 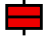 C2

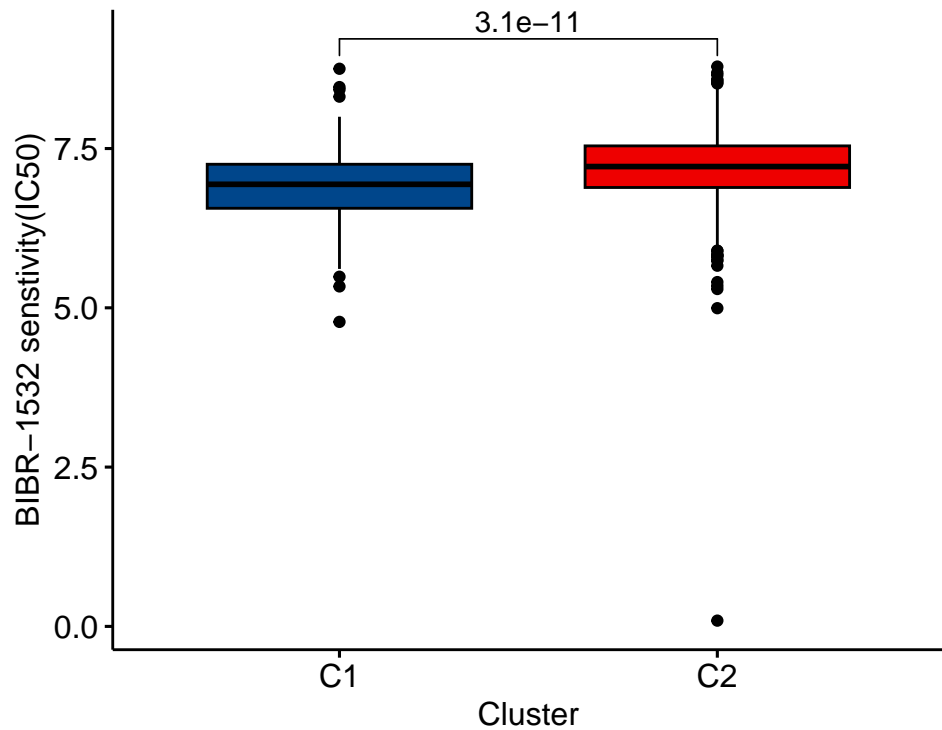

Supplement: Supplementary file 2 — Supplementary file2 (ZIP 3179 KB) [file 10238_2024_1372_MOESM2_ESM.zip › Supplementary Material/Drug2/drugSenstivity.BIBR-1532.pdf]

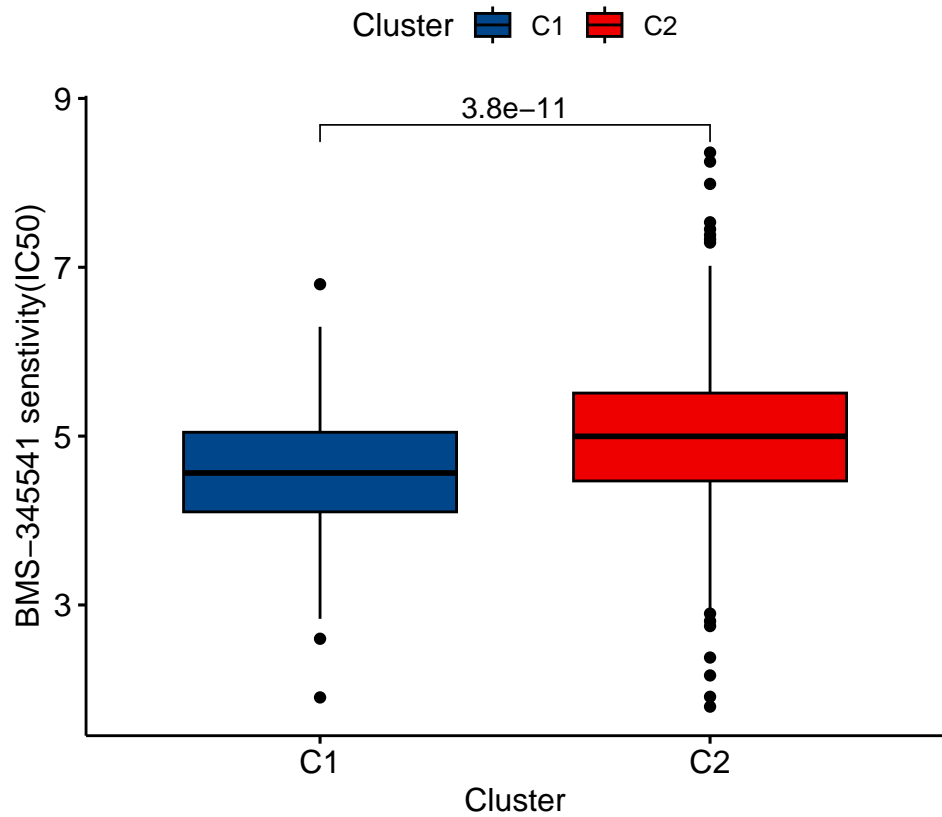

Supplement: Supplementary file 2 — Supplementary file2 (ZIP 3179 KB) [file 10238_2024_1372_MOESM2_ESM.zip › Supplementary Material/Drug2/drugSenstivity.BMS-345541.pdf]

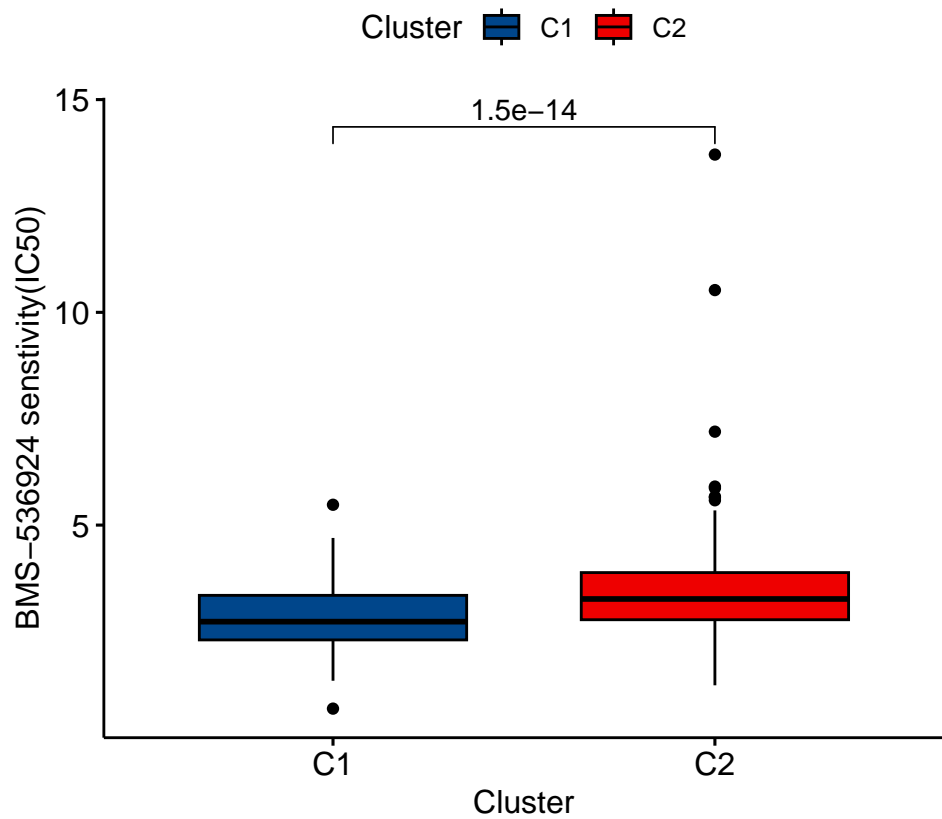

Supplement: Supplementary file 2 — Supplementary file2 (ZIP 3179 KB) [file 10238_2024_1372_MOESM2_ESM.zip › Supplementary Material/Drug2/drugSenstivity.BMS-536924.pdf]

Cluster 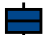 C1 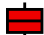 C2

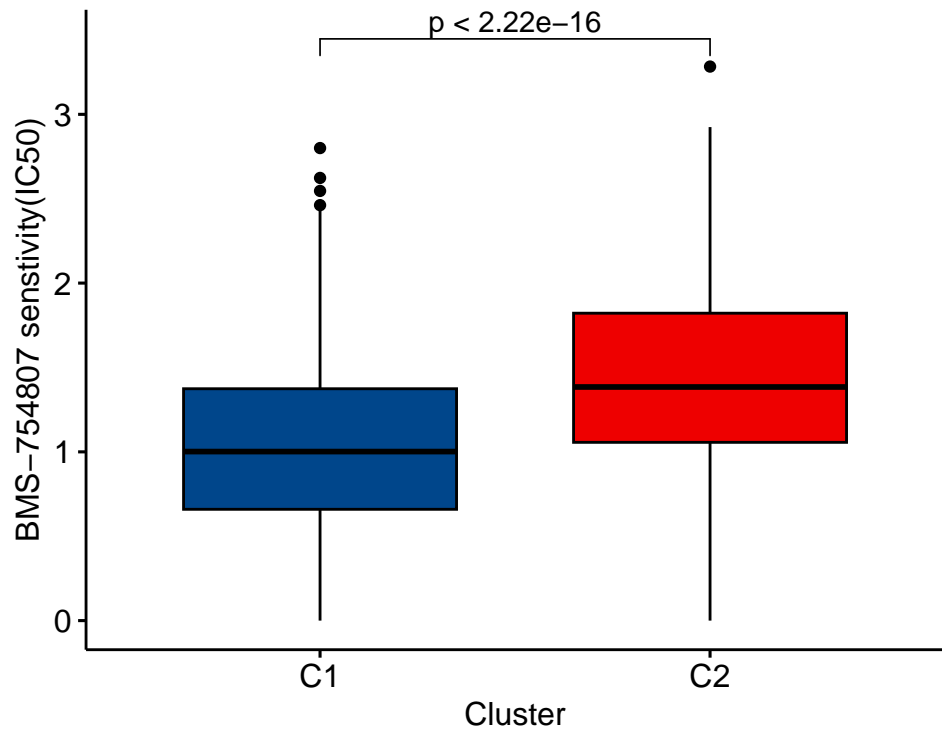

Supplement: Supplementary file 2 — Supplementary file2 (ZIP 3179 KB) [file 10238_2024_1372_MOESM2_ESM.zip › Supplementary Material/Drug2/drugSenstivity.BMS-754807.pdf]

Cluster 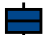 C1 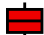 C2

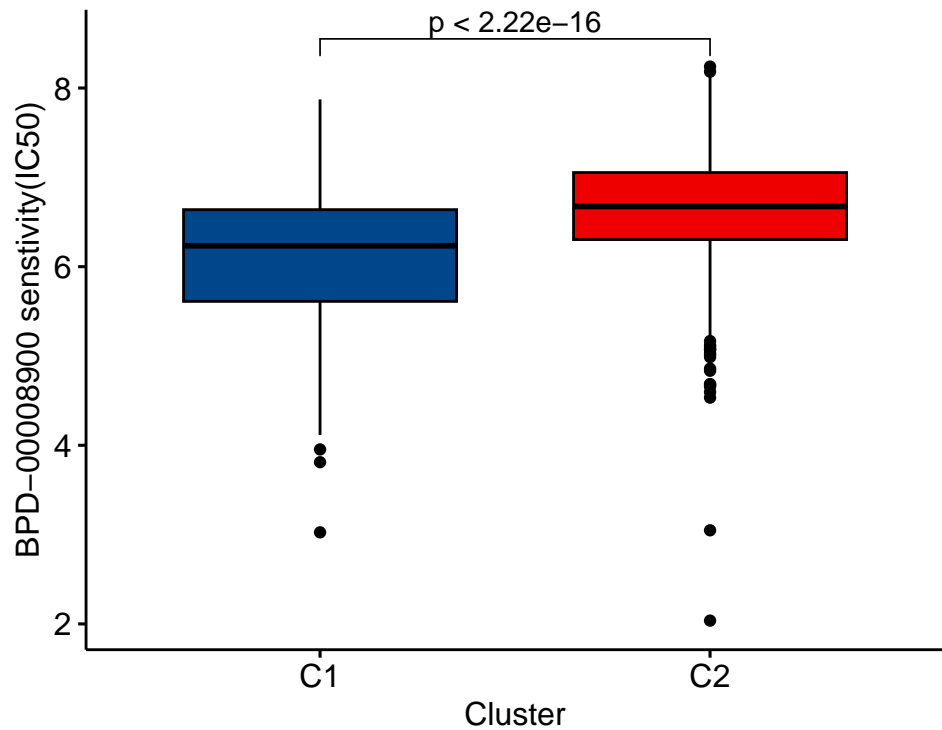

Supplement: Supplementary file 2 — Supplementary file2 (ZIP 3179 KB) [file 10238_2024_1372_MOESM2_ESM.zip › Supplementary Material/Drug2/drugSenstivity.BPD-00008900.pdf]

Cluster 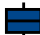 C1 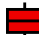 C2

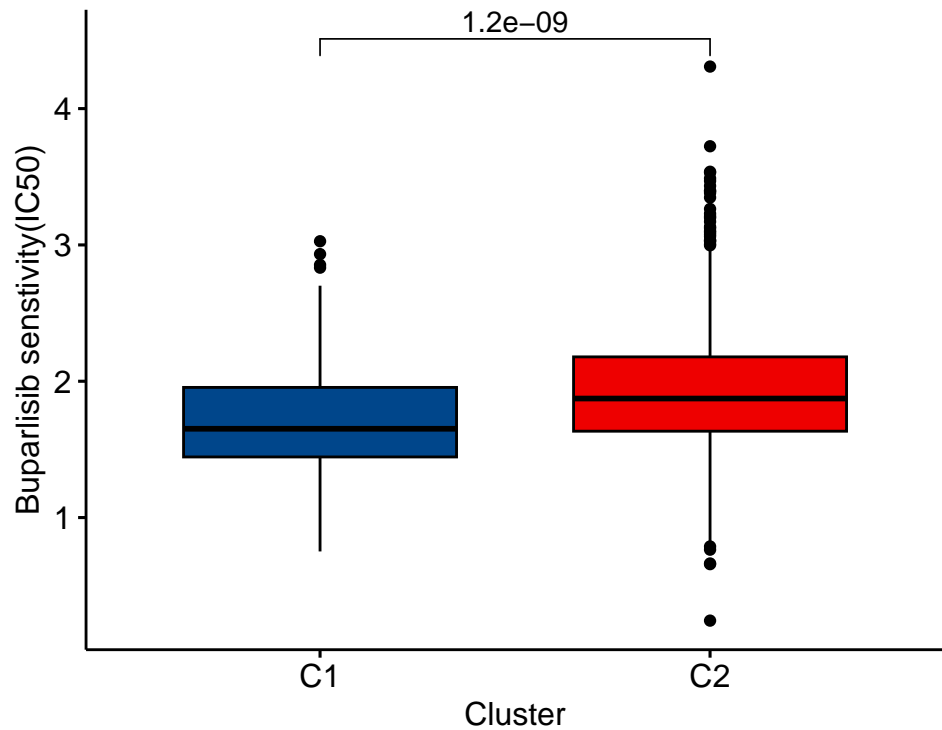

Supplement: Supplementary file 2 — Supplementary file2 (ZIP 3179 KB) [file 10238_2024_1372_MOESM2_ESM.zip › Supplementary Material/Drug2/drugSenstivity.Buparlisib.pdf]

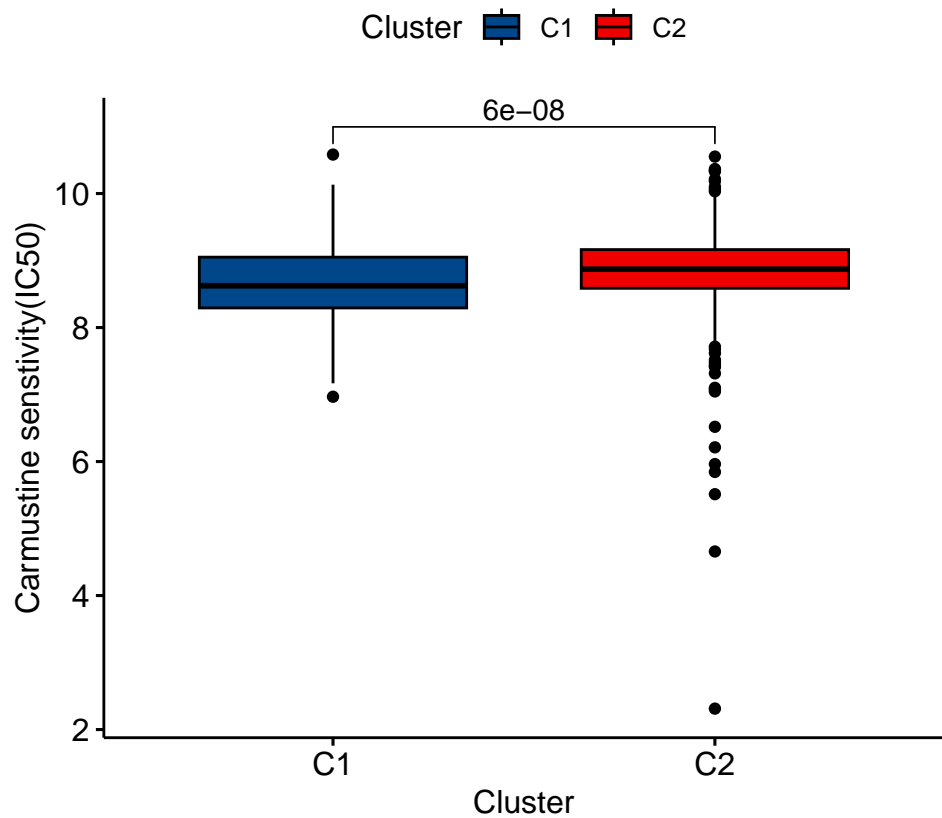

Supplement: Supplementary file 2 — Supplementary file2 (ZIP 3179 KB) [file 10238_2024_1372_MOESM2_ESM.zip › Supplementary Material/Drug2/drugSenstivity.Carmustine.pdf]

Cluster 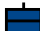 C1 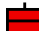 C2

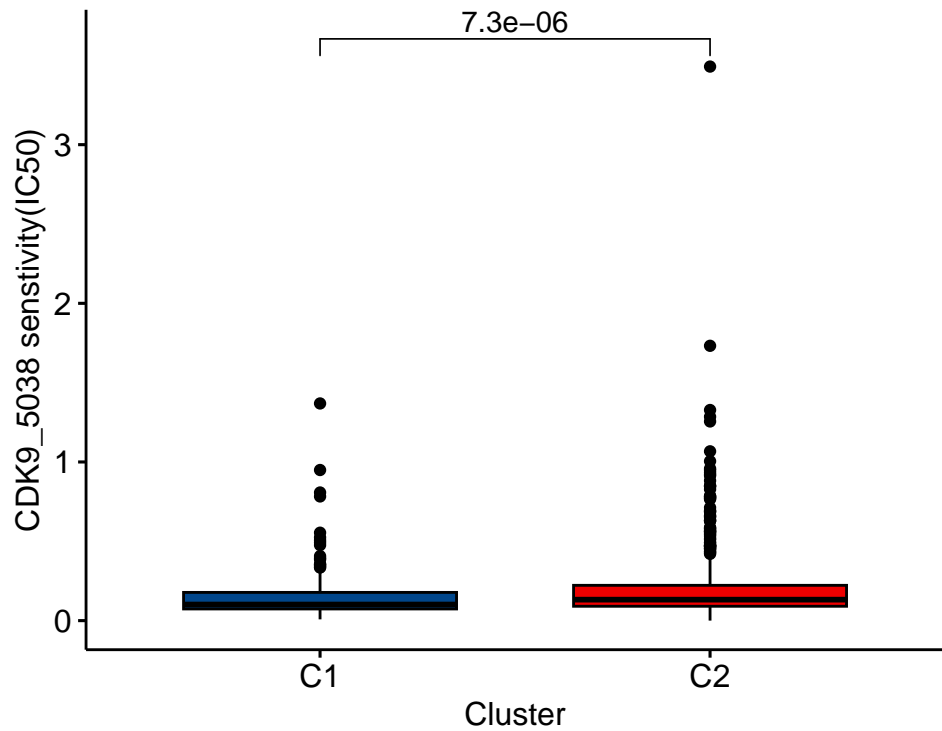

Supplement: Supplementary file 2 — Supplementary file2 (ZIP 3179 KB) [file 10238_2024_1372_MOESM2_ESM.zip › Supplementary Material/Drug2/drugSenstivity.CDK9_5038.pdf]

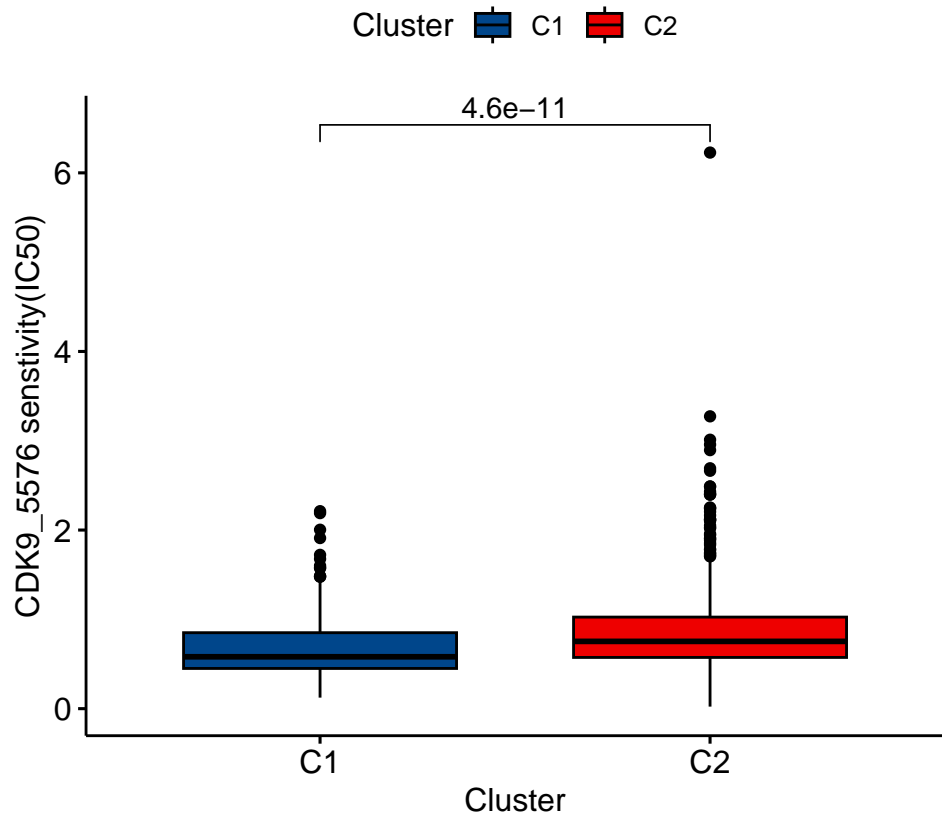

Supplement: Supplementary file 2 — Supplementary file2 (ZIP 3179 KB) [file 10238_2024_1372_MOESM2_ESM.zip › Supplementary Material/Drug2/drugSenstivity.CDK9_5576.pdf]

Cluster 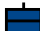 C1 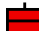 C2

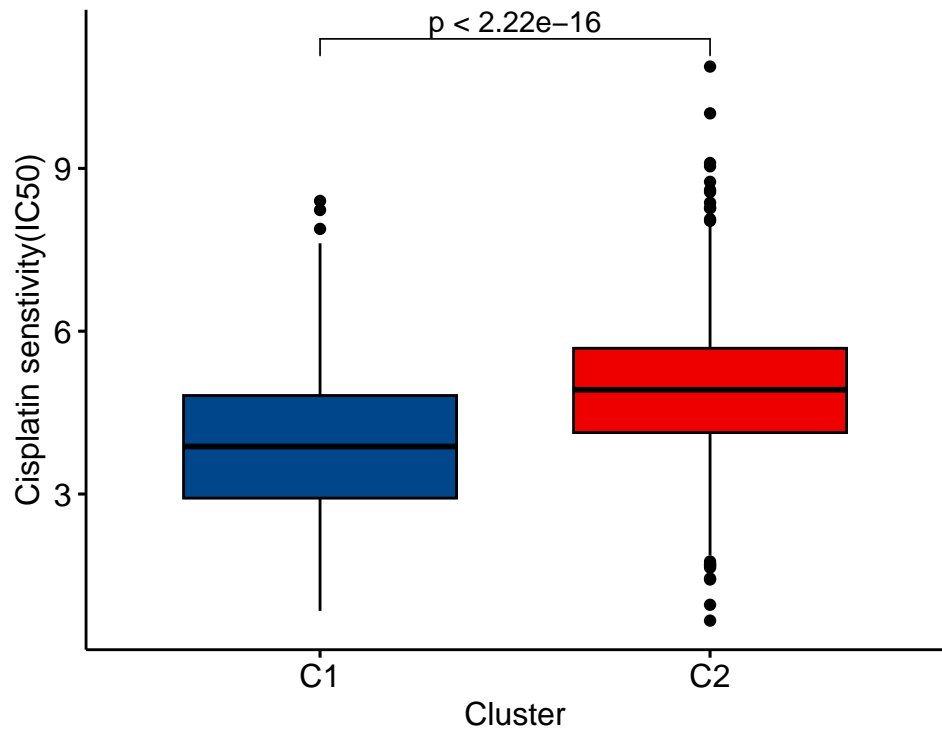

Supplement: Supplementary file 2 — Supplementary file2 (ZIP 3179 KB) [file 10238_2024_1372_MOESM2_ESM.zip › Supplementary Material/Drug2/drugSenstivity.Cisplatin.pdf]

Cluster 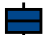 C1 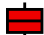 C2

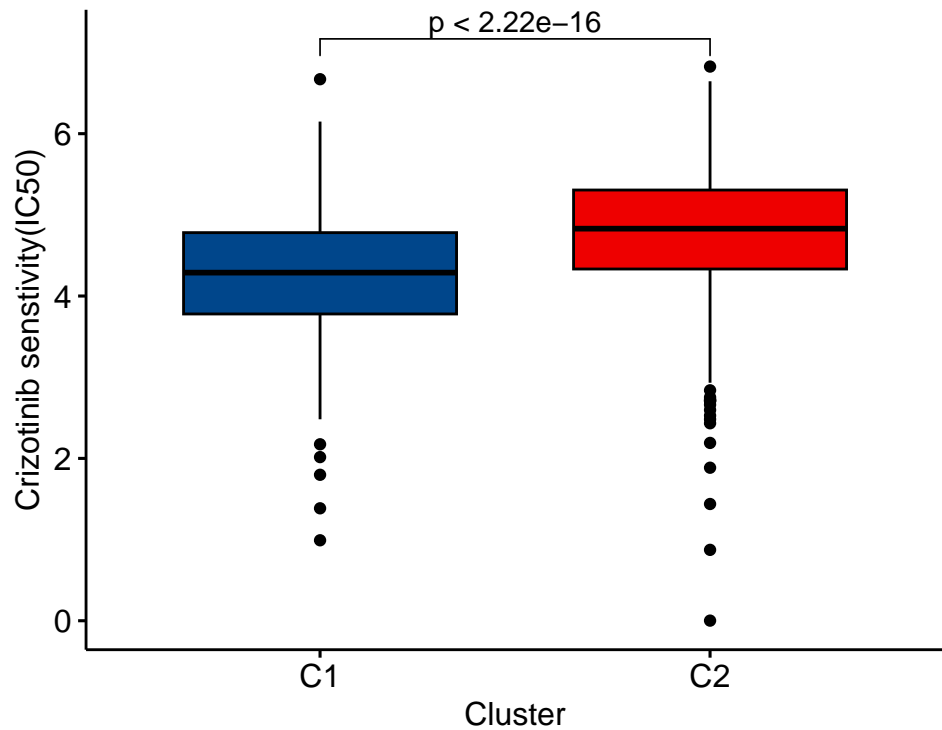

Supplement: Supplementary file 2 — Supplementary file2 (ZIP 3179 KB) [file 10238_2024_1372_MOESM2_ESM.zip › Supplementary Material/Drug2/drugSenstivity.Crizotinib.pdf]

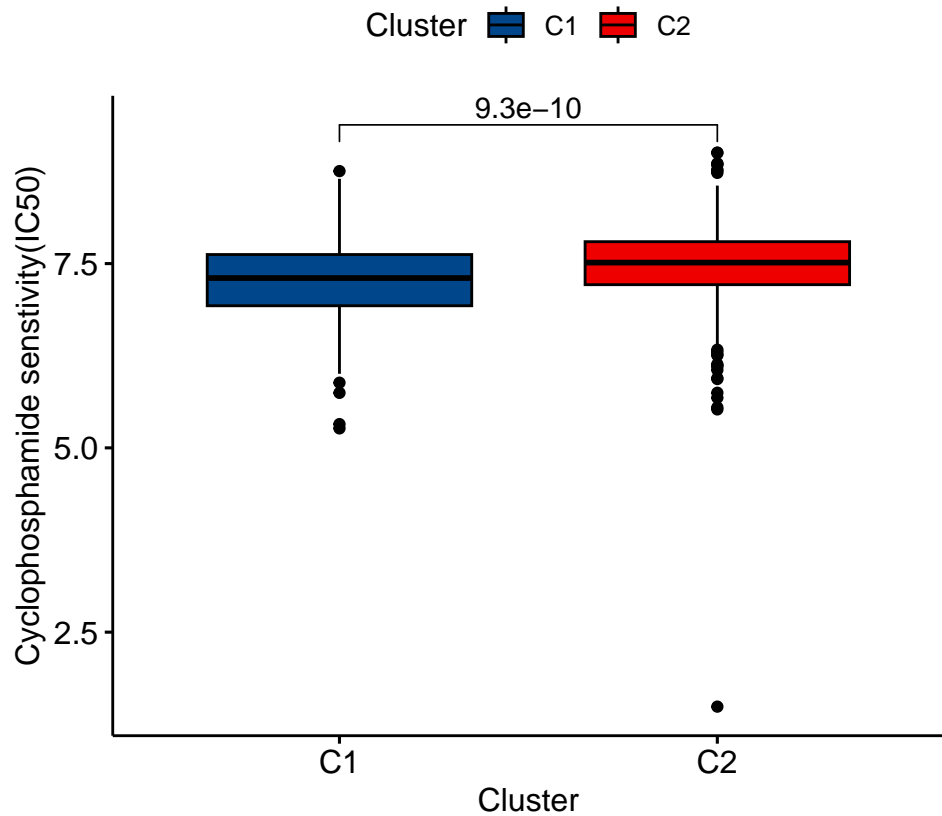

Supplement: Supplementary file 2 — Supplementary file2 (ZIP 3179 KB) [file 10238_2024_1372_MOESM2_ESM.zip › Supplementary Material/Drug2/drugSenstivity.Cyclophosphamide.pdf]

Cluster 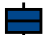 C1 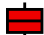 C2

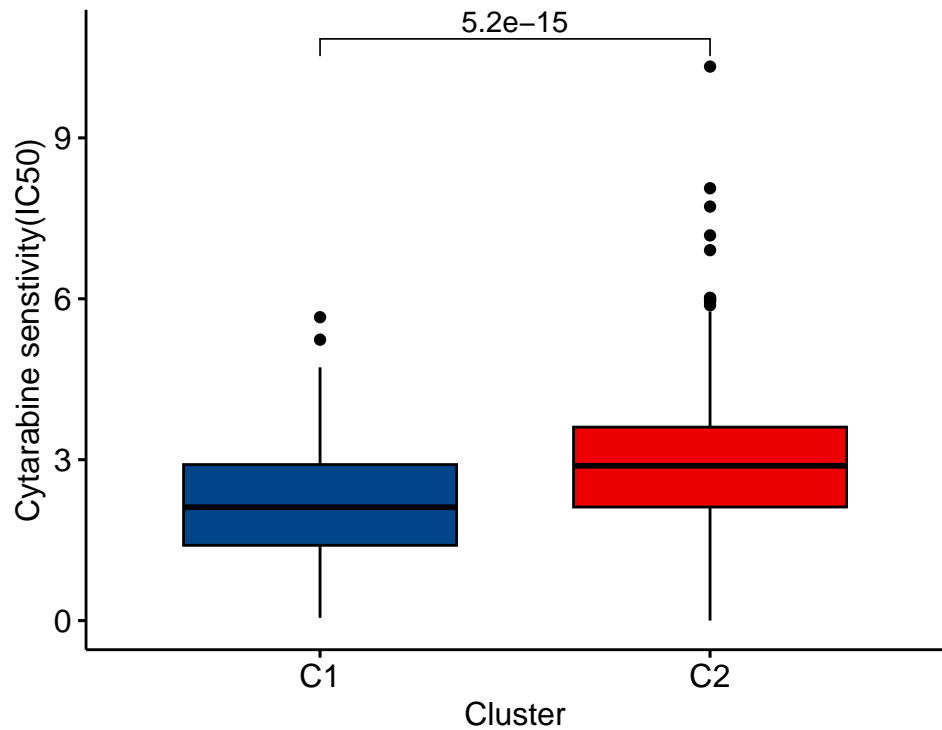

Supplement: Supplementary file 2 — Supplementary file2 (ZIP 3179 KB) [file 10238_2024_1372_MOESM2_ESM.zip › Supplementary Material/Drug2/drugSenstivity.Cytarabine.pdf]

Cluster 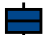 C1 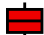 C2

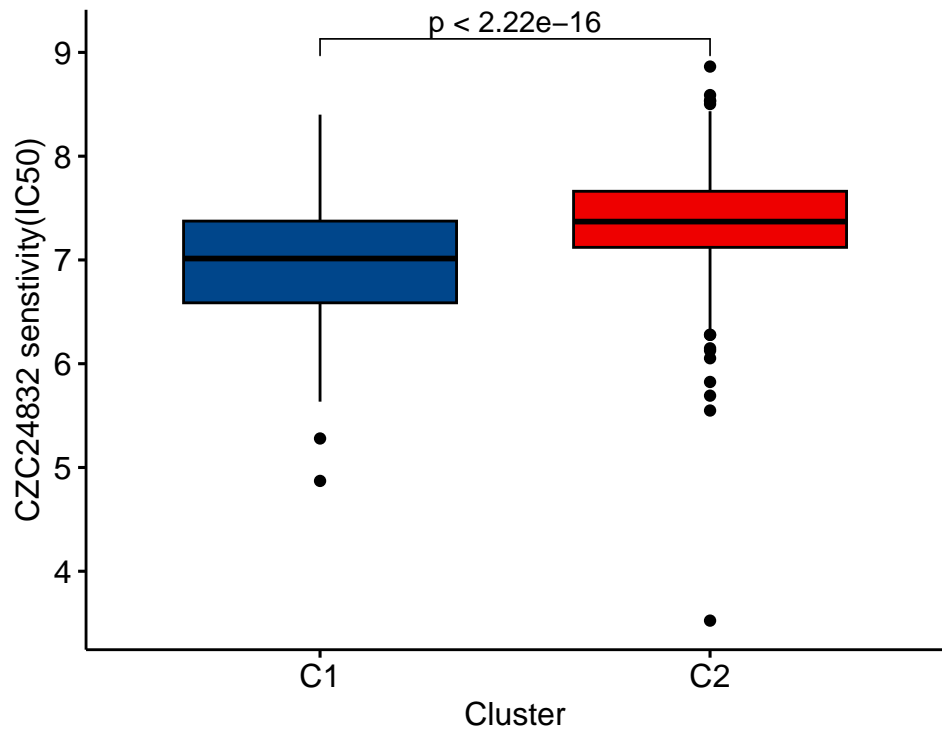

Supplement: Supplementary file 2 — Supplementary file2 (ZIP 3179 KB) [file 10238_2024_1372_MOESM2_ESM.zip › Supplementary Material/Drug2/drugSenstivity.CZC24832.pdf]

Cluster 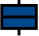 C1 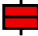 C2

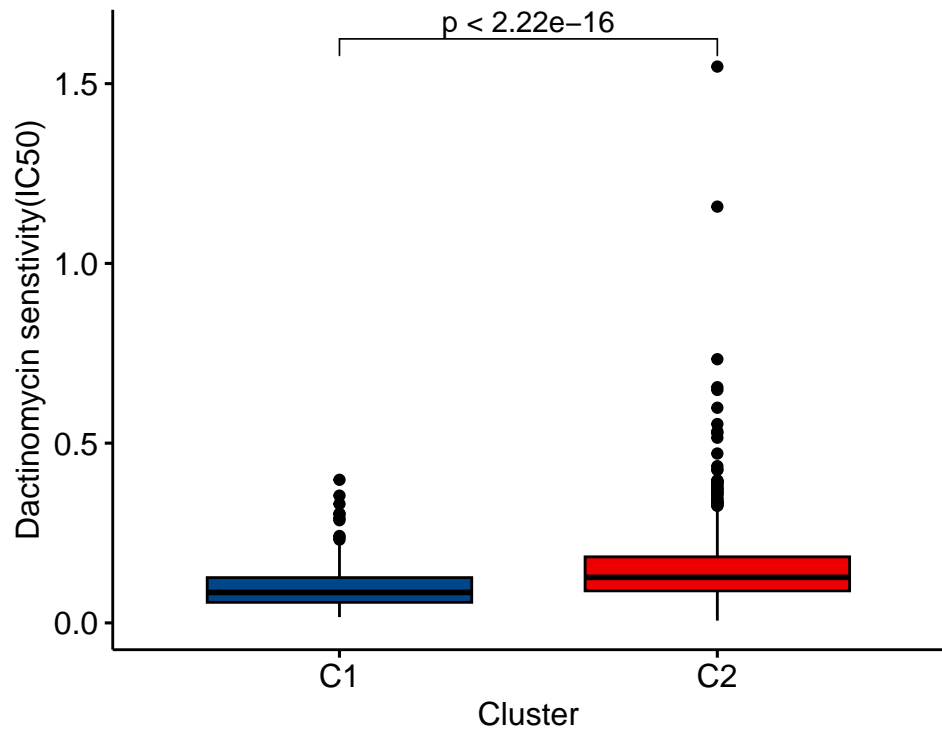

Supplement: Supplementary file 2 — Supplementary file2 (ZIP 3179 KB) [file 10238_2024_1372_MOESM2_ESM.zip › Supplementary Material/Drug2/drugSenstivity.Dactinomycin.pdf]

Cluster C1 C2

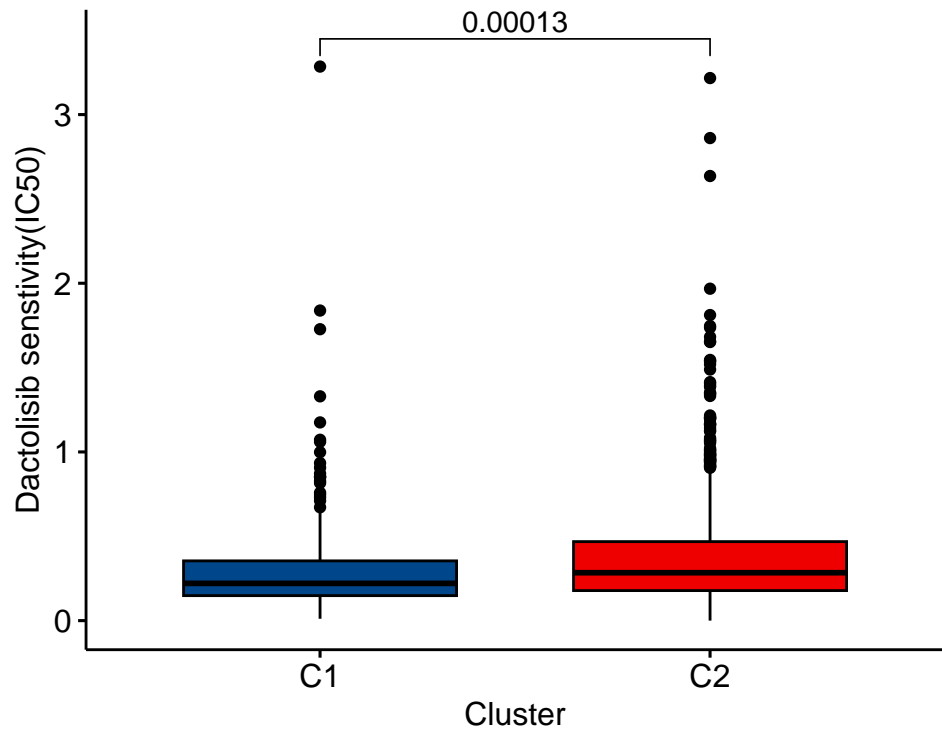

Supplement: Supplementary file 2 — Supplementary file2 (ZIP 3179 KB) [file 10238_2024_1372_MOESM2_ESM.zip › Supplementary Material/Drug2/drugSenstivity.Dactolisib.pdf]

Dasatinib sensitivity(IC50)

Cluster

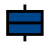

C1

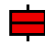

C2

3.6e-13

10.0

7.5

5.0

2.5

0.0

C1

C2

Cluster

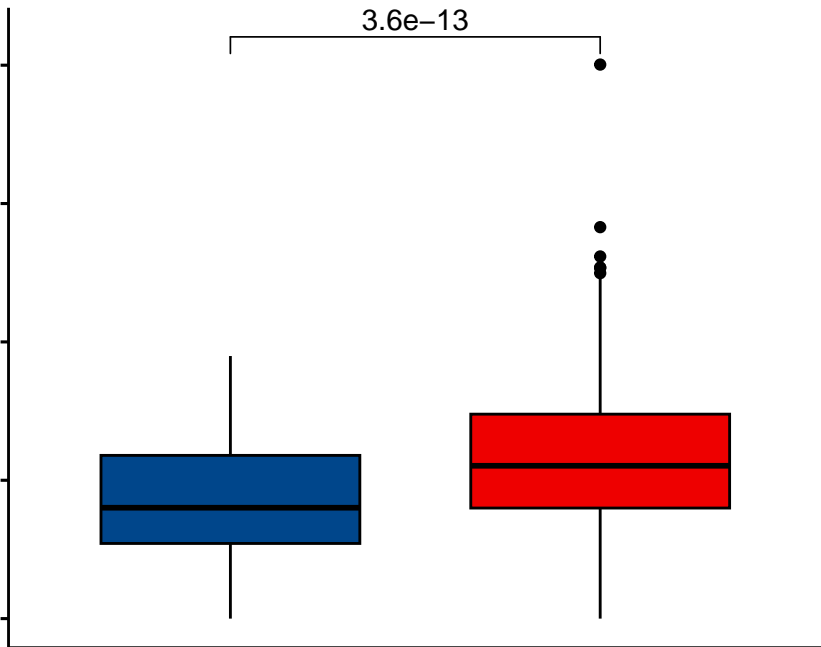

Supplement: Supplementary file 2 — Supplementary file2 (ZIP 3179 KB) [file 10238_2024_1372_MOESM2_ESM.zip › Supplementary Material/Drug2/drugSenstivity.Dasatinib.pdf]

Cluster 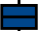 C1 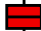 C2

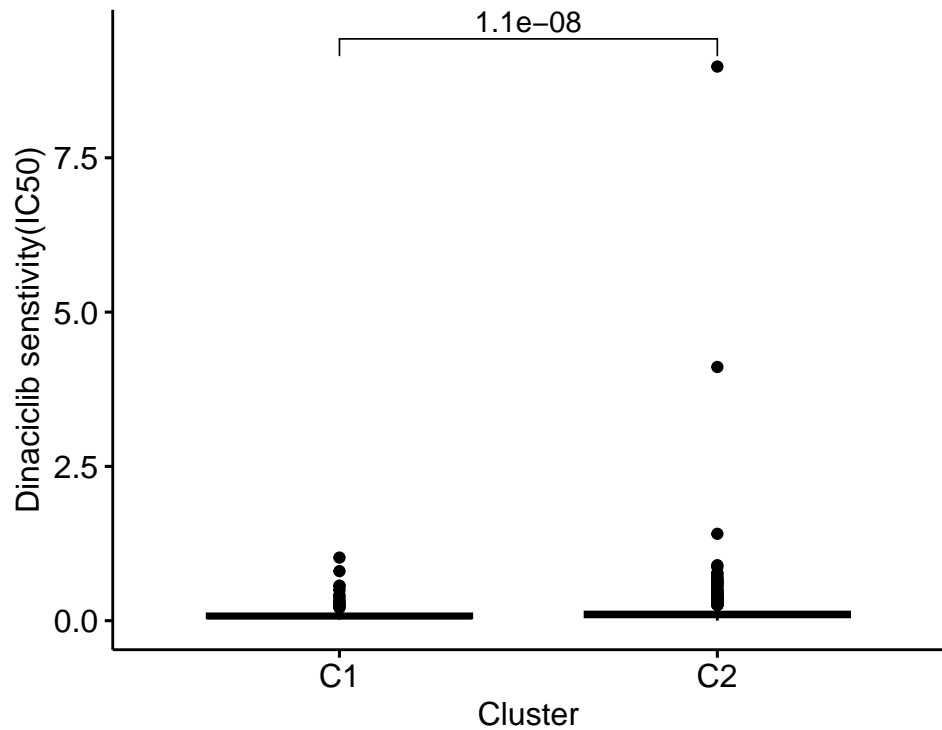

Supplement: Supplementary file 2 — Supplementary file2 (ZIP 3179 KB) [file 10238_2024_1372_MOESM2_ESM.zip › Supplementary Material/Drug2/drugSenstivity.Dinaciclib.pdf]

Cluster C1 C2

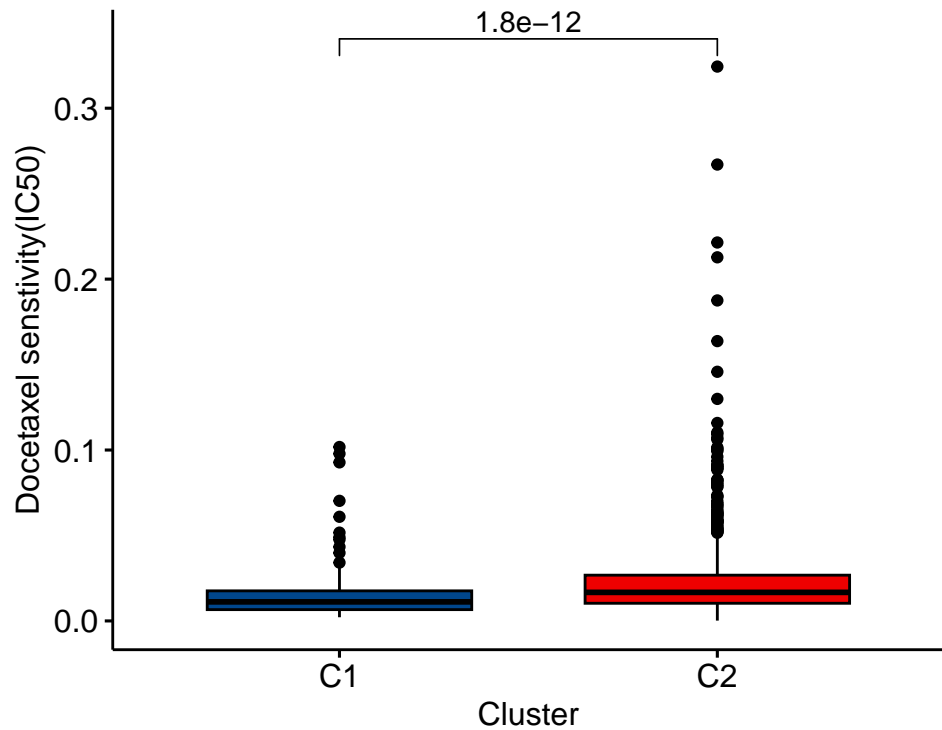

Supplement: Supplementary file 2 — Supplementary file2 (ZIP 3179 KB) [file 10238_2024_1372_MOESM2_ESM.zip › Supplementary Material/Drug2/drugSenstivity.Docetaxel.pdf]

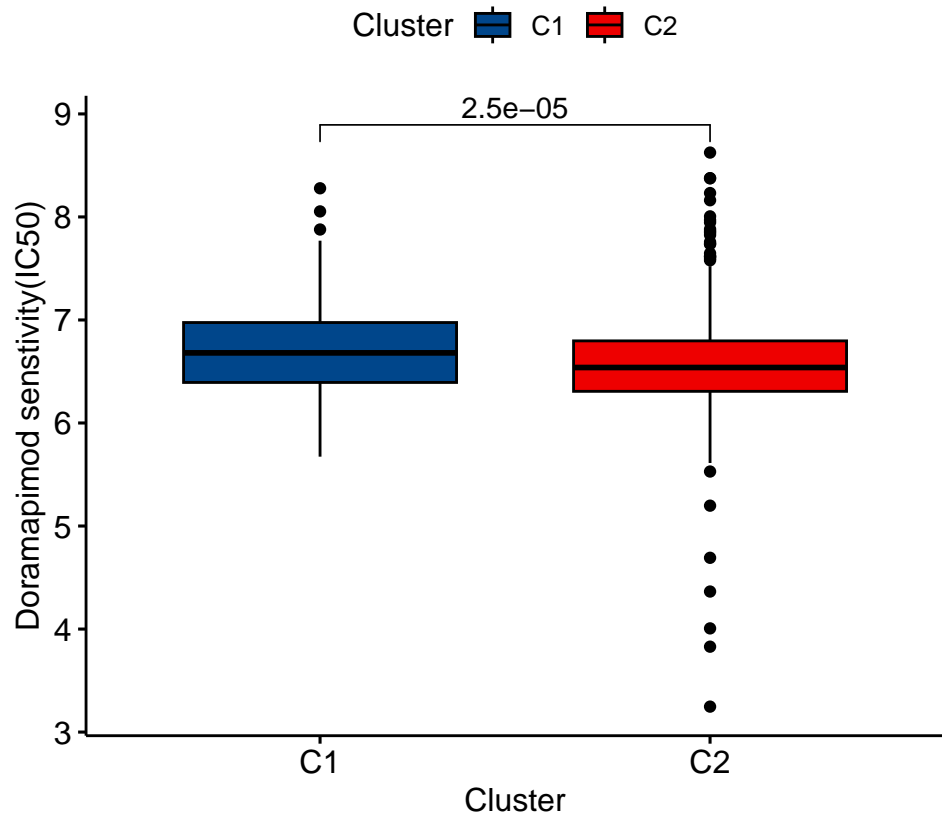

Supplement: Supplementary file 2 — Supplementary file2 (ZIP 3179 KB) [file 10238_2024_1372_MOESM2_ESM.zip › Supplementary Material/Drug2/drugSenstivity.Doramapimod.pdf]

Cluster C1 C2

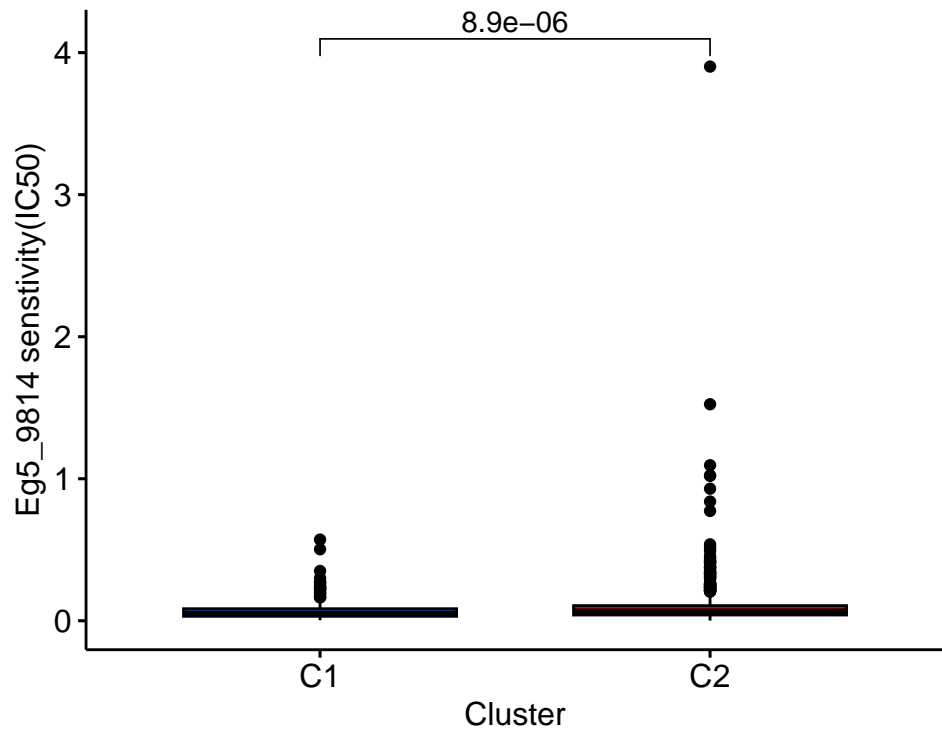

Supplement: Supplementary file 2 — Supplementary file2 (ZIP 3179 KB) [file 10238_2024_1372_MOESM2_ESM.zip › Supplementary Material/Drug2/drugSenstivity.Eg5_9814.pdf]

Cluster 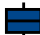 C1 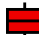 C2

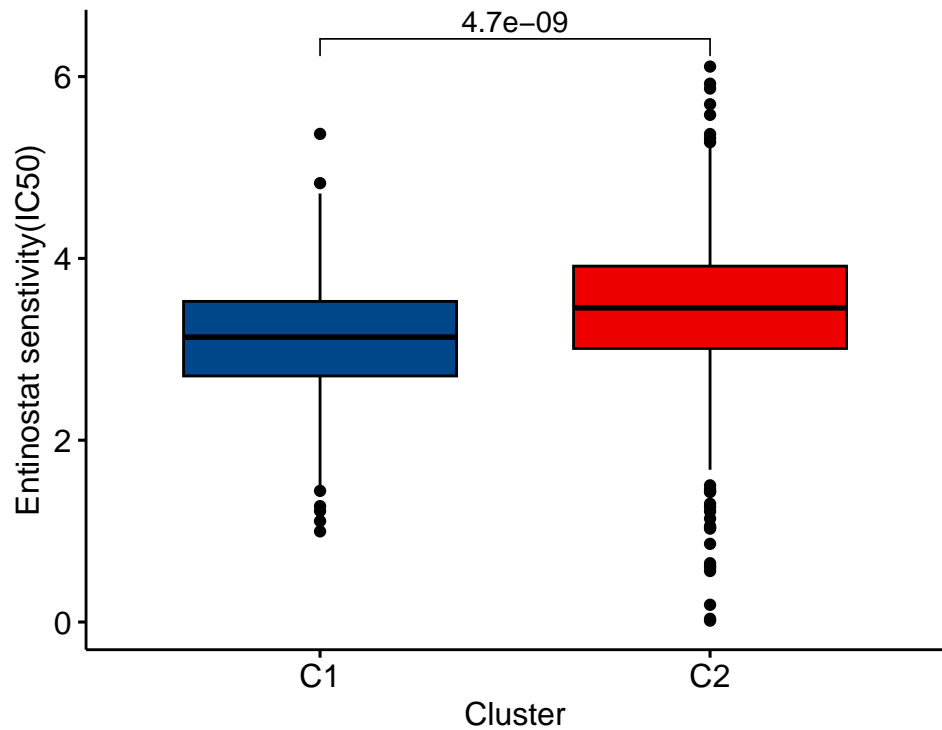

Supplement: Supplementary file 2 — Supplementary file2 (ZIP 3179 KB) [file 10238_2024_1372_MOESM2_ESM.zip › Supplementary Material/Drug2/drugSenstivity.Entinostat.pdf]

Cluster 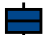 C1 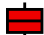 C2

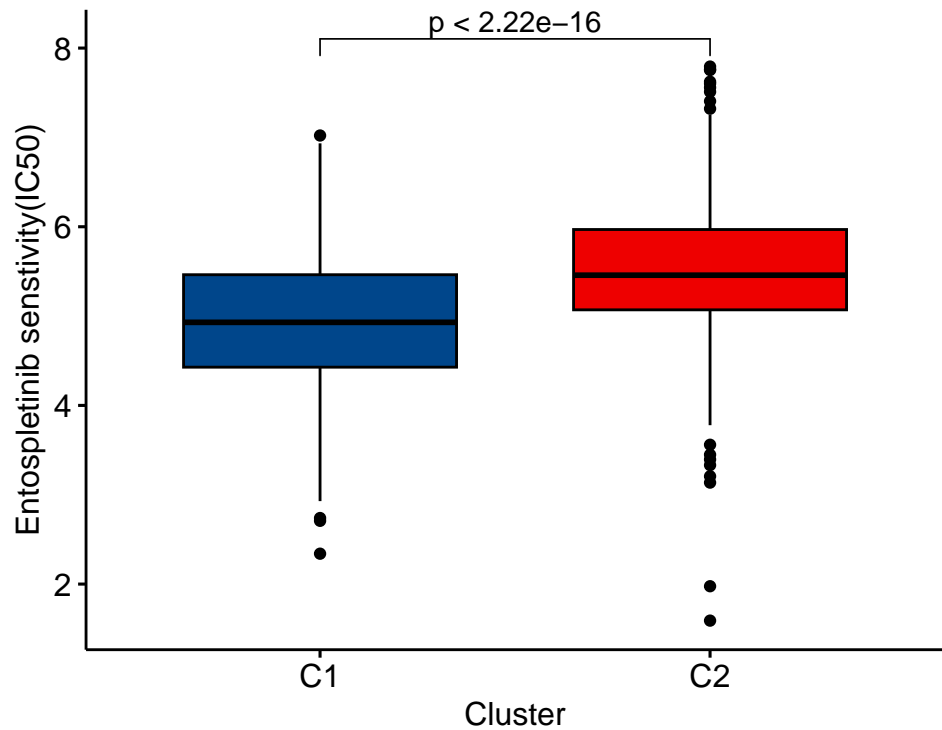

Supplement: Supplementary file 2 — Supplementary file2 (ZIP 3179 KB) [file 10238_2024_1372_MOESM2_ESM.zip › Supplementary Material/Drug2/drugSenstivity.Entospletinib.pdf]

EPZ004777 sensitivity(IC50)

Cluster

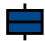

C1

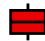

C2

5.1e-07

C1

C2

Cluster

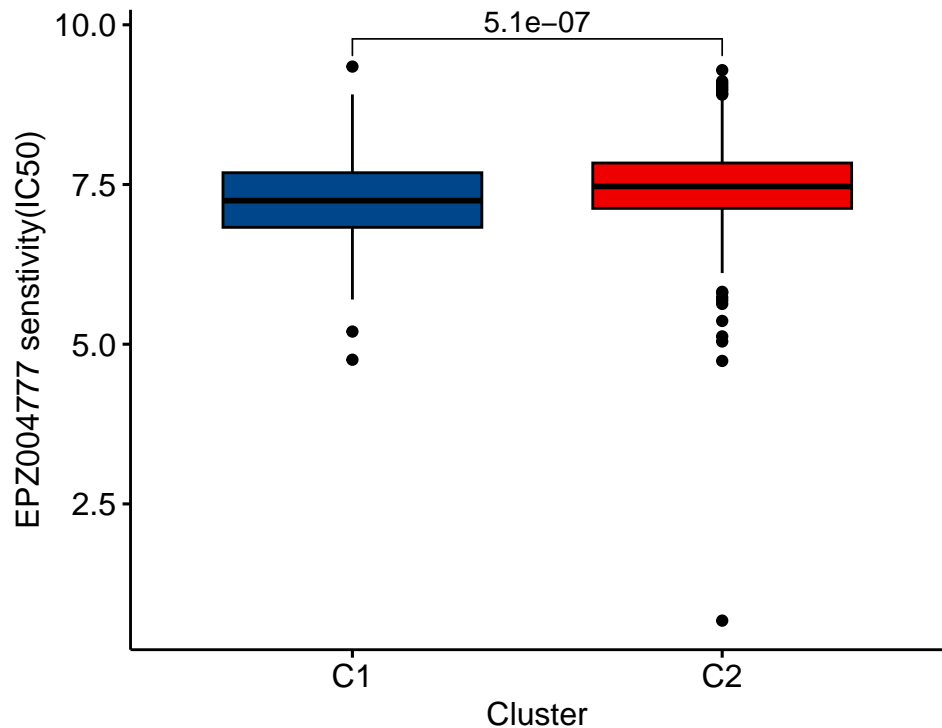

Supplement: Supplementary file 2 — Supplementary file2 (ZIP 3179 KB) [file 10238_2024_1372_MOESM2_ESM.zip › Supplementary Material/Drug2/drugSenstivity.EPZ004777.pdf]

EPZ5676 sensitivity(IC50)

Cluster

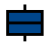

C1

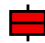

C2

$5.7e-10$

C1

C2

Cluster

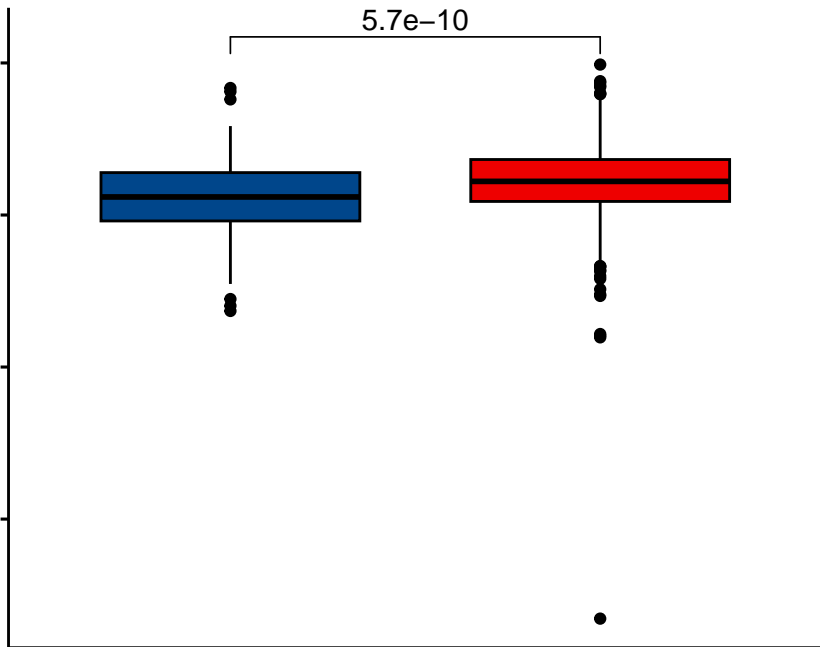

Supplement: Supplementary file 2 — Supplementary file2 (ZIP 3179 KB) [file 10238_2024_1372_MOESM2_ESM.zip › Supplementary Material/Drug2/drugSenstivity.EPZ5676.pdf]

Cluster 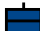 C1 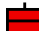 C2

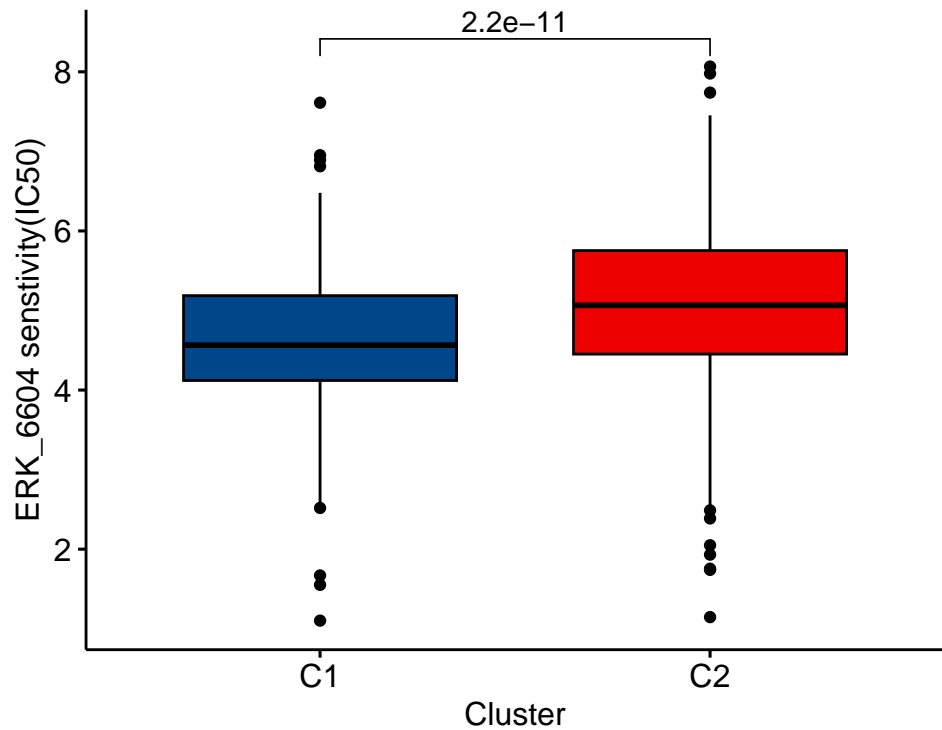

Supplement: Supplementary file 2 — Supplementary file2 (ZIP 3179 KB) [file 10238_2024_1372_MOESM2_ESM.zip › Supplementary Material/Drug2/drugSenstivity.ERK_6604.pdf]

Cluster C1 C2

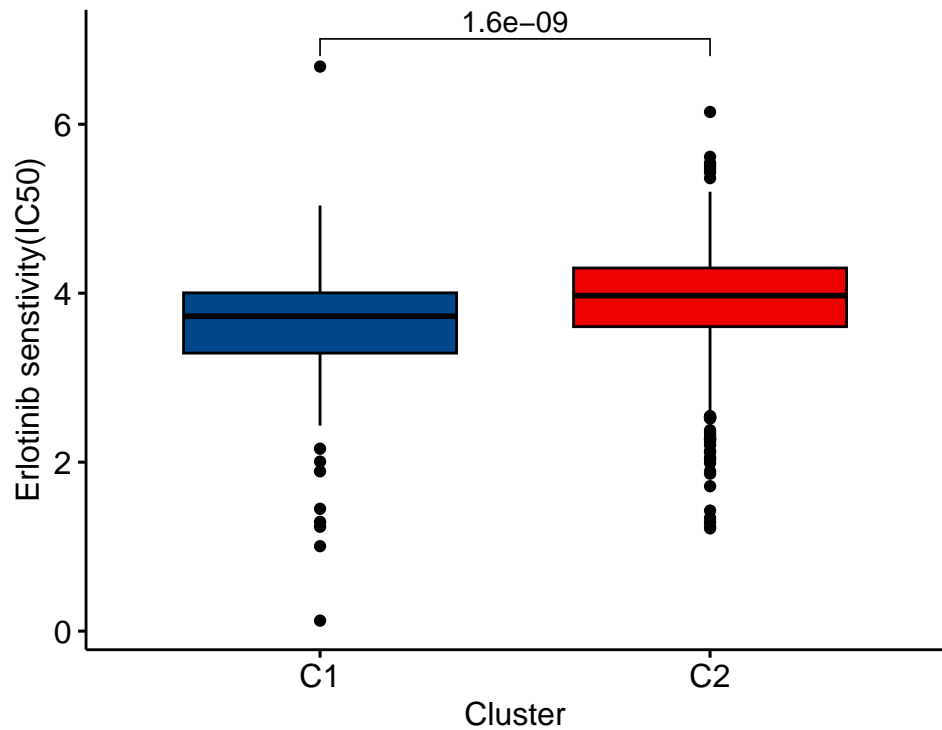

Supplement: Supplementary file 2 — Supplementary file2 (ZIP 3179 KB) [file 10238_2024_1372_MOESM2_ESM.zip › Supplementary Material/Drug2/drugSenstivity.Erlotinib.pdf]

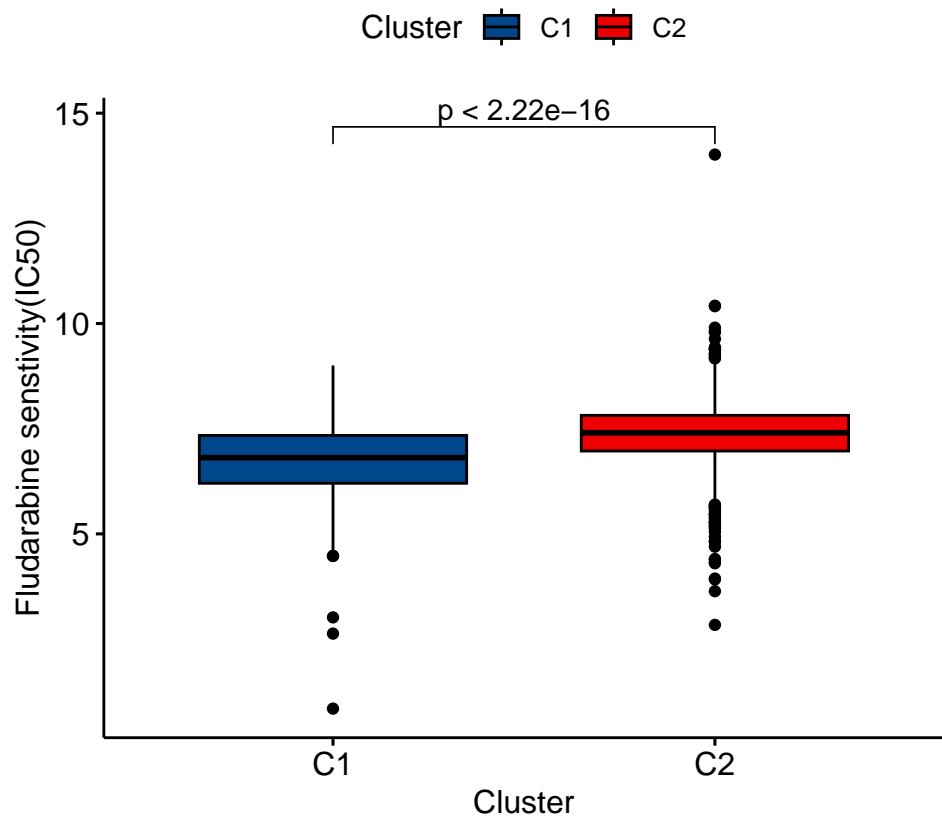

Supplement: Supplementary file 2 — Supplementary file2 (ZIP 3179 KB) [file 10238_2024_1372_MOESM2_ESM.zip › Supplementary Material/Drug2/drugSenstivity.Fludarabine.pdf]

Cluster 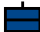 C1 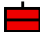 C2

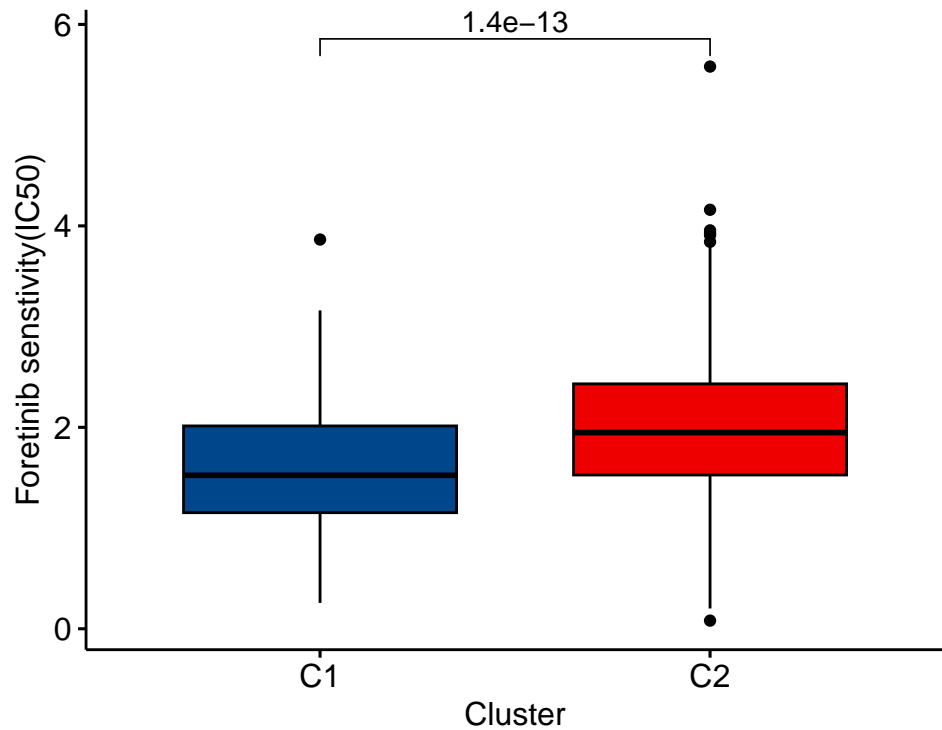

Supplement: Supplementary file 2 — Supplementary file2 (ZIP 3179 KB) [file 10238_2024_1372_MOESM2_ESM.zip › Supplementary Material/Drug2/drugSenstivity.Foretinib.pdf]

Cluster

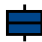

C1

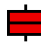

C2

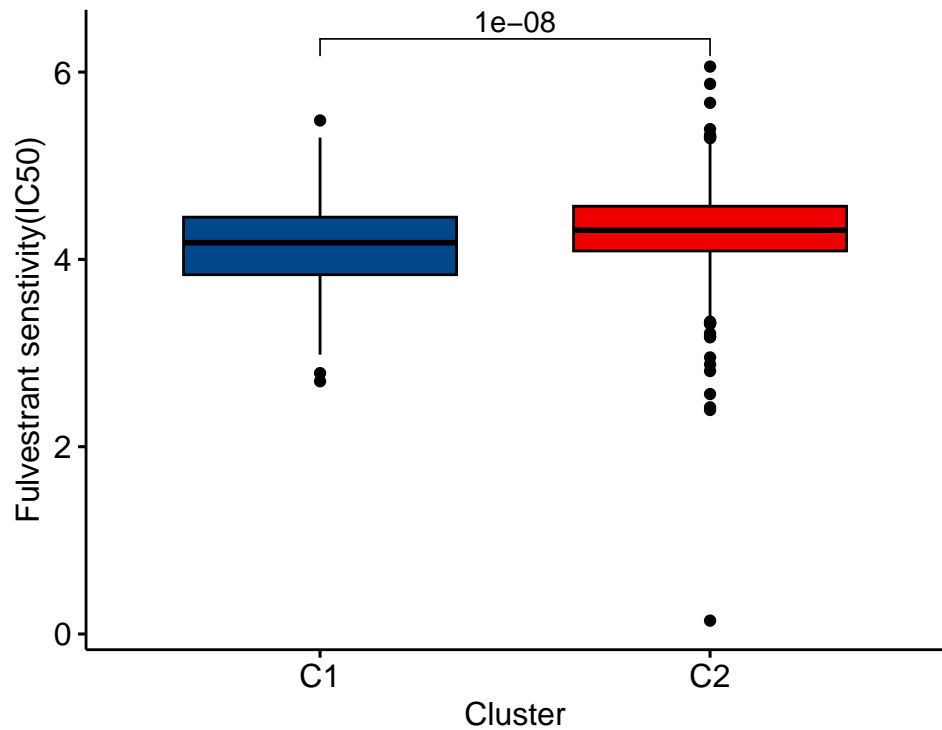

Supplement: Supplementary file 2 — Supplementary file2 (ZIP 3179 KB) [file 10238_2024_1372_MOESM2_ESM.zip › Supplementary Material/Drug2/drugSenstivity.Fulvestrant.pdf]

Cluster 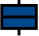 C1 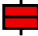 C2

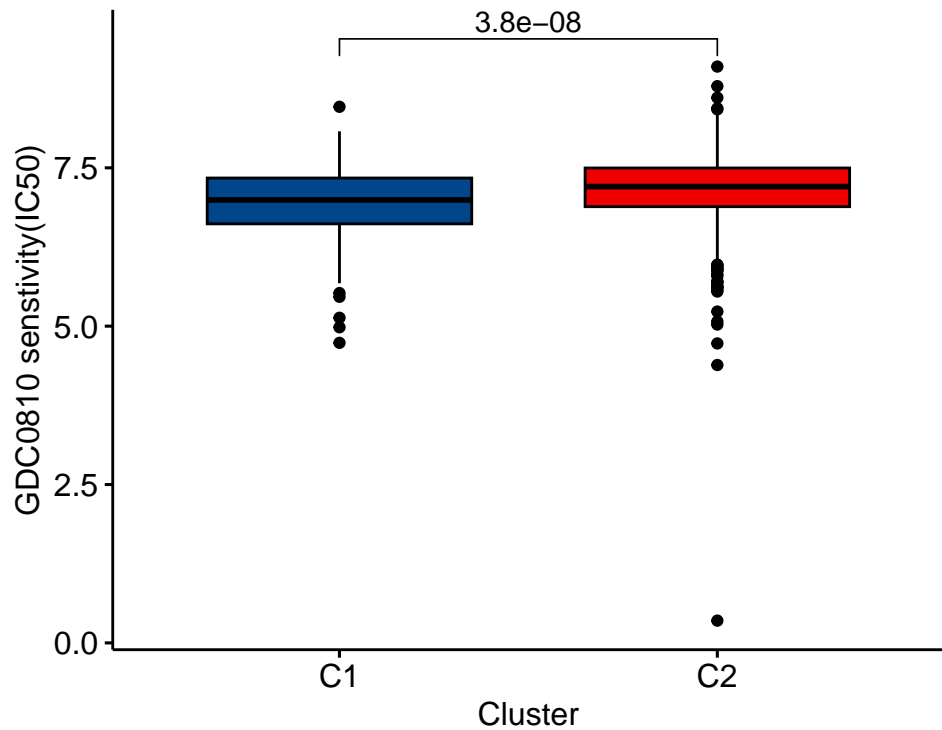

Supplement: Supplementary file 2 — Supplementary file2 (ZIP 3179 KB) [file 10238_2024_1372_MOESM2_ESM.zip › Supplementary Material/Drug2/drugSenstivity.GDC0810.pdf]

Cluster C1 C2

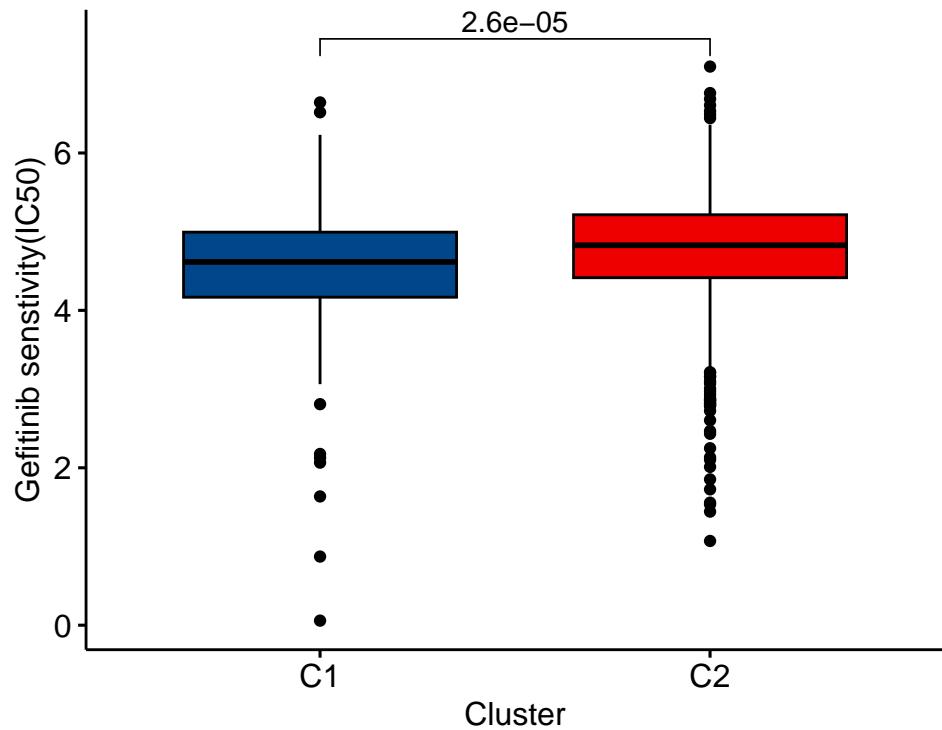

Supplement: Supplementary file 2 — Supplementary file2 (ZIP 3179 KB) [file 10238_2024_1372_MOESM2_ESM.zip › Supplementary Material/Drug2/drugSenstivity.Gefitinib.pdf]

Cluster 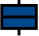 C1 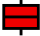 C2

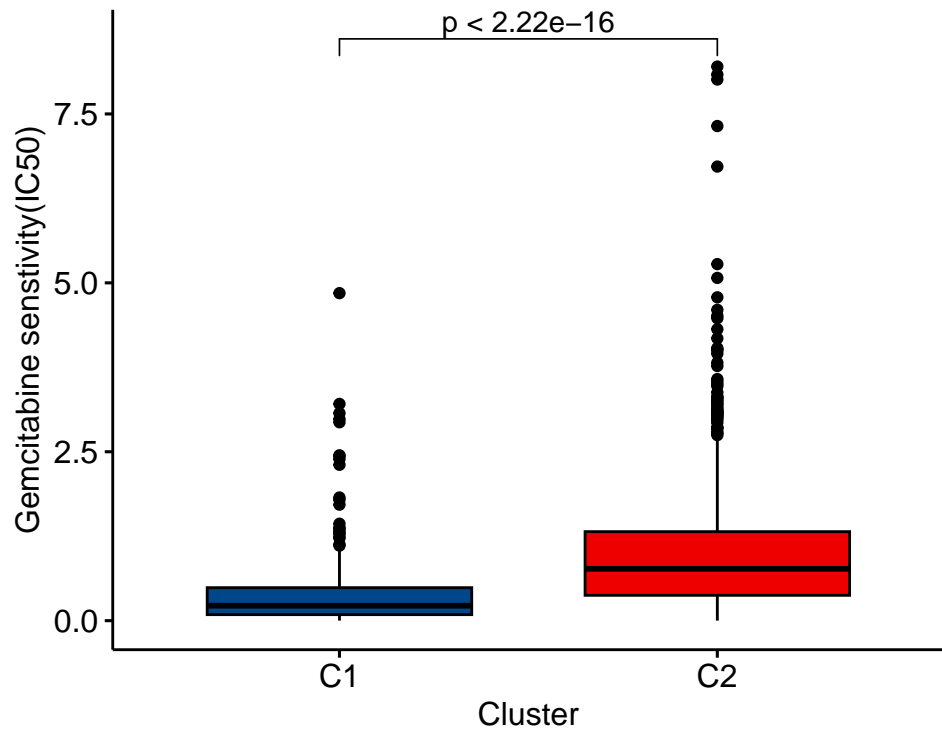

Supplement: Supplementary file 2 — Supplementary file2 (ZIP 3179 KB) [file 10238_2024_1372_MOESM2_ESM.zip › Supplementary Material/Drug2/drugSenstivity.Gemcitabine.pdf]

Cluster 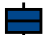 C1 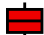 C2

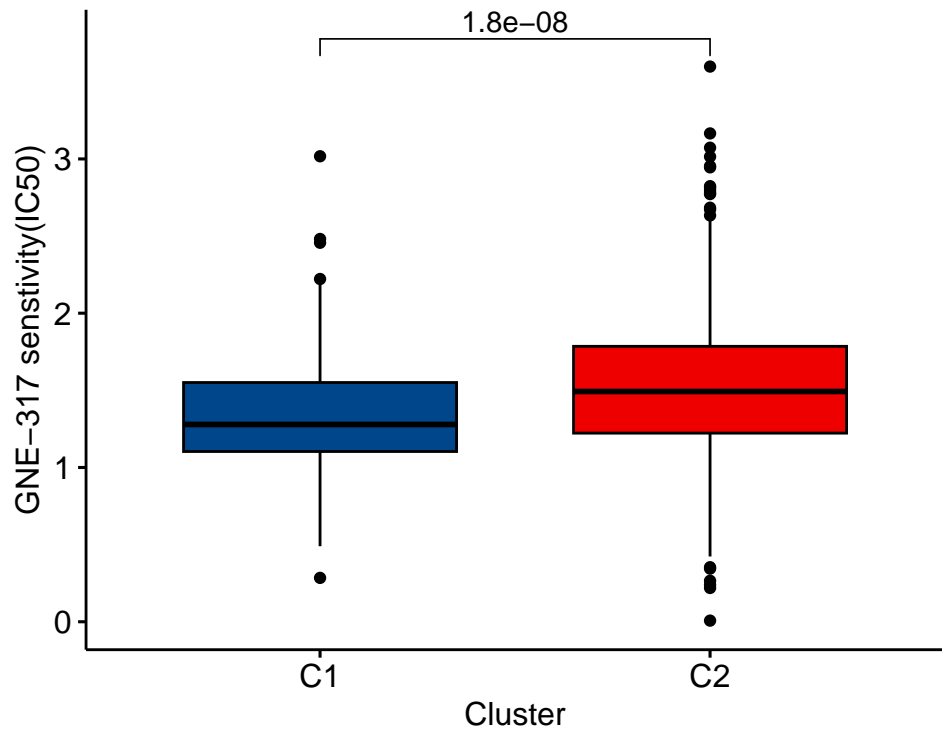

Supplement: Supplementary file 2 — Supplementary file2 (ZIP 3179 KB) [file 10238_2024_1372_MOESM2_ESM.zip › Supplementary Material/Drug2/drugSenstivity.GNE-317.pdf]

Cluster 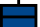 C1 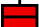 C2

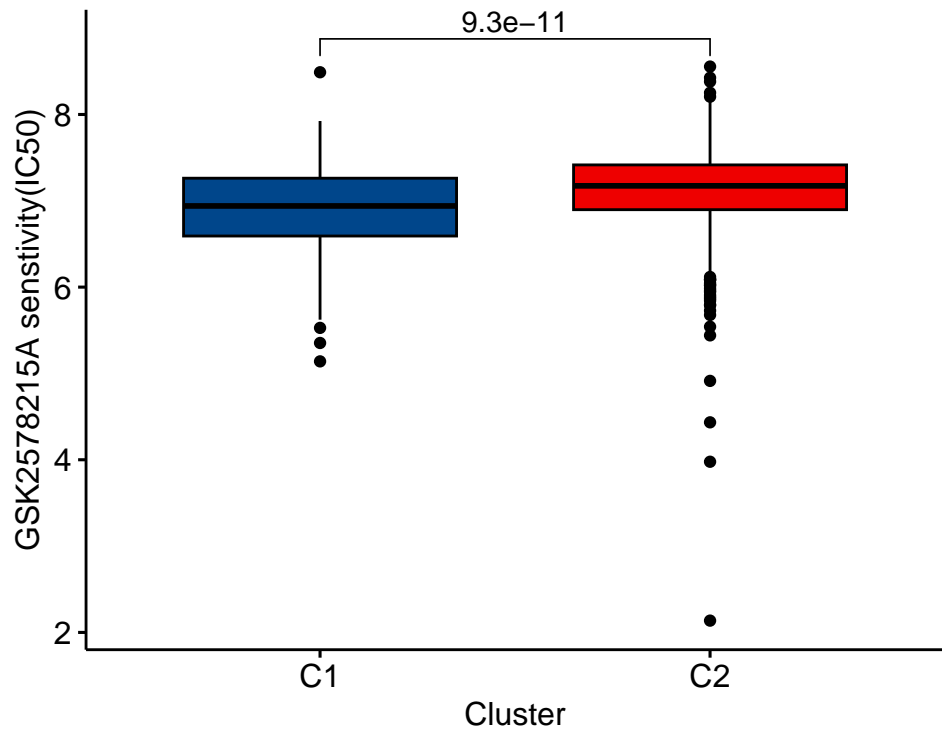

Supplement: Supplementary file 2 — Supplementary file2 (ZIP 3179 KB) [file 10238_2024_1372_MOESM2_ESM.zip › Supplementary Material/Drug2/drugSenstivity.GSK2578215A.pdf]

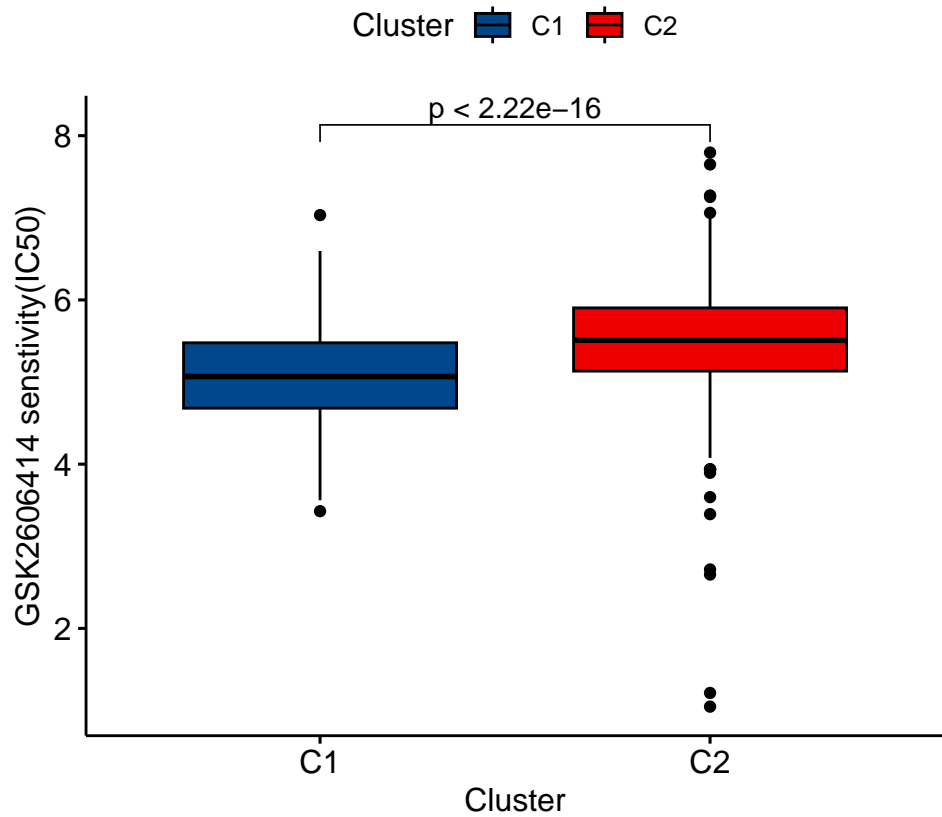

Supplement: Supplementary file 2 — Supplementary file2 (ZIP 3179 KB) [file 10238_2024_1372_MOESM2_ESM.zip › Supplementary Material/Drug2/drugSenstivity.GSK2606414.pdf]

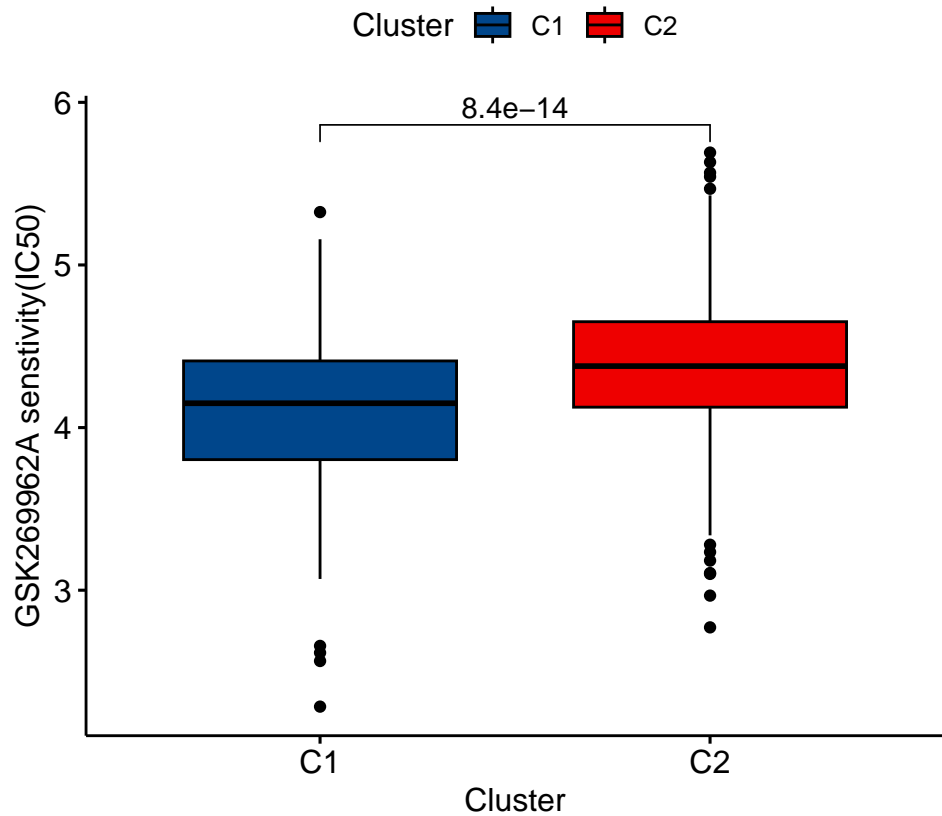

Supplement: Supplementary file 2 — Supplementary file2 (ZIP 3179 KB) [file 10238_2024_1372_MOESM2_ESM.zip › Supplementary Material/Drug2/drugSenstivity.GSK269962A.pdf]

GSK343 sensitivity(IC50)

Cluster

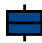

C1

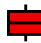

C2

$p < 2.22e-16$

C1

C2

Cluster

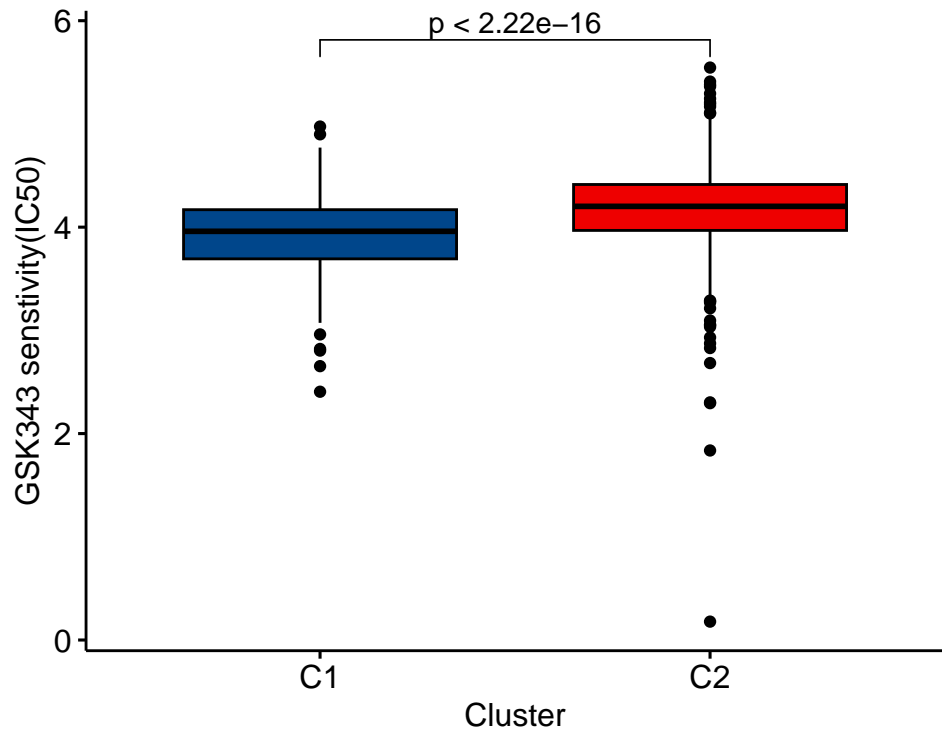

Supplement: Supplementary file 2 — Supplementary file2 (ZIP 3179 KB) [file 10238_2024_1372_MOESM2_ESM.zip › Supplementary Material/Drug2/drugSenstivity.GSK343.pdf]

Cluster 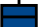 C1 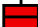 C2

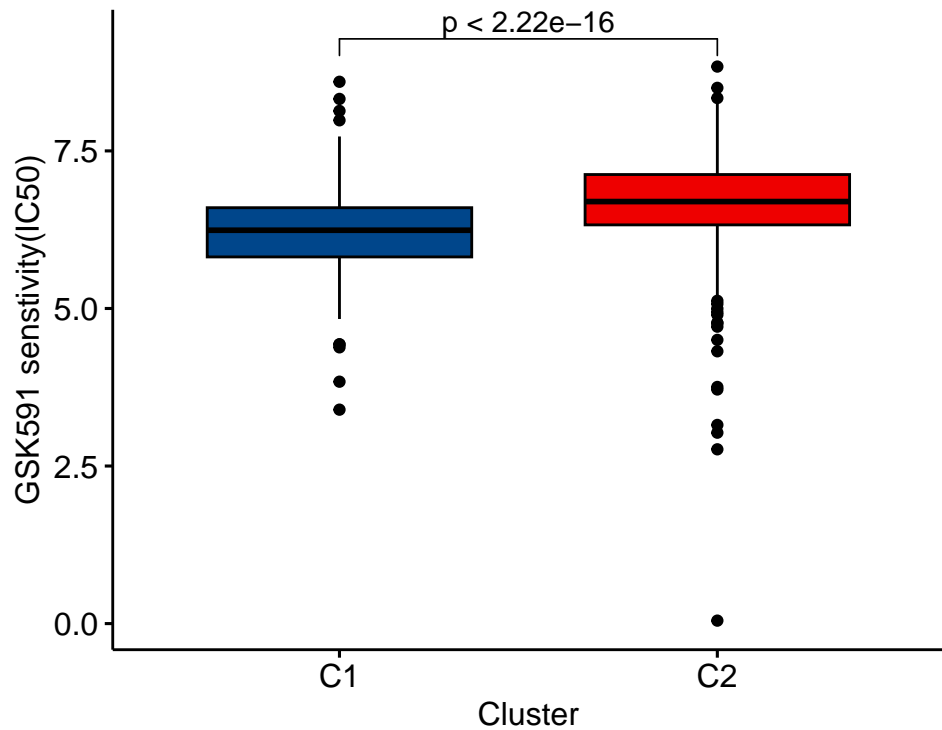

Supplement: Supplementary file 2 — Supplementary file2 (ZIP 3179 KB) [file 10238_2024_1372_MOESM2_ESM.zip › Supplementary Material/Drug2/drugSenstivity.GSK591.pdf]

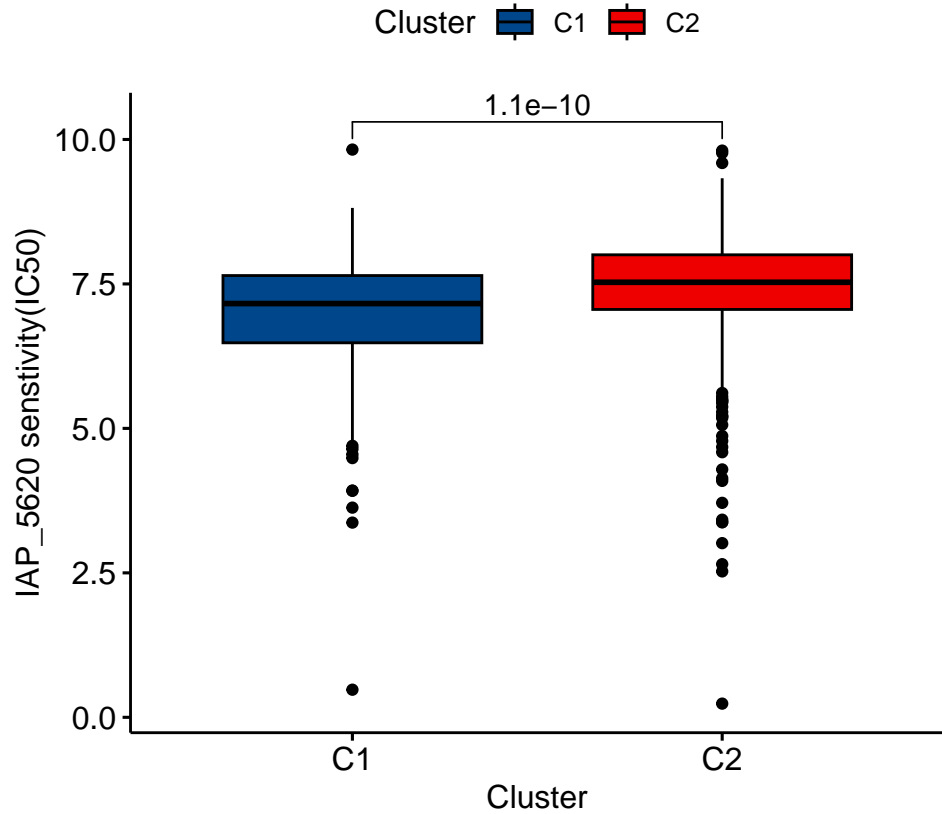

Supplement: Supplementary file 2 — Supplementary file2 (ZIP 3179 KB) [file 10238_2024_1372_MOESM2_ESM.zip › Supplementary Material/Drug2/drugSenstivity.IAP_5620.pdf]

Cluster 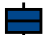 C1 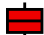 C2

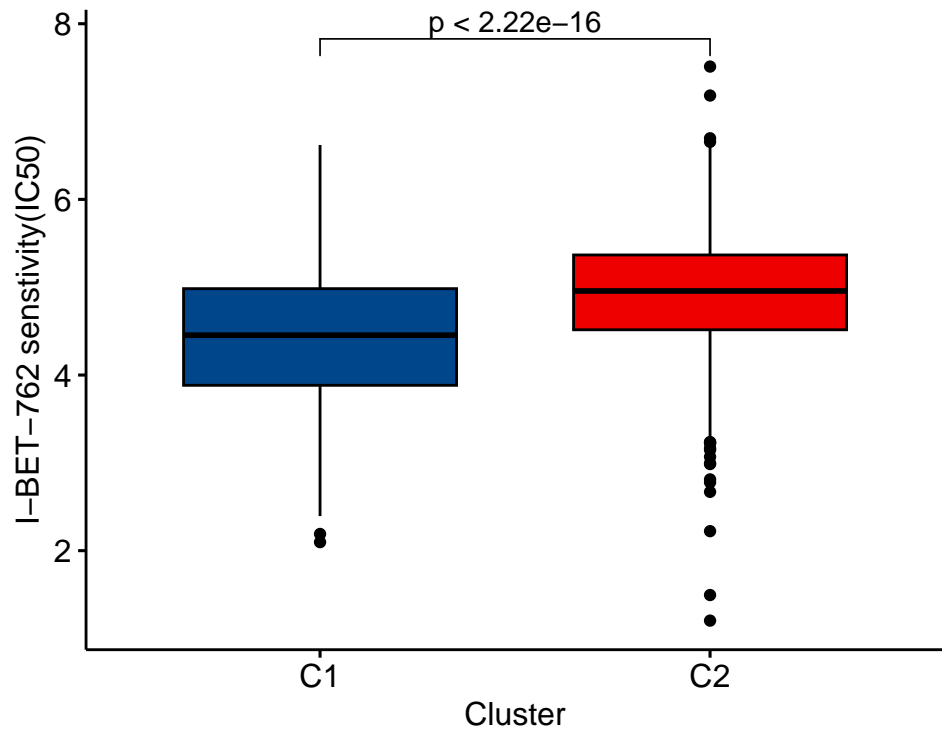

Supplement: Supplementary file 2 — Supplementary file2 (ZIP 3179 KB) [file 10238_2024_1372_MOESM2_ESM.zip › Supplementary Material/Drug2/drugSenstivity.I-BET-762.pdf]

Cluster 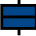 C1 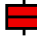 C2

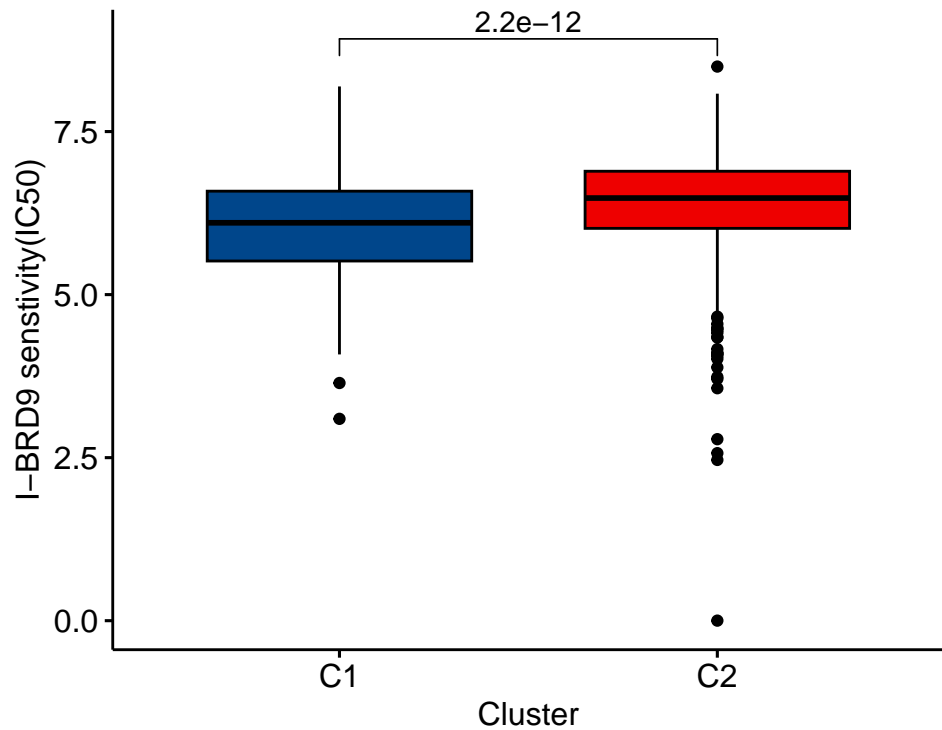

Supplement: Supplementary file 2 — Supplementary file2 (ZIP 3179 KB) [file 10238_2024_1372_MOESM2_ESM.zip › Supplementary Material/Drug2/drugSenstivity.I-BRD9.pdf]

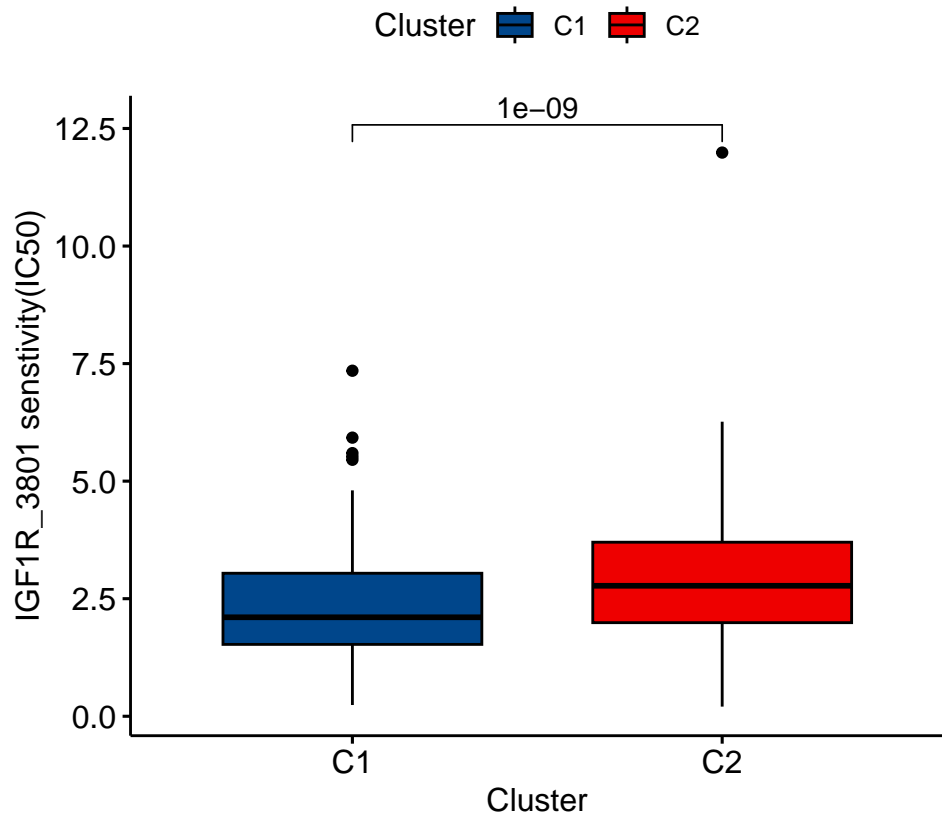

Supplement: Supplementary file 2 — Supplementary file2 (ZIP 3179 KB) [file 10238_2024_1372_MOESM2_ESM.zip › Supplementary Material/Drug2/drugSenstivity.IGF1R_3801.pdf]

Cluster C1 C2

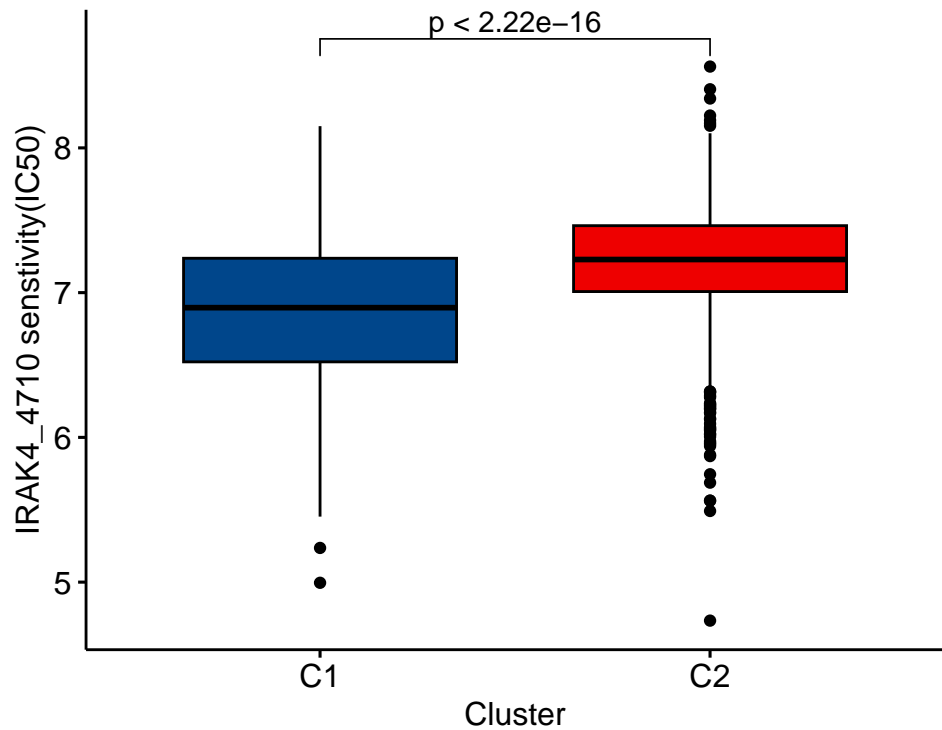

Supplement: Supplementary file 2 — Supplementary file2 (ZIP 3179 KB) [file 10238_2024_1372_MOESM2_ESM.zip › Supplementary Material/Drug2/drugSenstivity.IRAK4_4710.pdf]

Cluster C1 C2

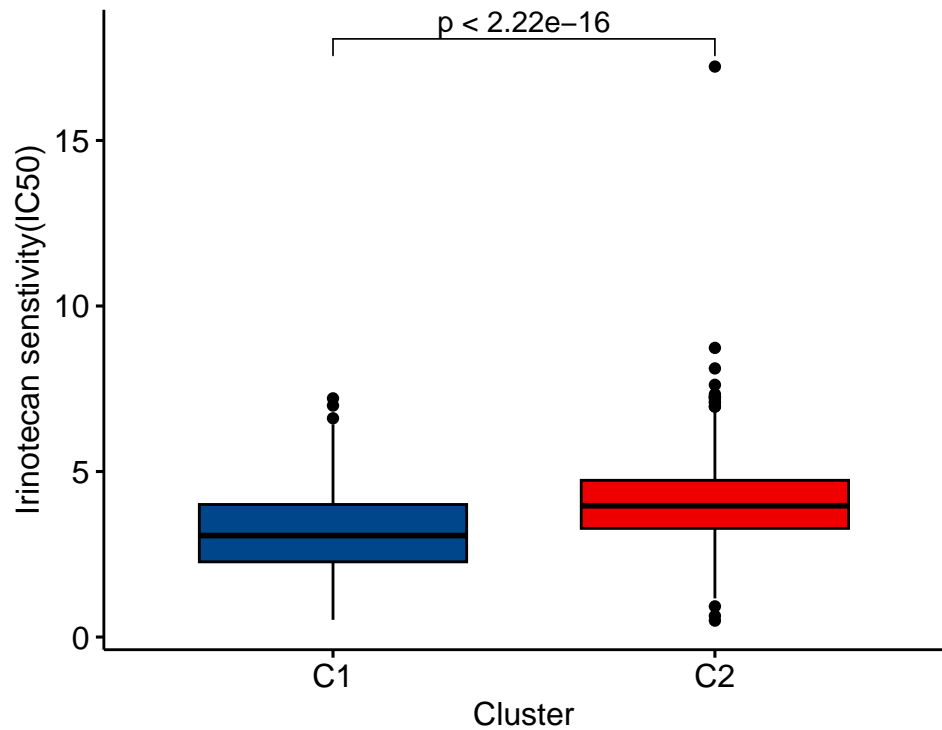

Supplement: Supplementary file 2 — Supplementary file2 (ZIP 3179 KB) [file 10238_2024_1372_MOESM2_ESM.zip › Supplementary Material/Drug2/drugSenstivity.Irinotecan.pdf]

Cluster C1 C2

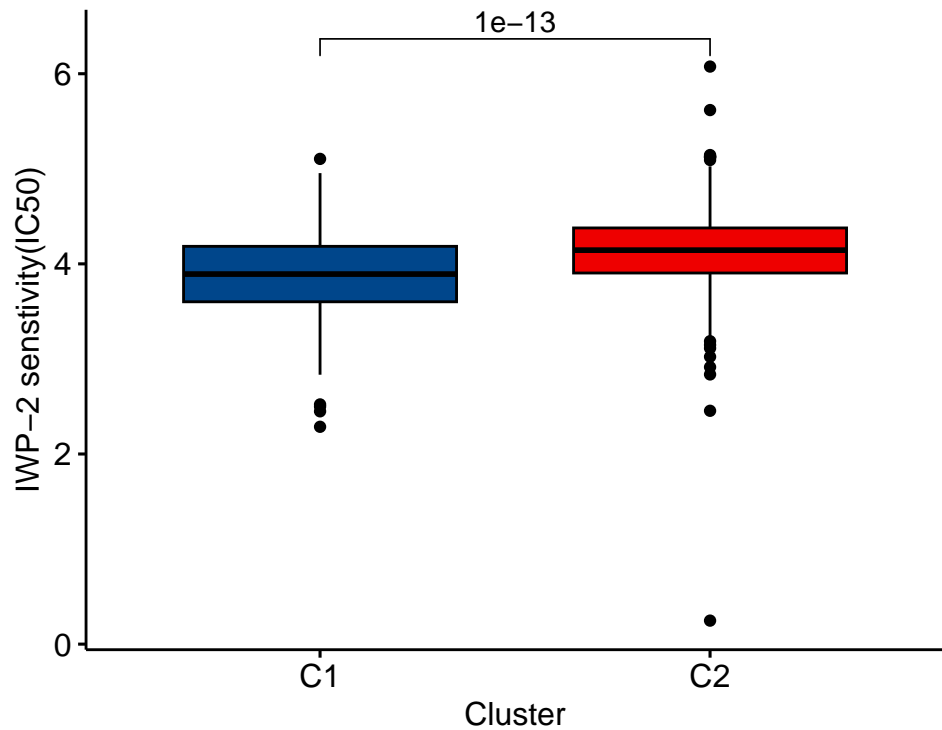

Supplement: Supplementary file 2 — Supplementary file2 (ZIP 3179 KB) [file 10238_2024_1372_MOESM2_ESM.zip › Supplementary Material/Drug2/drugSenstivity.IWP-2.pdf]

Cluster 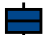 C1 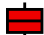 C2

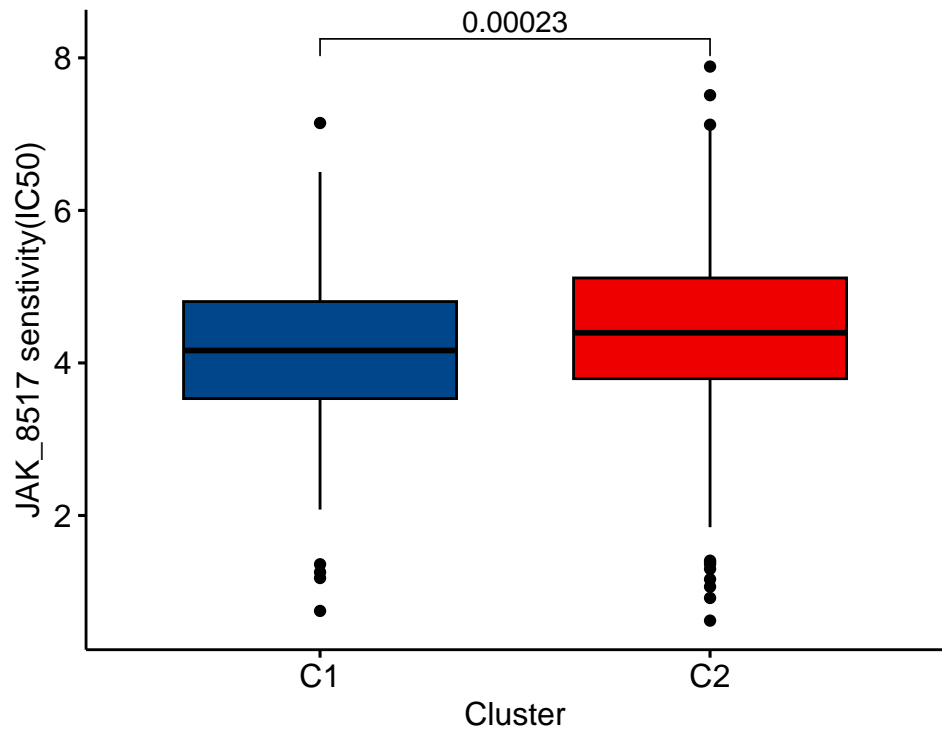

Supplement: Supplementary file 2 — Supplementary file2 (ZIP 3179 KB) [file 10238_2024_1372_MOESM2_ESM.zip › Supplementary Material/Drug2/drugSenstivity.JAK_8517.pdf]

Cluster 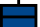 C1 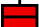 C2

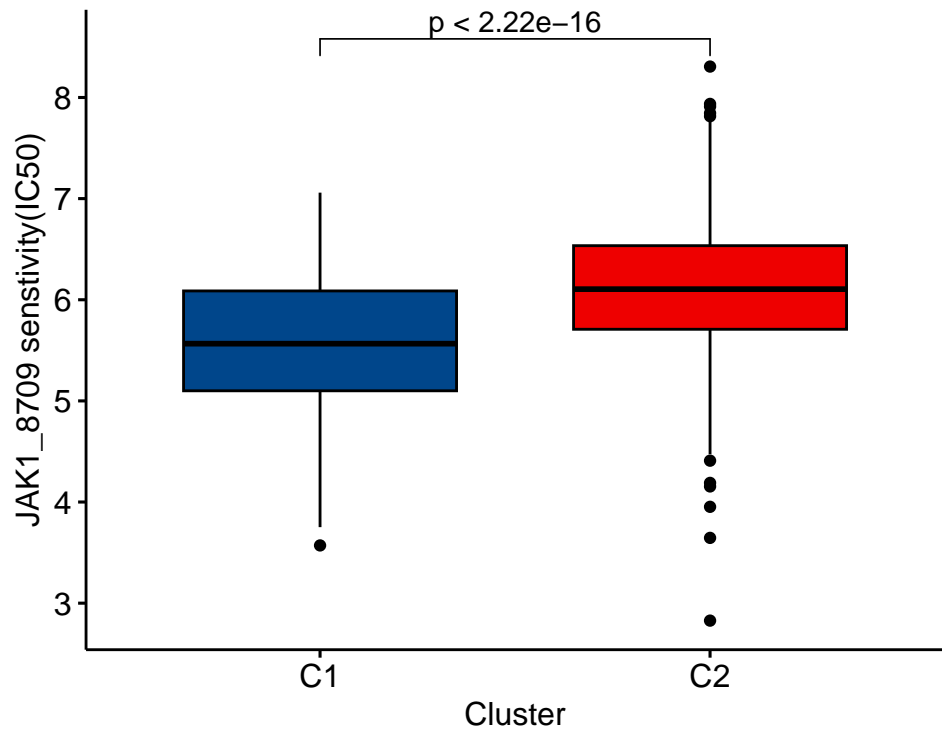

Supplement: Supplementary file 2 — Supplementary file2 (ZIP 3179 KB) [file 10238_2024_1372_MOESM2_ESM.zip › Supplementary Material/Drug2/drugSenstivity.JAK1_8709.pdf]

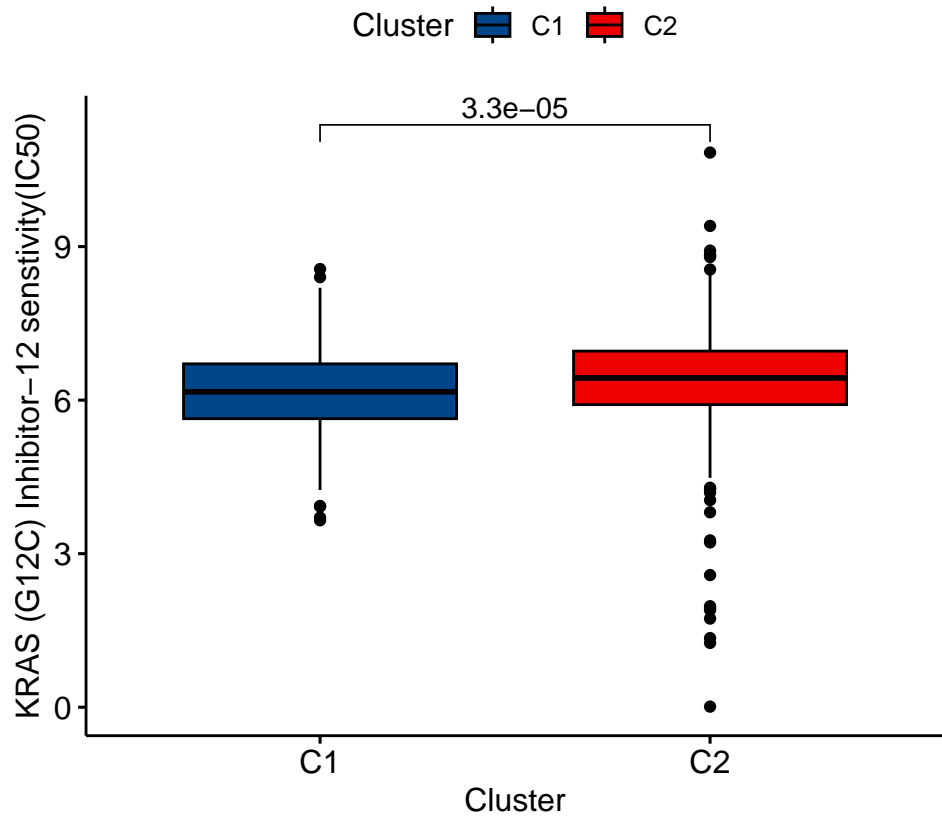

Supplement: Supplementary file 2 — Supplementary file2 (ZIP 3179 KB) [file 10238_2024_1372_MOESM2_ESM.zip › Supplementary Material/Drug2/drugSenstivity.KRAS (G12C) Inhibitor-12.pdf]

Cluster 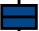 C1 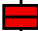 C2

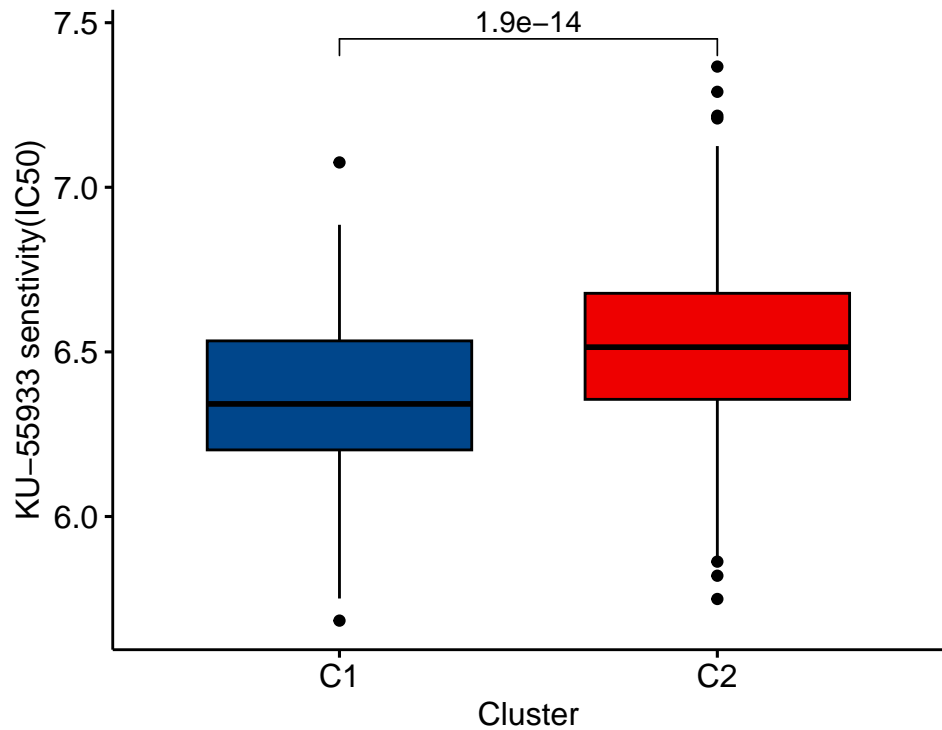

Supplement: Supplementary file 2 — Supplementary file2 (ZIP 3179 KB) [file 10238_2024_1372_MOESM2_ESM.zip › Supplementary Material/Drug2/drugSenstivity.KU-55933.pdf]

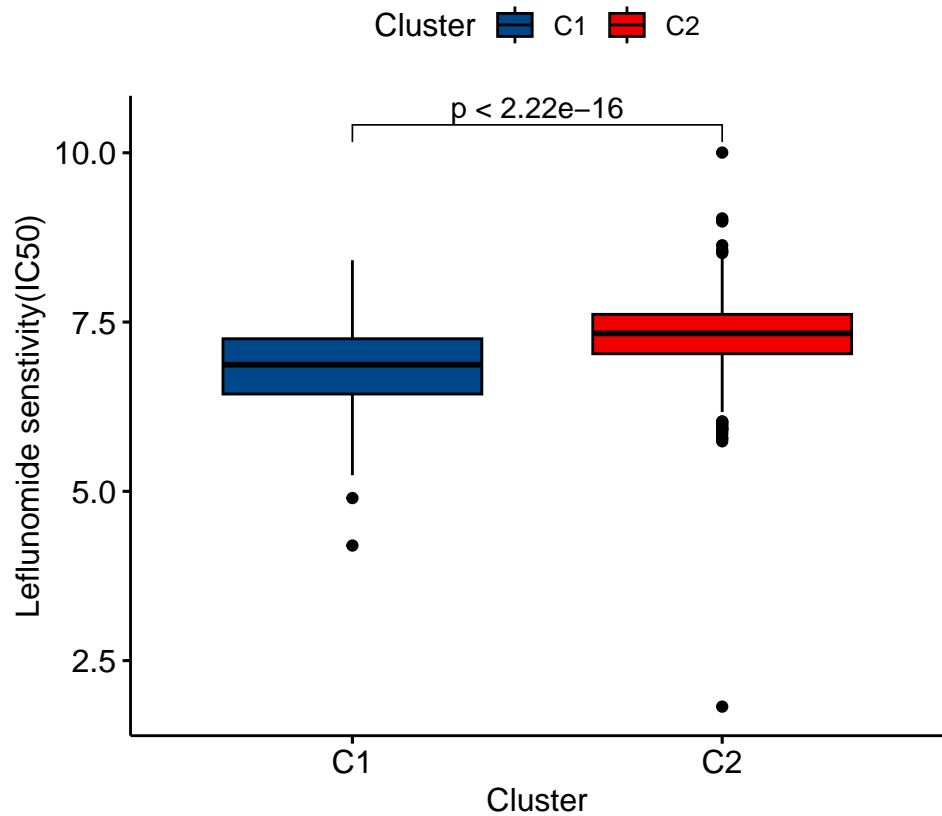

Supplement: Supplementary file 2 — Supplementary file2 (ZIP 3179 KB) [file 10238_2024_1372_MOESM2_ESM.zip › Supplementary Material/Drug2/drugSenstivity.Leflunomide.pdf]

Cluster 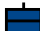 C1 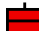 C2

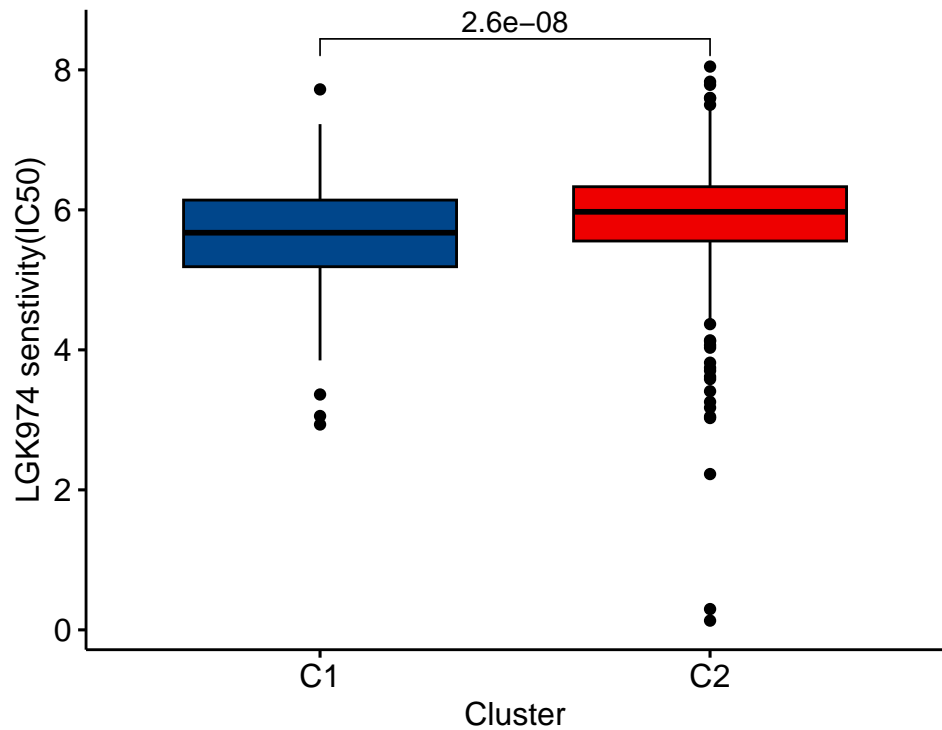

Supplement: Supplementary file 2 — Supplementary file2 (ZIP 3179 KB) [file 10238_2024_1372_MOESM2_ESM.zip › Supplementary Material/Drug2/drugSenstivity.LGK974.pdf]

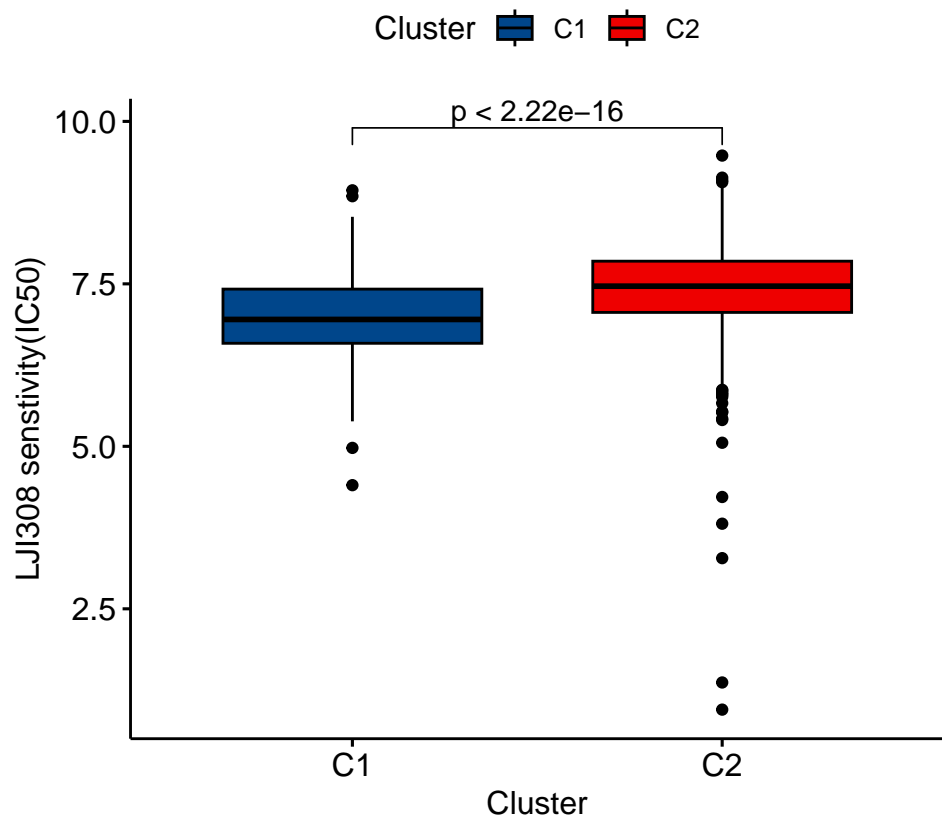

Supplement: Supplementary file 2 — Supplementary file2 (ZIP 3179 KB) [file 10238_2024_1372_MOESM2_ESM.zip › Supplementary Material/Drug2/drugSenstivity.LJI308.pdf]

Cluster 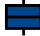 C1 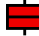 C2

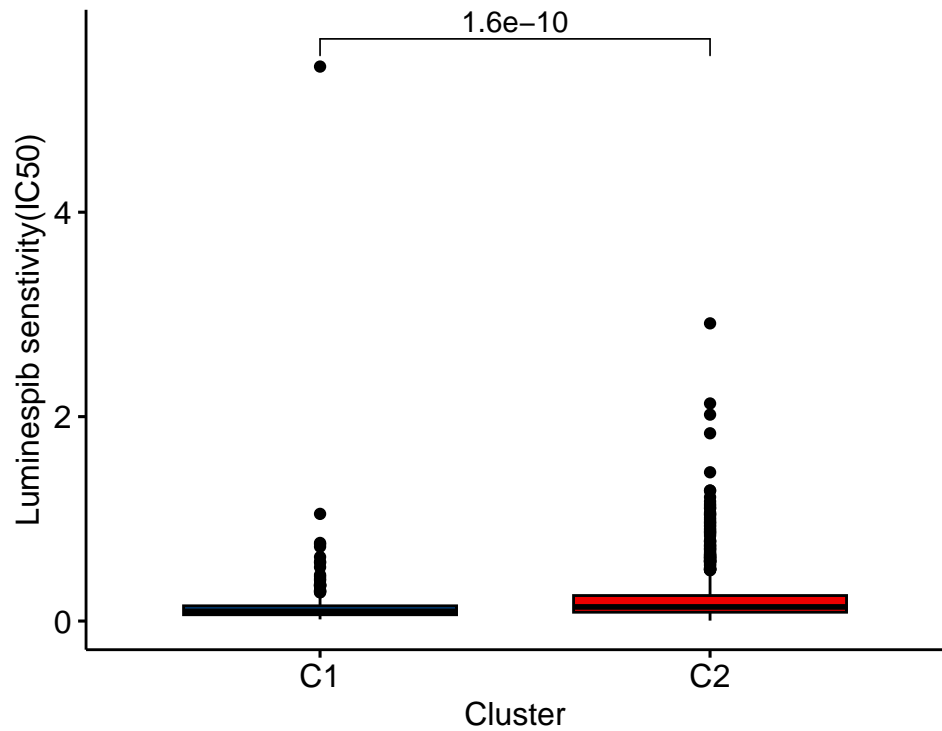

Supplement: Supplementary file 2 — Supplementary file2 (ZIP 3179 KB) [file 10238_2024_1372_MOESM2_ESM.zip › Supplementary Material/Drug2/drugSenstivity.Luminespib.pdf]

Cluster 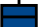 C1 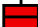 C2

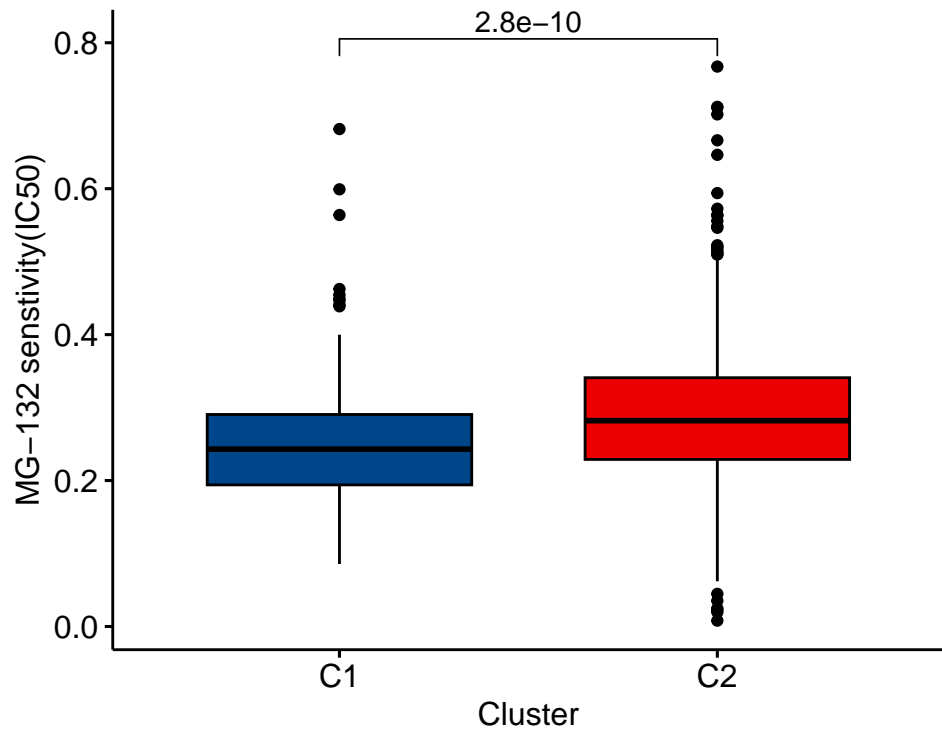

Supplement: Supplementary file 2 — Supplementary file2 (ZIP 3179 KB) [file 10238_2024_1372_MOESM2_ESM.zip › Supplementary Material/Drug2/drugSenstivity.MG-132.pdf]

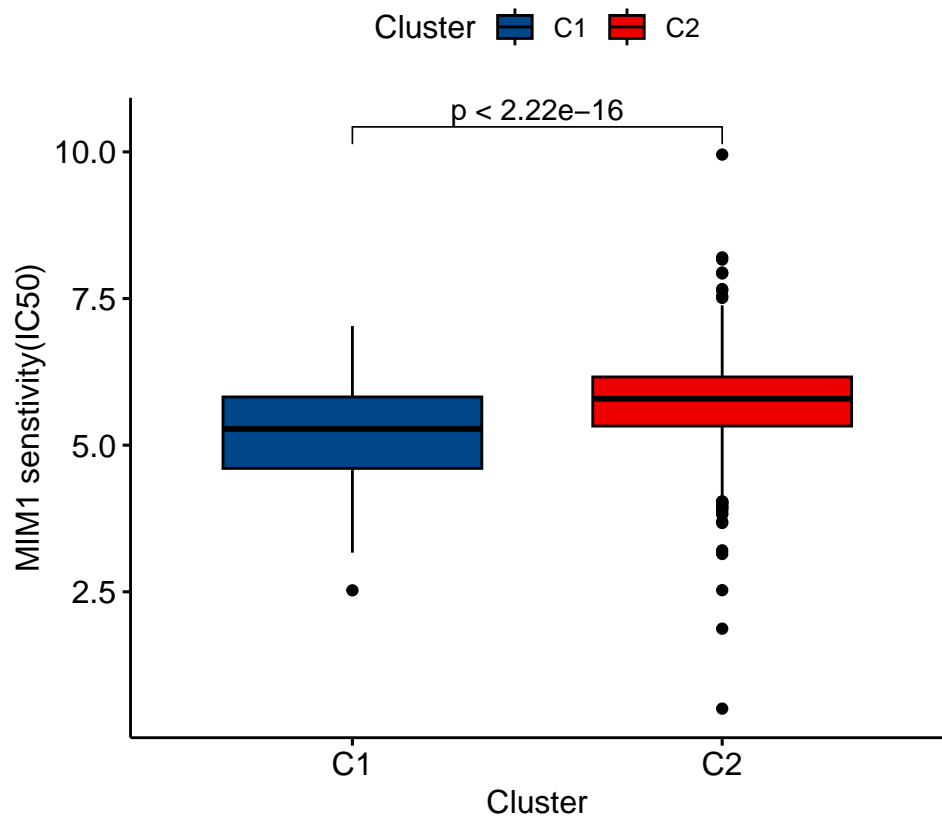

Supplement: Supplementary file 2 — Supplementary file2 (ZIP 3179 KB) [file 10238_2024_1372_MOESM2_ESM.zip › Supplementary Material/Drug2/drugSenstivity.MIM1.pdf]

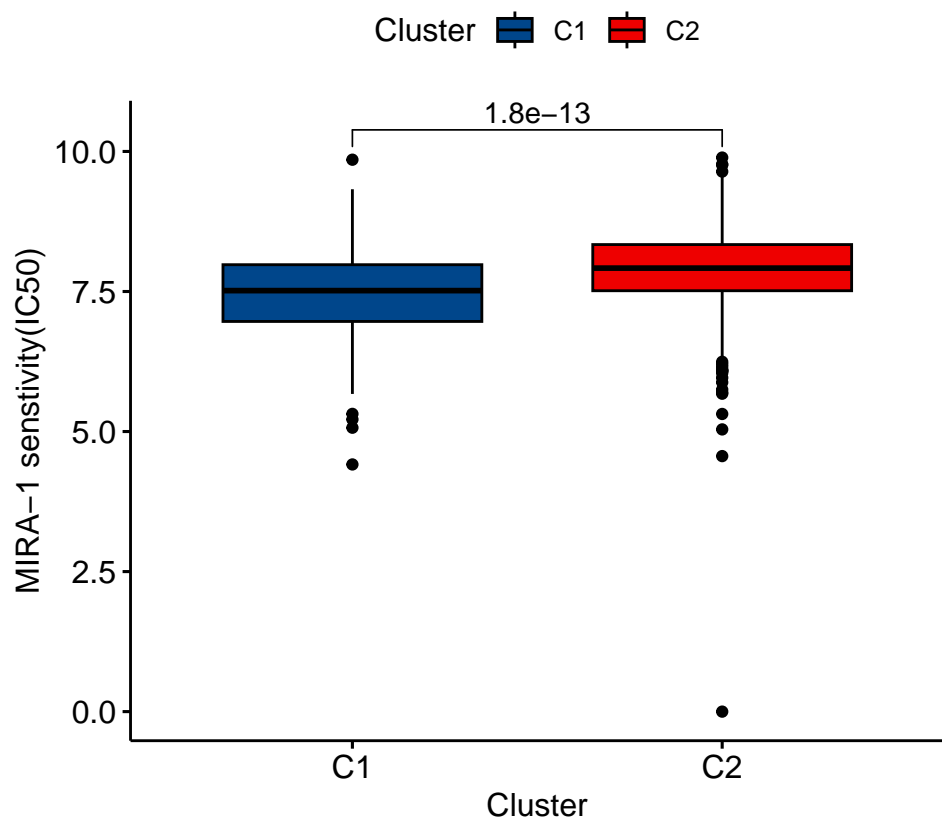

Supplement: Supplementary file 2 — Supplementary file2 (ZIP 3179 KB) [file 10238_2024_1372_MOESM2_ESM.zip › Supplementary Material/Drug2/drugSenstivity.MIRA-1.pdf]

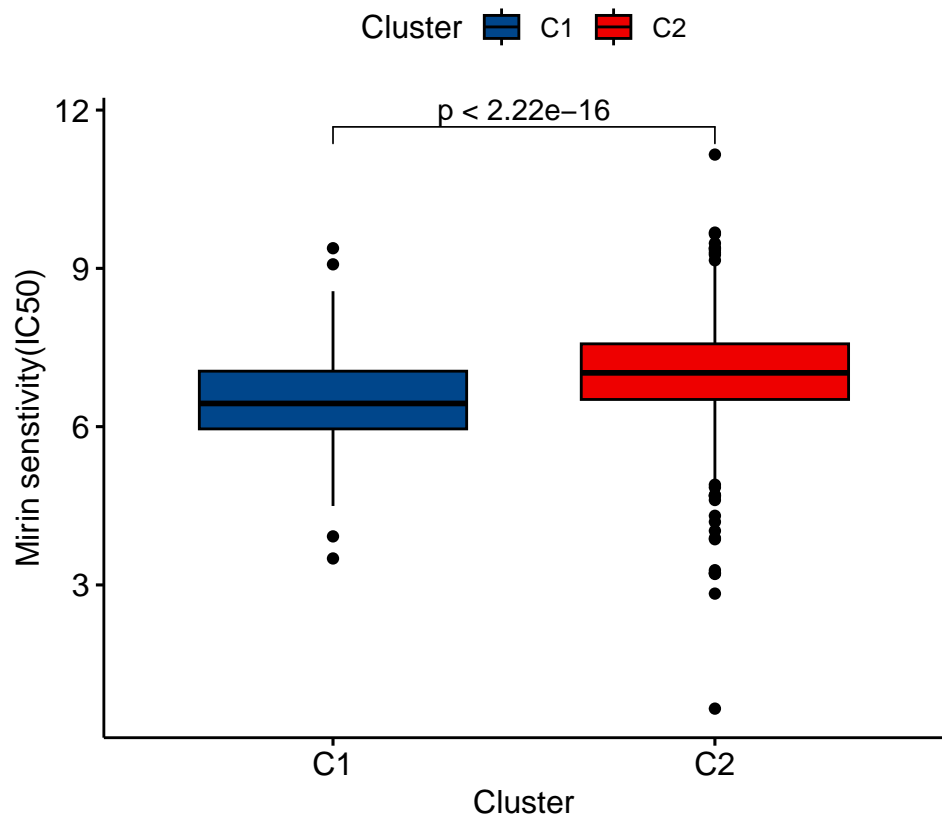

Supplement: Supplementary file 2 — Supplementary file2 (ZIP 3179 KB) [file 10238_2024_1372_MOESM2_ESM.zip › Supplementary Material/Drug2/drugSenstivity.Mirin.pdf]

Cluster 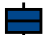 C1 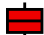 C2

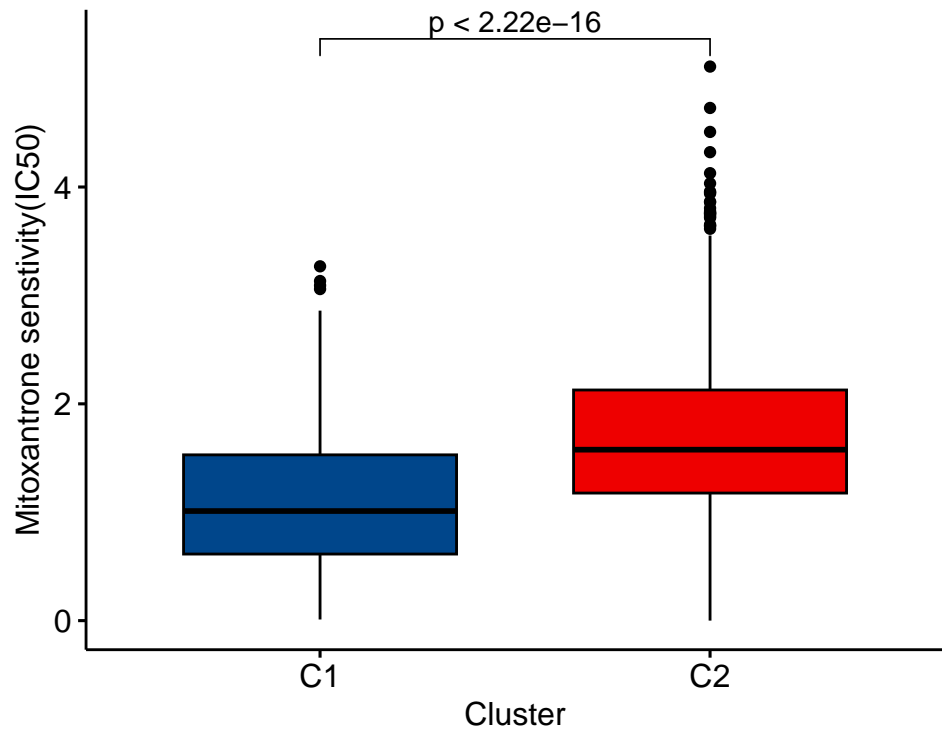

Supplement: Supplementary file 2 — Supplementary file2 (ZIP 3179 KB) [file 10238_2024_1372_MOESM2_ESM.zip › Supplementary Material/Drug2/drugSenstivity.Mitoxantrone.pdf]

MK-1775 sensitivity(IC50)

Cluster C1 C2

$p < 2.22e-16$

C1

C2

Cluster

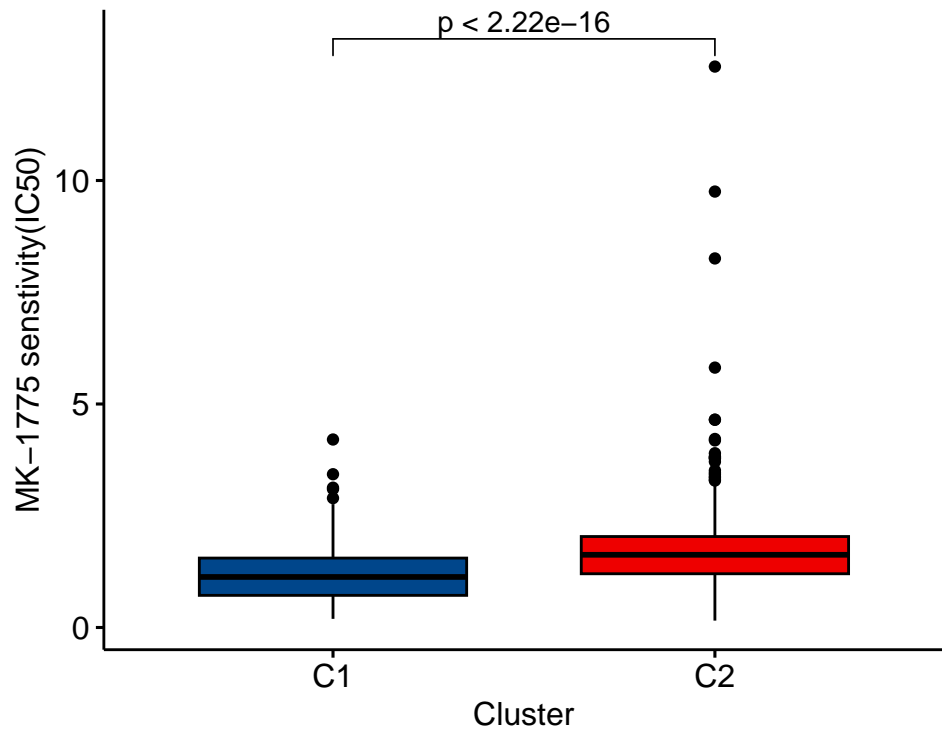

Supplement: Supplementary file 2 — Supplementary file2 (ZIP 3179 KB) [file 10238_2024_1372_MOESM2_ESM.zip › Supplementary Material/Drug2/drugSenstivity.MK-1775.pdf]

Cluster 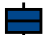 C1 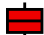 C2

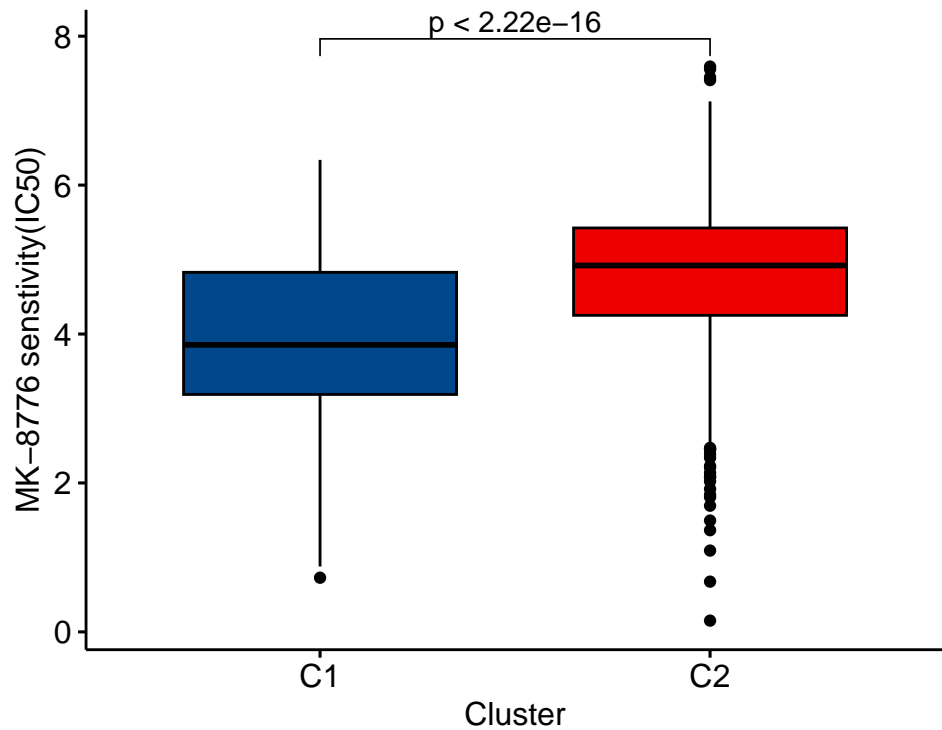

Supplement: Supplementary file 2 — Supplementary file2 (ZIP 3179 KB) [file 10238_2024_1372_MOESM2_ESM.zip › Supplementary Material/Drug2/drugSenstivity.MK-8776.pdf]

Cluster 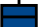 C1 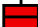 C2

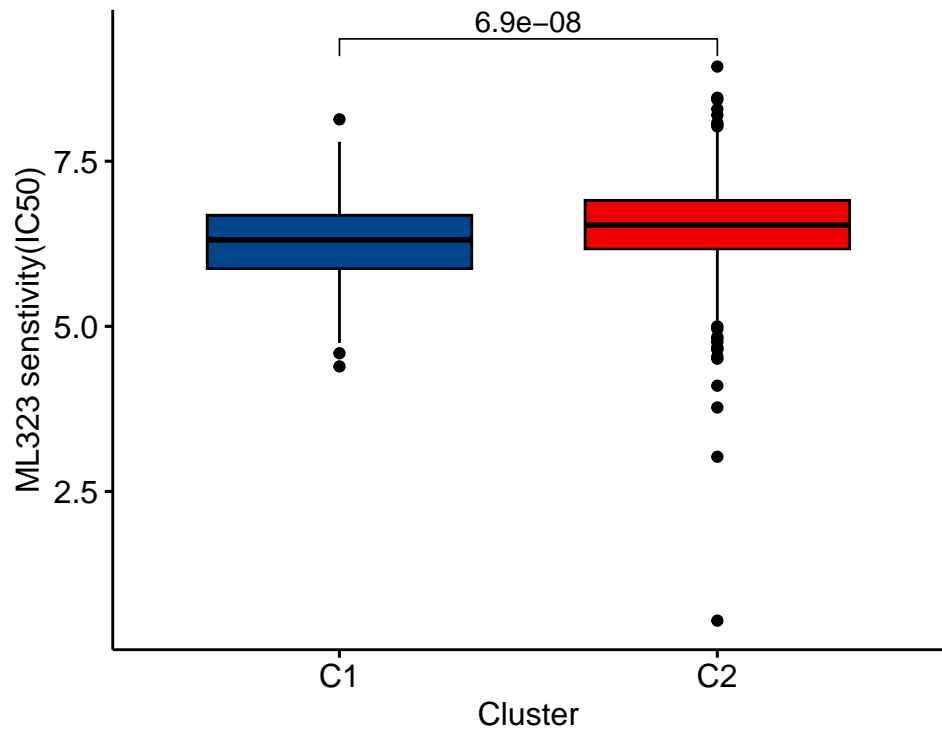

Supplement: Supplementary file 2 — Supplementary file2 (ZIP 3179 KB) [file 10238_2024_1372_MOESM2_ESM.zip › Supplementary Material/Drug2/drugSenstivity.ML323.pdf]

MN-64 sensitivity(IC50)

Cluster 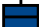 C1 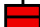 C2

$3.9e-05$

C1

C2

Cluster

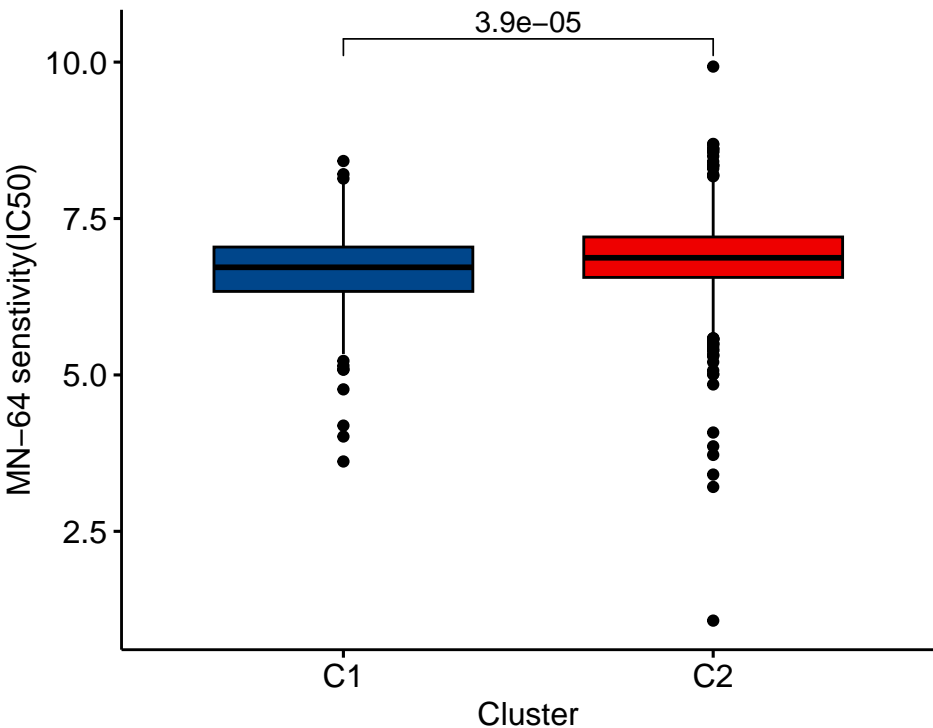

Supplement: Supplementary file 2 — Supplementary file2 (ZIP 3179 KB) [file 10238_2024_1372_MOESM2_ESM.zip › Supplementary Material/Drug2/drugSenstivity.MN-64.pdf]

Cluster 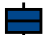 C1 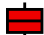 C2

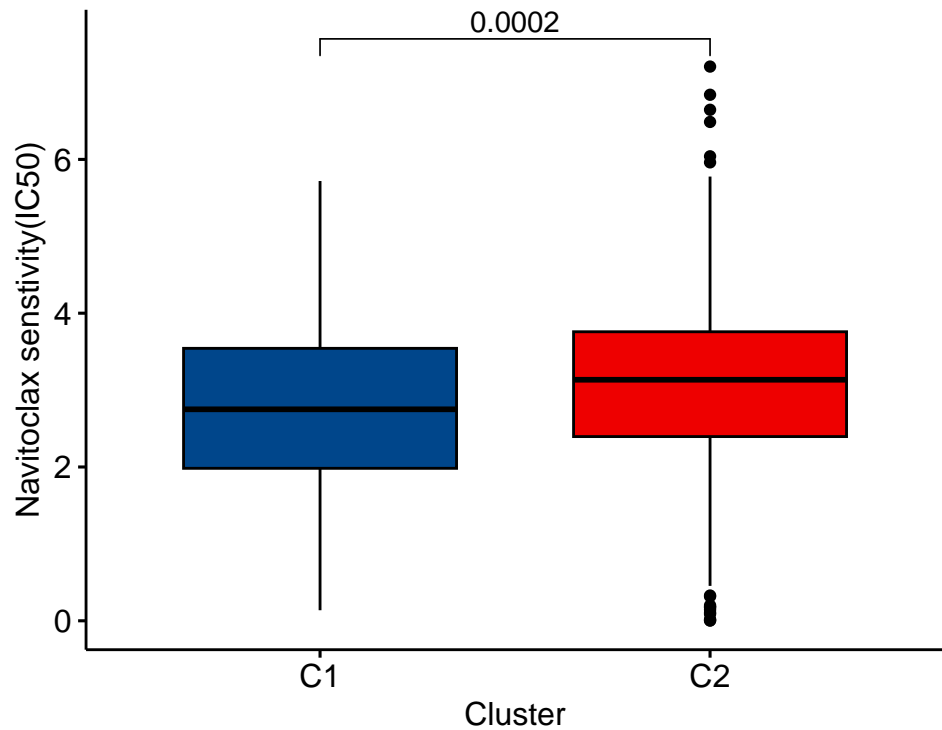

Supplement: Supplementary file 2 — Supplementary file2 (ZIP 3179 KB) [file 10238_2024_1372_MOESM2_ESM.zip › Supplementary Material/Drug2/drugSenstivity.Navitoclax.pdf]
