# Supplementary figures and images for: The prognostic genes model of breast cancer drug resistance based on single-cell sequencing analysis and transcriptome analysis (part 1 of 2)
Source: Clin Exp Med. 2024 May 25;24(1):113. doi: 10.1007/s10238-024-01372-6 (PMC11127859; doi:10.1007/s10238-024-01372-6)

risk low high

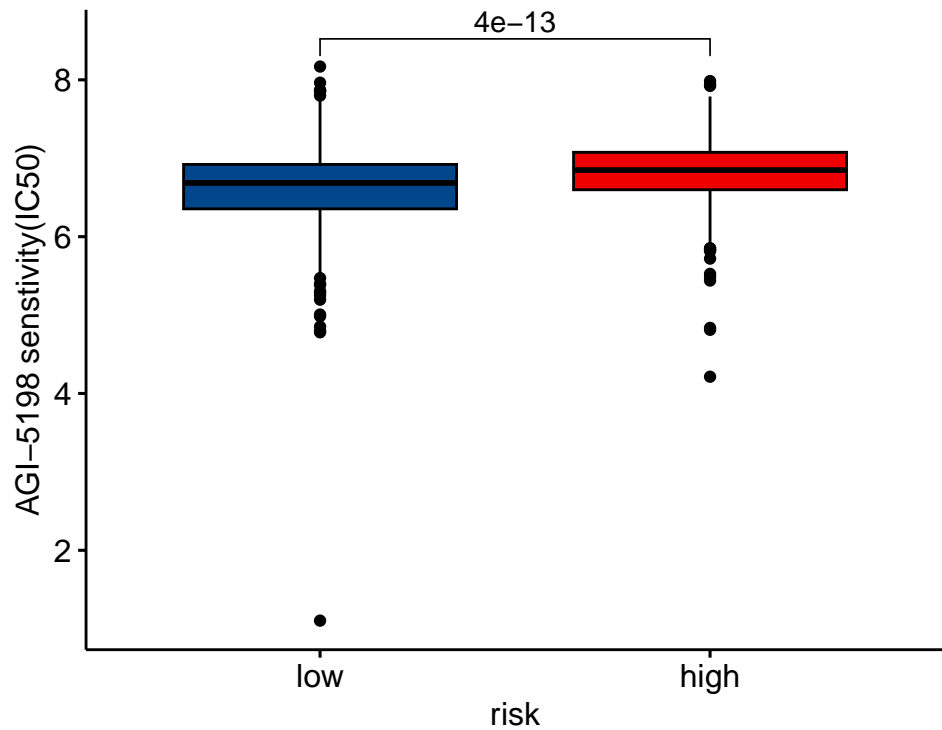

Supplement: Supplementary file 2 — Supplementary file2 (ZIP 3179 KB) [file 10238_2024_1372_MOESM2_ESM.zip › Supplementary Material/Drug1/drugSenstivity.AGI-5198.pdf]

risk low high

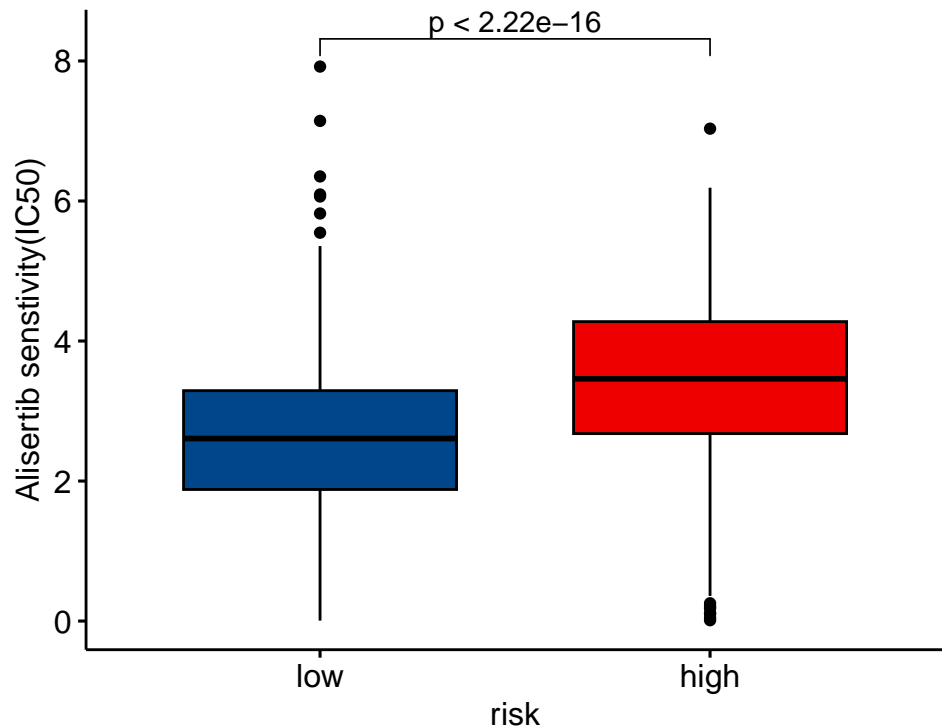

Supplement: Supplementary file 2 — Supplementary file2 (ZIP 3179 KB) [file 10238_2024_1372_MOESM2_ESM.zip › Supplementary Material/Drug1/drugSenstivity.Alisertib.pdf]

risk low high

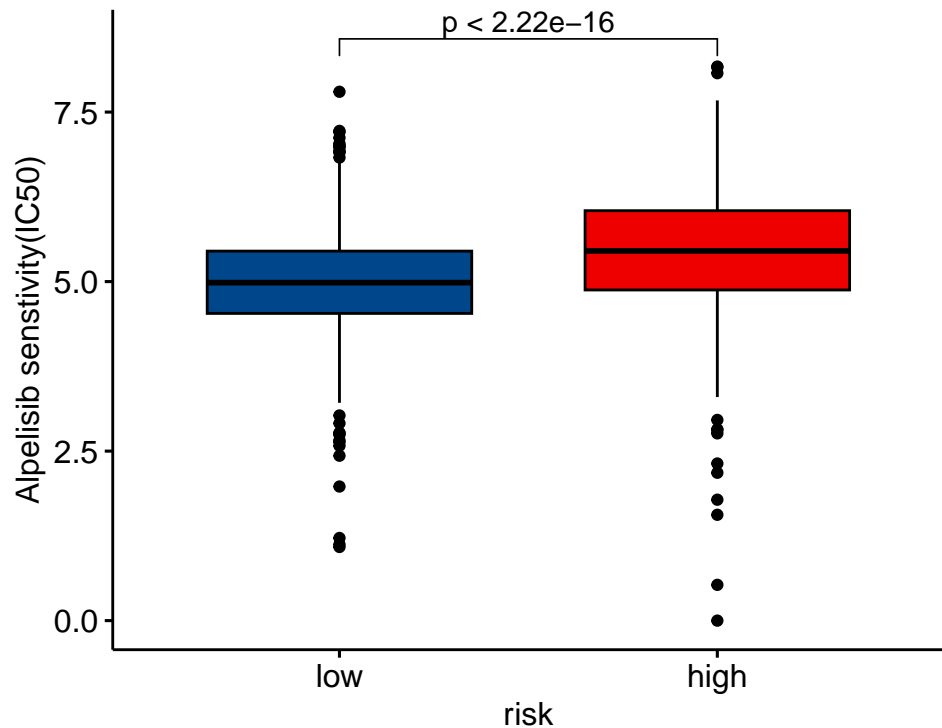

Supplement: Supplementary file 2 — Supplementary file2 (ZIP 3179 KB) [file 10238_2024_1372_MOESM2_ESM.zip › Supplementary Material/Drug1/drugSenstivity.Alpelisib.pdf]

risk low high

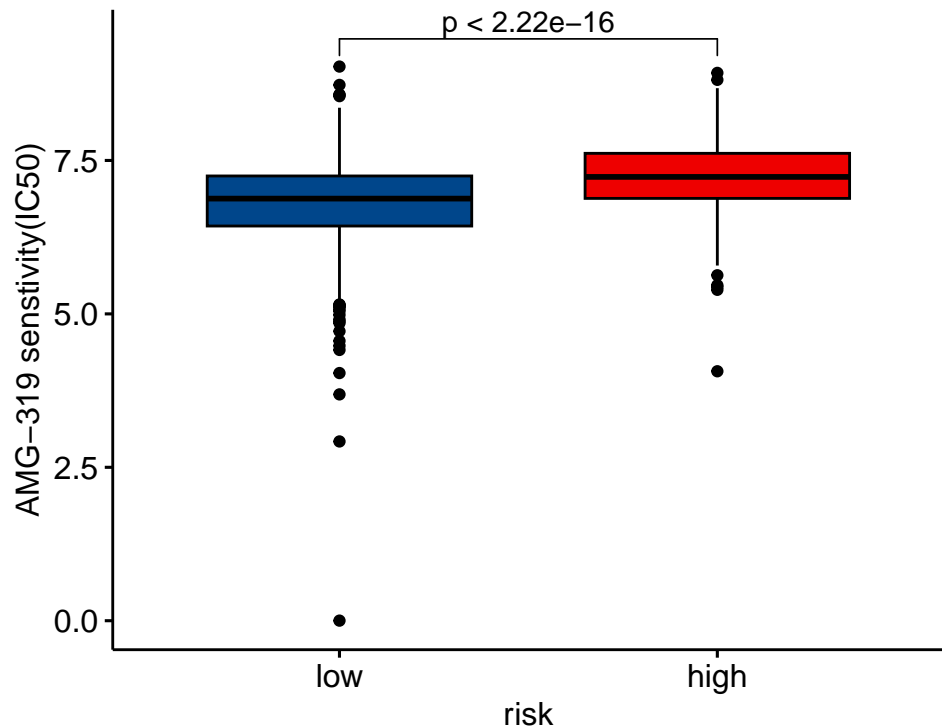

Supplement: Supplementary file 2 — Supplementary file2 (ZIP 3179 KB) [file 10238_2024_1372_MOESM2_ESM.zip › Supplementary Material/Drug1/drugSenstivity.AMG-319.pdf]

AT13148 sensitivity(IC50)

risk low high

$p < 2.22e-16$

low

high

risk

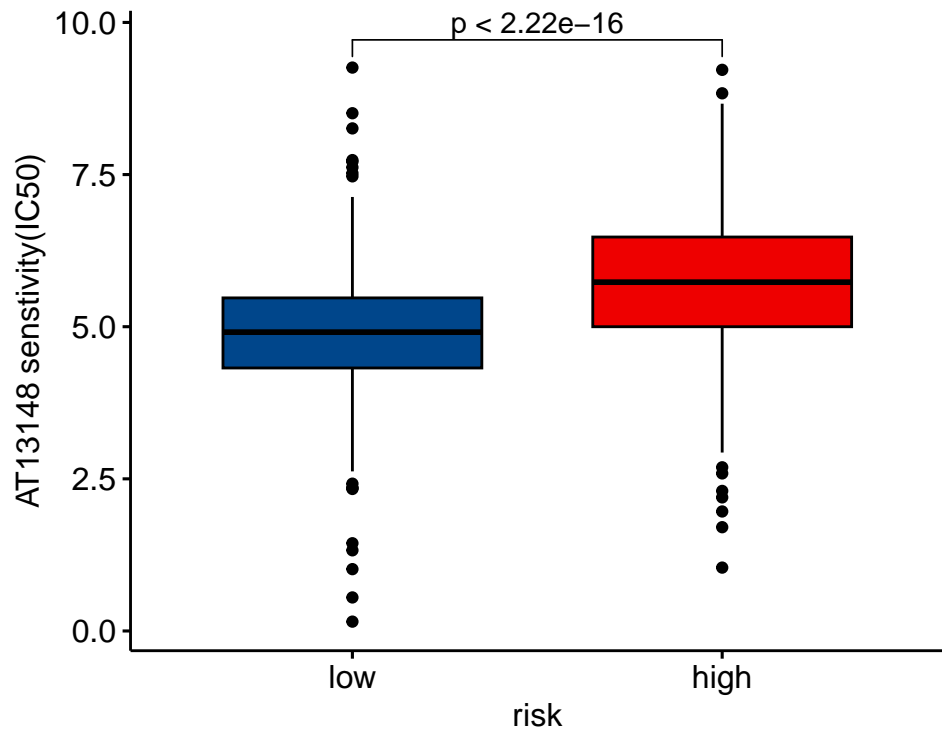

Supplement: Supplementary file 2 — Supplementary file2 (ZIP 3179 KB) [file 10238_2024_1372_MOESM2_ESM.zip › Supplementary Material/Drug1/drugSenstivity.AT13148.pdf]

risk low high

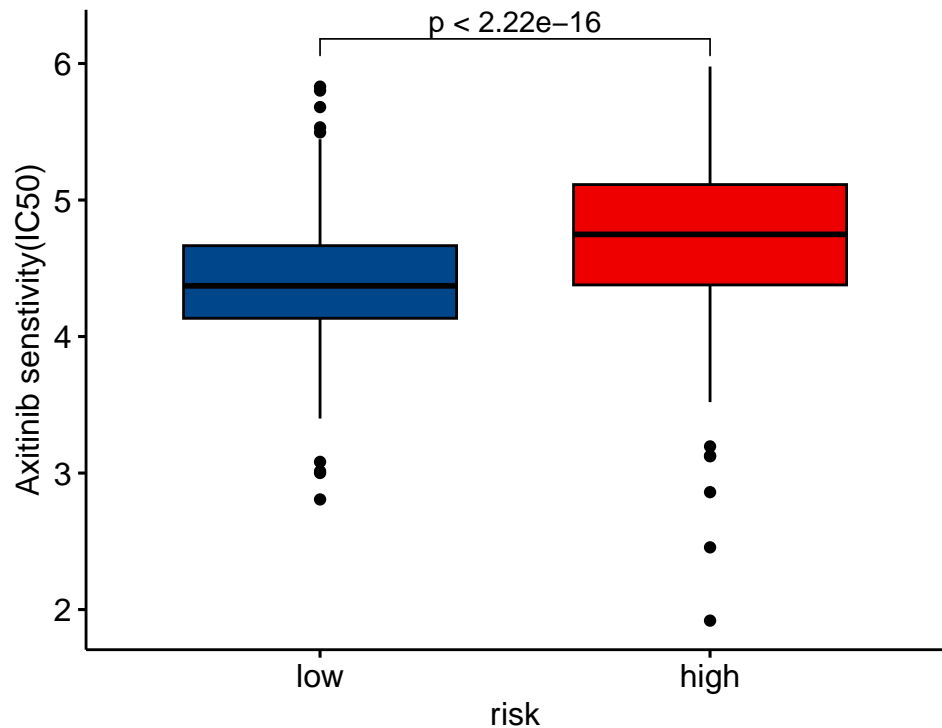

Supplement: Supplementary file 2 — Supplementary file2 (ZIP 3179 KB) [file 10238_2024_1372_MOESM2_ESM.zip › Supplementary Material/Drug1/drugSenstivity.Axitinib.pdf]

risk low high

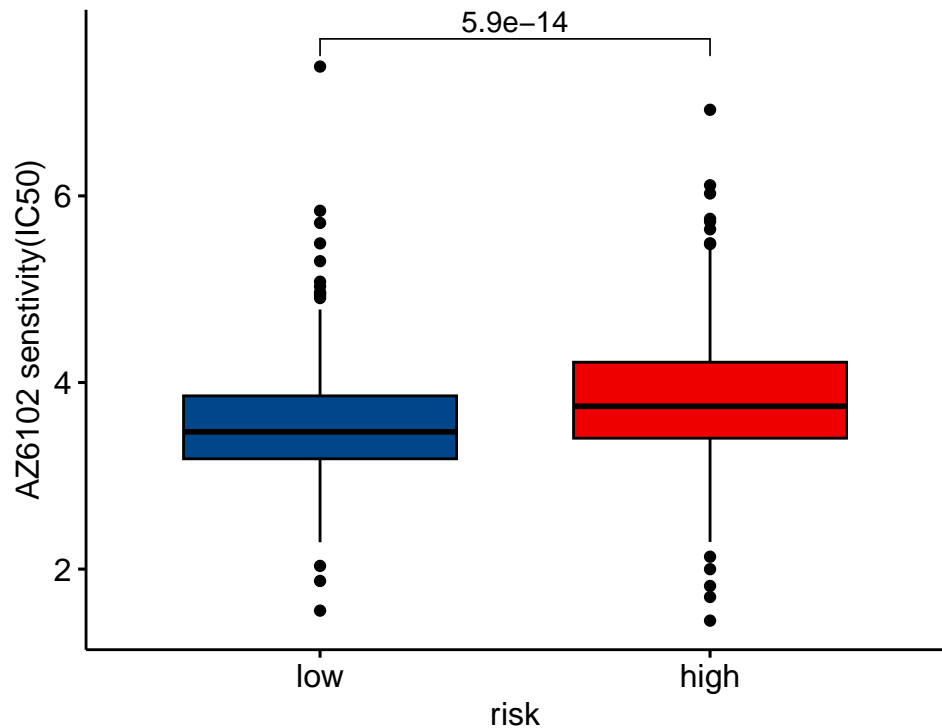

Supplement: Supplementary file 2 — Supplementary file2 (ZIP 3179 KB) [file 10238_2024_1372_MOESM2_ESM.zip › Supplementary Material/Drug1/drugSenstivity.AZ6102.pdf]

risk 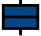 low 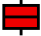 high

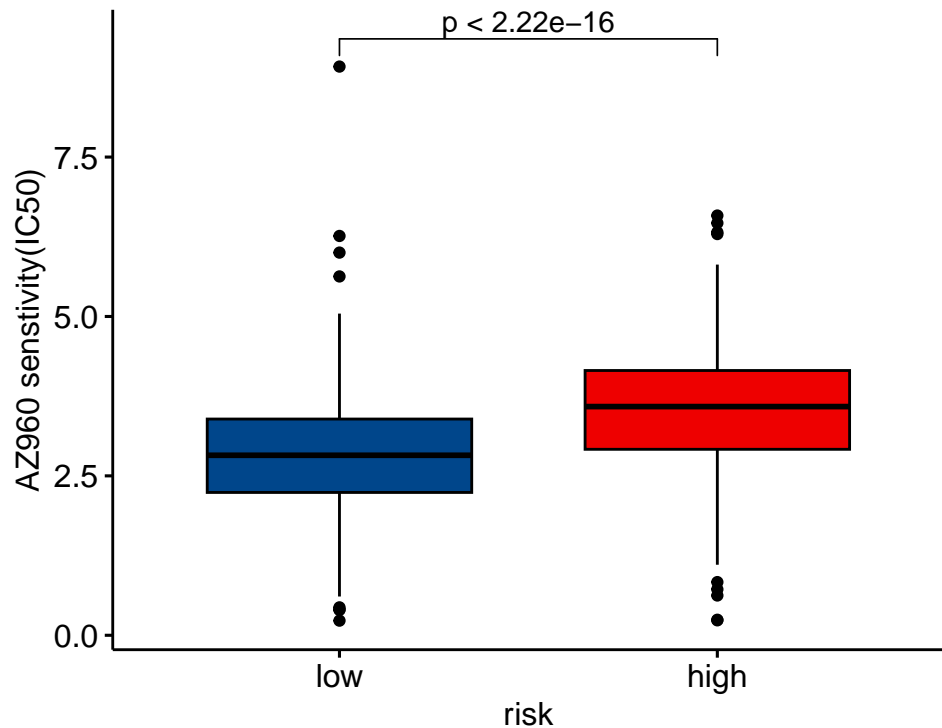

Supplement: Supplementary file 2 — Supplementary file2 (ZIP 3179 KB) [file 10238_2024_1372_MOESM2_ESM.zip › Supplementary Material/Drug1/drugSenstivity.AZ960.pdf]

risk low high

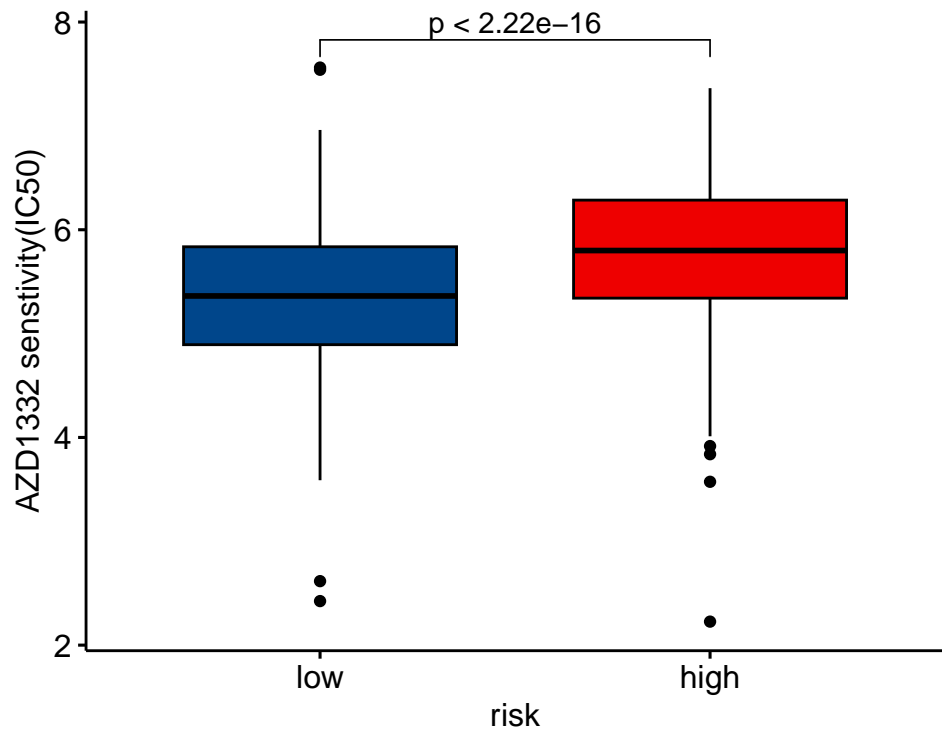

Supplement: Supplementary file 2 — Supplementary file2 (ZIP 3179 KB) [file 10238_2024_1372_MOESM2_ESM.zip › Supplementary Material/Drug1/drugSenstivity.AZD1332.pdf]

risk low high

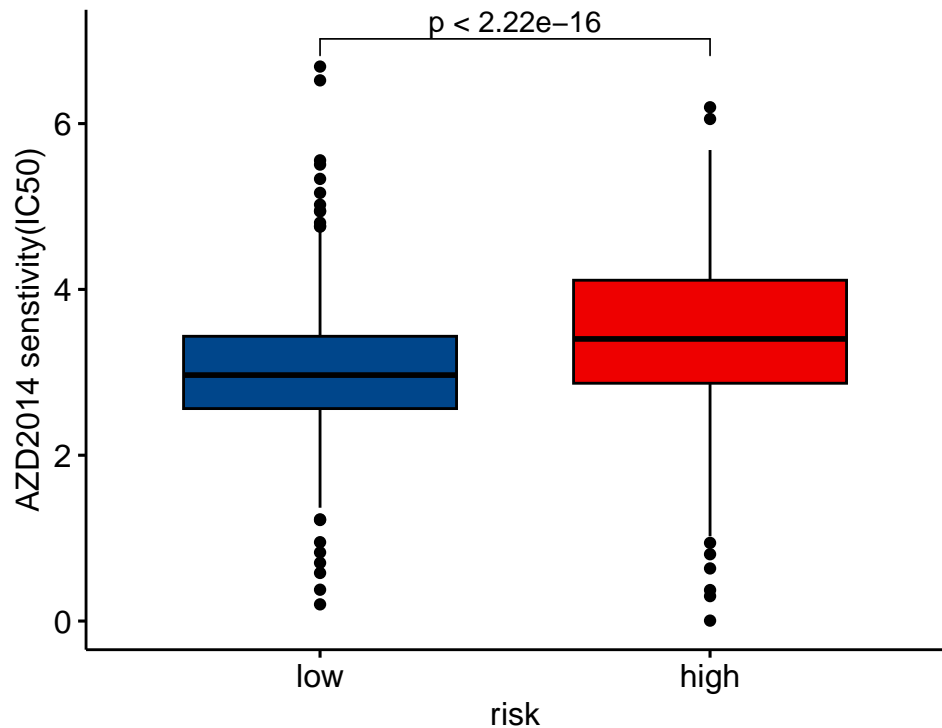

Supplement: Supplementary file 2 — Supplementary file2 (ZIP 3179 KB) [file 10238_2024_1372_MOESM2_ESM.zip › Supplementary Material/Drug1/drugSenstivity.AZD2014.pdf]

risk low high

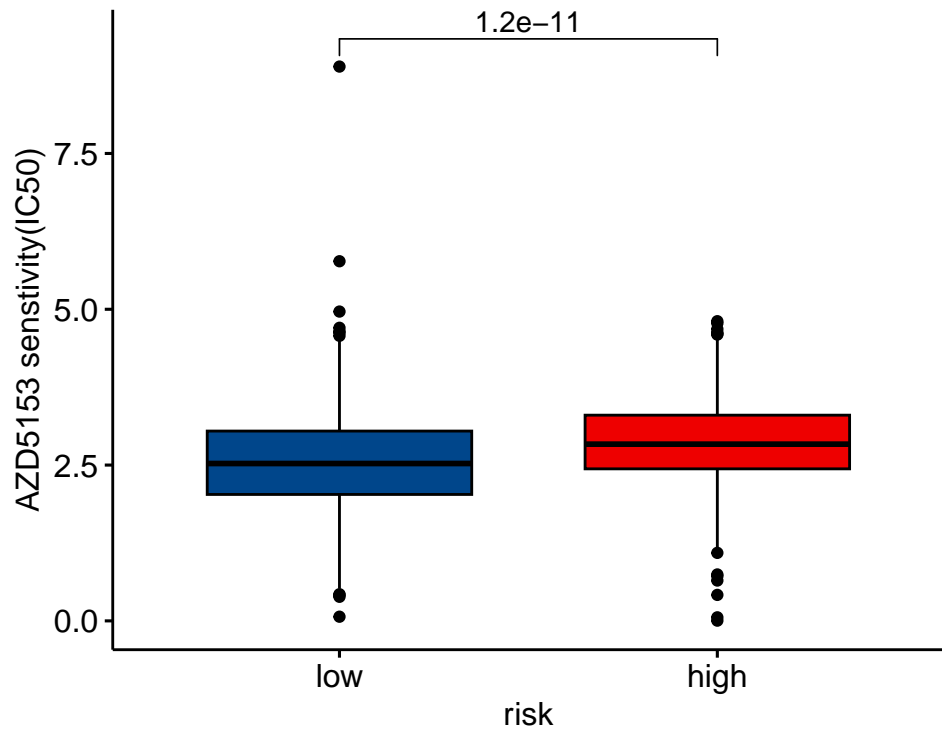

Supplement: Supplementary file 2 — Supplementary file2 (ZIP 3179 KB) [file 10238_2024_1372_MOESM2_ESM.zip › Supplementary Material/Drug1/drugSenstivity.AZD5153.pdf]

risk low high

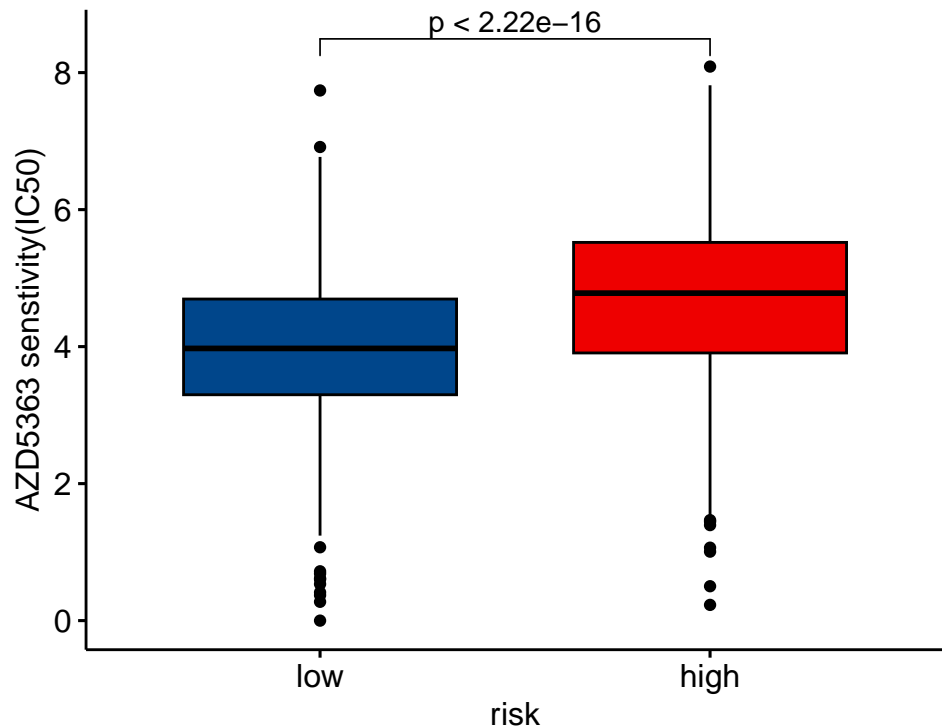

Supplement: Supplementary file 2 — Supplementary file2 (ZIP 3179 KB) [file 10238_2024_1372_MOESM2_ESM.zip › Supplementary Material/Drug1/drugSenstivity.AZD5363.pdf]

risk low high

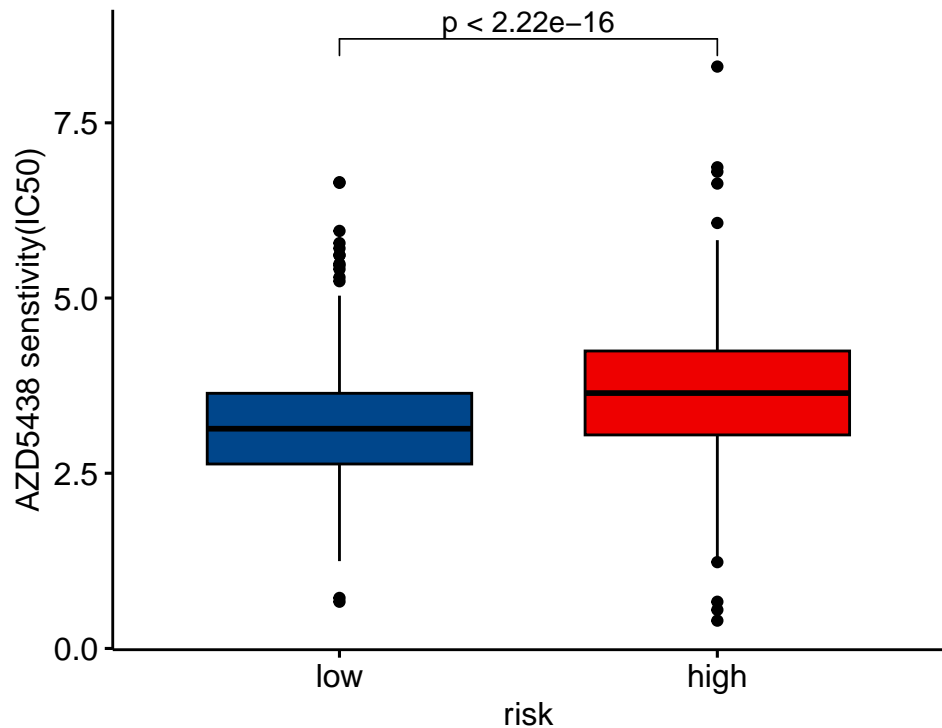

Supplement: Supplementary file 2 — Supplementary file2 (ZIP 3179 KB) [file 10238_2024_1372_MOESM2_ESM.zip › Supplementary Material/Drug1/drugSenstivity.AZD5438.pdf]

risk low high

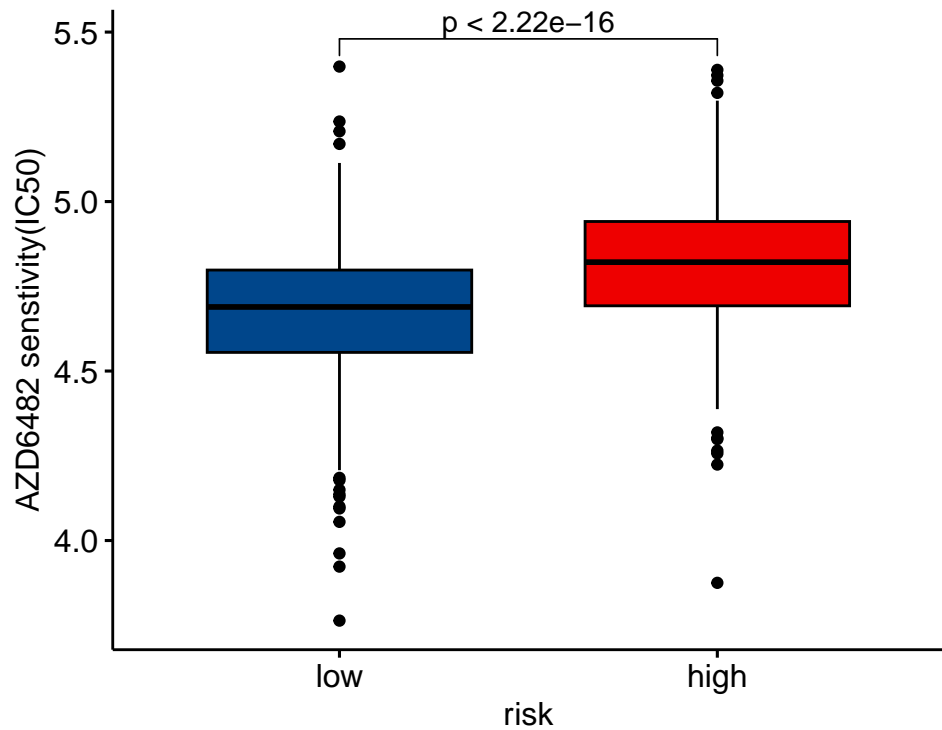

Supplement: Supplementary file 2 — Supplementary file2 (ZIP 3179 KB) [file 10238_2024_1372_MOESM2_ESM.zip › Supplementary Material/Drug1/drugSenstivity.AZD6482.pdf]

AZD6738 sensitivity(IC50)

risk low high

8.9e-11

low

high

risk

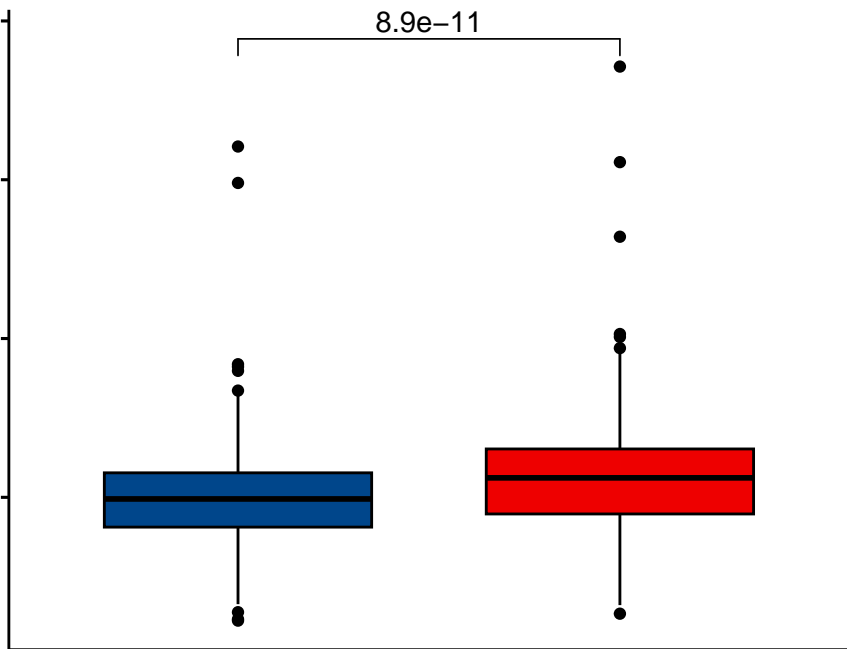

Supplement: Supplementary file 2 — Supplementary file2 (ZIP 3179 KB) [file 10238_2024_1372_MOESM2_ESM.zip › Supplementary Material/Drug1/drugSenstivity.AZD6738.pdf]

AZD7762 sensitivity(IC50)

risk low high

$7.1\text{e-}15$

15

10

5

0

low

high

risk

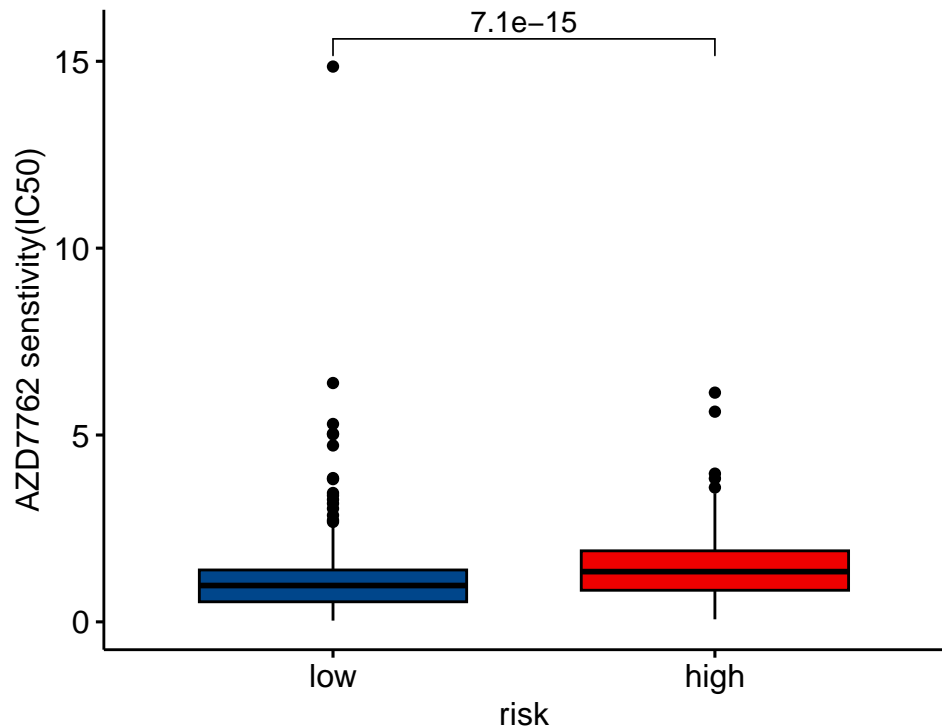

Supplement: Supplementary file 2 — Supplementary file2 (ZIP 3179 KB) [file 10238_2024_1372_MOESM2_ESM.zip › Supplementary Material/Drug1/drugSenstivity.AZD7762.pdf]

AZD8055 sensitivity(IC50)

risk low high

$p < 2.22e-16$

low

high

risk

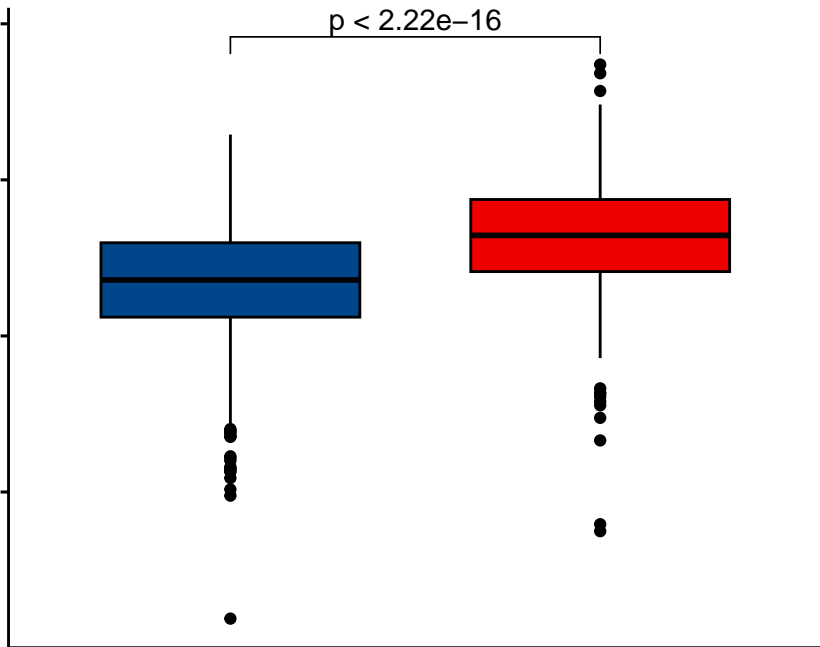

Supplement: Supplementary file 2 — Supplementary file2 (ZIP 3179 KB) [file 10238_2024_1372_MOESM2_ESM.zip › Supplementary Material/Drug1/drugSenstivity.AZD8055.pdf]

risk low high

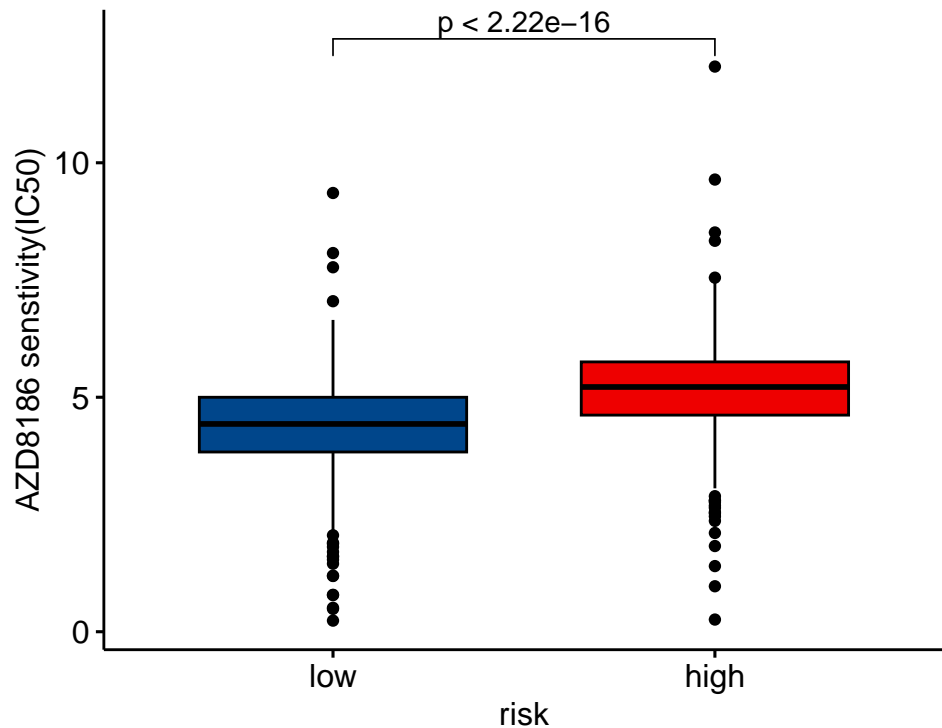

Supplement: Supplementary file 2 — Supplementary file2 (ZIP 3179 KB) [file 10238_2024_1372_MOESM2_ESM.zip › Supplementary Material/Drug1/drugSenstivity.AZD8186.pdf]

risk low high

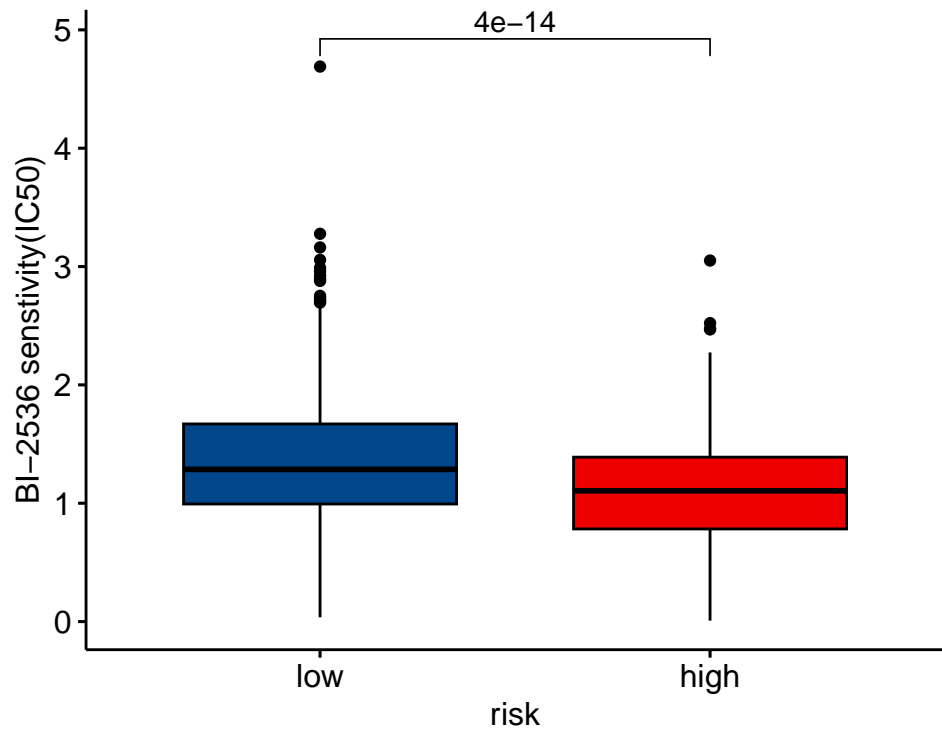

Supplement: Supplementary file 2 — Supplementary file2 (ZIP 3179 KB) [file 10238_2024_1372_MOESM2_ESM.zip › Supplementary Material/Drug1/drugSenstivity.BI-2536.pdf]

BMS-536924 sensitivity(IC50)

risk 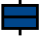 low 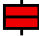 high

$p < 2.22e-16$

low

high

risk

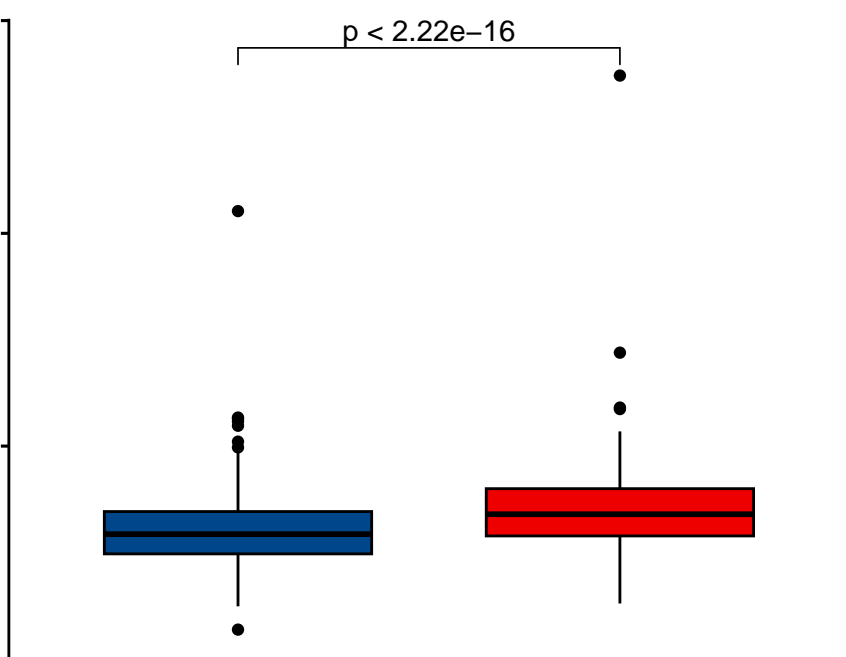

Supplement: Supplementary file 2 — Supplementary file2 (ZIP 3179 KB) [file 10238_2024_1372_MOESM2_ESM.zip › Supplementary Material/Drug1/drugSenstivity.BMS-536924.pdf]

risk 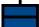 low 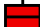 high

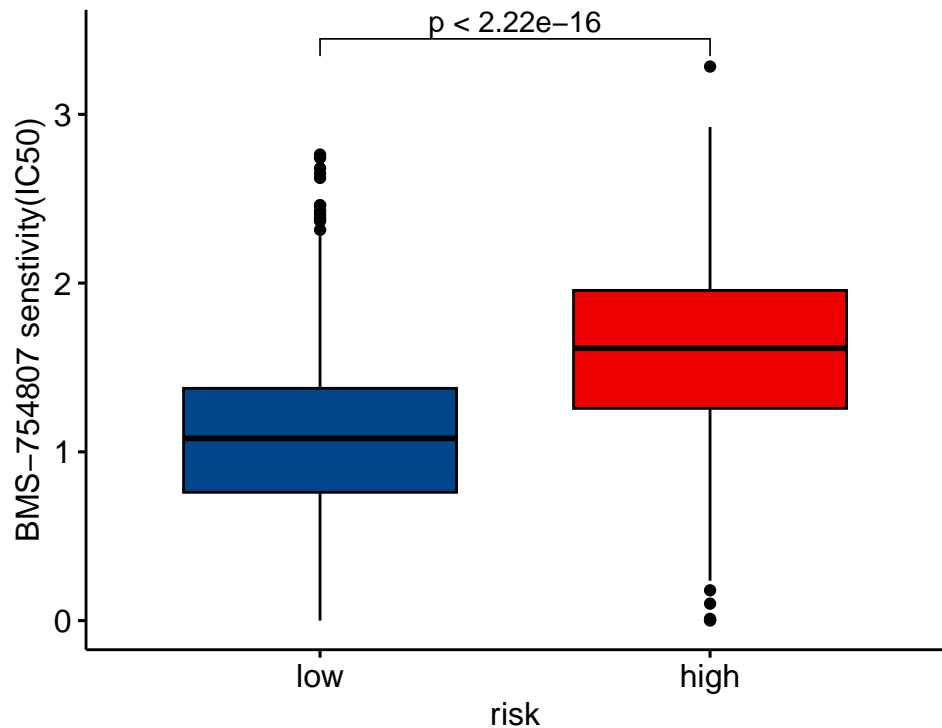

Supplement: Supplementary file 2 — Supplementary file2 (ZIP 3179 KB) [file 10238_2024_1372_MOESM2_ESM.zip › Supplementary Material/Drug1/drugSenstivity.BMS-754807.pdf]

risk low high

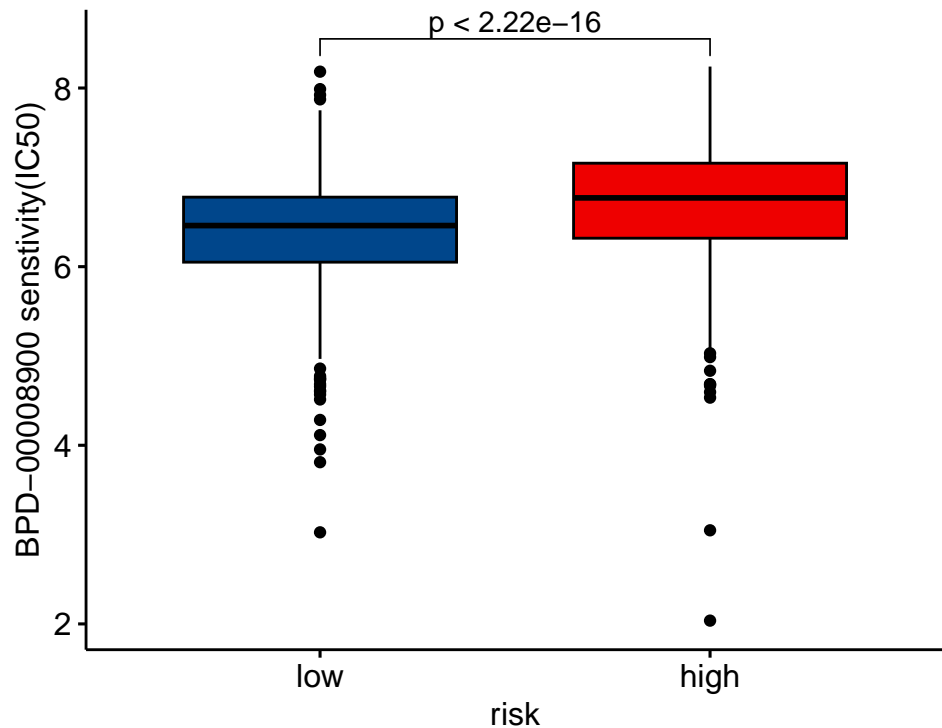

Supplement: Supplementary file 2 — Supplementary file2 (ZIP 3179 KB) [file 10238_2024_1372_MOESM2_ESM.zip › Supplementary Material/Drug1/drugSenstivity.BPD-00008900.pdf]

risk low high

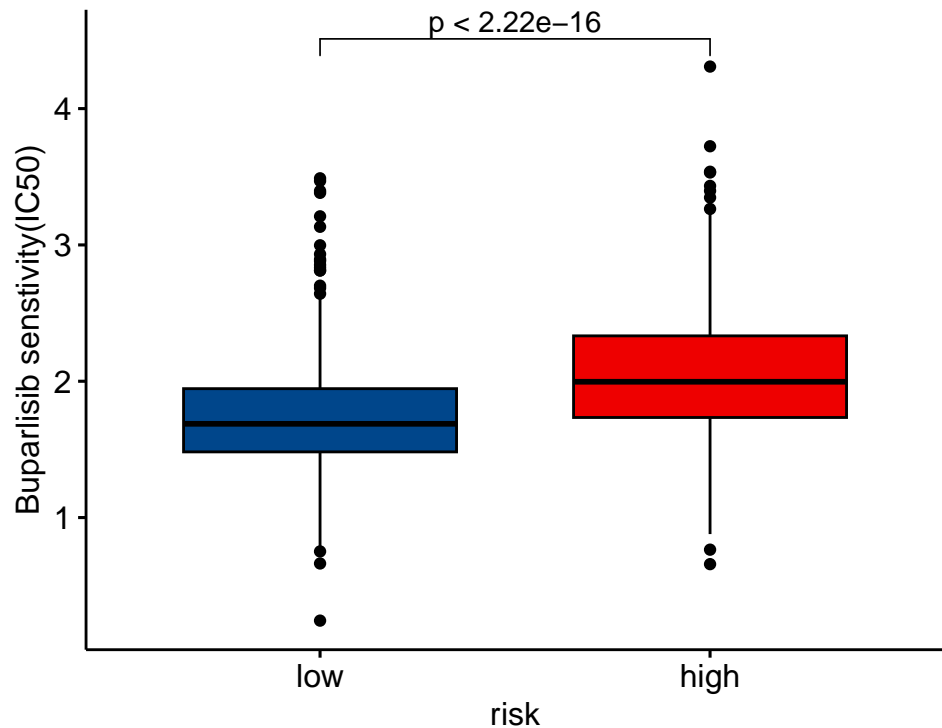

Supplement: Supplementary file 2 — Supplementary file2 (ZIP 3179 KB) [file 10238_2024_1372_MOESM2_ESM.zip › Supplementary Material/Drug1/drugSenstivity.Buparlisib.pdf]

risk low high

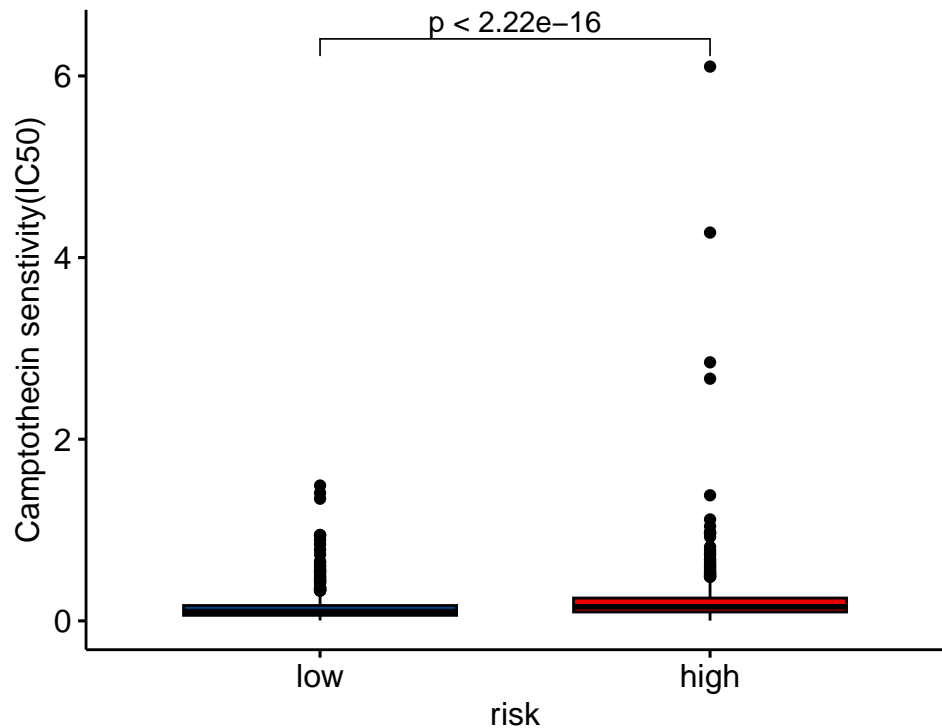

Supplement: Supplementary file 2 — Supplementary file2 (ZIP 3179 KB) [file 10238_2024_1372_MOESM2_ESM.zip › Supplementary Material/Drug1/drugSenstivity.Camptothecin.pdf]

risk low high

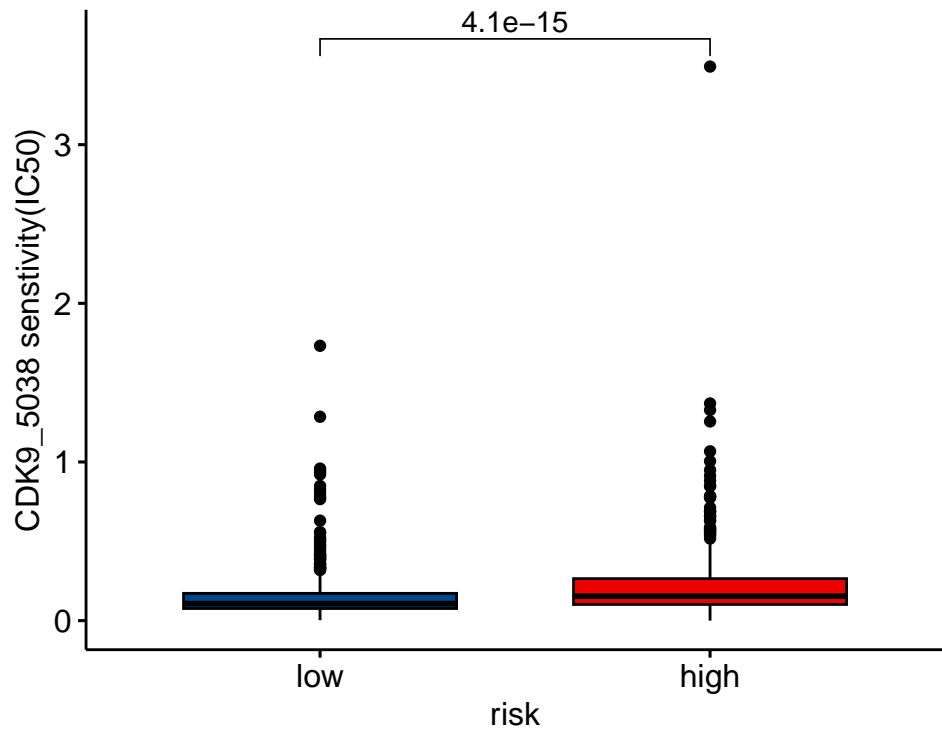

Supplement: Supplementary file 2 — Supplementary file2 (ZIP 3179 KB) [file 10238_2024_1372_MOESM2_ESM.zip › Supplementary Material/Drug1/drugSenstivity.CDK9_5038.pdf]

risk low high

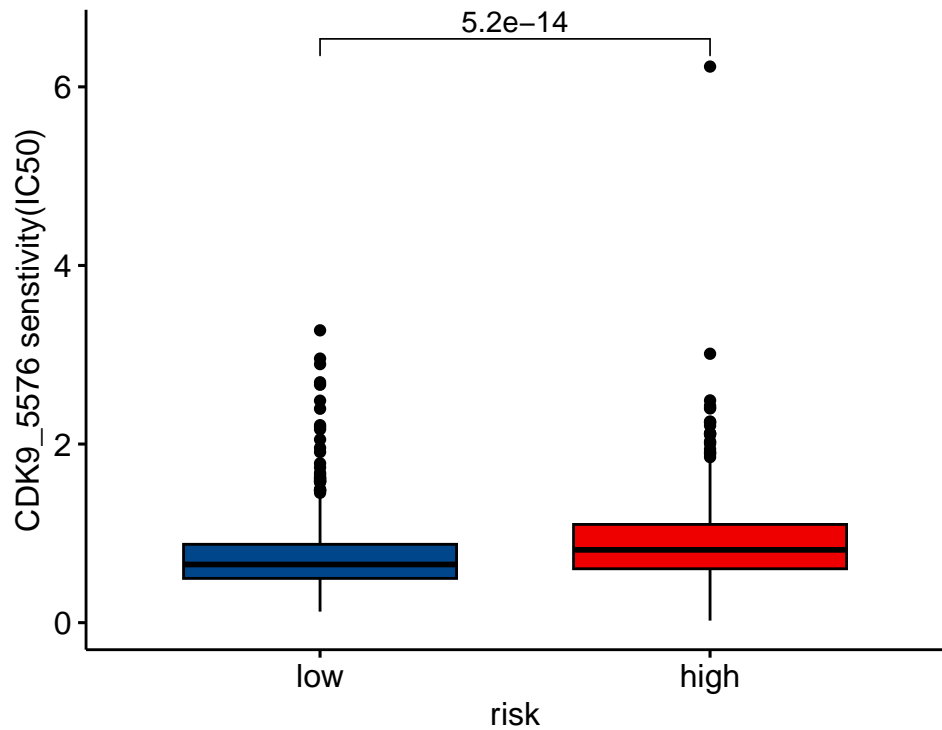

Supplement: Supplementary file 2 — Supplementary file2 (ZIP 3179 KB) [file 10238_2024_1372_MOESM2_ESM.zip › Supplementary Material/Drug1/drugSenstivity.CDK9_5576.pdf]

Cediranib sensitivity(IC50)

risk low high

1.4e-11

low

high

risk

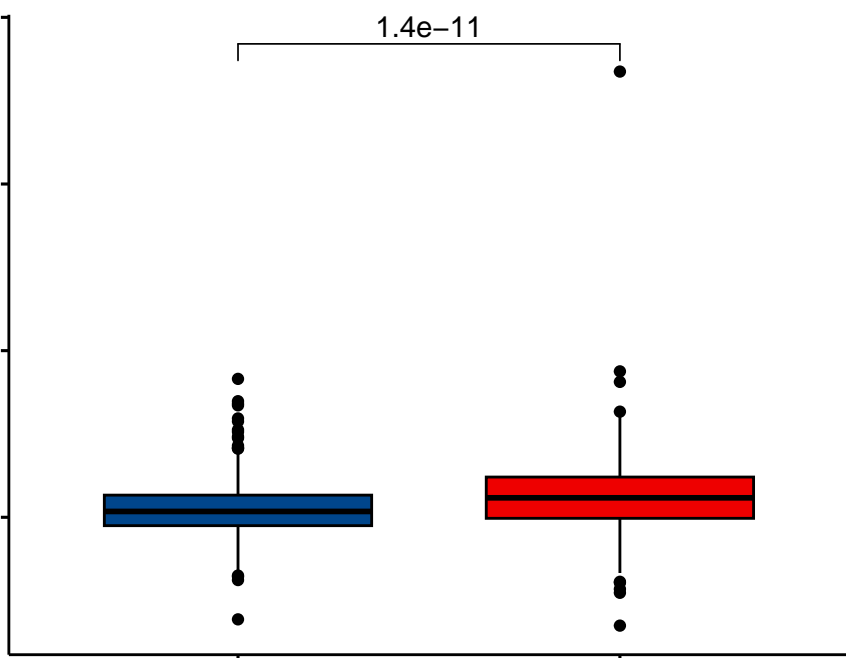

Supplement: Supplementary file 2 — Supplementary file2 (ZIP 3179 KB) [file 10238_2024_1372_MOESM2_ESM.zip › Supplementary Material/Drug1/drugSenstivity.Cediranib.pdf]

risk low high

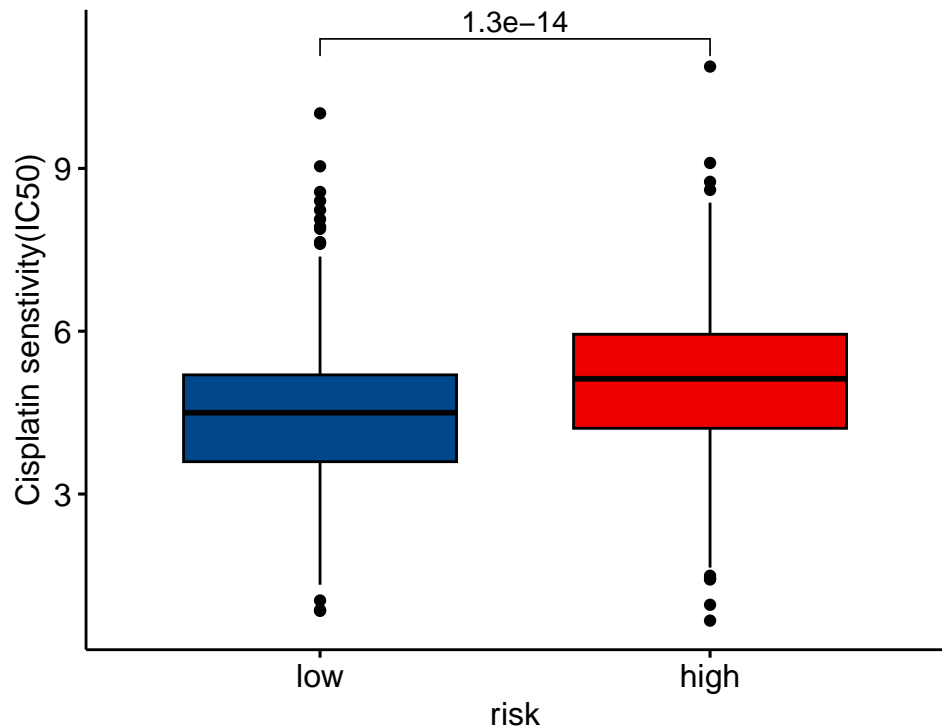

Supplement: Supplementary file 2 — Supplementary file2 (ZIP 3179 KB) [file 10238_2024_1372_MOESM2_ESM.zip › Supplementary Material/Drug1/drugSenstivity.Cisplatin.pdf]

risk low high

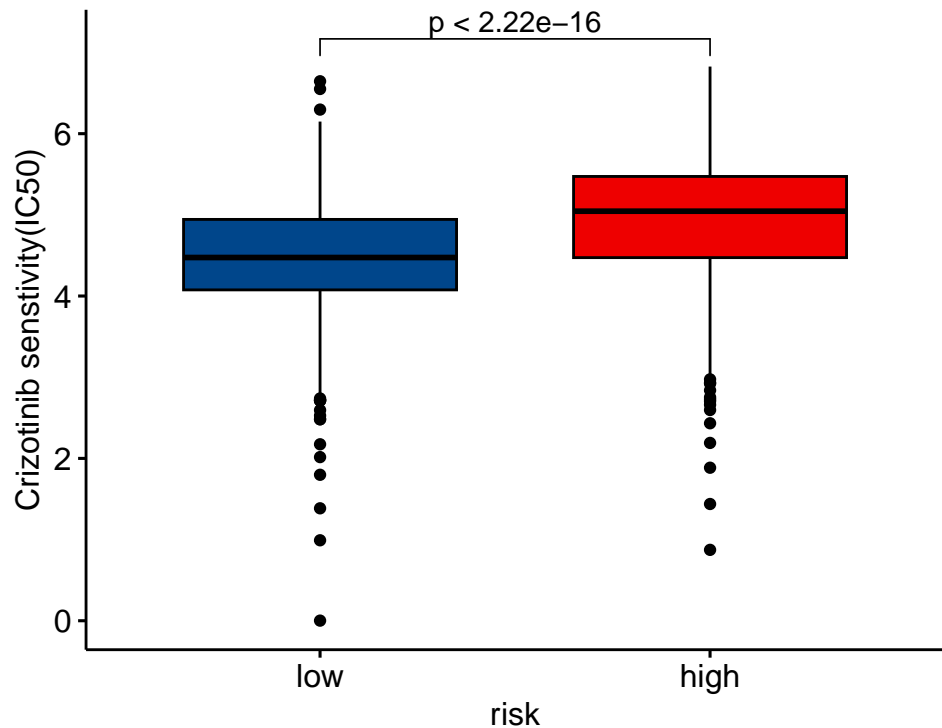

Supplement: Supplementary file 2 — Supplementary file2 (ZIP 3179 KB) [file 10238_2024_1372_MOESM2_ESM.zip › Supplementary Material/Drug1/drugSenstivity.Crizotinib.pdf]

risk low high

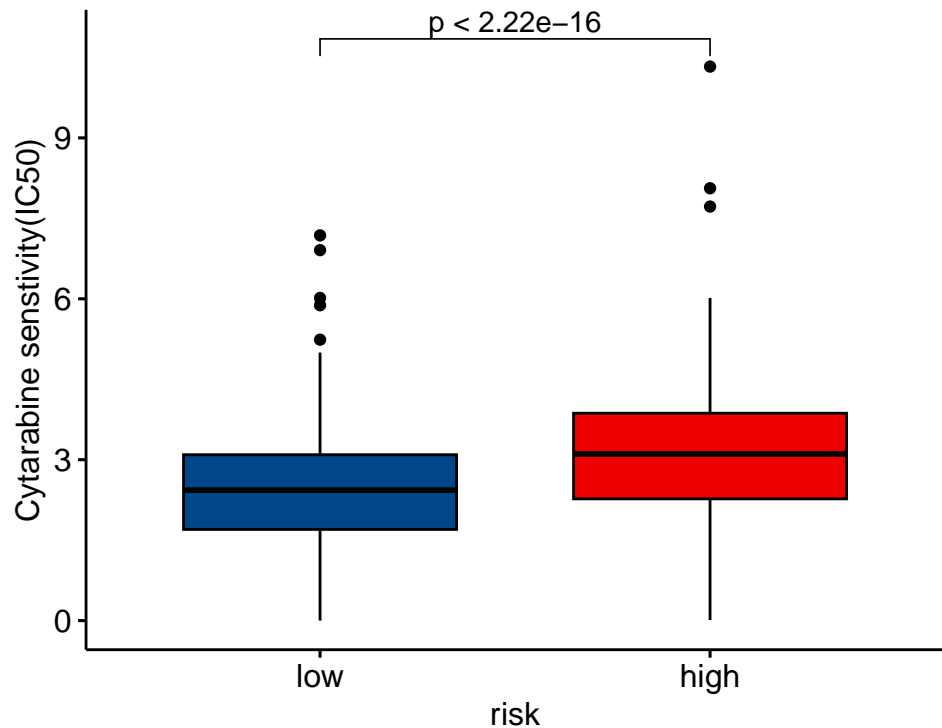

Supplement: Supplementary file 2 — Supplementary file2 (ZIP 3179 KB) [file 10238_2024_1372_MOESM2_ESM.zip › Supplementary Material/Drug1/drugSenstivity.Cytarabine.pdf]

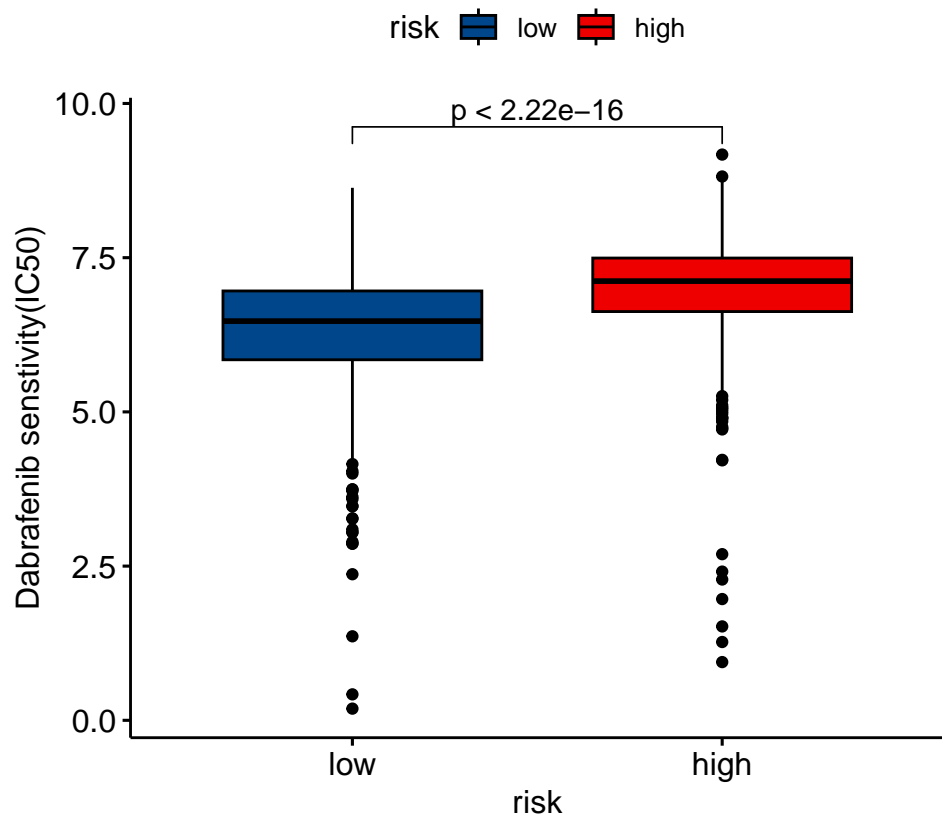

Supplement: Supplementary file 2 — Supplementary file2 (ZIP 3179 KB) [file 10238_2024_1372_MOESM2_ESM.zip › Supplementary Material/Drug1/drugSenstivity.Dabrafenib.pdf]

risk low high

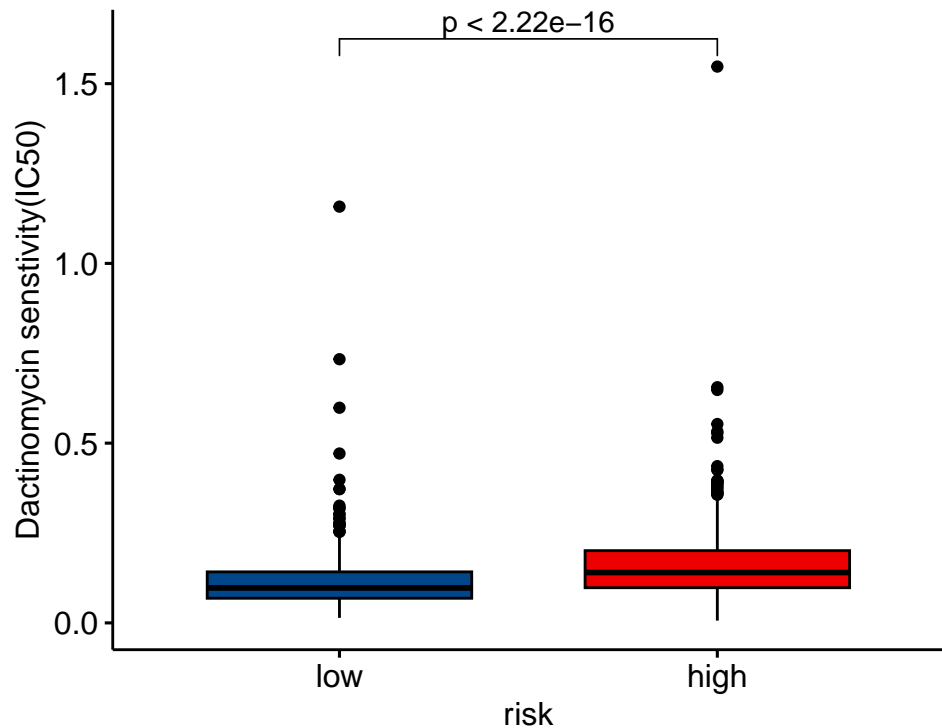

Supplement: Supplementary file 2 — Supplementary file2 (ZIP 3179 KB) [file 10238_2024_1372_MOESM2_ESM.zip › Supplementary Material/Drug1/drugSenstivity.Dactinomycin.pdf]

risk low high

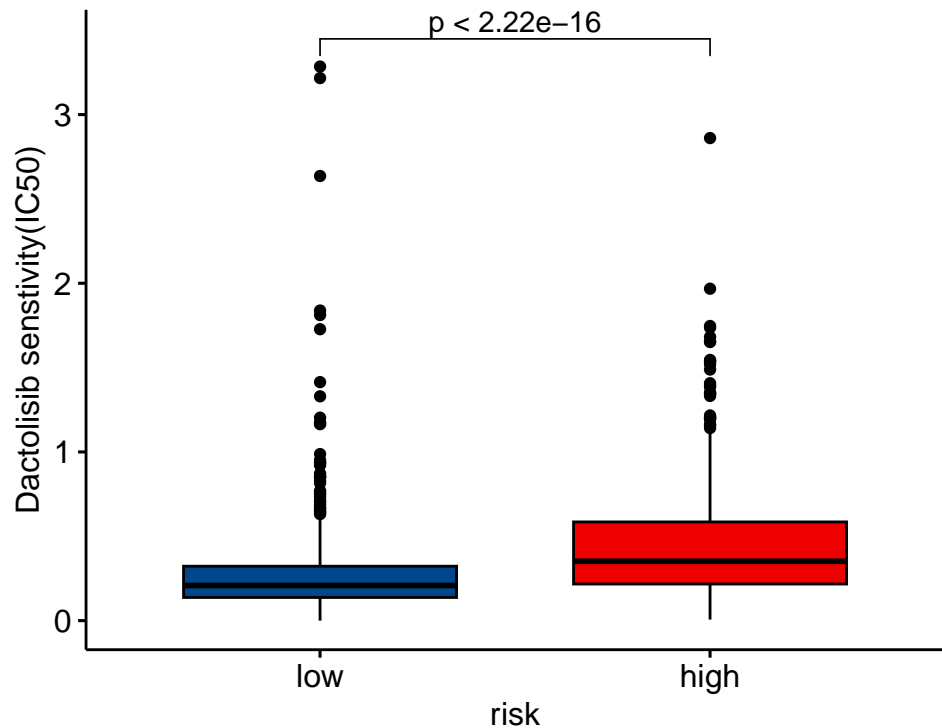

Supplement: Supplementary file 2 — Supplementary file2 (ZIP 3179 KB) [file 10238_2024_1372_MOESM2_ESM.zip › Supplementary Material/Drug1/drugSenstivity.Dactolisib.pdf]

Dasatinib sensitivity(IC50)

risk low high

$p < 2.22e-16$

10.0

7.5

5.0

2.5

0.0

low

high

risk

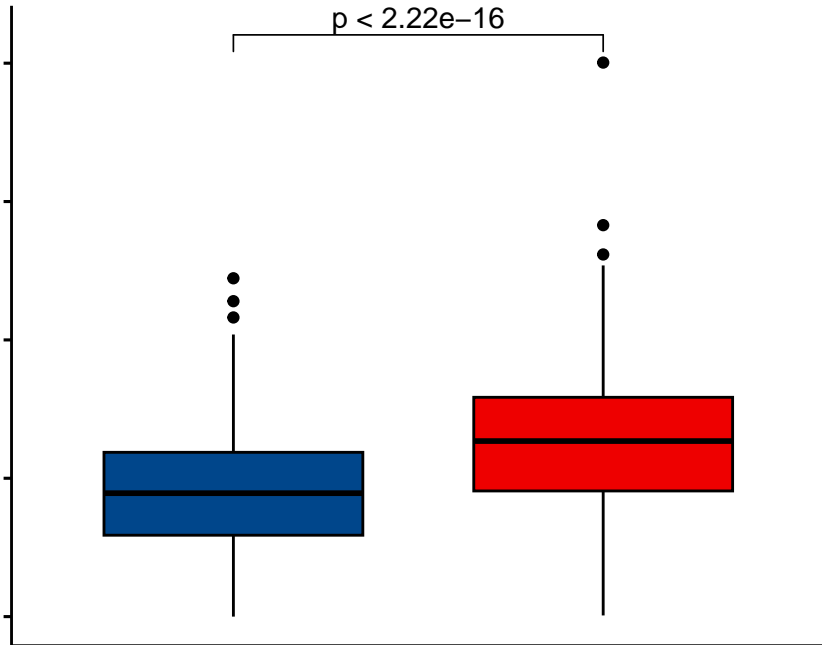

Supplement: Supplementary file 2 — Supplementary file2 (ZIP 3179 KB) [file 10238_2024_1372_MOESM2_ESM.zip › Supplementary Material/Drug1/drugSenstivity.Dasatinib.pdf]

risk low high

$p < 2.22e-16$

Docetaxel sensitivity(IC50)

0.3

0.2

0.1

0.0

low

high

risk

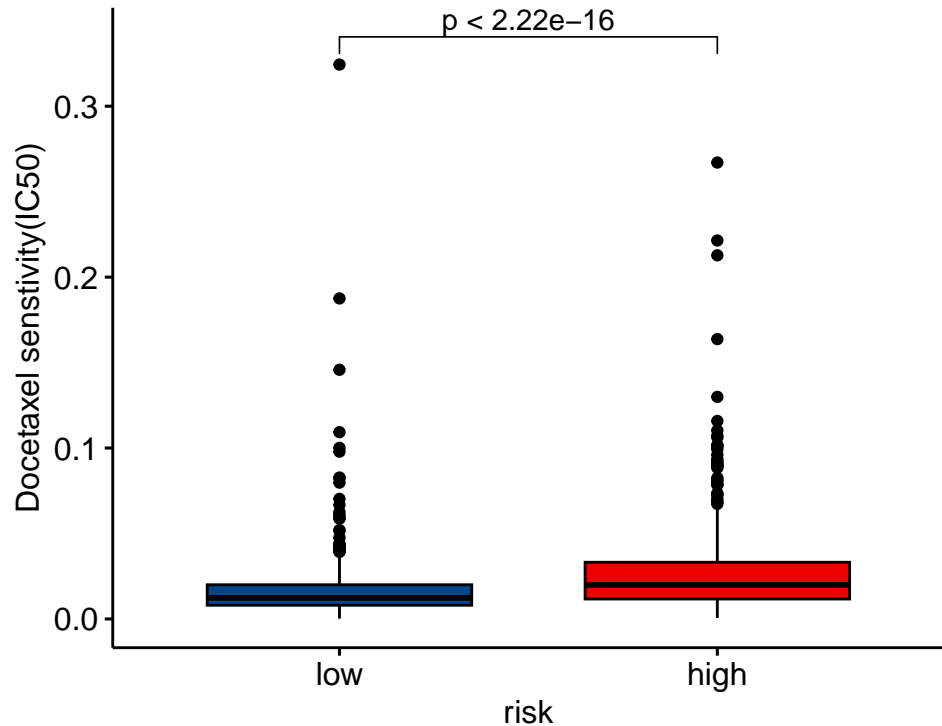

Supplement: Supplementary file 2 — Supplementary file2 (ZIP 3179 KB) [file 10238_2024_1372_MOESM2_ESM.zip › Supplementary Material/Drug1/drugSenstivity.Docetaxel.pdf]

risk low high

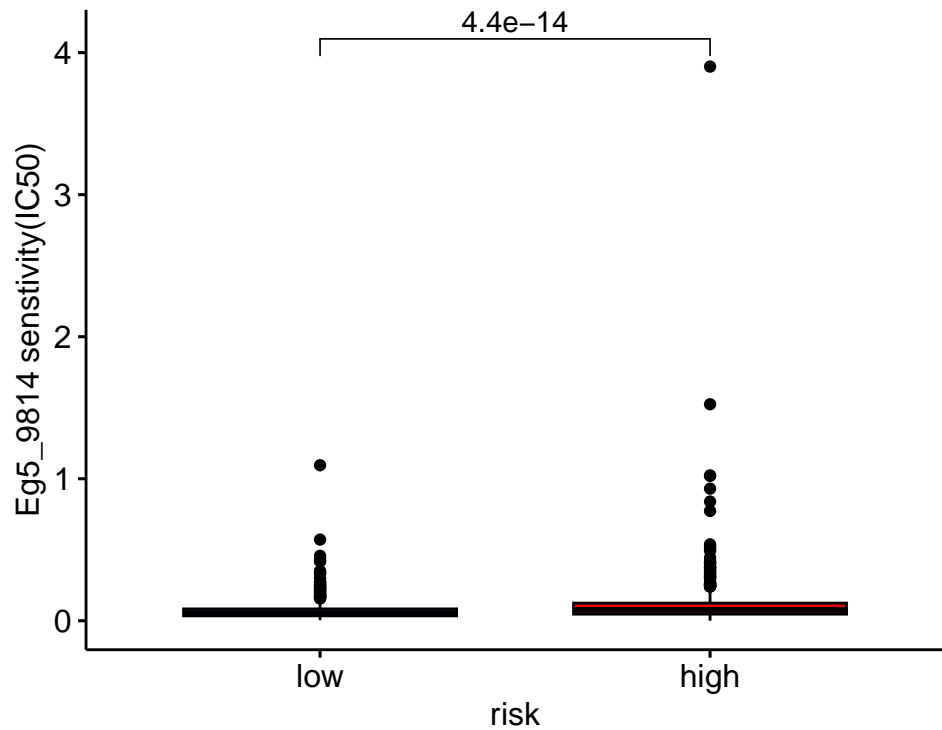

Supplement: Supplementary file 2 — Supplementary file2 (ZIP 3179 KB) [file 10238_2024_1372_MOESM2_ESM.zip › Supplementary Material/Drug1/drugSenstivity.Eg5_9814.pdf]

risk low high

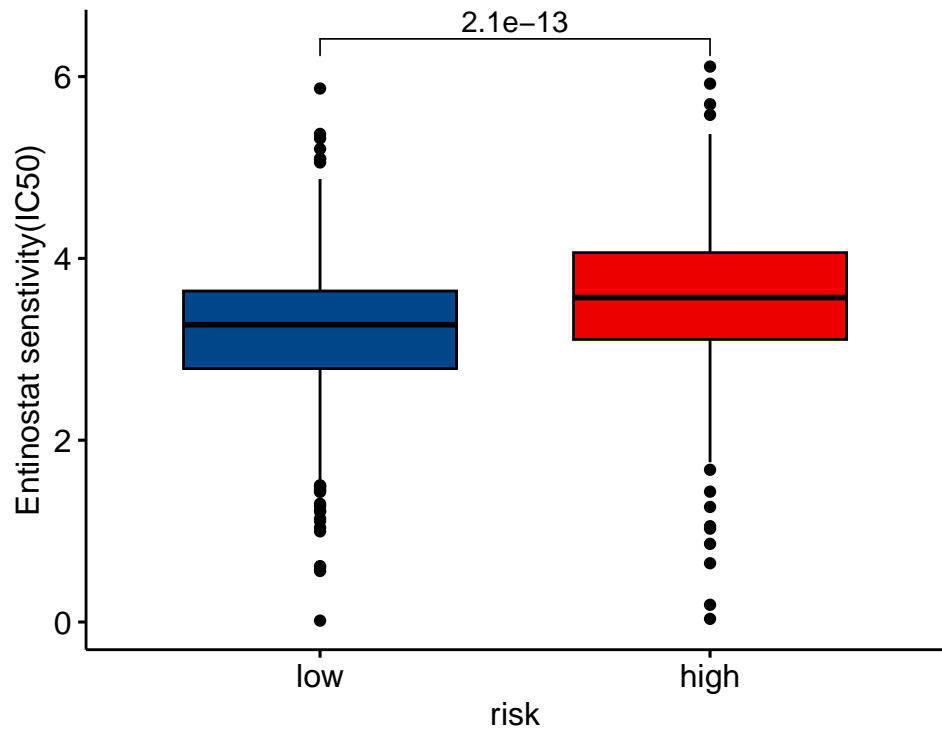

Supplement: Supplementary file 2 — Supplementary file2 (ZIP 3179 KB) [file 10238_2024_1372_MOESM2_ESM.zip › Supplementary Material/Drug1/drugSenstivity.Entinostat.pdf]

risk low high

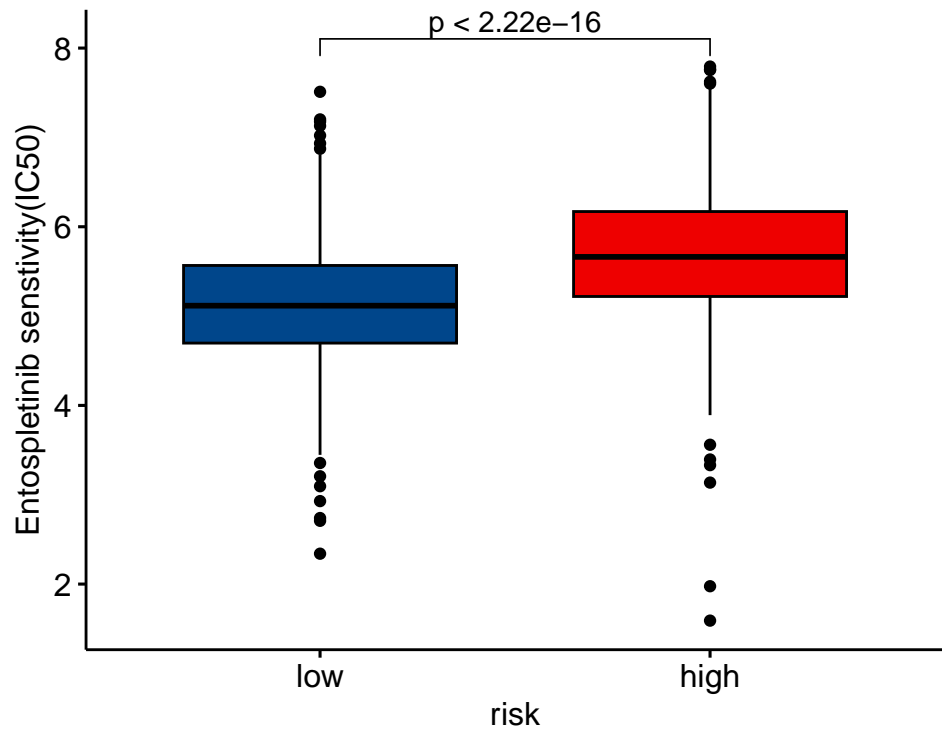

Supplement: Supplementary file 2 — Supplementary file2 (ZIP 3179 KB) [file 10238_2024_1372_MOESM2_ESM.zip › Supplementary Material/Drug1/drugSenstivity.Entospletinib.pdf]

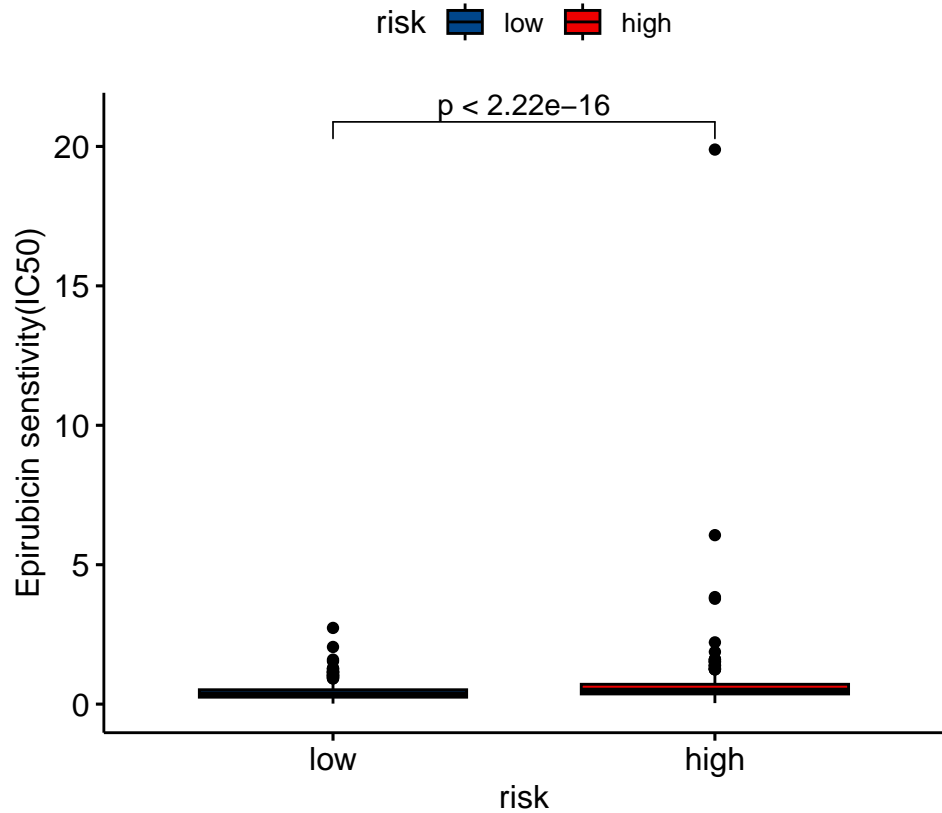

Supplement: Supplementary file 2 — Supplementary file2 (ZIP 3179 KB) [file 10238_2024_1372_MOESM2_ESM.zip › Supplementary Material/Drug1/drugSenstivity.Epirubicin.pdf]

EPZ004777 sensitivity(IC50)

risk low high

5.6e-16

low

high

risk

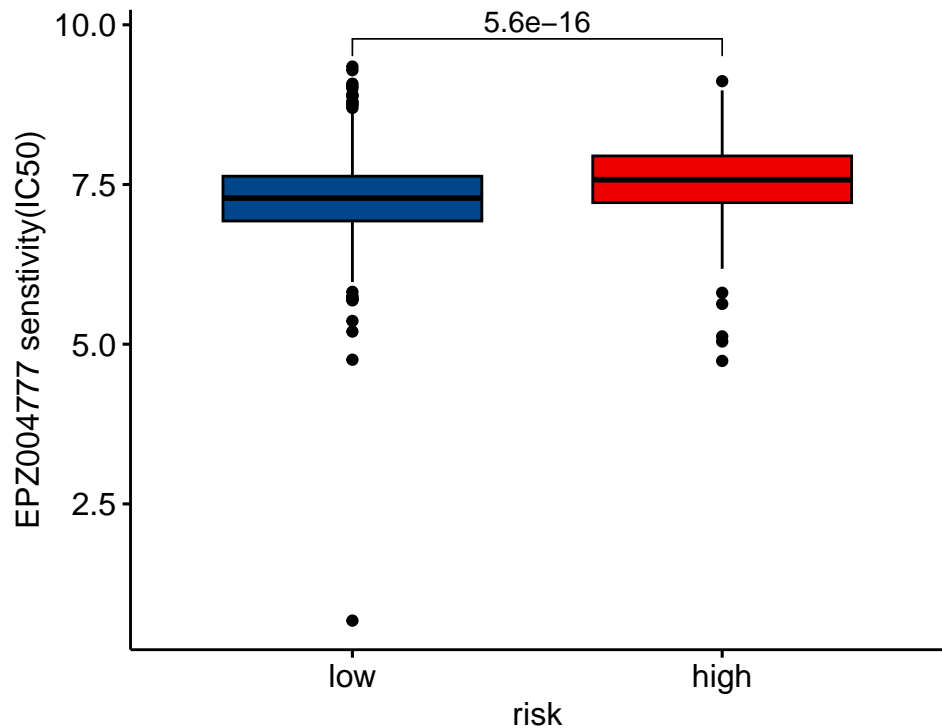

Supplement: Supplementary file 2 — Supplementary file2 (ZIP 3179 KB) [file 10238_2024_1372_MOESM2_ESM.zip › Supplementary Material/Drug1/drugSenstivity.EPZ004777.pdf]

risk low high

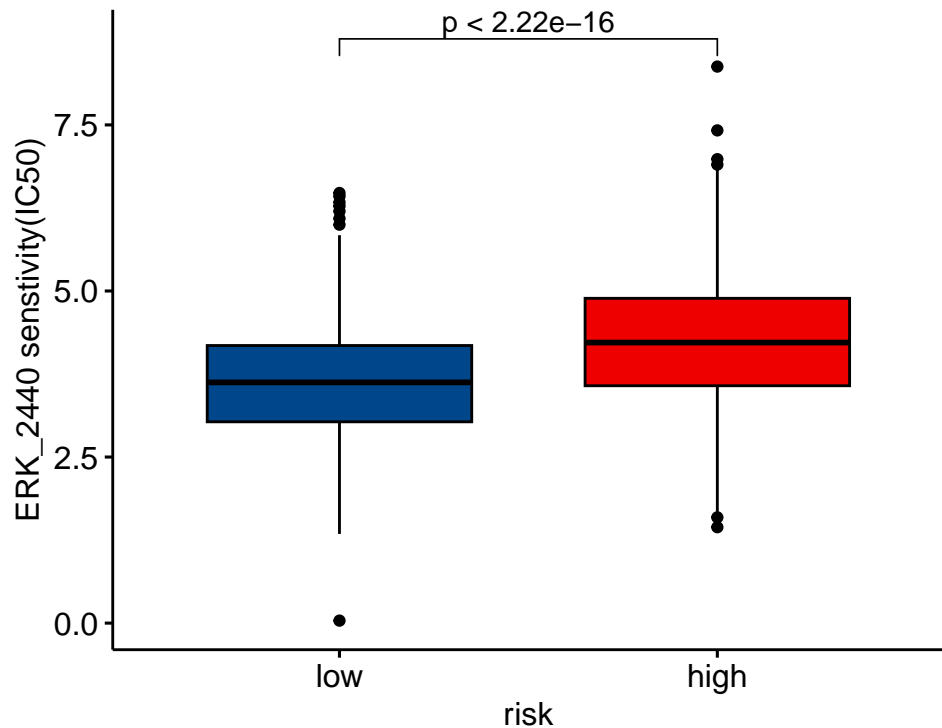

Supplement: Supplementary file 2 — Supplementary file2 (ZIP 3179 KB) [file 10238_2024_1372_MOESM2_ESM.zip › Supplementary Material/Drug1/drugSenstivity.ERK_2440.pdf]

risk low high

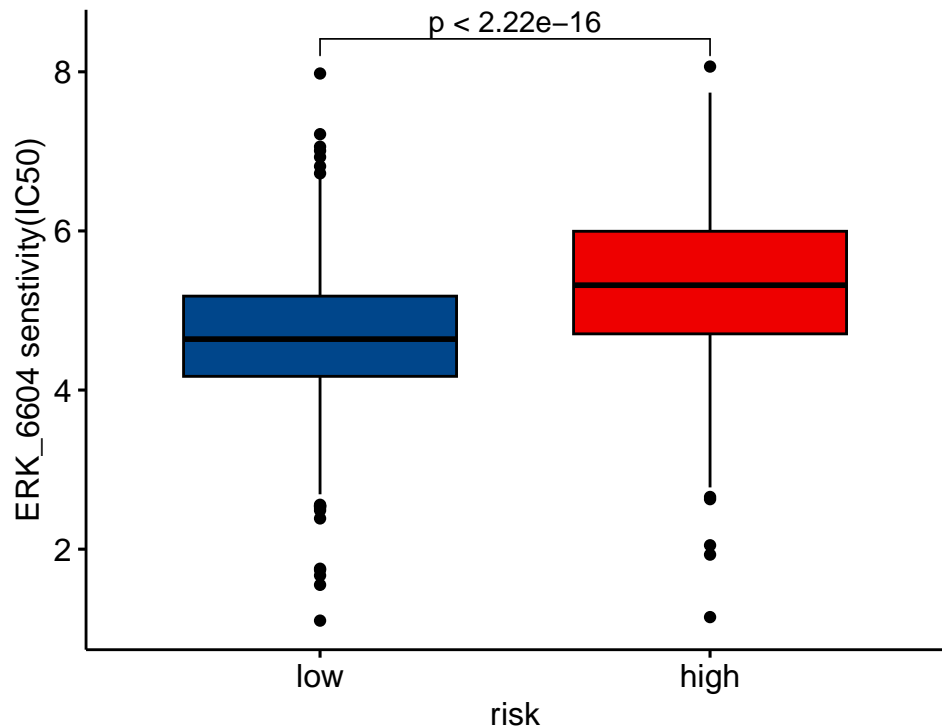

Supplement: Supplementary file 2 — Supplementary file2 (ZIP 3179 KB) [file 10238_2024_1372_MOESM2_ESM.zip › Supplementary Material/Drug1/drugSenstivity.ERK_6604.pdf]

risk low high

$p < 2.22e-16$

Fludarabine sensitivity(IC50)

low

high

risk

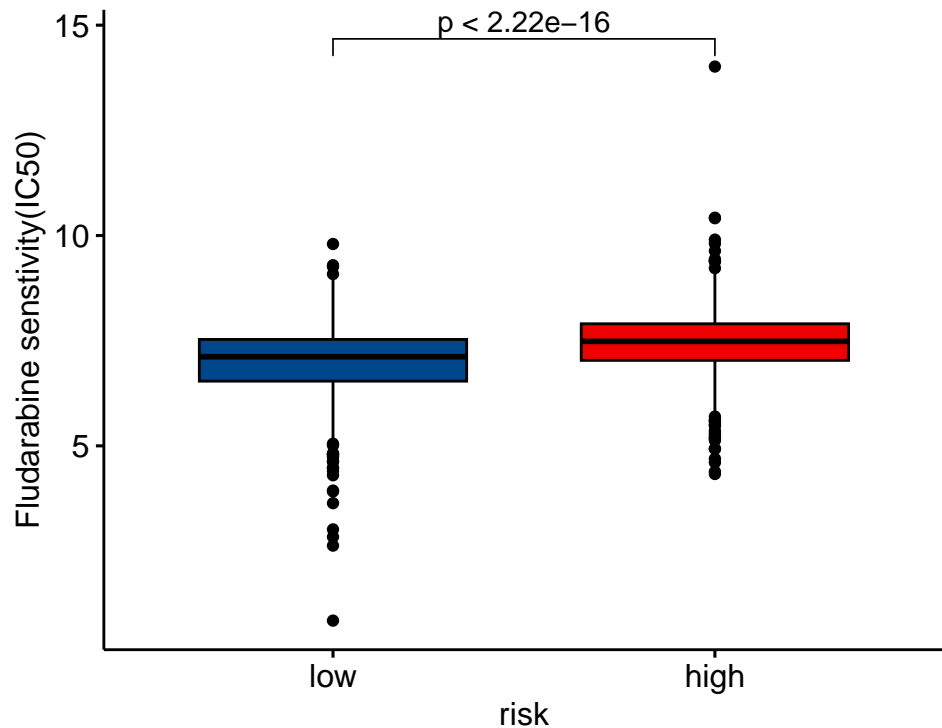

Supplement: Supplementary file 2 — Supplementary file2 (ZIP 3179 KB) [file 10238_2024_1372_MOESM2_ESM.zip › Supplementary Material/Drug1/drugSenstivity.Fludarabine.pdf]

risk 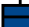 low 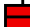 high

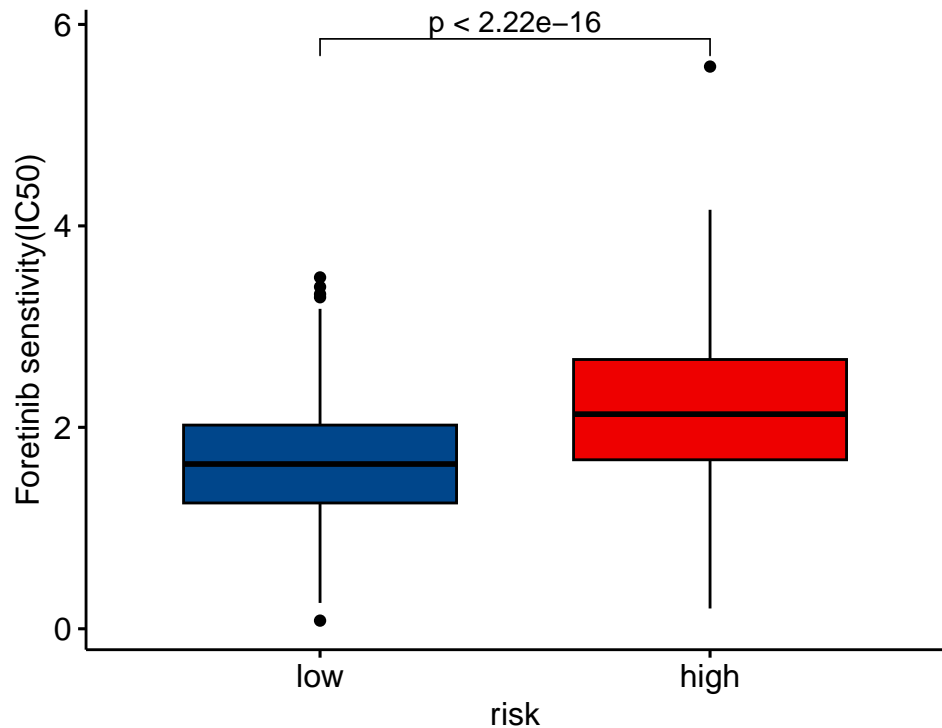

Supplement: Supplementary file 2 — Supplementary file2 (ZIP 3179 KB) [file 10238_2024_1372_MOESM2_ESM.zip › Supplementary Material/Drug1/drugSenstivity.Foretinib.pdf]

risk low high

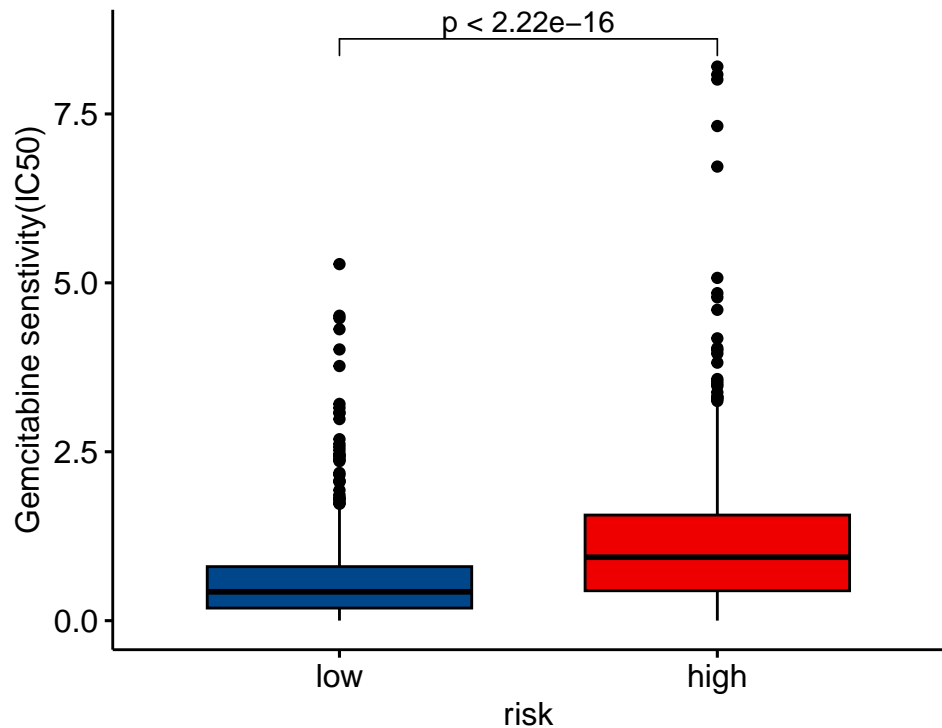

Supplement: Supplementary file 2 — Supplementary file2 (ZIP 3179 KB) [file 10238_2024_1372_MOESM2_ESM.zip › Supplementary Material/Drug1/drugSenstivity.Gemcitabine.pdf]

risk 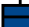 low 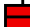 high

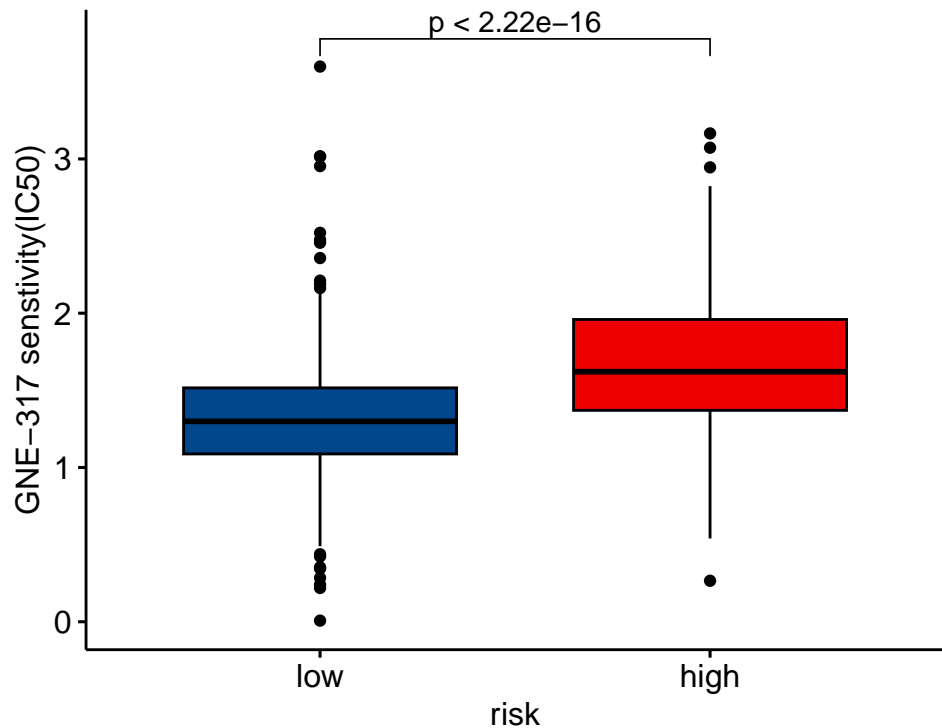

Supplement: Supplementary file 2 — Supplementary file2 (ZIP 3179 KB) [file 10238_2024_1372_MOESM2_ESM.zip › Supplementary Material/Drug1/drugSenstivity.GNE-317.pdf]

risk low high

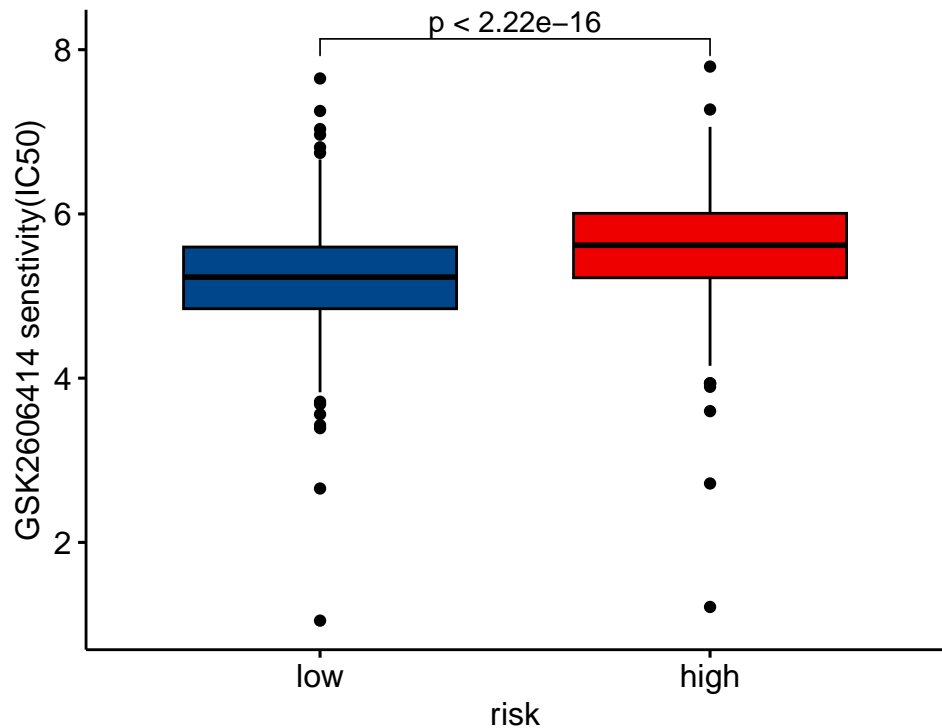

Supplement: Supplementary file 2 — Supplementary file2 (ZIP 3179 KB) [file 10238_2024_1372_MOESM2_ESM.zip › Supplementary Material/Drug1/drugSenstivity.GSK2606414.pdf]

GSK269962A sensitivity(IC50)

risk low high

$p < 2.22e-16$

low

high

risk

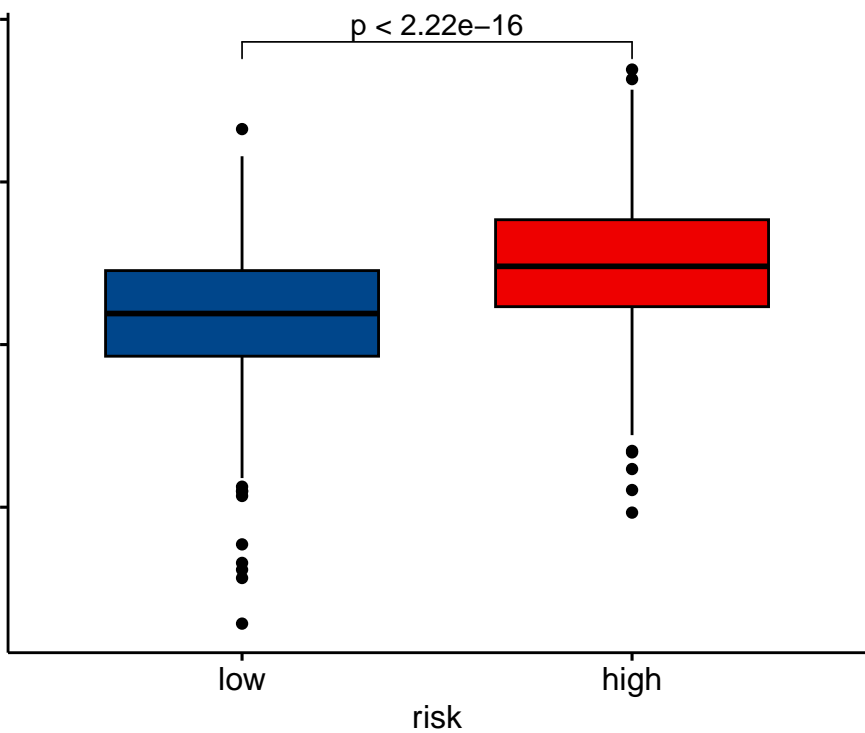

Supplement: Supplementary file 2 — Supplementary file2 (ZIP 3179 KB) [file 10238_2024_1372_MOESM2_ESM.zip › Supplementary Material/Drug1/drugSenstivity.GSK269962A.pdf]

risk low high

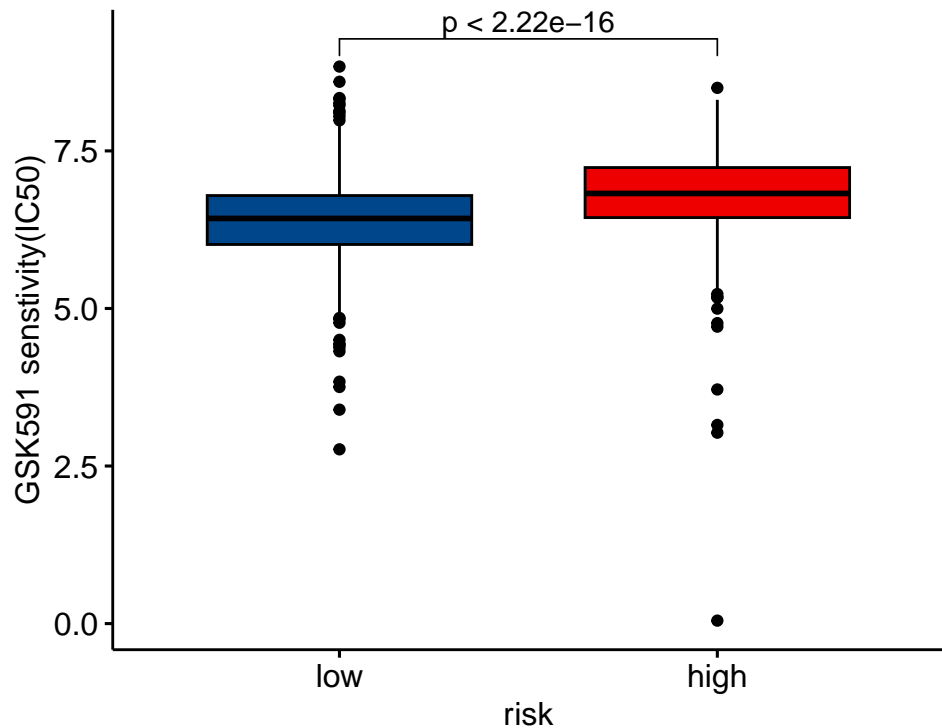

Supplement: Supplementary file 2 — Supplementary file2 (ZIP 3179 KB) [file 10238_2024_1372_MOESM2_ESM.zip › Supplementary Material/Drug1/drugSenstivity.GSK591.pdf]

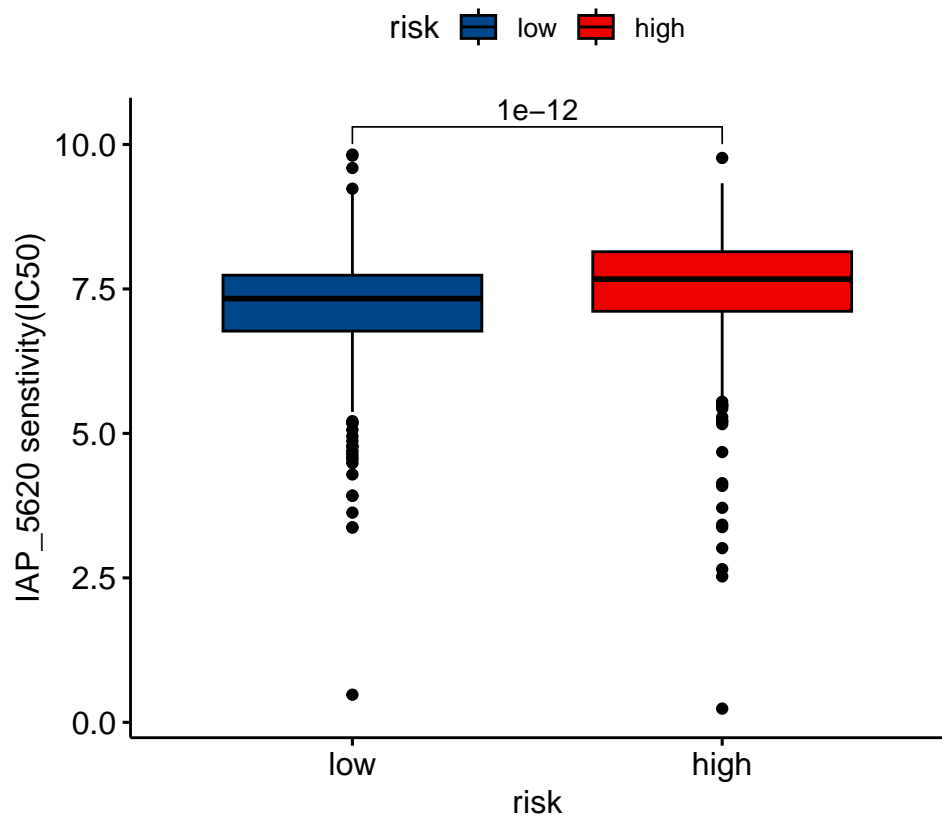

Supplement: Supplementary file 2 — Supplementary file2 (ZIP 3179 KB) [file 10238_2024_1372_MOESM2_ESM.zip › Supplementary Material/Drug1/drugSenstivity.IAP_5620.pdf]

risk low high

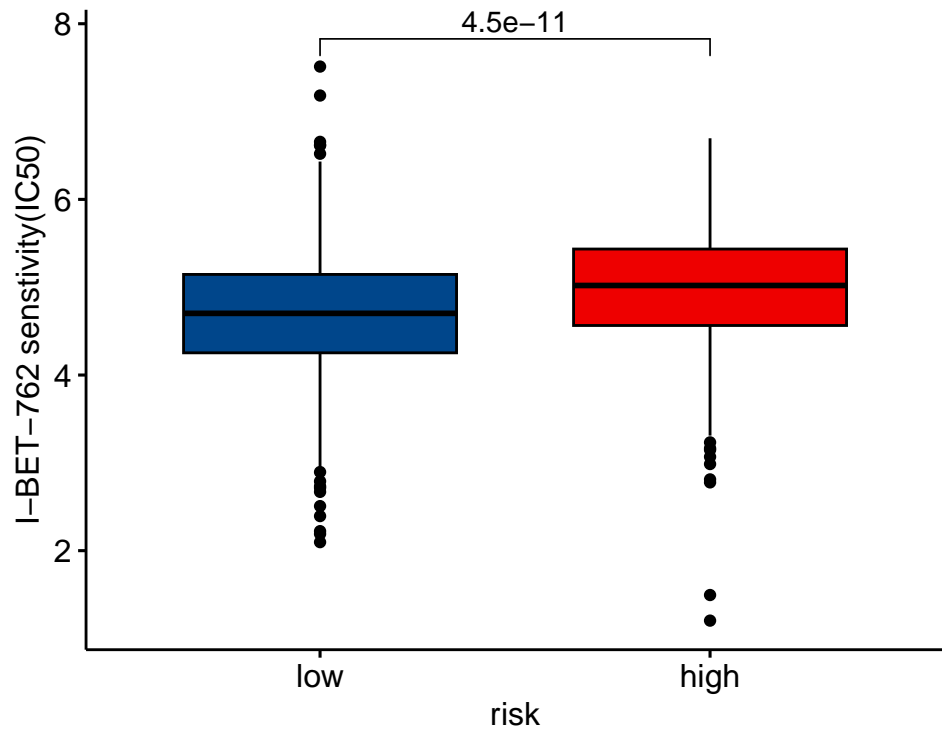

Supplement: Supplementary file 2 — Supplementary file2 (ZIP 3179 KB) [file 10238_2024_1372_MOESM2_ESM.zip › Supplementary Material/Drug1/drugSenstivity.I-BET-762.pdf]

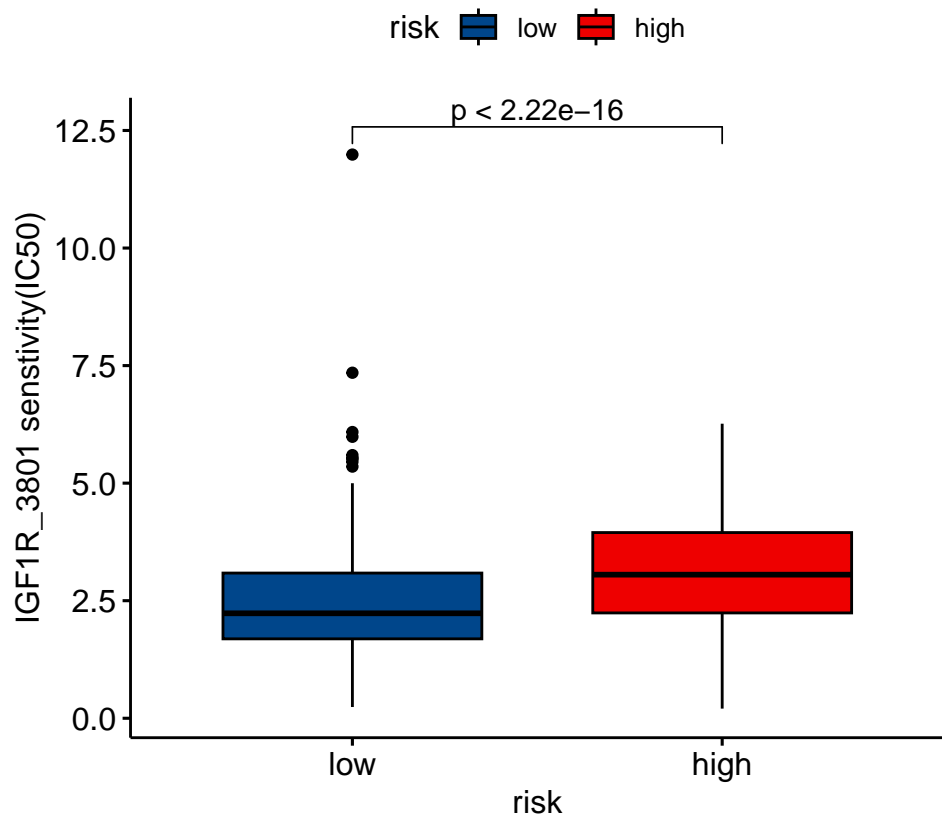

Supplement: Supplementary file 2 — Supplementary file2 (ZIP 3179 KB) [file 10238_2024_1372_MOESM2_ESM.zip › Supplementary Material/Drug1/drugSenstivity.IGF1R_3801.pdf]

risk low high

$p < 2.22e-16$

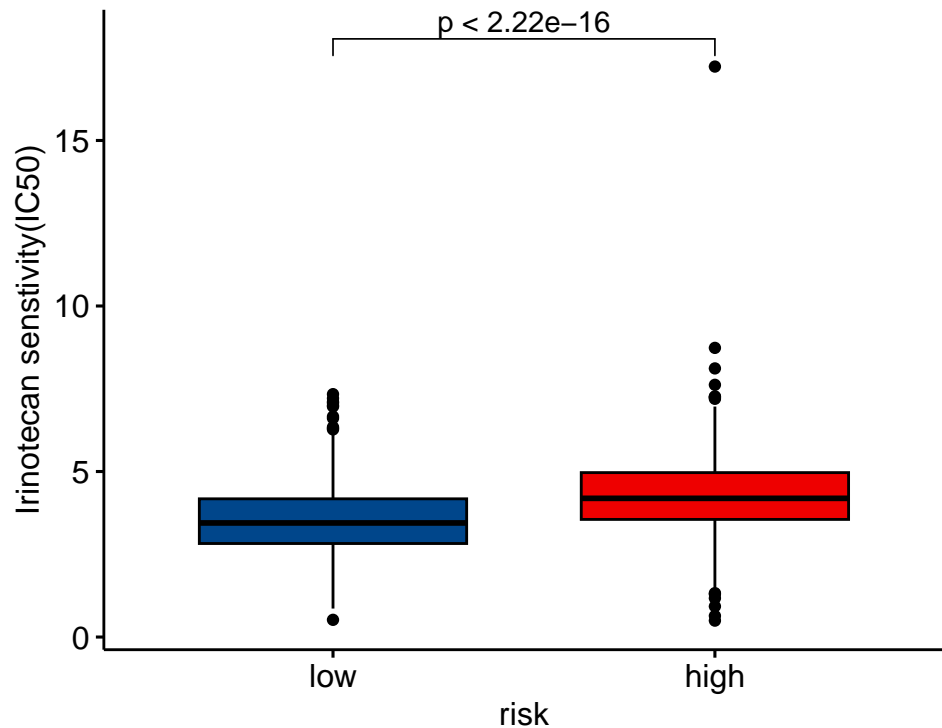

Supplement: Supplementary file 2 — Supplementary file2 (ZIP 3179 KB) [file 10238_2024_1372_MOESM2_ESM.zip › Supplementary Material/Drug1/drugSenstivity.Irinotecan.pdf]

risk low high

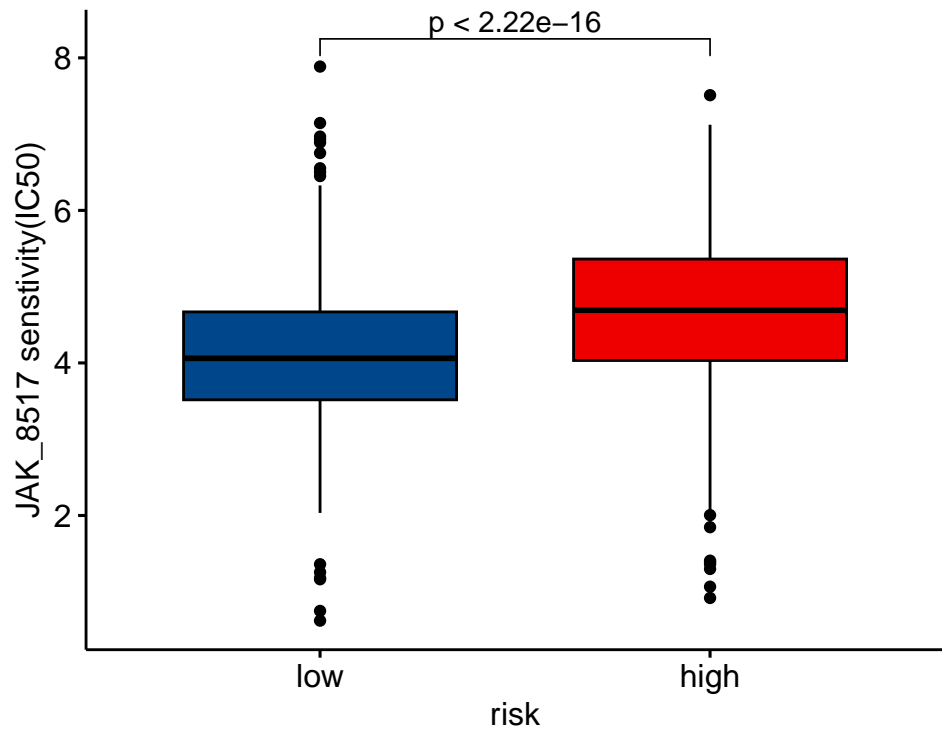

Supplement: Supplementary file 2 — Supplementary file2 (ZIP 3179 KB) [file 10238_2024_1372_MOESM2_ESM.zip › Supplementary Material/Drug1/drugSenstivity.JAK_8517.pdf]

risk low high

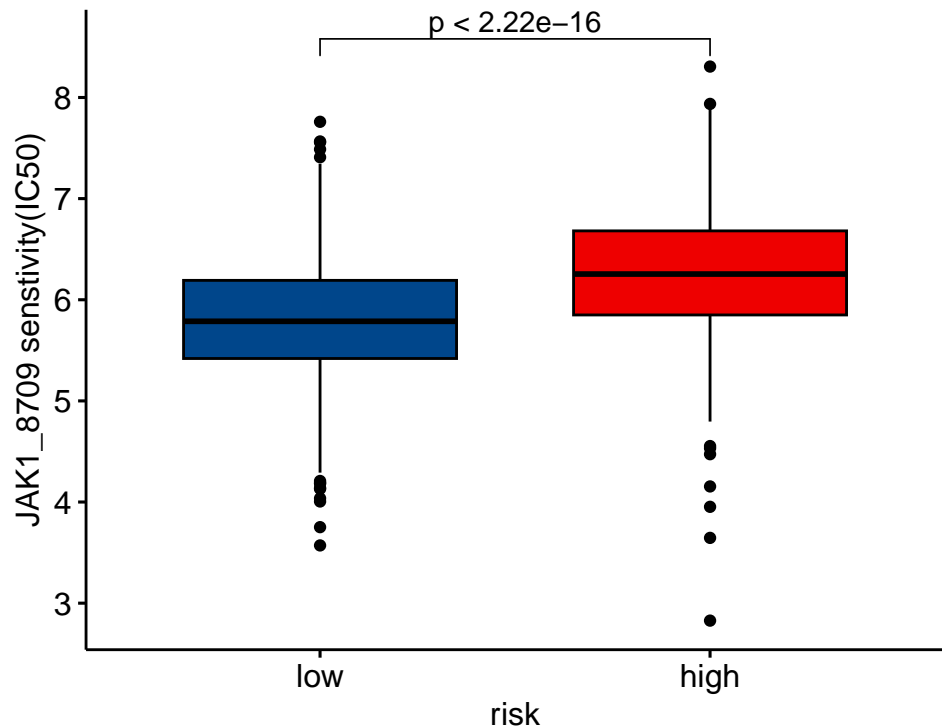

Supplement: Supplementary file 2 — Supplementary file2 (ZIP 3179 KB) [file 10238_2024_1372_MOESM2_ESM.zip › Supplementary Material/Drug1/drugSenstivity.JAK1_8709.pdf]

risk low high

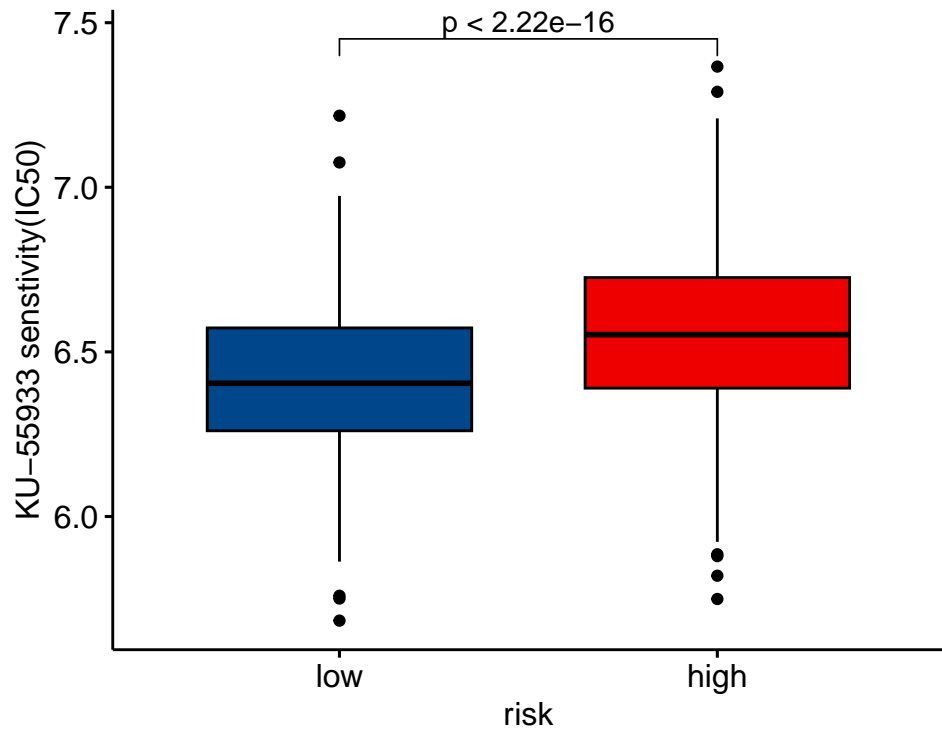

Supplement: Supplementary file 2 — Supplementary file2 (ZIP 3179 KB) [file 10238_2024_1372_MOESM2_ESM.zip › Supplementary Material/Drug1/drugSenstivity.KU-55933.pdf]

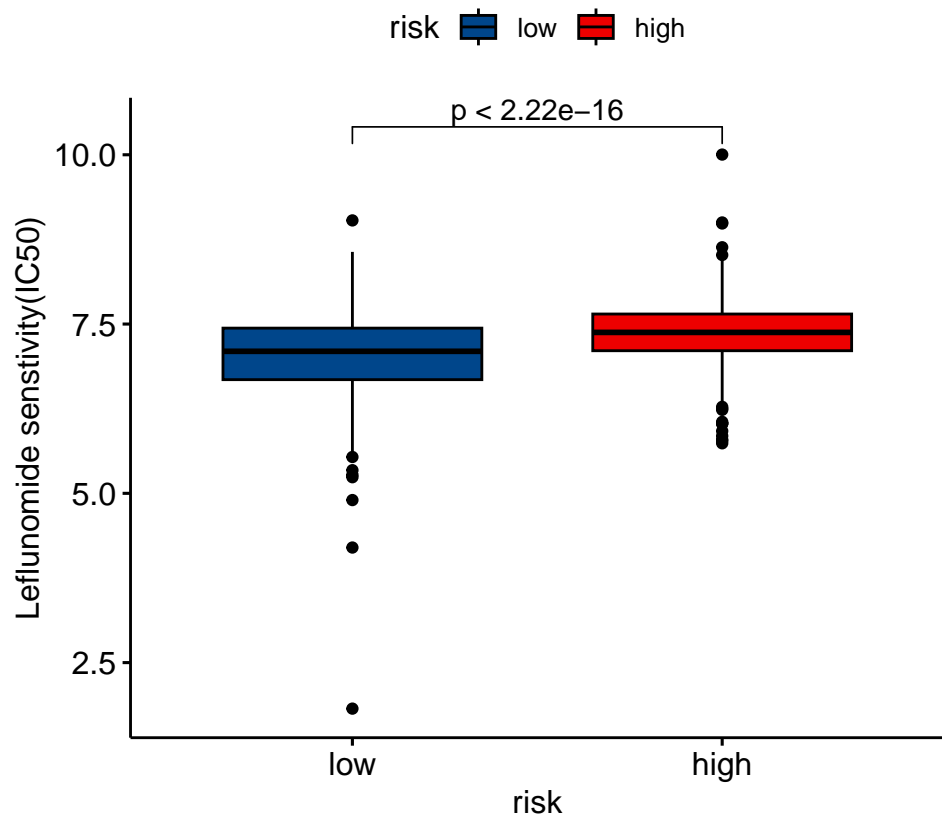

Supplement: Supplementary file 2 — Supplementary file2 (ZIP 3179 KB) [file 10238_2024_1372_MOESM2_ESM.zip › Supplementary Material/Drug1/drugSenstivity.Leflunomide.pdf]

risk low high

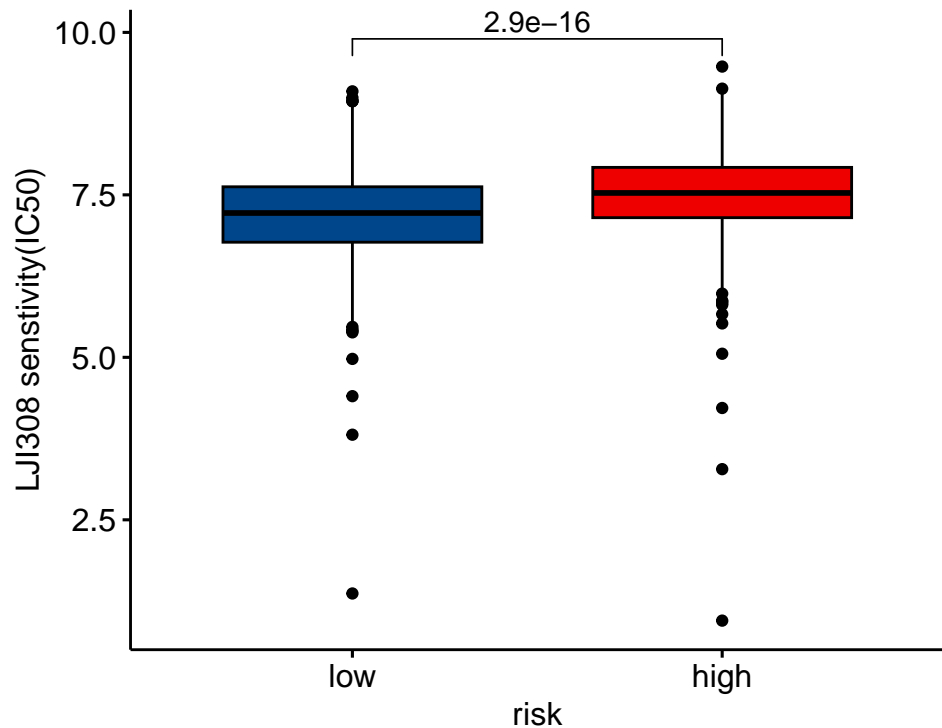

Supplement: Supplementary file 2 — Supplementary file2 (ZIP 3179 KB) [file 10238_2024_1372_MOESM2_ESM.zip › Supplementary Material/Drug1/drugSenstivity.LJI308.pdf]

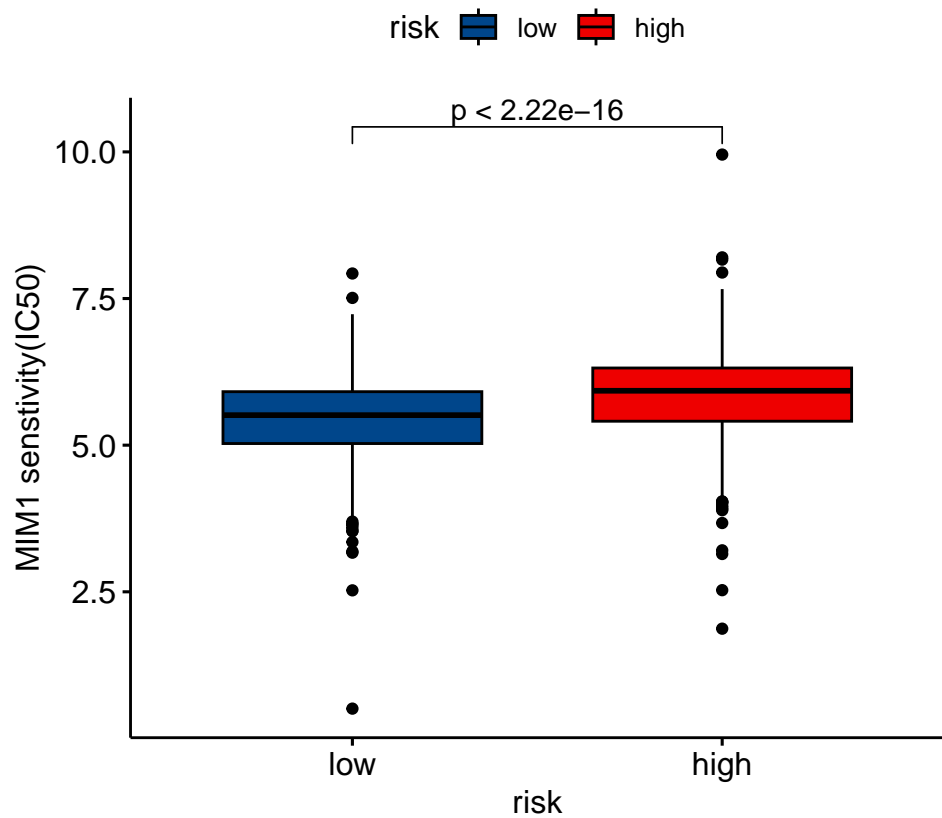

Supplement: Supplementary file 2 — Supplementary file2 (ZIP 3179 KB) [file 10238_2024_1372_MOESM2_ESM.zip › Supplementary Material/Drug1/drugSenstivity.MIM1.pdf]

MIRA-1 sensitivity(IC50)

risk low high

$7.6e-14$

low

high

risk

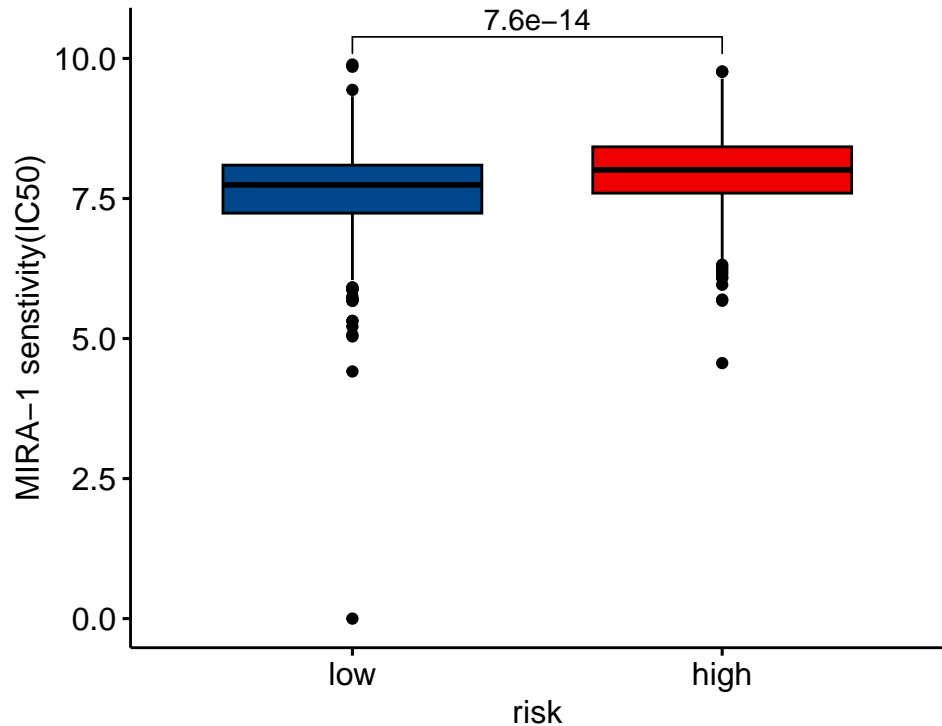

Supplement: Supplementary file 2 — Supplementary file2 (ZIP 3179 KB) [file 10238_2024_1372_MOESM2_ESM.zip › Supplementary Material/Drug1/drugSenstivity.MIRA-1.pdf]

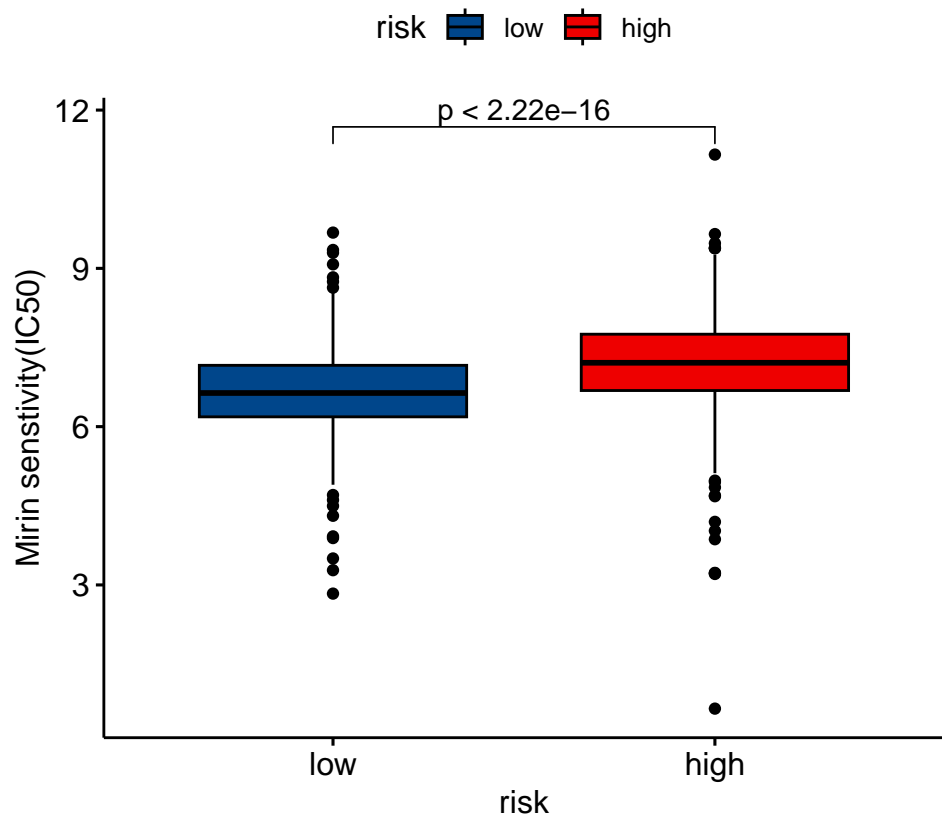

Supplement: Supplementary file 2 — Supplementary file2 (ZIP 3179 KB) [file 10238_2024_1372_MOESM2_ESM.zip › Supplementary Material/Drug1/drugSenstivity.Mirin.pdf]

risk low high

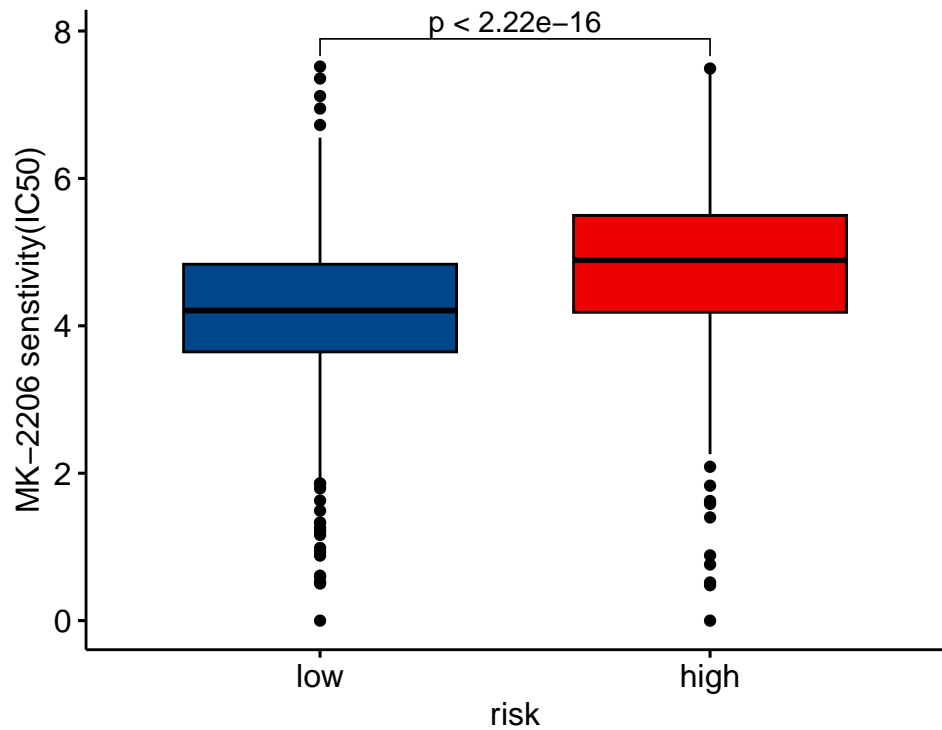

Supplement: Supplementary file 2 — Supplementary file2 (ZIP 3179 KB) [file 10238_2024_1372_MOESM2_ESM.zip › Supplementary Material/Drug1/drugSenstivity.MK-2206.pdf]

risk low high

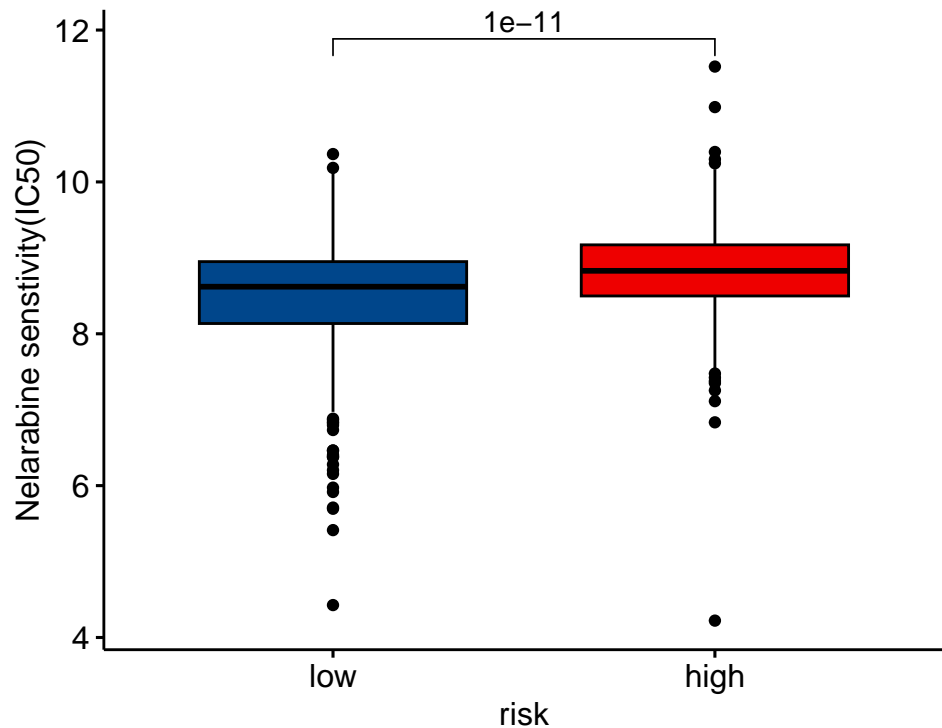

Supplement: Supplementary file 2 — Supplementary file2 (ZIP 3179 KB) [file 10238_2024_1372_MOESM2_ESM.zip › Supplementary Material/Drug1/drugSenstivity.Nelarabine.pdf]

risk low high

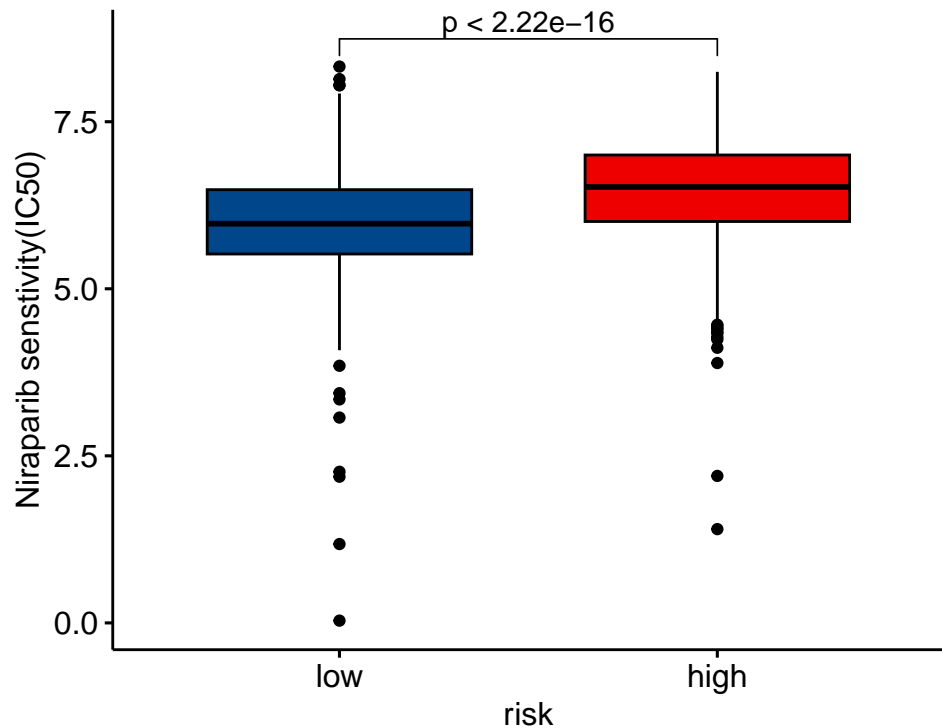

Supplement: Supplementary file 2 — Supplementary file2 (ZIP 3179 KB) [file 10238_2024_1372_MOESM2_ESM.zip › Supplementary Material/Drug1/drugSenstivity.Niraparib.pdf]

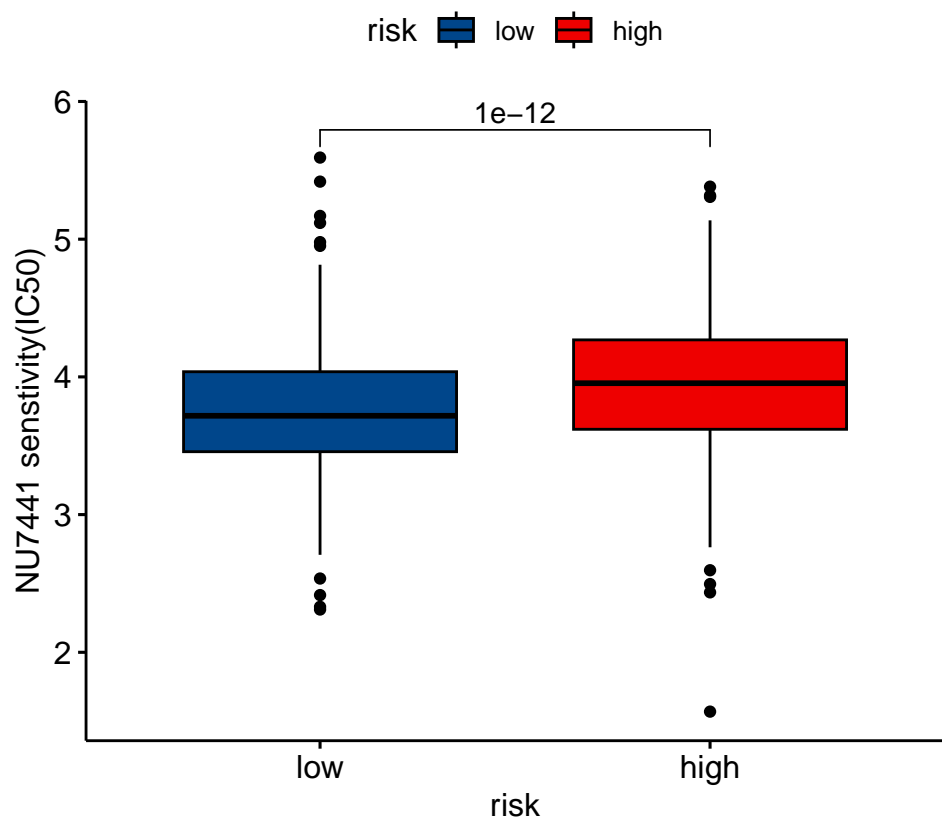

Supplement: Supplementary file 2 — Supplementary file2 (ZIP 3179 KB) [file 10238_2024_1372_MOESM2_ESM.zip › Supplementary Material/Drug1/drugSenstivity.NU7441.pdf]

Nutlin-3a (-) sensitivity(IC50)

risk low high

$p < 2.22e-16$

low

high

risk

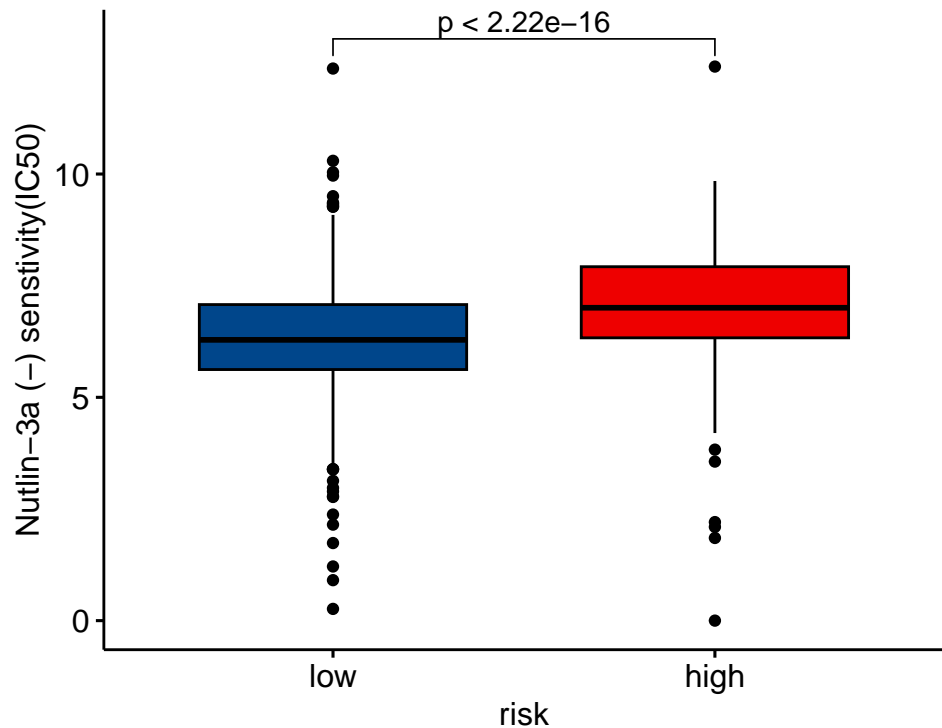

Supplement: Supplementary file 2 — Supplementary file2 (ZIP 3179 KB) [file 10238_2024_1372_MOESM2_ESM.zip › Supplementary Material/Drug1/drugSenstivity.Nutlin-3a (-).pdf]

risk low high

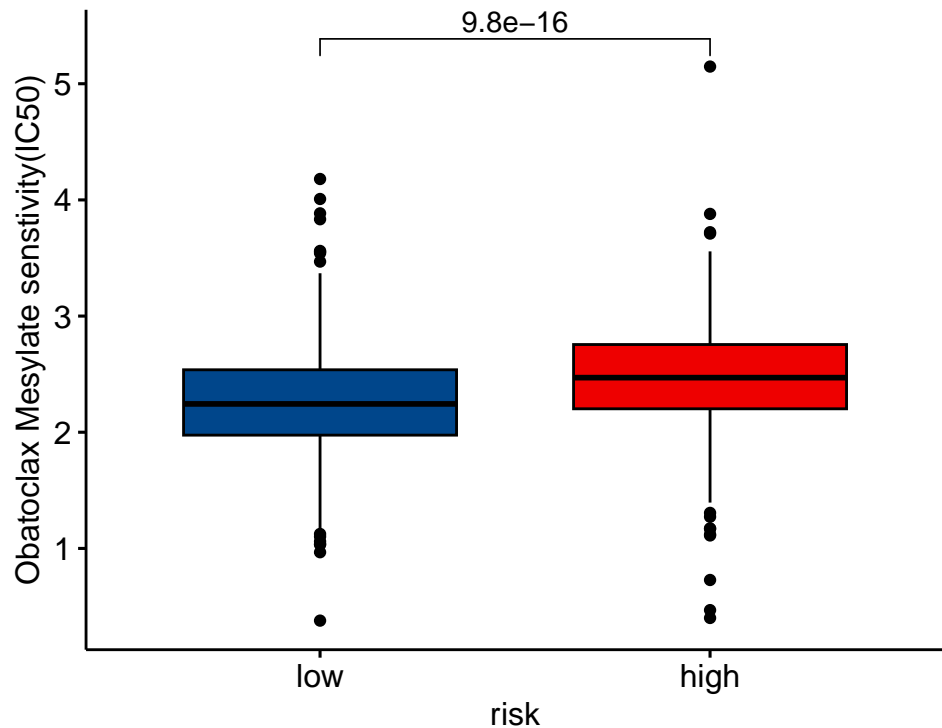

Supplement: Supplementary file 2 — Supplementary file2 (ZIP 3179 KB) [file 10238_2024_1372_MOESM2_ESM.zip › Supplementary Material/Drug1/drugSenstivity.Obatoclax Mesylate.pdf]

risk low high

$p < 2.22e-16$

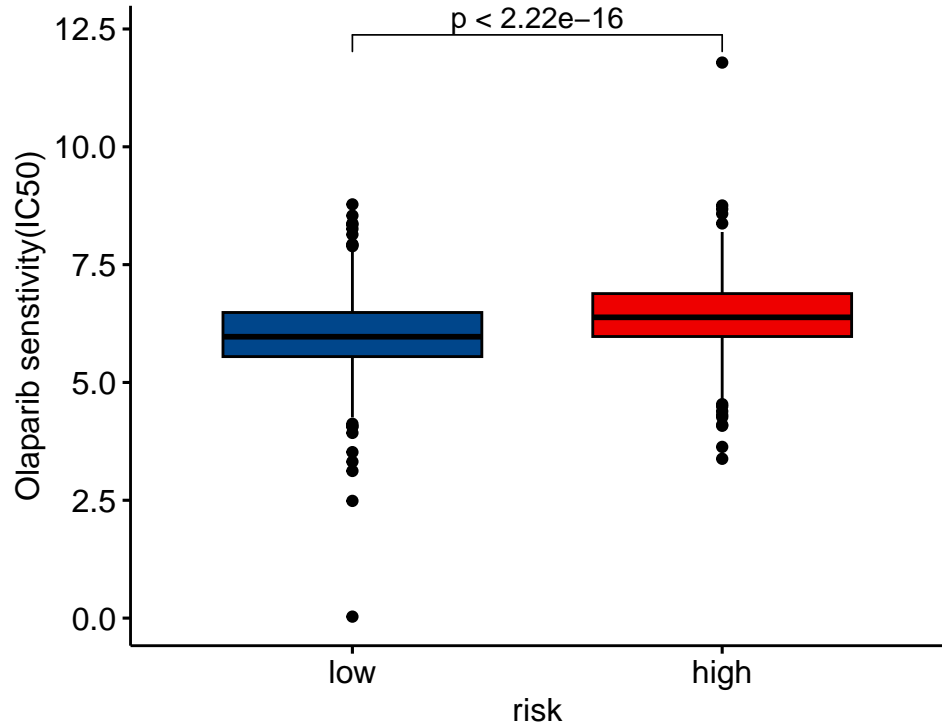

Supplement: Supplementary file 2 — Supplementary file2 (ZIP 3179 KB) [file 10238_2024_1372_MOESM2_ESM.zip › Supplementary Material/Drug1/drugSenstivity.Olaparib.pdf]

risk low high

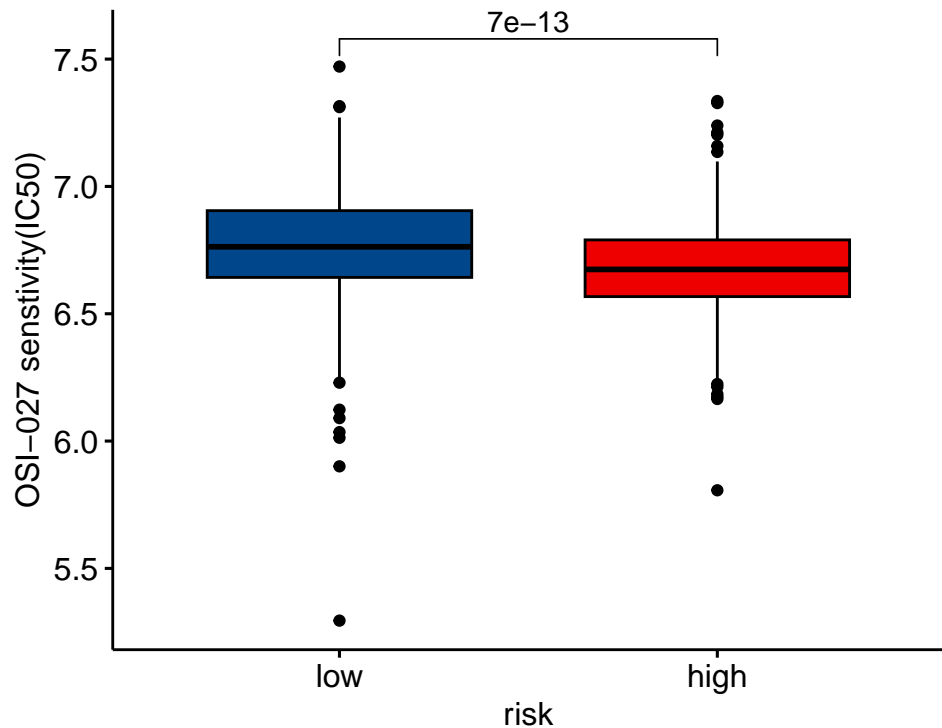

Supplement: Supplementary file 2 — Supplementary file2 (ZIP 3179 KB) [file 10238_2024_1372_MOESM2_ESM.zip › Supplementary Material/Drug1/drugSenstivity.OSI-027.pdf]

risk low high

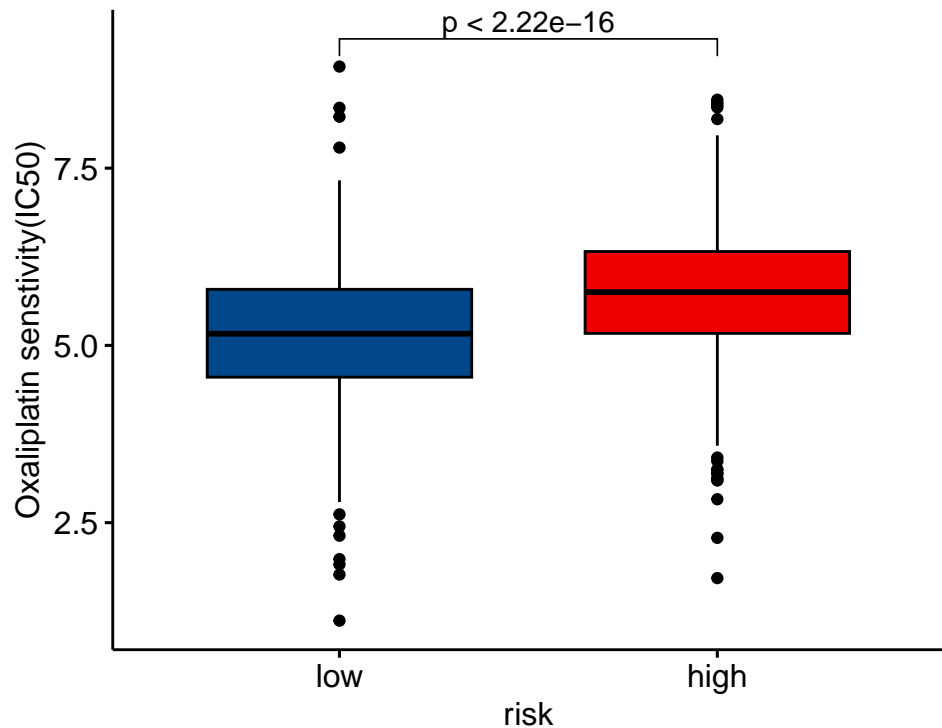

Supplement: Supplementary file 2 — Supplementary file2 (ZIP 3179 KB) [file 10238_2024_1372_MOESM2_ESM.zip › Supplementary Material/Drug1/drugSenstivity.Oxaliplatin.pdf]

risk low high

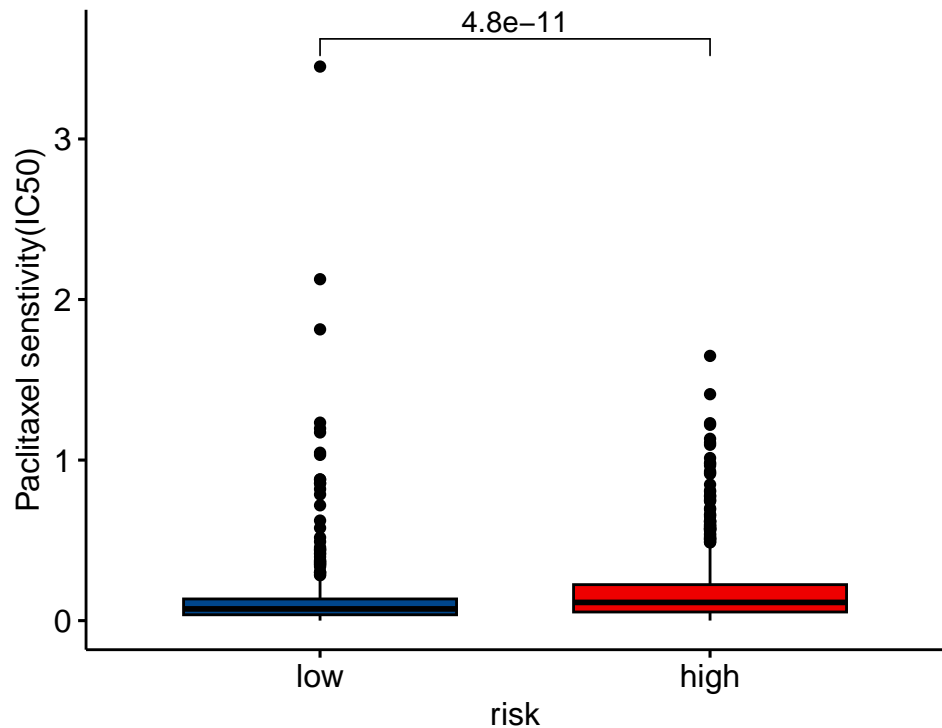

Supplement: Supplementary file 2 — Supplementary file2 (ZIP 3179 KB) [file 10238_2024_1372_MOESM2_ESM.zip › Supplementary Material/Drug1/drugSenstivity.Paclitaxel.pdf]

risk low high

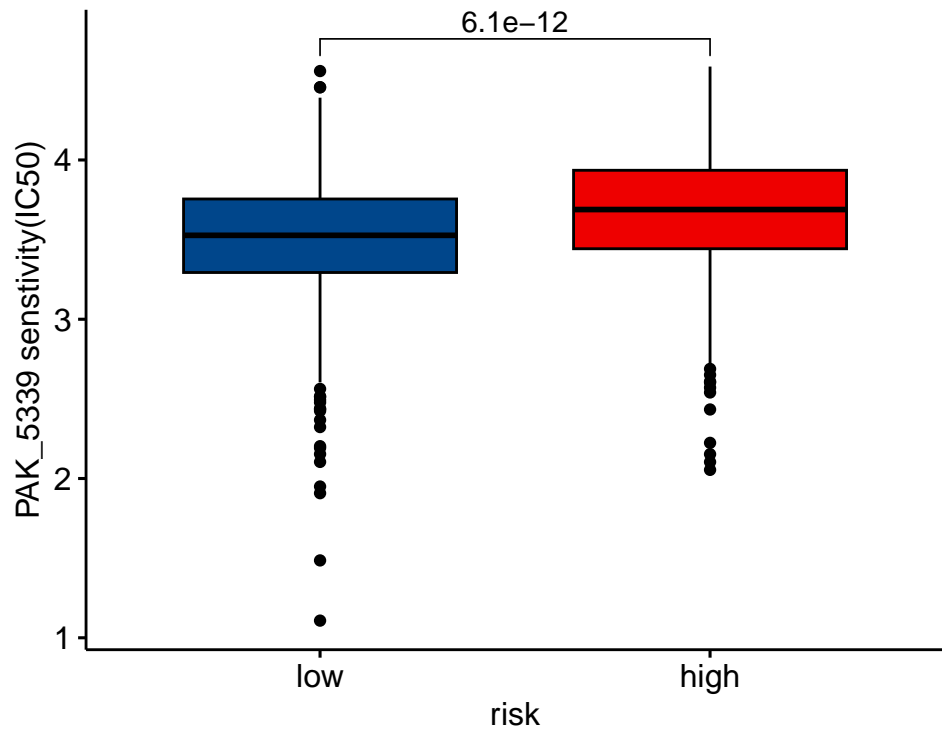

Supplement: Supplementary file 2 — Supplementary file2 (ZIP 3179 KB) [file 10238_2024_1372_MOESM2_ESM.zip › Supplementary Material/Drug1/drugSenstivity.PAK_5339.pdf]

risk low high

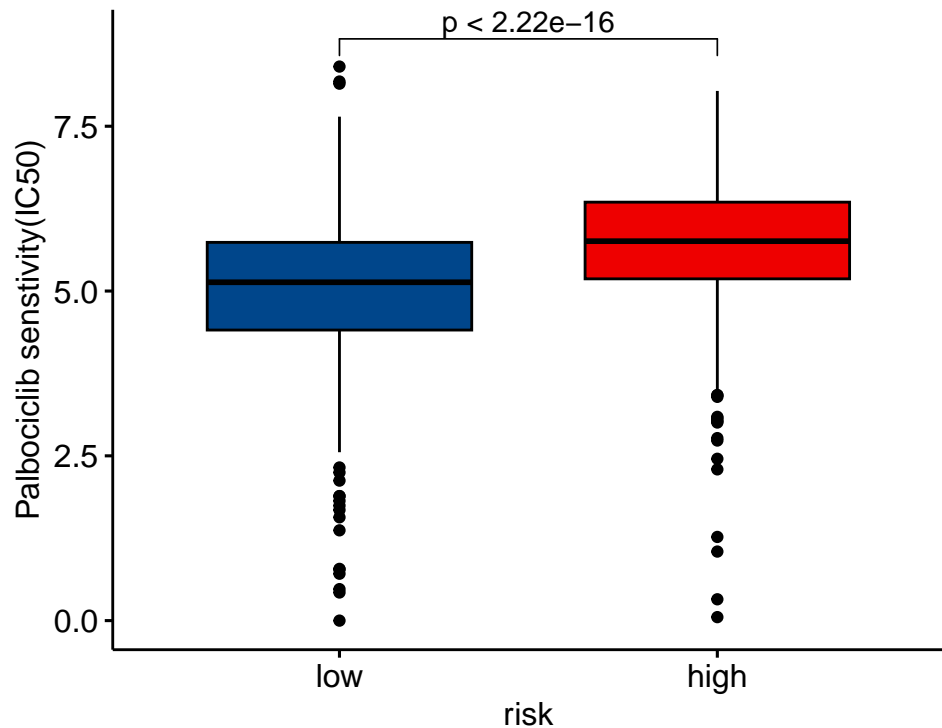

Supplement: Supplementary file 2 — Supplementary file2 (ZIP 3179 KB) [file 10238_2024_1372_MOESM2_ESM.zip › Supplementary Material/Drug1/drugSenstivity.Palbociclib.pdf]

PCI-34051 sensitivity(IC50)

risk low high

$2.3e-13$

low

high

risk

10.0  
7.5  
5.0  
2.5  
0.0

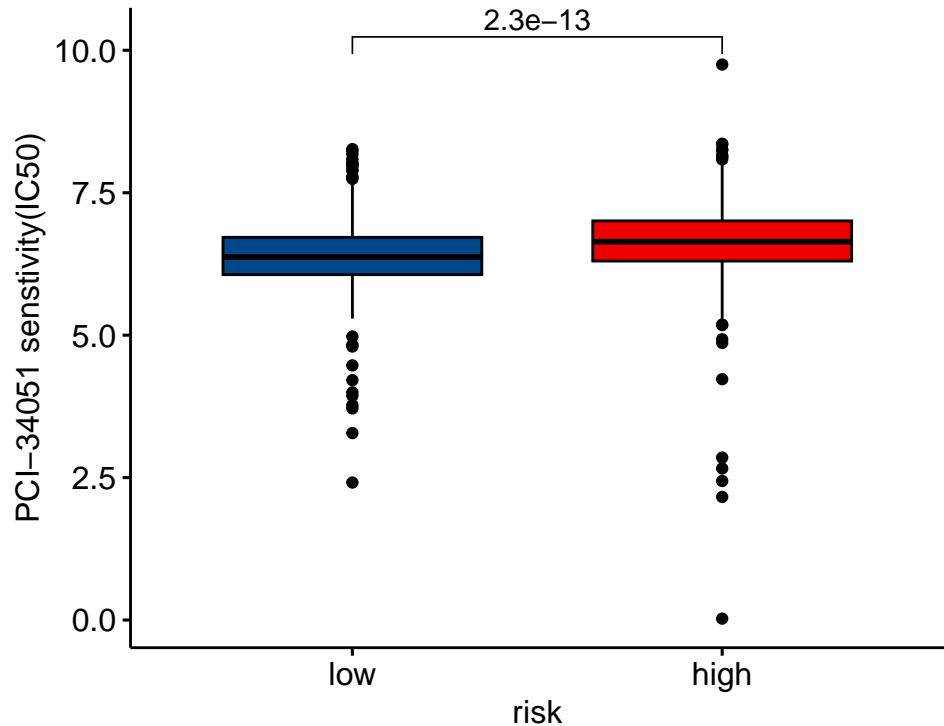

Supplement: Supplementary file 2 — Supplementary file2 (ZIP 3179 KB) [file 10238_2024_1372_MOESM2_ESM.zip › Supplementary Material/Drug1/drugSenstivity.PCI-34051.pdf]

risk 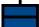 low 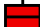 high

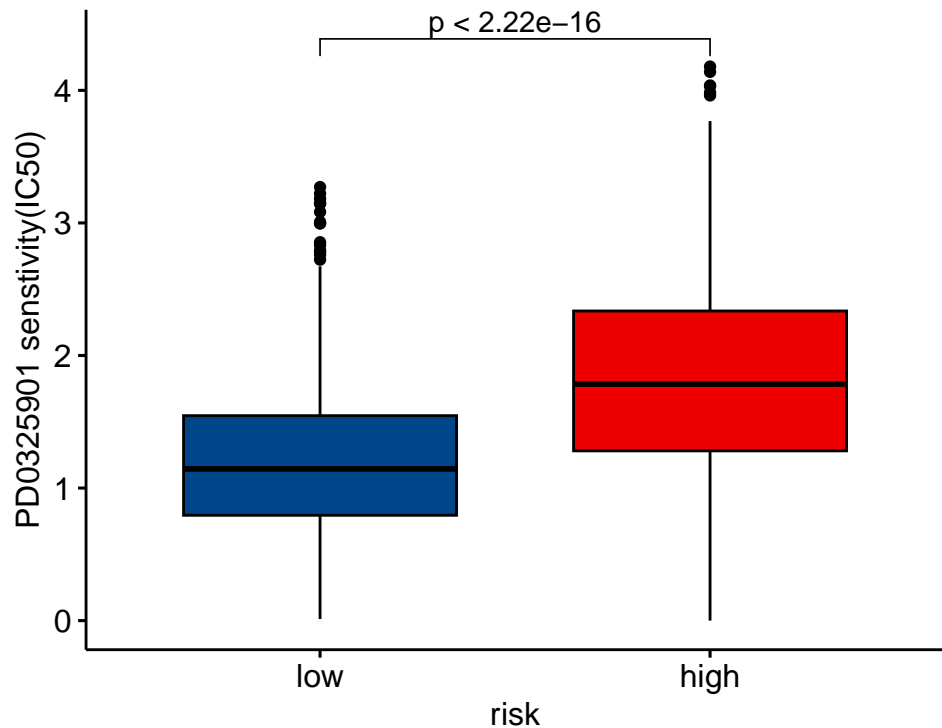

Supplement: Supplementary file 2 — Supplementary file2 (ZIP 3179 KB) [file 10238_2024_1372_MOESM2_ESM.zip › Supplementary Material/Drug1/drugSenstivity.PD0325901.pdf]

risk 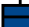 low 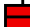 high

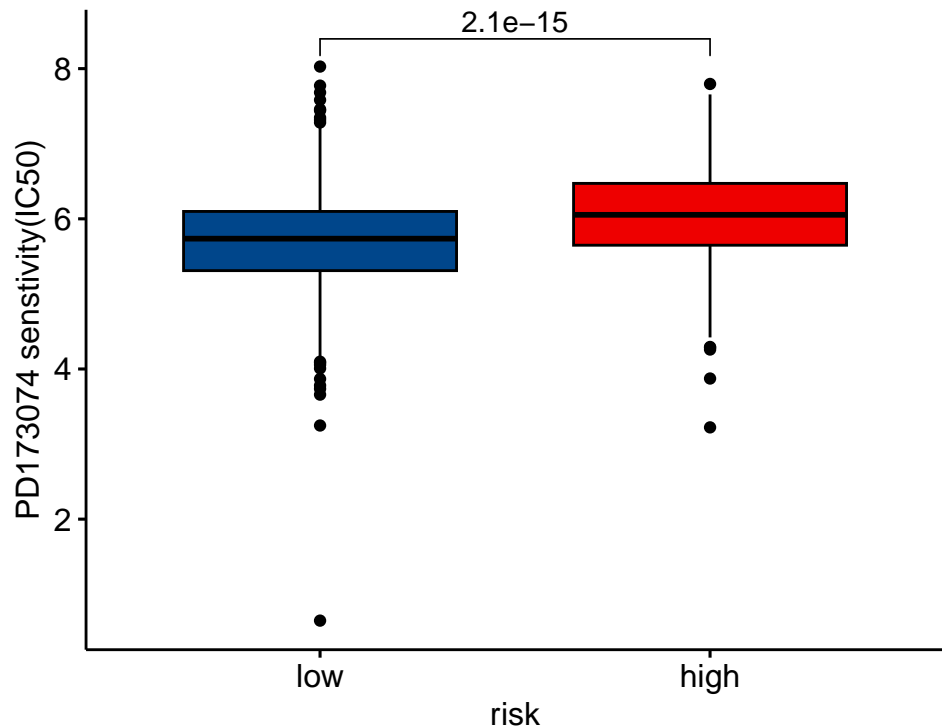

Supplement: Supplementary file 2 — Supplementary file2 (ZIP 3179 KB) [file 10238_2024_1372_MOESM2_ESM.zip › Supplementary Material/Drug1/drugSenstivity.PD173074.pdf]

risk low high

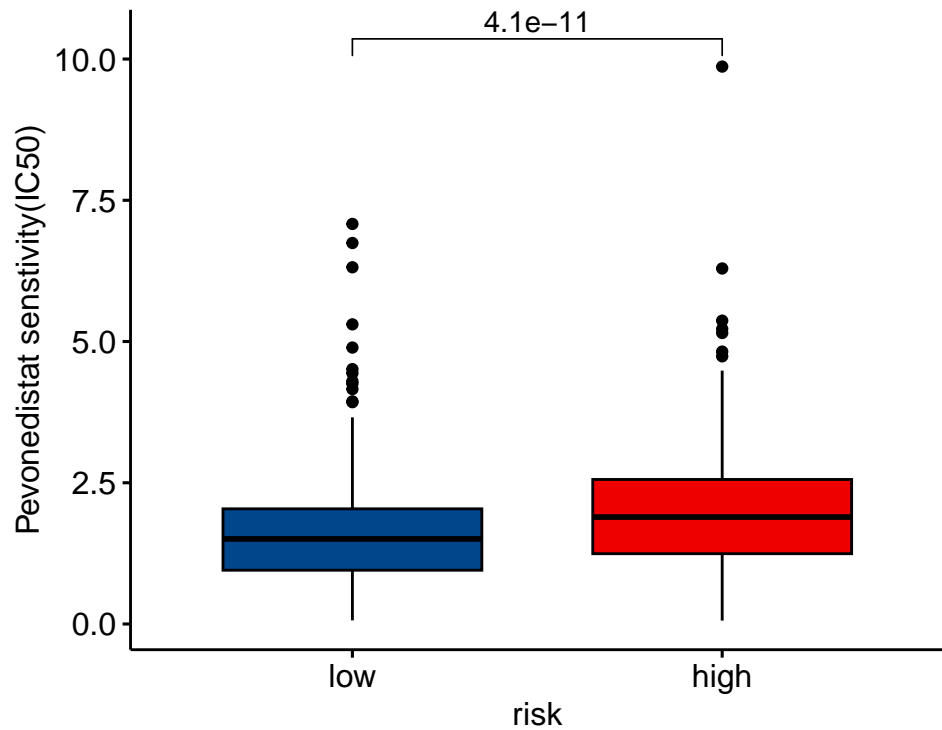

Supplement: Supplementary file 2 — Supplementary file2 (ZIP 3179 KB) [file 10238_2024_1372_MOESM2_ESM.zip › Supplementary Material/Drug1/drugSenstivity.Pevonedistat.pdf]

PF-4708671 sensitivity(IC50)

risk low high

$p < 2.22e-16$

low

high

risk

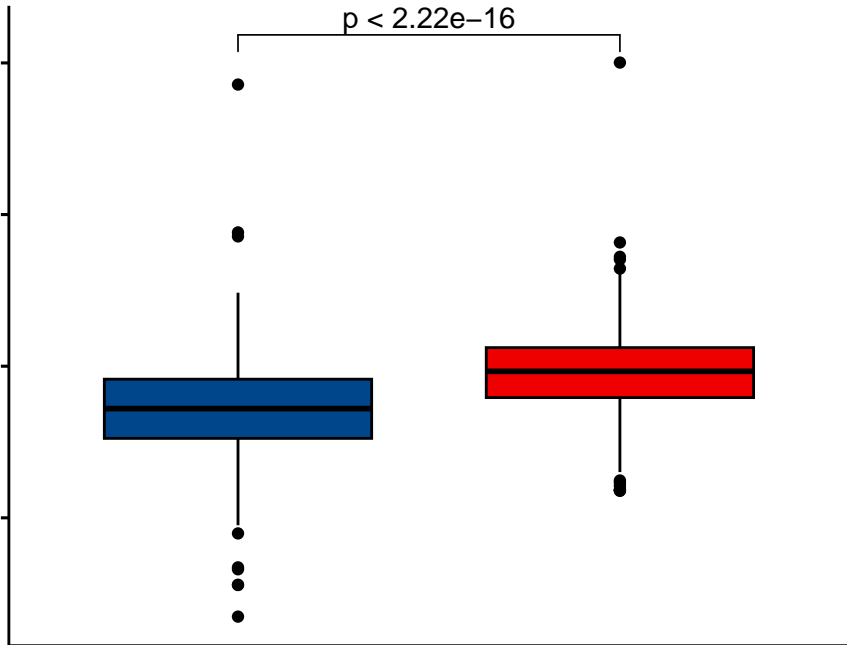

Supplement: Supplementary file 2 — Supplementary file2 (ZIP 3179 KB) [file 10238_2024_1372_MOESM2_ESM.zip › Supplementary Material/Drug1/drugSenstivity.PF-4708671.pdf]

risk low high

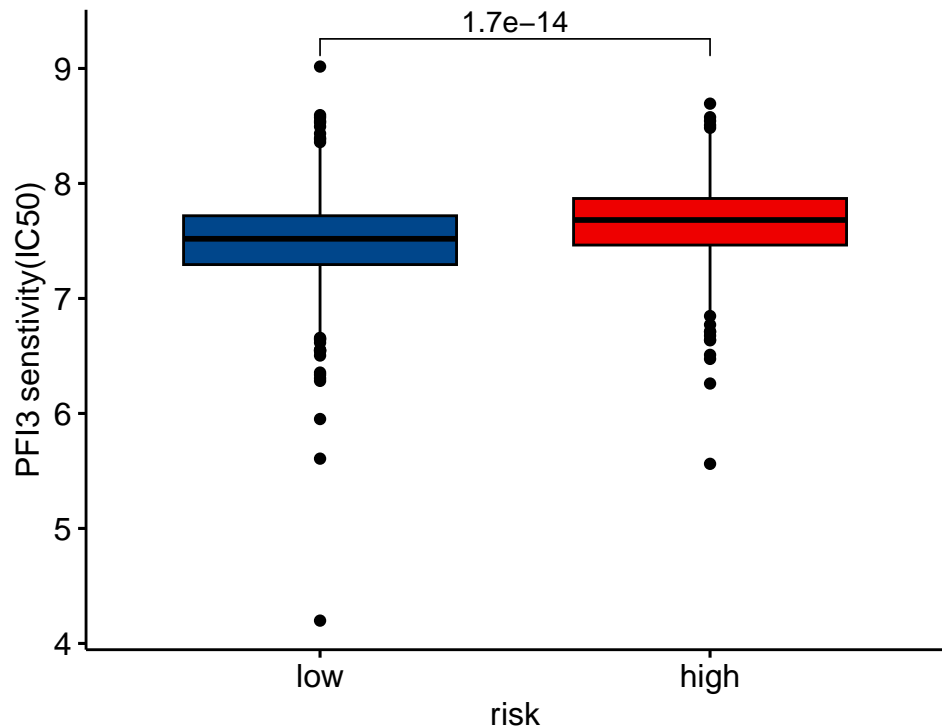

Supplement: Supplementary file 2 — Supplementary file2 (ZIP 3179 KB) [file 10238_2024_1372_MOESM2_ESM.zip › Supplementary Material/Drug1/drugSenstivity.PFI3.pdf]

risk low high

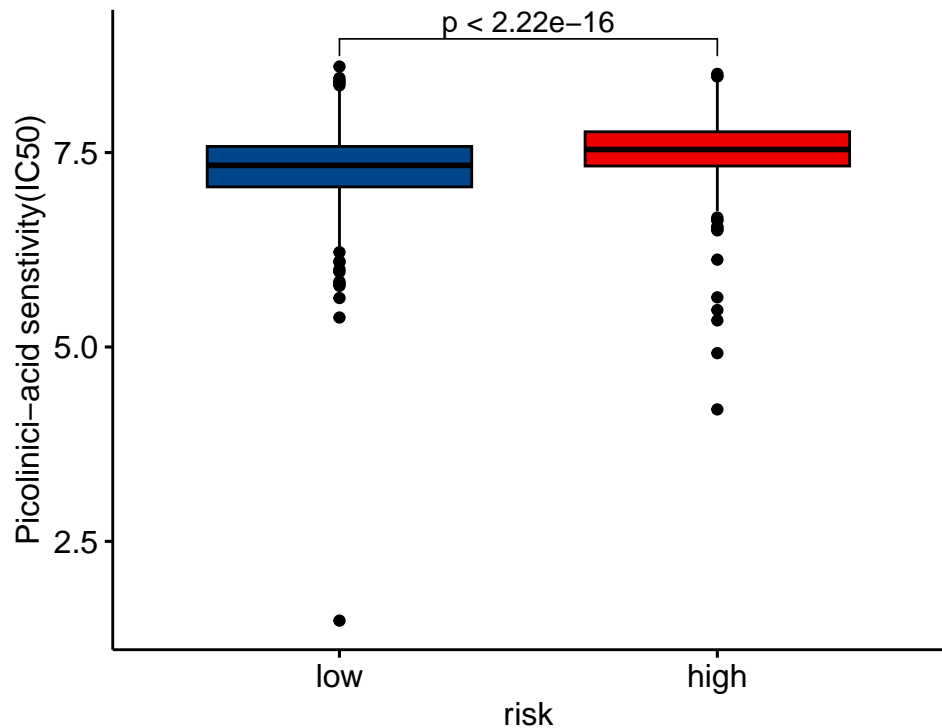

Supplement: Supplementary file 2 — Supplementary file2 (ZIP 3179 KB) [file 10238_2024_1372_MOESM2_ESM.zip › Supplementary Material/Drug1/drugSenstivity.Picolinici-acid.pdf]

risk low high

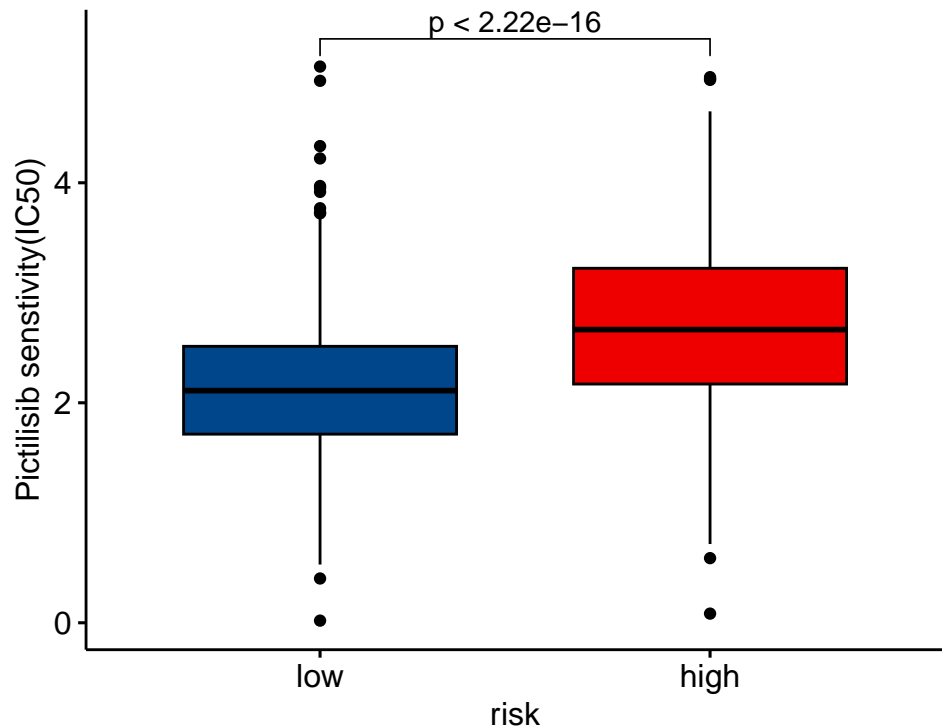

Supplement: Supplementary file 2 — Supplementary file2 (ZIP 3179 KB) [file 10238_2024_1372_MOESM2_ESM.zip › Supplementary Material/Drug1/drugSenstivity.Pictilisib.pdf]

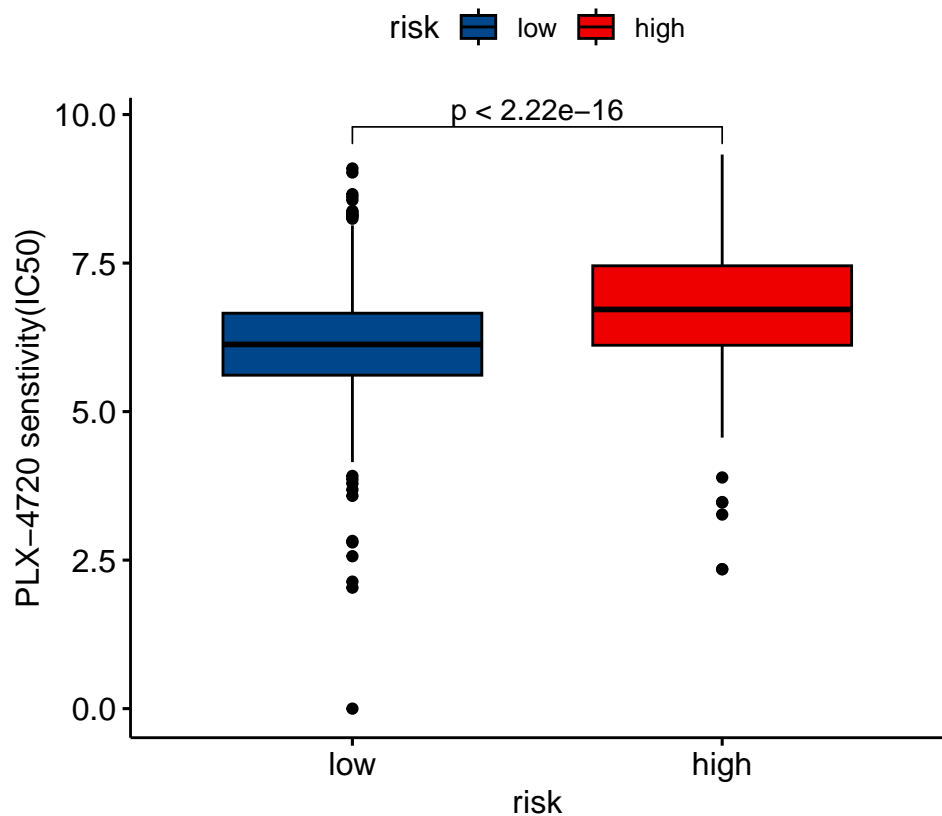

Supplement: Supplementary file 2 — Supplementary file2 (ZIP 3179 KB) [file 10238_2024_1372_MOESM2_ESM.zip › Supplementary Material/Drug1/drugSenstivity.PLX-4720.pdf]

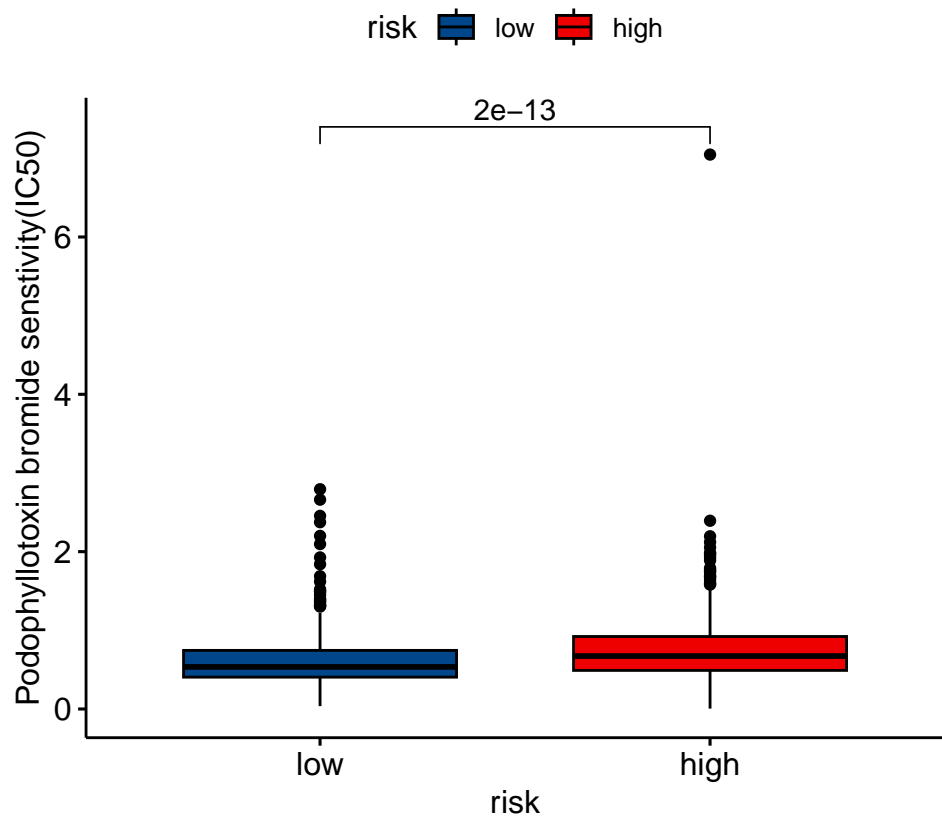

Supplement: Supplementary file 2 — Supplementary file2 (ZIP 3179 KB) [file 10238_2024_1372_MOESM2_ESM.zip › Supplementary Material/Drug1/drugSenstivity.Podophyllotoxin bromide.pdf]

risk low high

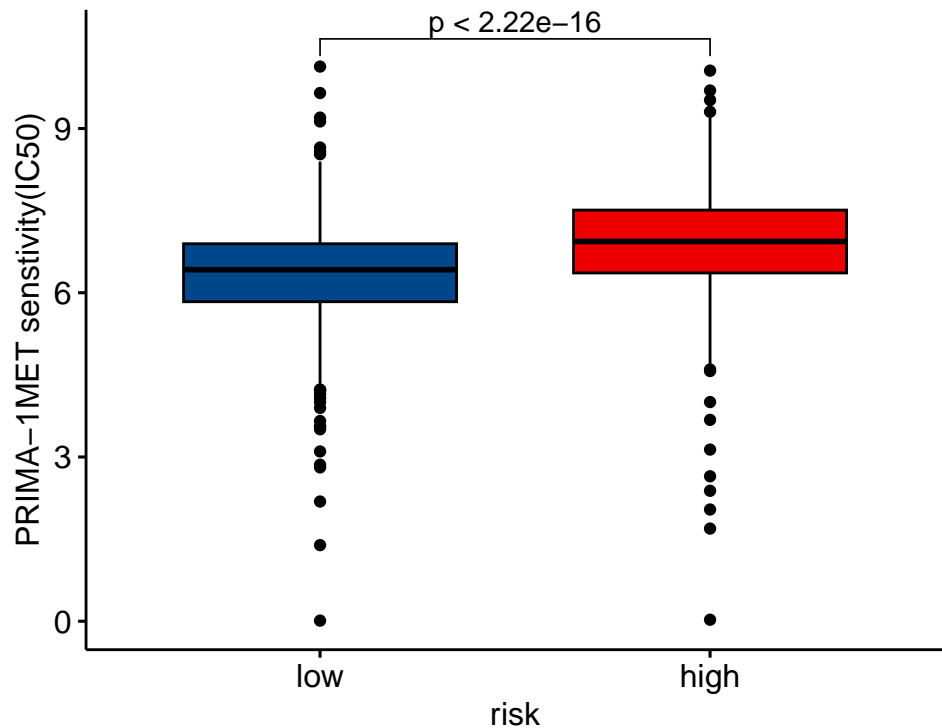

Supplement: Supplementary file 2 — Supplementary file2 (ZIP 3179 KB) [file 10238_2024_1372_MOESM2_ESM.zip › Supplementary Material/Drug1/drugSenstivity.PRIMA-1MET.pdf]

risk low high

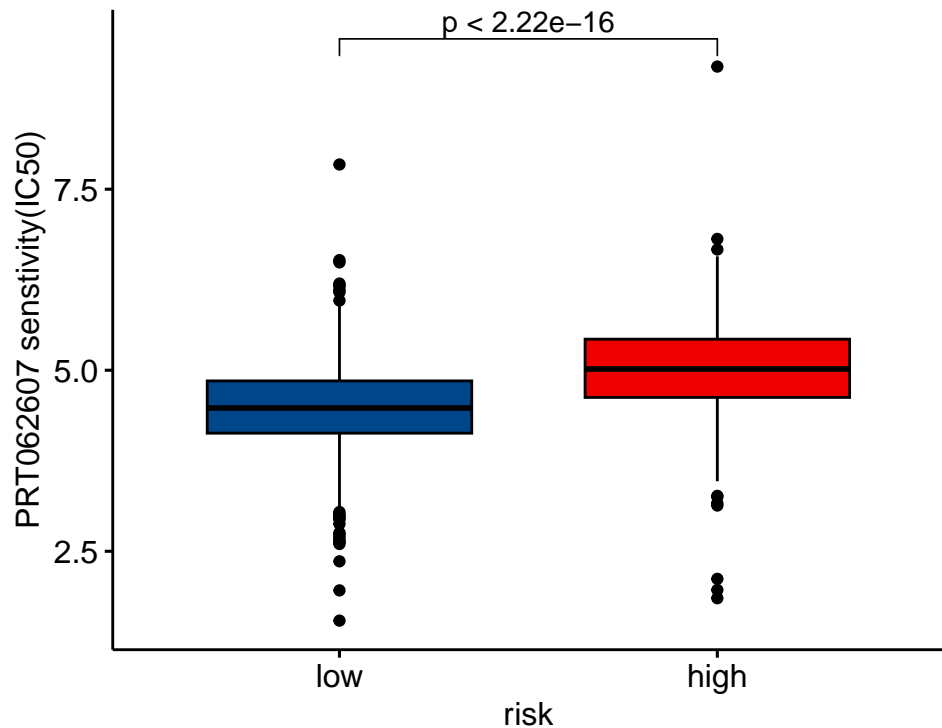

Supplement: Supplementary file 2 — Supplementary file2 (ZIP 3179 KB) [file 10238_2024_1372_MOESM2_ESM.zip › Supplementary Material/Drug1/drugSenstivity.PRT062607.pdf]

risk 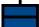 low 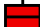 high

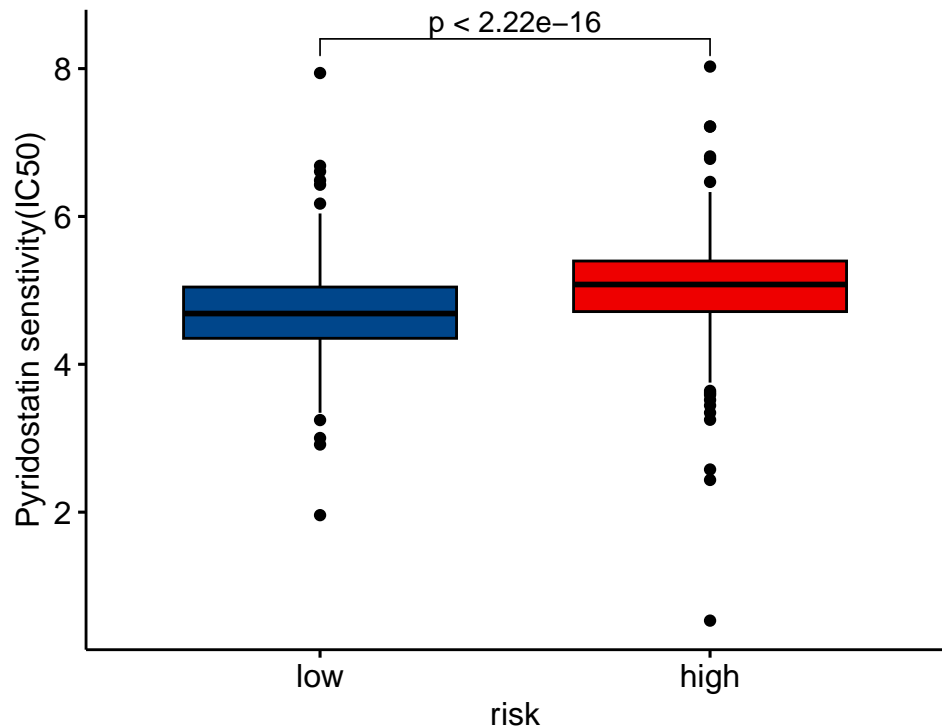

Supplement: Supplementary file 2 — Supplementary file2 (ZIP 3179 KB) [file 10238_2024_1372_MOESM2_ESM.zip › Supplementary Material/Drug1/drugSenstivity.Pyridostatin.pdf]

risk low high

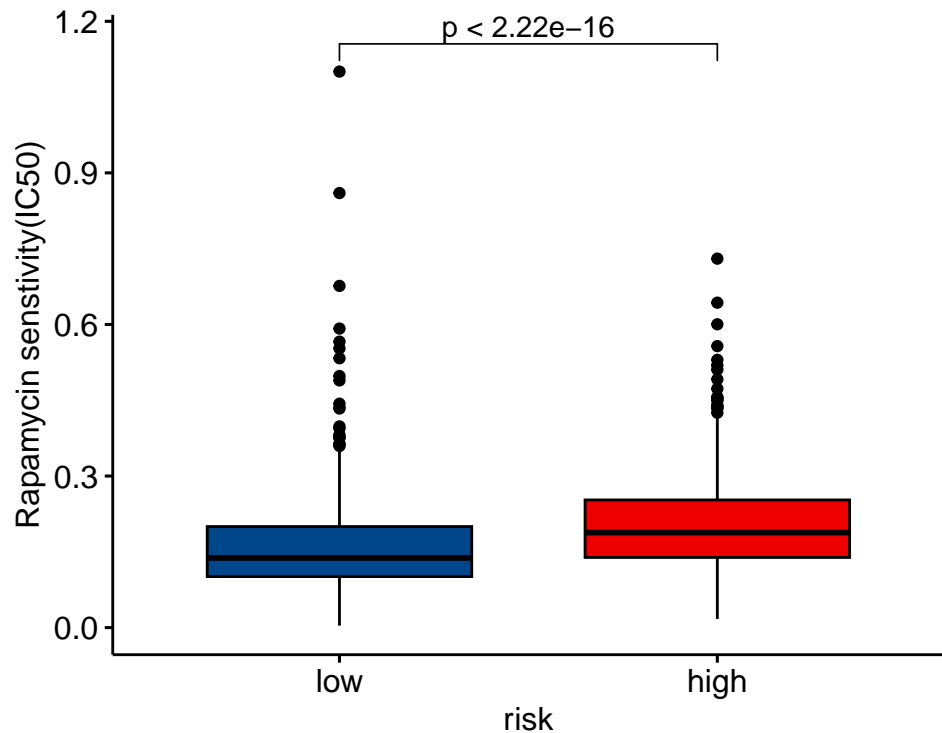

Supplement: Supplementary file 2 — Supplementary file2 (ZIP 3179 KB) [file 10238_2024_1372_MOESM2_ESM.zip › Supplementary Material/Drug1/drugSenstivity.Rapamycin.pdf]

risk low high

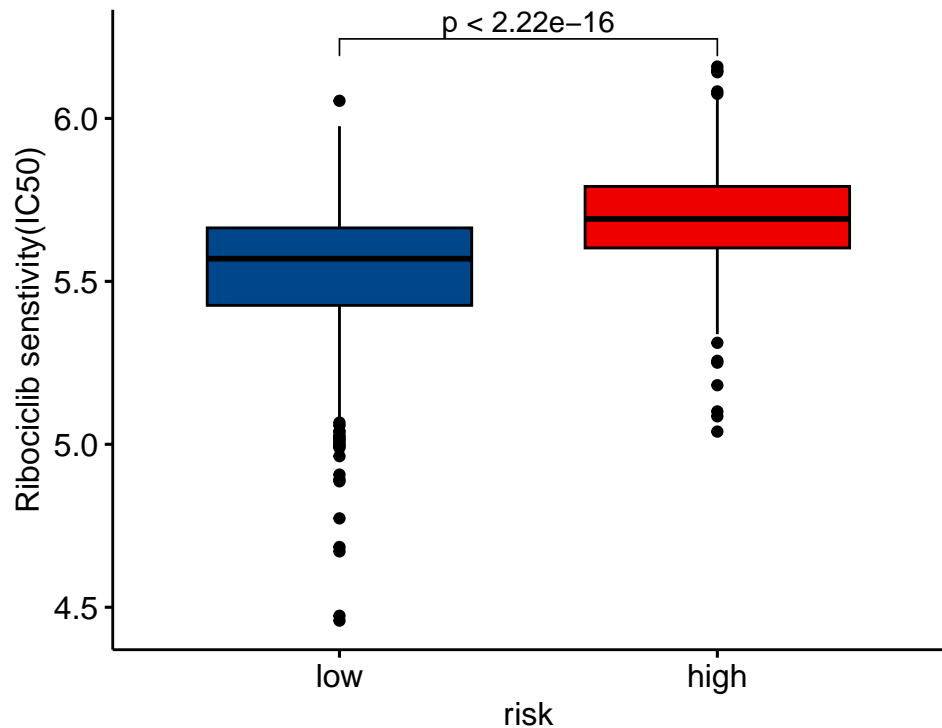

Supplement: Supplementary file 2 — Supplementary file2 (ZIP 3179 KB) [file 10238_2024_1372_MOESM2_ESM.zip › Supplementary Material/Drug1/drugSenstivity.Ribociclib.pdf]

risk low high

$p < 2.22e-16$

Ruxolitinib sensitivity(IC50)

10.0  
7.5  
5.0  
2.5  
0.0

low

high

risk

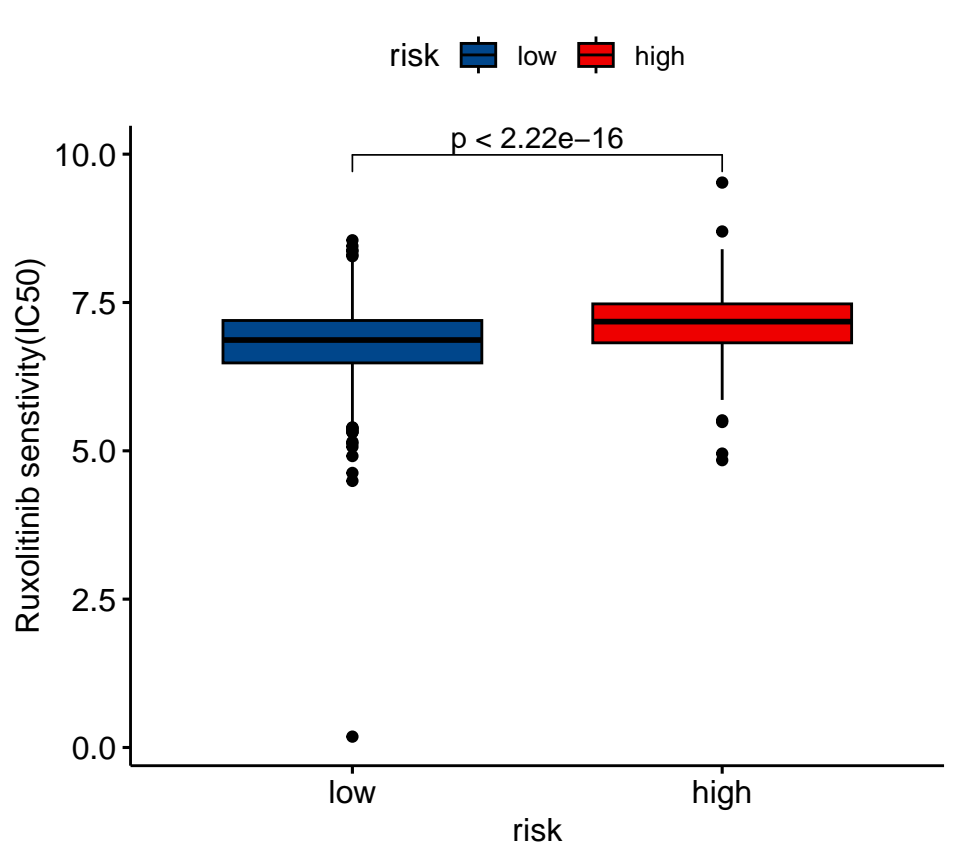

Supplement: Supplementary file 2 — Supplementary file2 (ZIP 3179 KB) [file 10238_2024_1372_MOESM2_ESM.zip › Supplementary Material/Drug1/drugSenstivity.Ruxolitinib.pdf]

risk low high

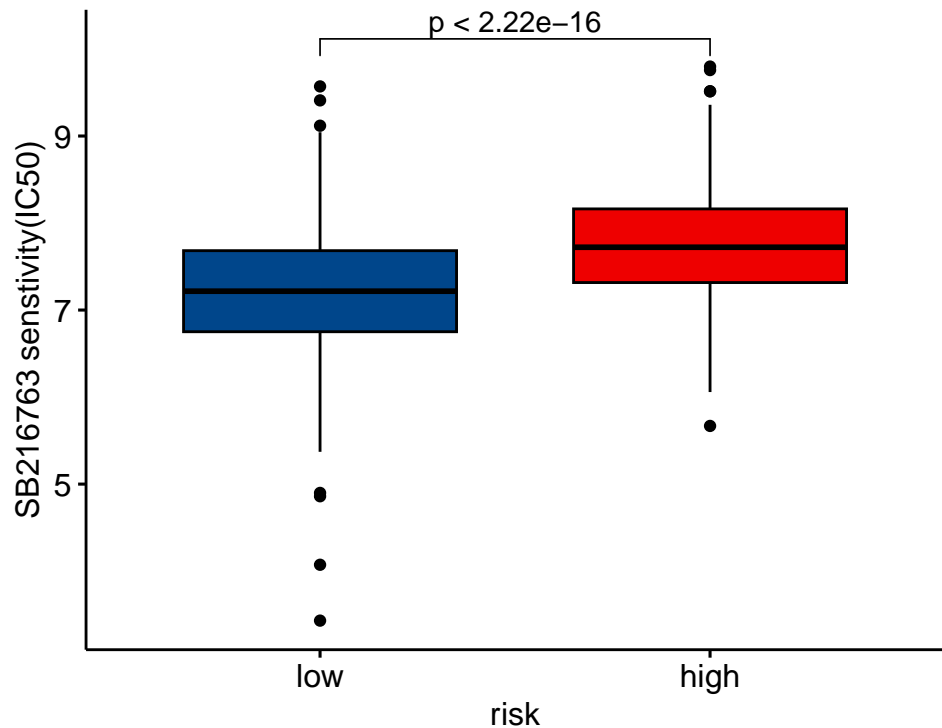

Supplement: Supplementary file 2 — Supplementary file2 (ZIP 3179 KB) [file 10238_2024_1372_MOESM2_ESM.zip › Supplementary Material/Drug1/drugSenstivity.SB216763.pdf]

risk low high

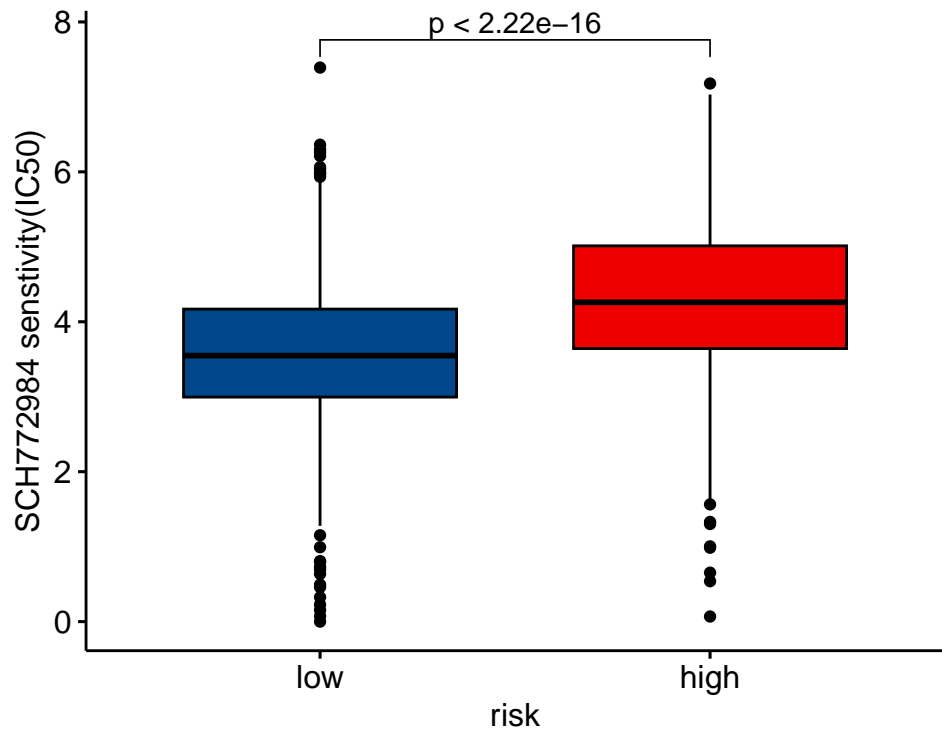

Supplement: Supplementary file 2 — Supplementary file2 (ZIP 3179 KB) [file 10238_2024_1372_MOESM2_ESM.zip › Supplementary Material/Drug1/drugSenstivity.SCH772984.pdf]

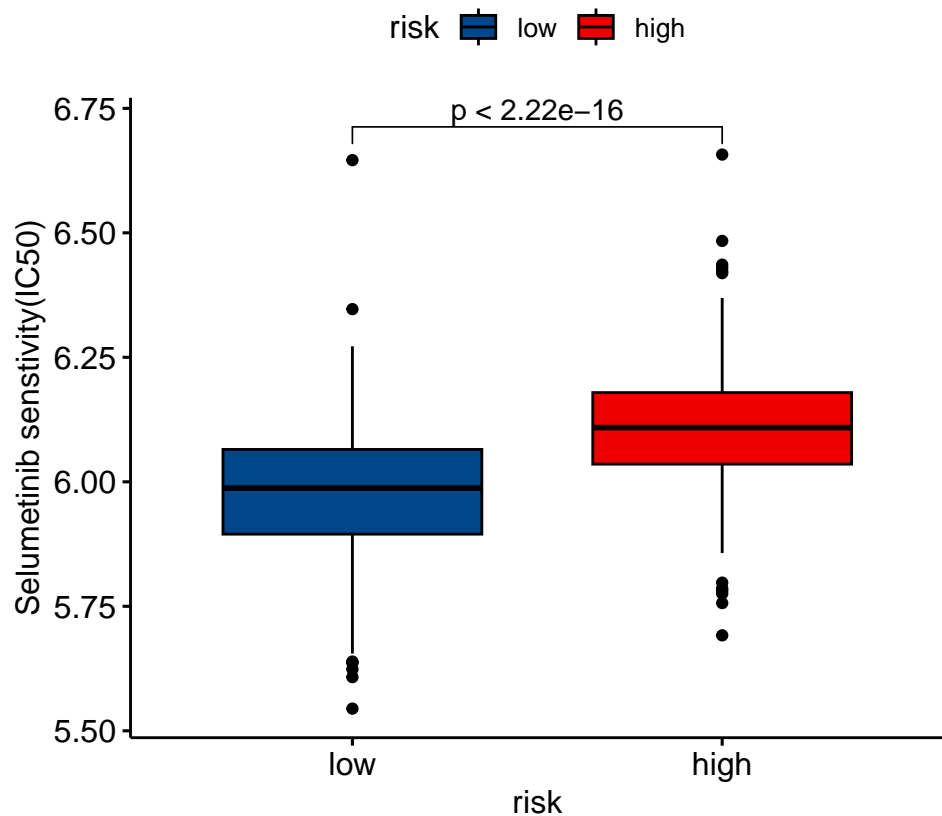

Supplement: Supplementary file 2 — Supplementary file2 (ZIP 3179 KB) [file 10238_2024_1372_MOESM2_ESM.zip › Supplementary Material/Drug1/drugSenstivity.Selumetinib.pdf]

risk low high

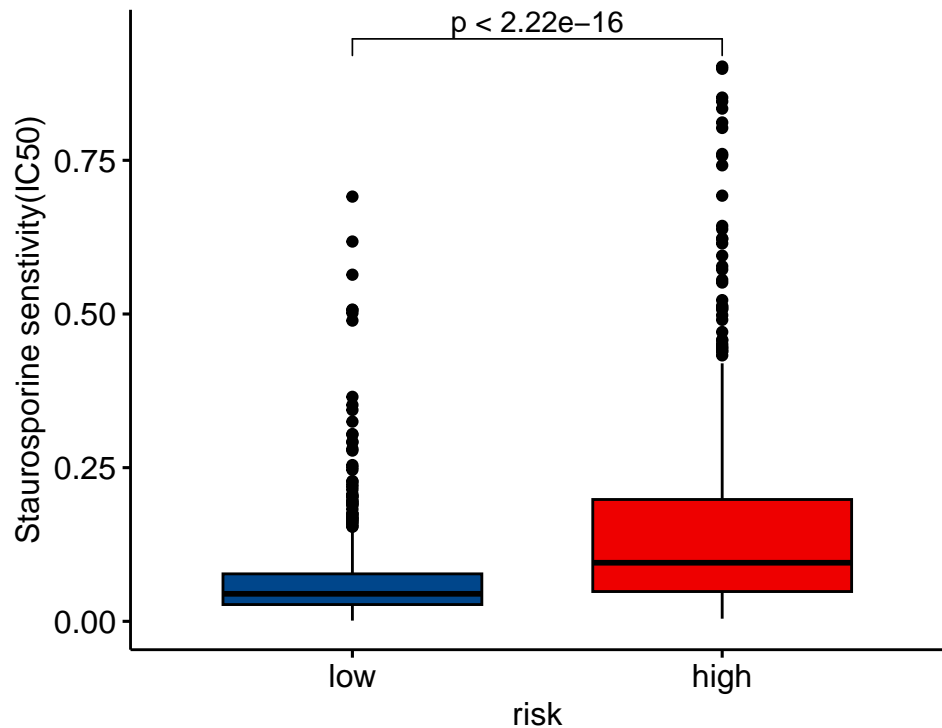

Supplement: Supplementary file 2 — Supplementary file2 (ZIP 3179 KB) [file 10238_2024_1372_MOESM2_ESM.zip › Supplementary Material/Drug1/drugSenstivity.Staurosporine.pdf]

risk low high

$p < 2.22e-16$

Talazoparib sensitivity(IC50)

low

high

risk

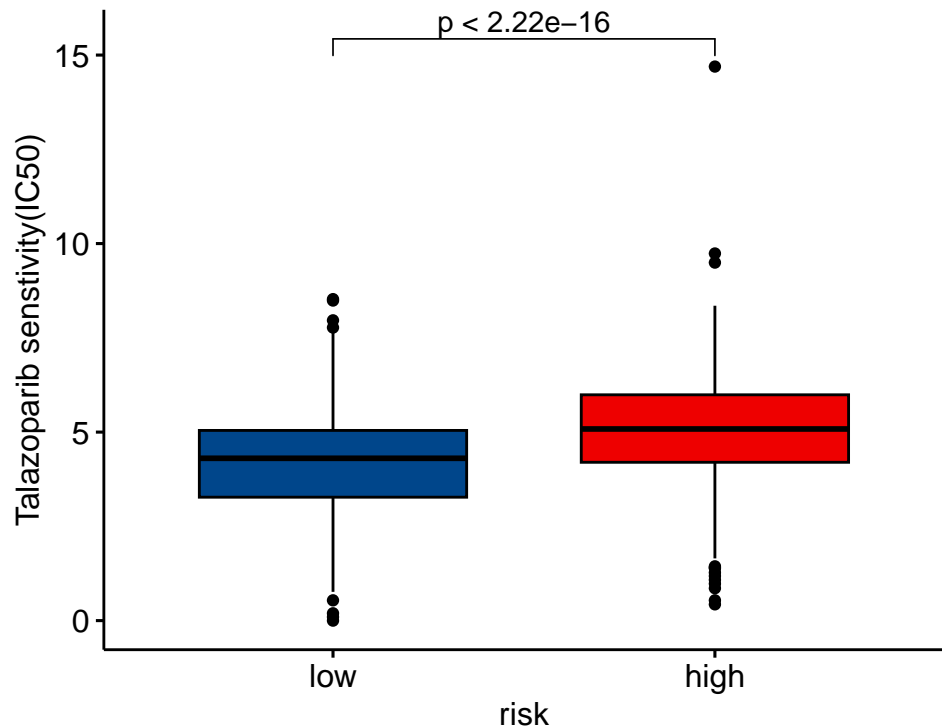

Supplement: Supplementary file 2 — Supplementary file2 (ZIP 3179 KB) [file 10238_2024_1372_MOESM2_ESM.zip › Supplementary Material/Drug1/drugSenstivity.Talazoparib.pdf]

risk low high

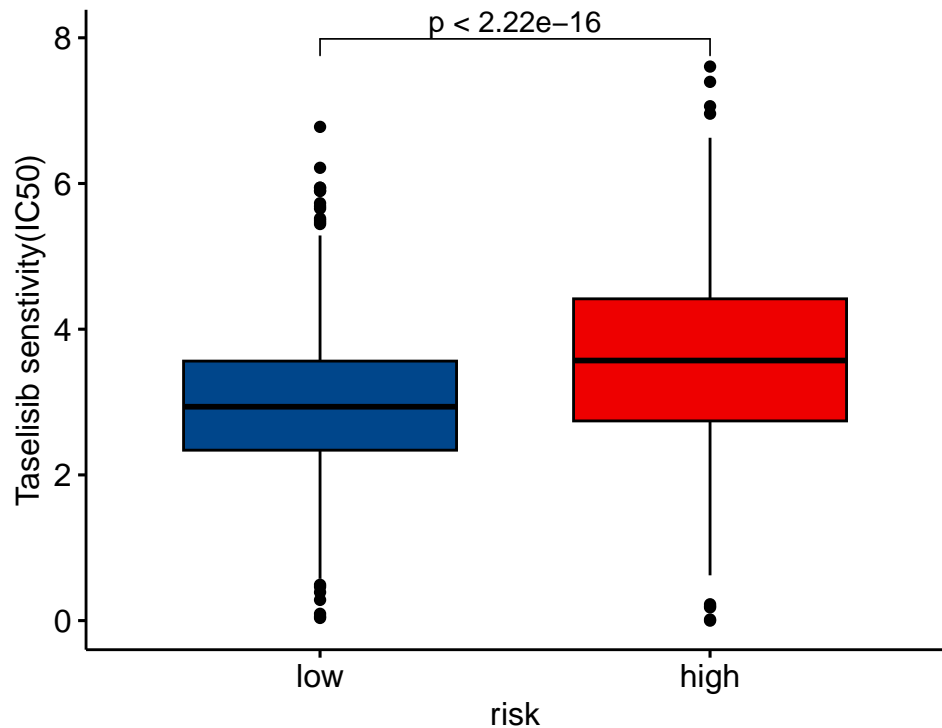

Supplement: Supplementary file 2 — Supplementary file2 (ZIP 3179 KB) [file 10238_2024_1372_MOESM2_ESM.zip › Supplementary Material/Drug1/drugSenstivity.Taselisib.pdf]

risk low high

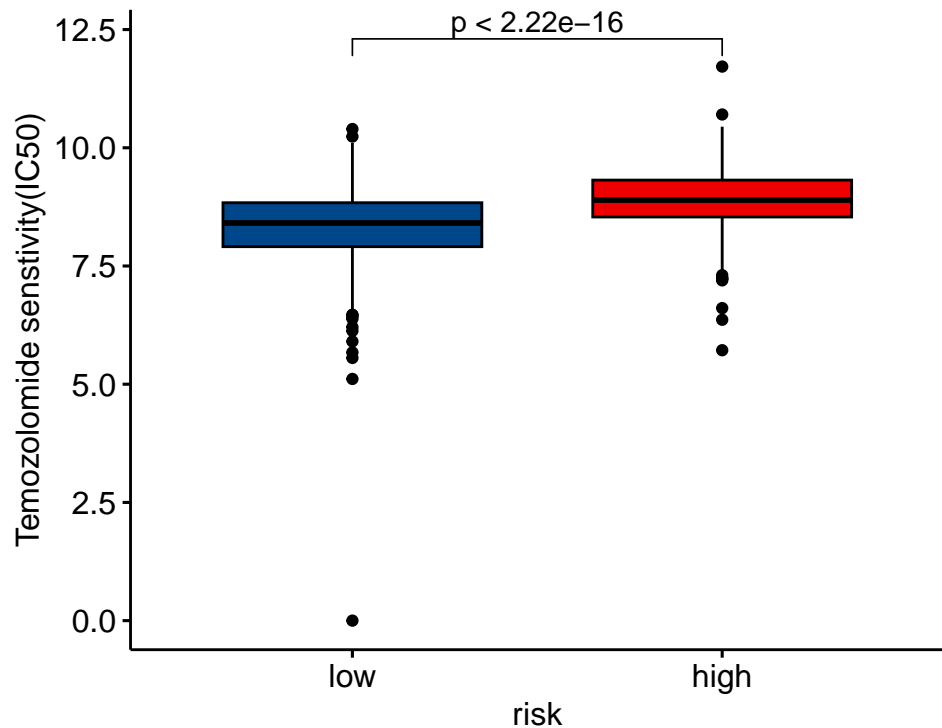

Supplement: Supplementary file 2 — Supplementary file2 (ZIP 3179 KB) [file 10238_2024_1372_MOESM2_ESM.zip › Supplementary Material/Drug1/drugSenstivity.Temozolomide.pdf]

risk low high

$p < 2.22e-16$

Teniposide sensitivity(IC50)

10.0  
7.5  
5.0  
2.5  
0.0

low

high

risk

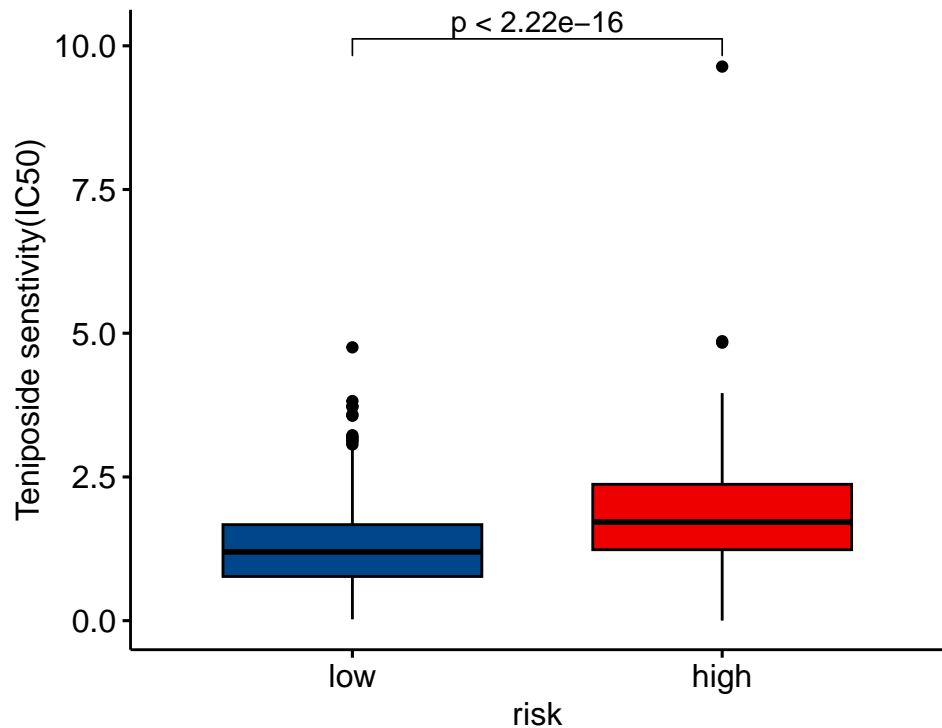

Supplement: Supplementary file 2 — Supplementary file2 (ZIP 3179 KB) [file 10238_2024_1372_MOESM2_ESM.zip › Supplementary Material/Drug1/drugSenstivity.Teniposide.pdf]

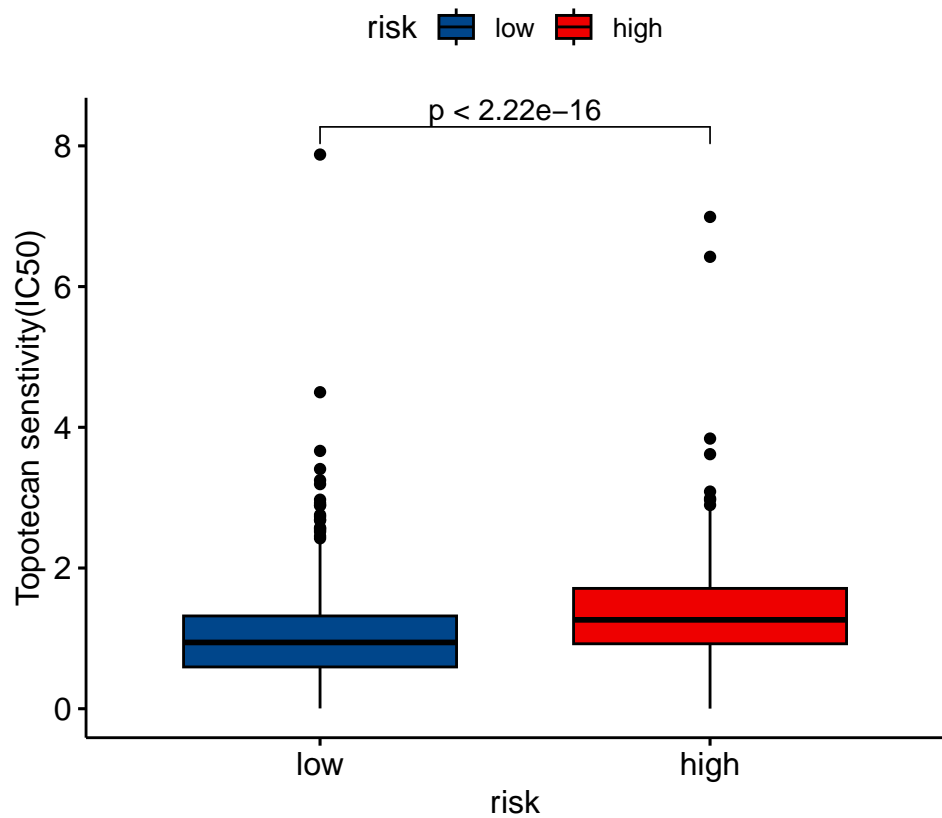

Supplement: Supplementary file 2 — Supplementary file2 (ZIP 3179 KB) [file 10238_2024_1372_MOESM2_ESM.zip › Supplementary Material/Drug1/drugSenstivity.Topotecan.pdf]

risk low high

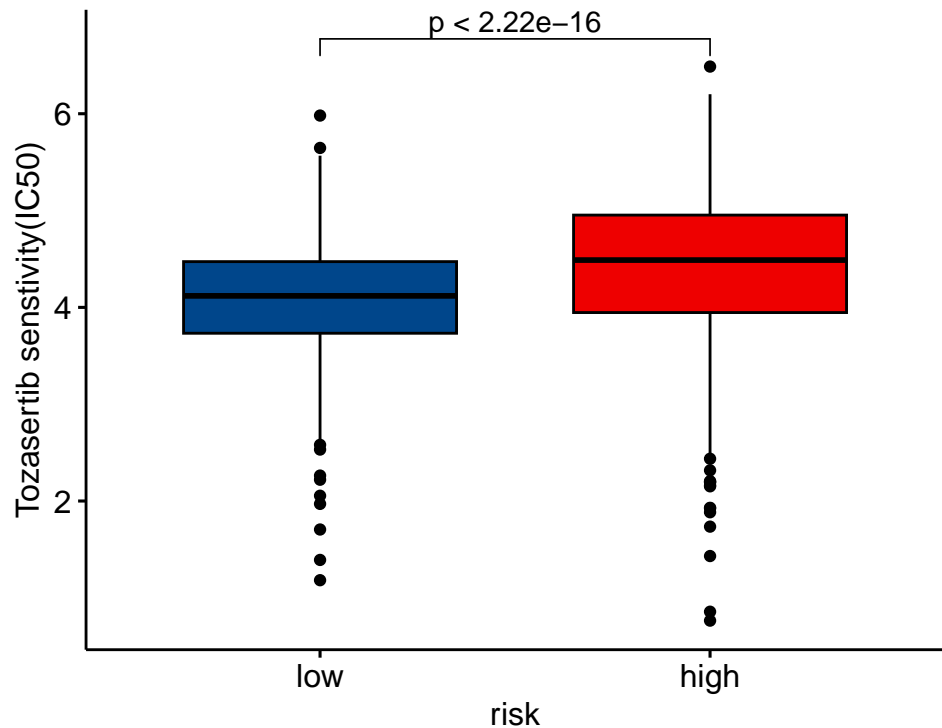

Supplement: Supplementary file 2 — Supplementary file2 (ZIP 3179 KB) [file 10238_2024_1372_MOESM2_ESM.zip › Supplementary Material/Drug1/drugSenstivity.Tozasertib.pdf]

risk 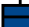 low 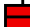 high

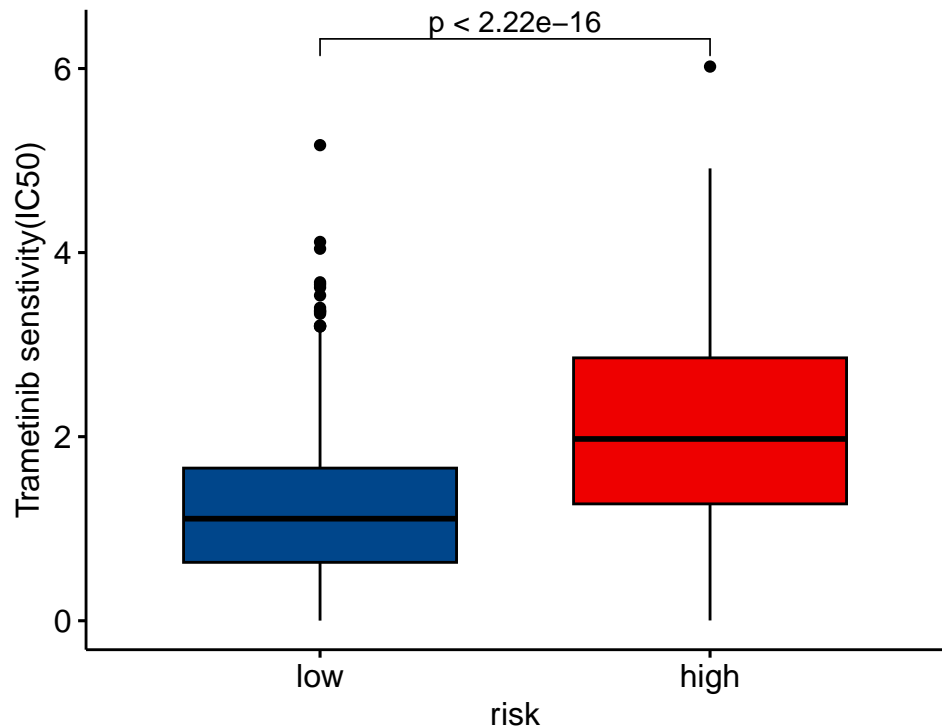

Supplement: Supplementary file 2 — Supplementary file2 (ZIP 3179 KB) [file 10238_2024_1372_MOESM2_ESM.zip › Supplementary Material/Drug1/drugSenstivity.Trametinib.pdf]
