# Supplementary material for: The prognostic genes model of breast cancer drug resistance based on single-cell sequencing analysis and transcriptome analysis
Source: Clin Exp Med. 2024 May 25;24(1):113. doi: 10.1007/s10238-024-01372-6 (PMC11127859; doi:10.1007/s10238-024-01372-6)
Supplement: Supplementary file 2 — Supplementary file2 (ZIP 3179 KB) [file 10238_2024_1372_MOESM2_ESM.zip › Supplementary Material/Drug2/drugSenstivity.PF-4708671.pdf]

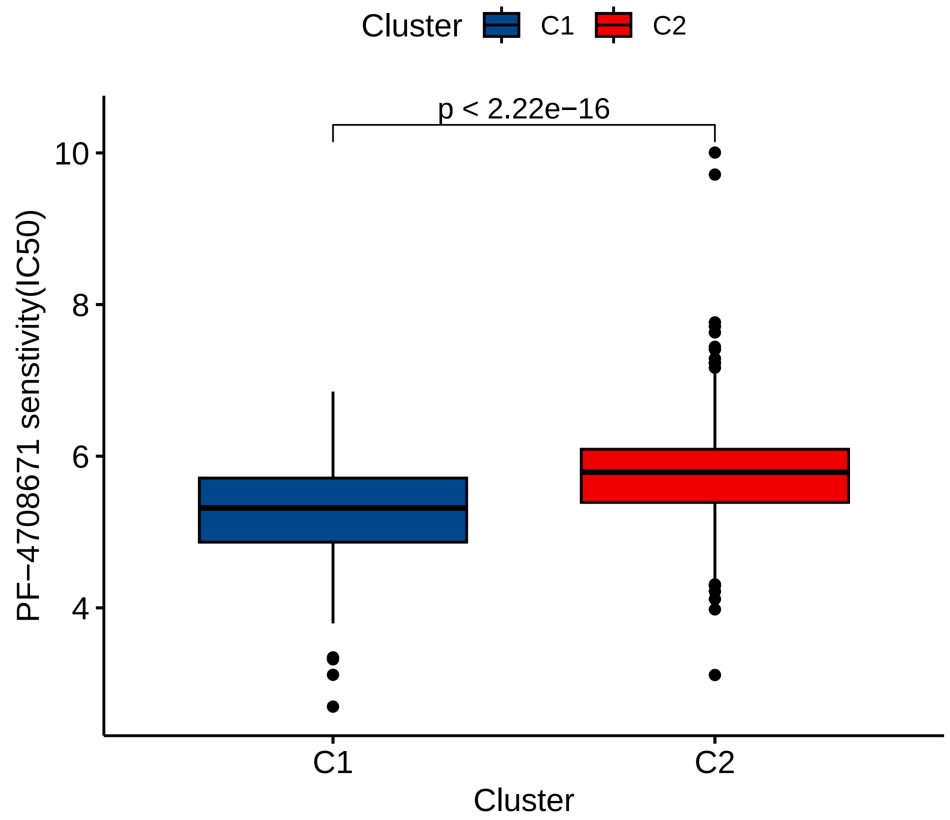

Cluster
C1
C2
p < 2.22e−16
10
8
6
4
PF−4708671 senstivity(IC50)
C1
C2
Cluster
