# Supplementary material for: The prognostic genes model of breast cancer drug resistance based on single-cell sequencing analysis and transcriptome analysis
Source: Clin Exp Med. 2024 May 25;24(1):113. doi: 10.1007/s10238-024-01372-6 (PMC11127859; doi:10.1007/s10238-024-01372-6)
Supplement: Supplementary file 2 — Supplementary file2 (ZIP 3179 KB) [file 10238_2024_1372_MOESM2_ESM.zip › Supplementary Material/Immune/cor.APC_co_inhibition.pdf]

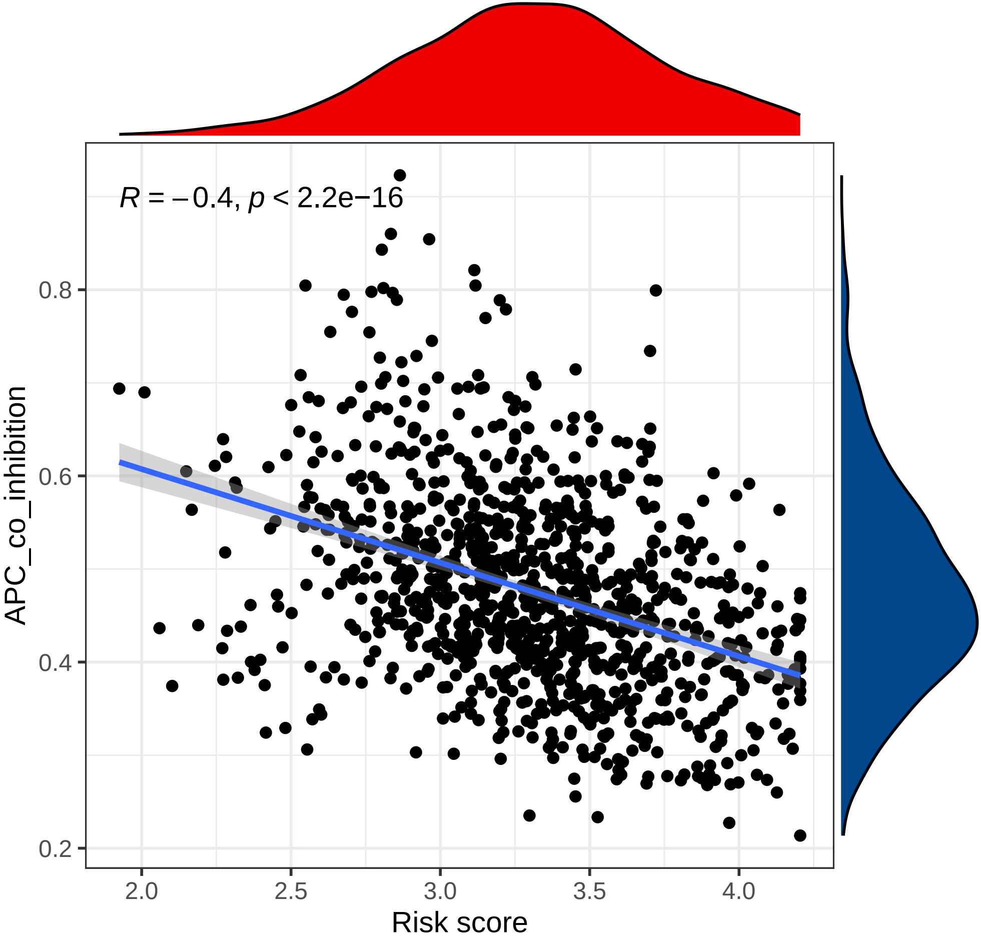

$R = -0.4$, $p < 2.2e-16$
0.8
0.6
0.4
0.2
APC_co_inhibition
2.0
2.5
3.0
3.5
4.0
Risk score
